# Global burden and risk factors of type 2 diabetes mellitus from 1990 to 2021, with forecasts to 2050

**DOI:** 10.3389/fendo.2025.1538143

**Published:** 2025-08-14

**Authors:** Qian Huang, Yang Li, Minggang Yu, Zhizi Lv, Fengyi Lu, Ning Xu, Qingqing Zhang, Jiayun Shen, Jinfeng Zhu, Hua Jiang

**Affiliations:** ^1^ Department of General Practice, Shanghai East Hospital, Tongji University School of Medicine, Shanghai, China; ^2^ Department of Geriatrics, Shanghai East Hospital, Tongji University School of Medicine, Shanghai, China; ^3^ Department of General Practice, Xietu Subdistrict Community Health Service Center, Shanghai, China; ^4^ Shanghai Oriental Clinical College, Nanjing Medical University, Shanghai, China; ^5^ Hunan Provincial Key Laboratory of the Research and Development of Novel Pharmaceutical Preparations, Changsha Medical University, Changsha, China

**Keywords:** global burden, type 2 diabetes mellitus, trend analysis, decomposition analysis, risk factor, forecast, sociodemographic index

## Abstract

**Background:**

Type 2 diabetes mellitus (T2DM) presents a substantial strain on global healthcare systems. This study seeks to offer robust scientific evidence for the effective prevention and management of T2DM globally through a comprehensive analysis of the disease’s burden, trends, and risk factors from 1990 to 2021, as well as future trajectories from 2022 to 2050.

**Methods:**

Data for this study were sourced from the 2021 Global Burden of Disease (GBD) study. T2DM burden was assessed through incidence, prevalence, mortality, and disability-adjusted life years (DALYs), stratified by age, sex, sociodemographic index (SDI), 21 GBD regions, and 204 countries/territories. Trends from 1990 to 2021 were quantified by estimating annual percentage changes. Decomposition analysis identified the primary population-level drivers of T2DM burden changes. The population attributable fraction assessed the contribution of risk factors to the T2DM burden over the past 30 years, while the Bayesian age–period–cohort model was employed to predict the future burden driven by risk factors.

**Results:**

In 2021, T2DM affected 506.0 million individuals, with 23.9 million new cases, 1.6 million deaths, and 75.3 million DALYs. Between 1990 and 2021, both absolute and relative burdens of T2DM increased, particularly among males, older adults, and individuals under 40. Regions with higher SDI generally exhibited higher age-standardized incidence and prevalence rates, while those with lower SDI had elevated age-standardized mortality and DALY rates. Oceania stood out as an exception, with the highest relative burdens across all four indicators, most notably in the Marshall Islands and Fiji. The increases in incidence, DALYs, and prevalence were predominantly driven by population growth and epidemiological shifts, with aging contributing significantly to the rise in mortality. Elevated fasting plasma glucose, body mass index (BMI), and particulate pollution were major contributors to higher T2DM-related mortality and DALY rates. By 2050, high BMI, alcohol consumption, and sugary beverages are anticipated to increasingly influence the T2DM burden.

**Conclusion:**

Focused, preventive interventions targeting key risk factors in high-burden groups can effectively reduce the global T2DM burden.

## 1 Introduction

Diabetes is the most prevalent chronic metabolic disorder, marked by persistent hyperglycemia. As the condition progresses, it can lead to a range of disabling or even life-threatening complications (1). The International Diabetes Federation reports that by 2021, approximately 537 million adults worldwide were living with diabetes, with projections suggesting that the number will rise to 783 million by 2045 (2). Type 2 diabetes mellitus (T2DM), accounting for more than 90% of all diabetes cases, is the most common form of the disease (3). T2DM places significant strain on healthcare systems. A recent study in Malaysia found that cardiovascular hospitalization costs associated with T2DM amounted to RM4.8 million ($1.15 million), approximately 30% higher than those for non-T2DM individuals (4). The etiology of T2DM is multifactorial, encompassing factors such as overweight (5), behavioral risks (6, 7), and dietary habits (8, 9). Regular evaluation of the T2DM burden and its risk factors is essential to developing effective prevention and control strategies.

The Global Burden of Disease (GBD) study provides the most comprehensive estimates of disease burden at the global, regional, and national levels. Most studies on the burden of T2DM have relied on data from the GBD 2019 study (10, 11), but these studies often focus on specific risk factors, such as low physical activity (LPA) (10), or target specific populations or regions (12, 13). In contrast, GBD 2021 offers the most current epidemiological insights into the T2DM burden, utilizing advanced standardization techniques for a more comprehensive and systematic assessment of disease trends. It also carries stronger practical relevance for policy and intervention planning. Recent studies have provided global diabetes prevalence data (across all clinical types) from 1990 to 2021, offering valuable support for global diabetes prevention and management (14). However, the latest data on the global and regional T2DM burden, along with the key drivers behind these trends, remain unknown. A detailed and updated assessment of T2DM, including incidence, prevalence, mortality, and disability-adjusted life years (DALYs), is critical for refining diabetes management strategies and optimizing the allocation of healthcare resources.

Decomposition analysis plays a key role in isolating and quantifying the contributions of aging, population growth, and epidemiological changes to the global and regional disease burden, thereby facilitating a better understanding of the evolving patterns of T2DM across different regions (15). However, such an analysis has seldom been included in prior studies on the T2DM burden. Furthermore, most earlier research has concentrated on predicting overall future trends in T2DM burden or assessing the impact of individual risk factors on specific burden categories (14, 16). The future influence of multiple risk factors—particularly those linked to behavioral, environmental, and occupational risks, as well as those exhibiting upward trends—on T2DM mortality and DALY rates remains unclear.

Thus, this study utilized GBD 2021 data to comprehensively evaluate the global and regional disease burden of T2DM, examine temporal trends through multiple indicators, and systematically analyze the primary drivers and attributable risk factors. Additionally, future trends were predicted, offering valuable insights for T2DM policy development and intervention planning.

## 2 Methods

### 2.1 Data source

The original data for this study originates from GBD 2021, which, as an iterative update of GBD 2019, incorporates newly available epidemiological information and refined standardization techniques (17). This updated version provides a more comprehensive and systematic evaluation of the impact of 371 diseases, injuries, and 88 risk factors on global health across 204 countries and territories from 1990 to 2021. The GBD 2021 Disease and Injury Collaborators integrated data from 100,983 sources, including population registries, verbal autopsies, and censuses, to estimate key burden metrics such as global incidence, prevalence, and DALYs, including those for T2DM (18). T2DM-related deaths were estimated using the Cause of Death Ensemble model (14). Meanwhile, the GBD 2021 Risk Factors Collaborators combined data from 54,561 sources to generate 631 risk-outcome pairs by evaluating 88 risk factors and their associated health outcomes (19), providing a crucial dataset for analyzing T2DM-related attributable risk factors. These data can be accessed via the Institute for Health Metrics and Evaluation (IHME) website (http://ghdx.healthdata.org/gbd-results-tool). Additionally, sociodemographic index (SDI) data (20) and population forecast data (21) used in this study were derived from datasets funded by the Bill and Melinda Gates Foundation (BMGF) and are available on the official IHME website.

### 2.2 Definition of T2DM

T2DM cases in GBD 2021 are determined by subtracting type 1 diabetes mellitus (T1DM) estimates from overall diabetes estimates for each age, gender, and region (14). T2DM is identified in GBD 2021 using ICD-10 codes E11–E11.1 and E11.3–E11.9.

### 2.3 Definition, role, and quintile classification of the sociodemographic index value

The SDI serves as a comprehensive indicator reflecting the level of social and economic development in a country or region. It aids health professionals and policymakers in understanding the relationship between population health and economic development (22). SDI ranges from 0 (lowest) to 1 (highest) and is calculated using factors such as the total fertility rate for women under 25 years old (TFU25), the average educational attainment of individuals aged 15 and older (EDU15+), and the per capita lagged distributed income (LDI) (20, 23). Theoretically, regions with an SDI of 0 represent the lowest socioeconomic development related to health, while those with an SDI of 1 represent the highest (20). According to GBD 2021, SDI is categorized into five levels: high (0.81), high-middle (0.71–0.81), middle (0.62–0.71), low-middle (0.47–0.62), and low (< 0.47).

### 2.4 Decomposition analysis

The decomposition method proposed by Das Gupta (24) was utilized to analyze the factors contributing to changes in the T2DM burden from 1990 to 2021. This method categorizes the influencing factors into three key components: aging, population growth, and epidemiological changes. Following previous research (25), the relevant decomposition formula for prevalence analysis was applied, which is also applicable to the analysis of the other three burden indicators:


$$\text{PREVALENCE }{\text{a}}_{\text{y}},{\text{p}}_{\text{y}},{\text{e}}_{\text{y}}\;=\;\sum_{i=1}^{16}\left({\text{a}}_{\text{i},\text{y}}\times {\text{p}}_{y}\times {\text{e}}_{\text{i},\text{y}}\right)$$

In this context, PREVALENCE *a_y_,p_y_,e_y_* represents the total prevalence calculated for a specific year *y*, based on the age structure (*a_y_*), total population (*p_y_*), and prevalence rates (*e_y_*). Here, *a_i,y_* and *e_i,y_* denote the population proportion and prevalence rate for age group *i* in year *y*, respectively. The impact of individual factors on total prevalence can be analyzed by isolating these factors while holding the other variables constant.

### 2.5 Attributable risk factors and population attributable fraction

Seventeen attributable risk factors (**Supplementary Table S1**) for the burden of T2DM can be explored through the data visualization tool (https://vizhub.healthdata.org/gbd-compare/). The population attributable fraction (PAF) is used to quantify the proportion of the T2DM burden that could be reduced if exposure to specific attributable risk factors were minimized. The larger the PAF, the greater the contribution of a risk factor to T2DM. Comparing the PAFs of various risk factors in 2021 and evaluating their dynamic trends from 1990 to 2021 are critical for accurately identifying key intervention areas and formulating evidence-based health policies.

### 2.6 Impact of attributable risk factors on the future burden of T2DM

Building on previous research (26, 27), this study employed a Bayesian age–period–cohort (BAPC) model to predict trends in age-standardized mortality rates (ASMR) and age-standardized DALY rates (ASDR) related to T2DM, driven by significant attributable risk factors over the next 30 years. To address the inherent wide prediction intervals of the BAPC model and the complex convergence issues arising from the Markov Chain Monte Carlo (MCMC) sampling method, the Integrated Nested Laplace Approximation (INLA) algorithm was incorporated (28).

### 2.7 Statistical analysis

This study quantified the T2DM burden in GBD 2021 by analyzing the number of cases, percentage changes, and age-standardized rates (ASRs) per 100,000, along with 95% uncertainty intervals (UI) for four indicators: incidence, prevalence, DALYs, and deaths. To assess trends in ASRs from 1990 to 2021, the estimated annual percentage change (EAPC) and its 95% confidence interval (CI) were used. The EAPC was calculated from the slope (*β*) of the linear regression model (*y* = *α* + *βx* + *ϵ*), where *y* represents ln(ASR), *x* is the calendar year, and *ϵ* is the error term. The EAPC was then determined as 100 × [exp(*β*) − 1]. The 95% CI of the EAPC was calculated using the standard error derived from the log-linear regression. If both the EAPC and its 95% CI were greater than 0, this indicated an increasing trend in ASRs; if both were below 0, a decreasing trend was observed (10). The correlation between SDI and ASRs for each region and country was calculated using the Spearman correlation coefficient. All calculations were conducted using R software (version 4.4.1).

## 3 Results

### 3.1 Global burden and trends of T2DM

In 2021, approximately 506.0 million (95% UI: 470.0 to 545.3) people were affected by T2DM globally, representing a 290.5% increase from 1990 (**Supplementary Data Sheet 1**). The age-standardized prevalence rate (ASPR) rose from 3,023.8 to 5,885.4 per 100,000 population, with an EAPC of 2.12 (95% CI: 2.08 to 2.16). The number of T2DM incidence cases in 2021 was 23.9 million (95% UI: 22.1 to 25.8) people, reflecting a 220.6% increase from 1990 (**Supplementary Data Sheet 1**). The age-standardized incidence rate (ASIR) increased from 162.0 to 280.3 per 100,000 population, with an EAPC of 1.74 (95% CI: 1.72 to 1.76). T2DM-related deaths surged by 154.3%, reaching 1.6 million (95% UI: 1.5 to 1.7) people in 2021 (**Supplementary Data Sheet 1**). The ASMR was 19.0 (95% UI: 17.6 to 20.2) per 100,000 population, with an EAPC of 0.21 (95% CI: 0.15 to 0.27). Global DALYs due to T2DM increased by 202.2%, totaling 75.3 million (95% UI: 63.5 to 90.3) years in 2021 (**Supplementary Data Sheet 1**). The ASDR was 871.8 years (95% UI: 735.1 to 1,044.8) per 100,000 population, with an EAPC of 1.04 (95% CI: 1.00 to 1.08). Overall, the global burden of T2DM has significantly escalated over the past 30 years.

### 3.2 Gender and age patterns in the global burden and trends of T2DM

**Figure 1:**
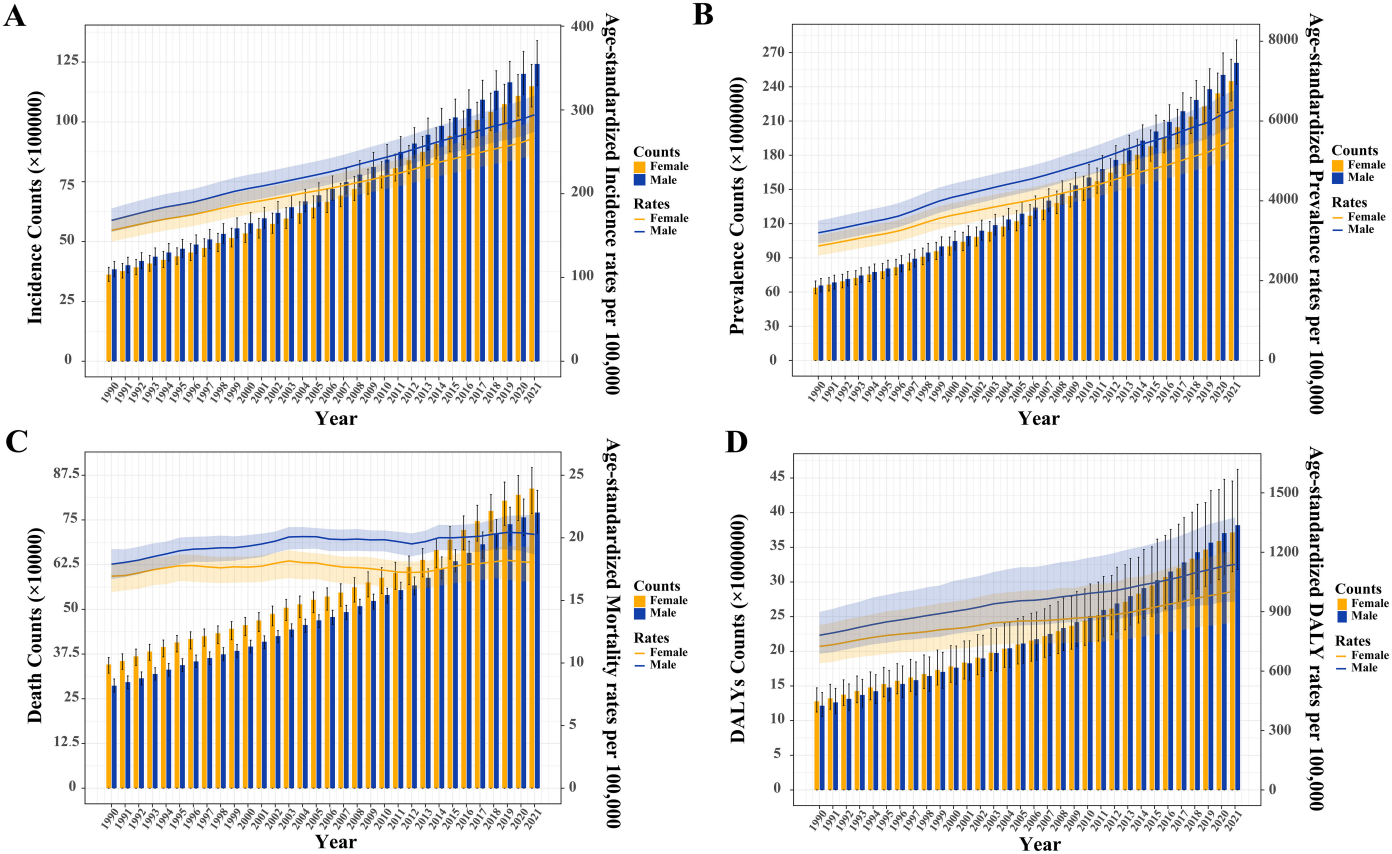
Trends in the absolute and relative burden of type 2 diabetes mellitus worldwide by sex (1990–2021). **(A)** Incidence counts and age-standardized incidence rates. **(B)** Prevalence counts and age-standardized prevalence rates. **(C)** Death counts and age-standardized mortality rates. **(D)** Disability-adjusted life years (DALYs) and age-standardized DALY rates.

From 1990 to 2021, the incidence and prevalence, ASIR, ASPR, ASMR, and ASDR of T2DM were consistently higher in men than in women worldwide (**Supplementary Data Sheet 1**; **Figures 1A–D**). However, the number of T2DM-related deaths among women surpassed that of men (**Figure 1C**).

**Figure 2:**
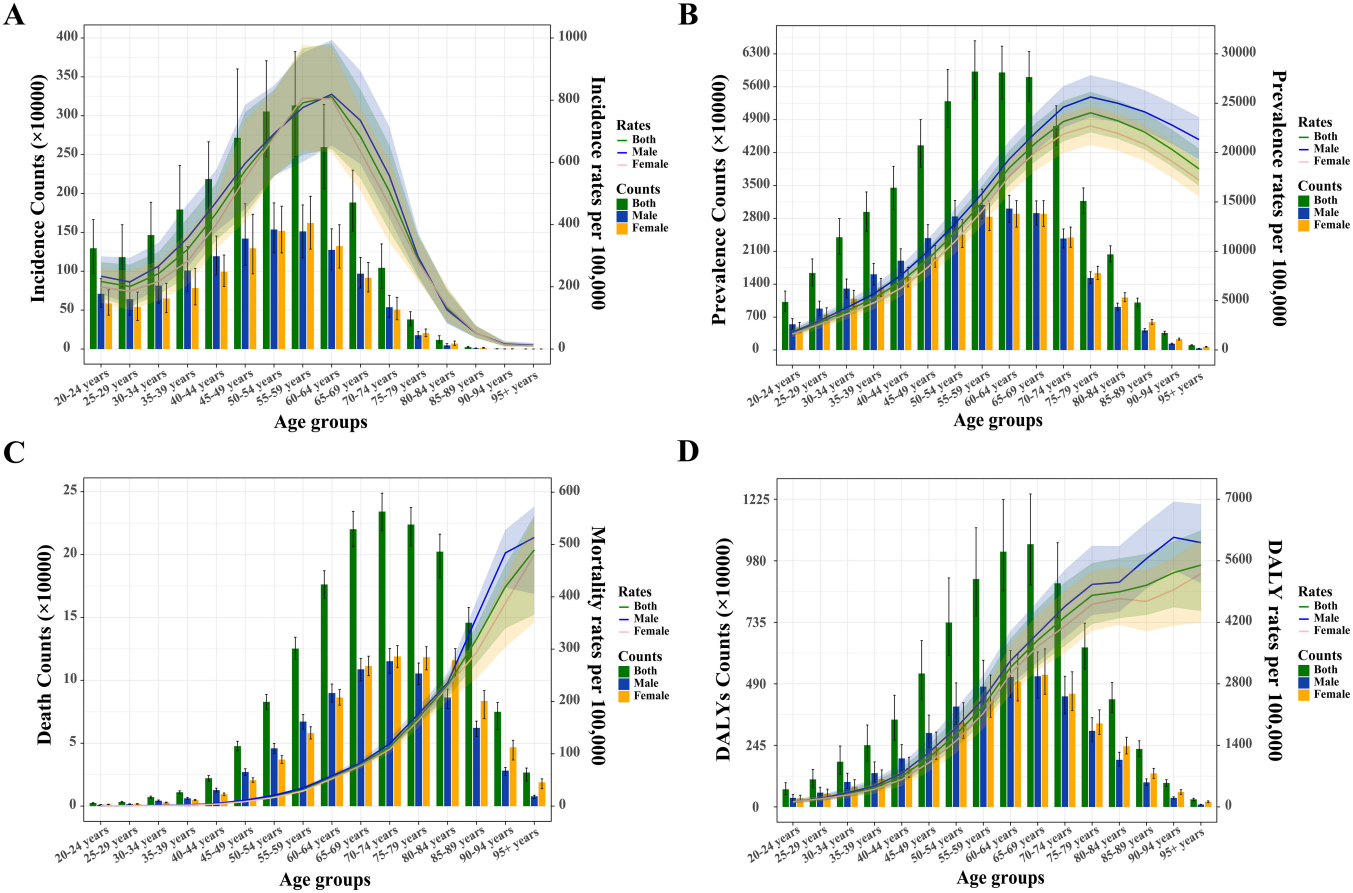
Absolute and relative burden of type 2 diabetes mellitus among adults aged 20 and over by sex in 2021. **(A)** Incidence counts and age-specific incidence rates. **(B)** Prevalence counts and age-specific prevalence rates. **(C)** Death counts and age-specific mortality rates. **(D)** Disability-adjusted life years (DALYs) and age-specific DALY rates.

In 2021, the 55–59 age group had the highest global incidence and prevalence, while the incidence rate peaked in the 60–64 age group and the prevalence rate in the 75–79 age group (**Figure 2**). Both global and sex-stratified T2DM death counts reached their highest in the 70–74 age group, while DALY counts peaked at ages 65-69 (**Figure 2**). From age 65–69 onward, the number of deaths and DALYs in women exceeded those in men. T2DM DALY rates, both globally and by gender, increased with age. **Supplementary Figure S1** illustrates the temporal trends in T2DM burden across age groups. From 1990 to 2021, the greatest increases in incidence, prevalence, death, and DALYs occurred in the middle-aged and older adult populations, particularly in those aged 95 and older (**Supplementary Tables S2–S5**). The 20–45 age group experienced the largest increase in the rates of these indicators.

### 3.3 Variations in the global burden and trends of T2DM across different regions and nations

#### 3.3.1 Analysis by SDI regions

From 1990 to 2021, all T2DM burden indicators across the five SDI regions, except for ASMR, demonstrated a marked increase (**Supplementary Data Sheet 1**; **Supplementary Figures S2A–H**). The middle-SDI region consistently exhibited the highest incidence, prevalence, mortality, and DALYs relative to other regions (**Supplementary Figures S2A, C, E, G**). The most significant growth in these metrics occurred in low-middle-SDI regions, with increases of 313.3% in incidence, 376.7% in prevalence, 229.7% in mortality, and 260.3% in DALYs (**Supplementary Data Sheet 1**). Since 2001 and 2012, the high-SDI region’s ASIR and ASPR surpassed those of other regions, reaching 335.0 (95% UI: 314.7–358.3) per 100,000 population and 6,575.4 (95% UI: 6,168.3–7,011.1) per 100,000 population by 2021, respectively, marking the highest increases (EAPC of 2.37 for ASIR and 2.71 for ASPR) (**Supplementary Data Sheet 1**; **Supplementary Figure S2**). The ASDR in the low-middle-SDI region overtook that of the low-SDI region in 2016, rising to 1,209.6 (95% UI: 1,045.3–1,411.3) per 100,000 population by 2021, with an EAPC of 1.42 (**Supplementary Data Sheet 1**; **Supplementary Figure S2**). ASMR trends varied across regions, with declines observed in high- and middle-high-SDI regions and increases in others.

#### 3.3.2 Analysis of 21 GBD regions

**Figure 3:**
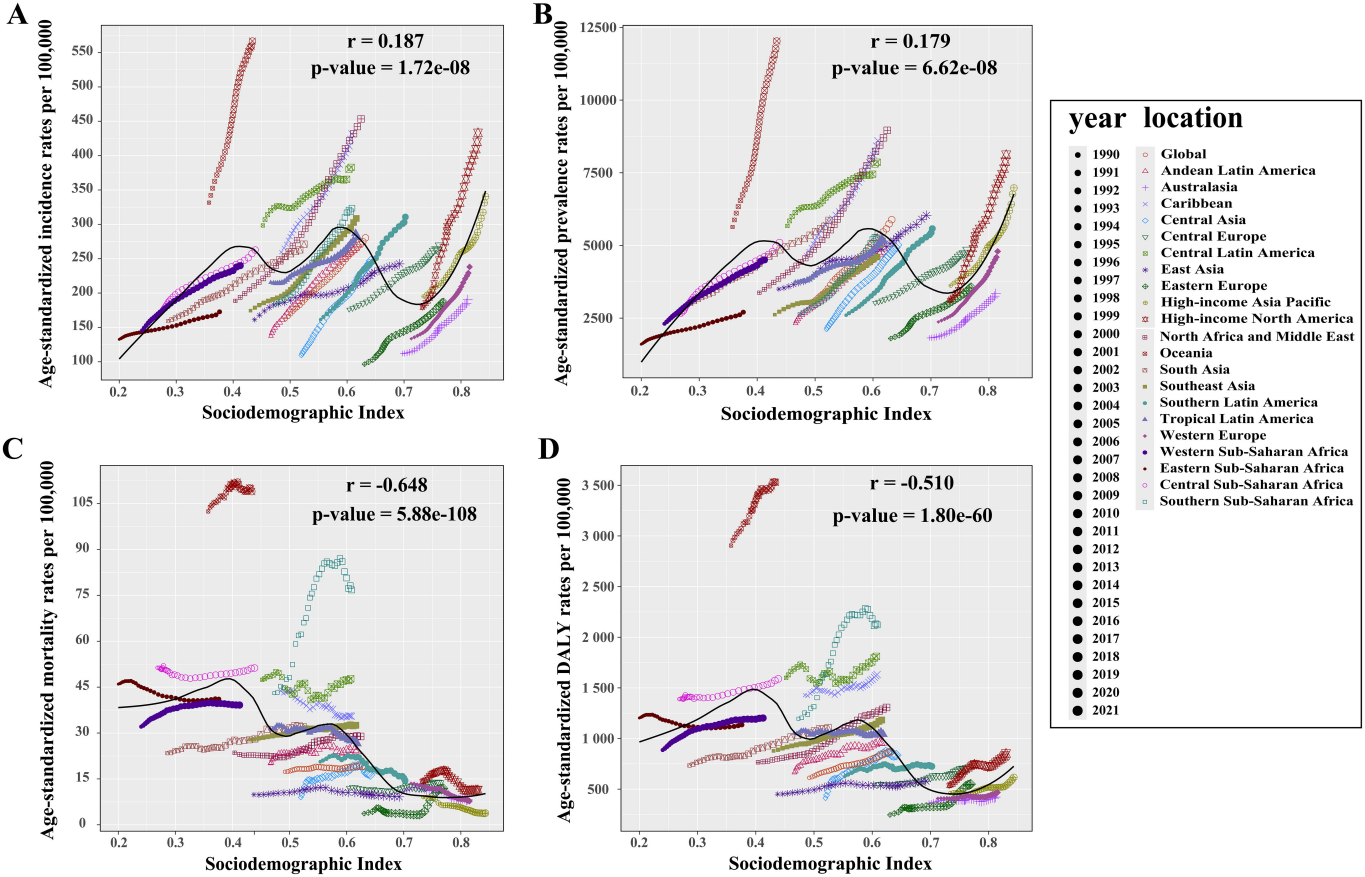
Relationship between the relative burden of type 2 diabetes mellitus and SDI value in 21 GBD regions. **(A)** Incidence rate vs. SDI. **(B)** Prevalence rate vs. SDI. **(C)** Mortality rate vs. SDI. **(D)** DALY rate vs. SDI. An *r* value greater than zero indicates a positive correlation between age-standardized rates and SDI, while an *r* value less than zero suggests a negative correlation. A *p*-value below 0.05 confirms the statistical significance of these correlations. SDI, sociodemographic index.

In the 21 GBD regions, strong correlations were found between SDI values and T2DM’s ASIR, ASPR, ASMR, and ASDR (all *p* < 0.001, **Figures 3A–D**), indicating a clear association between T2DM burden and socioeconomic development levels. Overall, SDI values positively correlated with ASIR (*r* = 0.187) and ASPR (*r* = 0.179), but negatively with ASMR (*r* = − 0.648) and ASDR (*r* = − 0.510). However, Oceania, a low-SDI region, exhibited the highest relative T2DM burden globally, with ASIR, ASPR, ASMR, and ASDR rates of 566.7 (95% UI: 534.6 to 599.2), 12,031.8 (95% UI: 11,274.1 to 12,881.7), 108.9 (95% UI: 92.8 to 130.5), and 3,529.2 (95% UI: 3,015.9 to 4,180.9) per 100,000 population, respectively (**Supplementary Data Sheet 1**). Between 1990 and 2021, High-income North America saw the largest increases in ASIR (EAPC: 3.01) and ASPR (EAPC: 3.28), while Eastern Europe experienced the largest rise in ASMR (EAPC: 2.73) and Southern Sub-Saharan Africa in ASDR (EAPC: 2.18) (**Supplementary Data Sheet 1**). In contrast, the high-income Asia Pacific recorded the greatest decrease in ASMR (EAPC: − 2.91), and Eastern Sub-Saharan Africa in ASDR (EAPC: − 0.41) (**Supplementary Data Sheet 1**).

#### 3.3.3 National-level analysis

**Figure 4:**
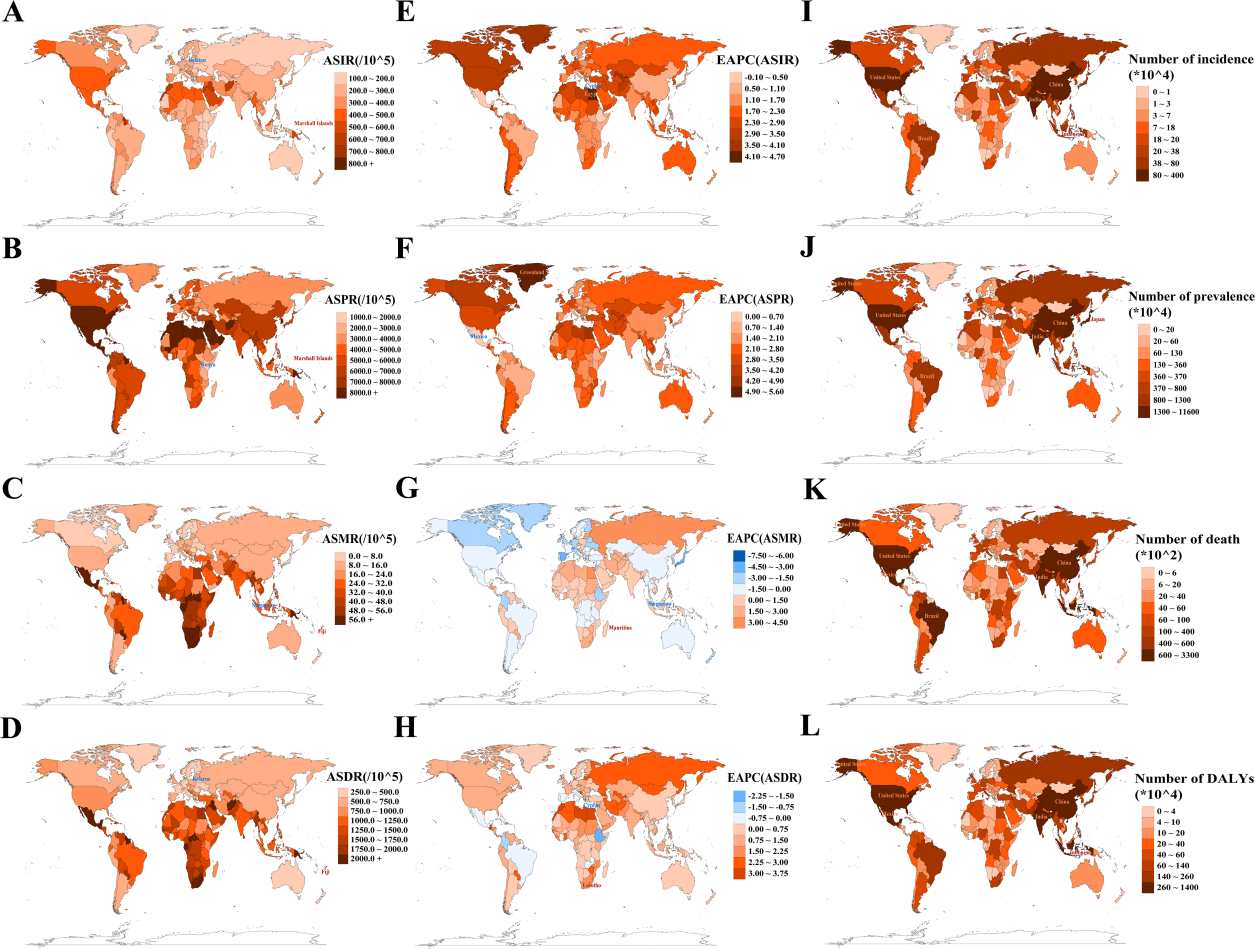
Analysis of absolute and relative burden, as well as trends, of type 2 diabetes mellitus in 204 countries/territories. **(A**, **E)** ASIR and its EAPC; **(B**, **F)** ASPR and its EAPC; **(C**, **G)** ASMR and its EAPC; **(D**, **H)** ASDR and its EAPC. Maps use red markers to indicate the highest burden/fastest growth, and blue markers to indicate the lowest burden/fastest decline trends. **(I**–**L)** Incidence/prevalence/death/DALY counts. Maps highlight the top five countries by absolute burden. ASIR/ASPR/ASMR/ASDR, age-standardized incidence/prevalence/mortality/DALY rate; EAPC, estimated annual percentage change; DALYs, disability-adjusted life years.

In 2021, among 204 countries and territories, the Marshall Islands recorded the highest ASIR at 877.7 (95% UI: 819.3 to 947.0) per 100,000 population and ASPR at 21,942.7 (95% UI: 20,398.2 to 23,914.2) per 100,000 population, while Belarus reported the lowest ASIR (129.5 per 100,000 population [95% UI: 116.4 to 141.7]) and Kenya the lowest ASPR (1,858.5 per 100,000 population [95% UI: 1,674.3 to 2,051.8]) (**Figure 4A, B**; **Supplementary Tables S6**, **S7**). Fiji had the highest ASMR at 265.2 per 100,000 population (95% UI: 213.0 to 328.3) and ASDR at 7,322.7 per 100,000 population (95% UI: 5,942.6 to 9,076.0), whereas Singapore reported the lowest ASMR (2.0 per 100,000 population [95% UI: 1.7 to 2.2]) and Belarus had the lowest ASDR (307.0 per 100,000 population [95% UI: 237.1 to 397.0]) (**Figures 4C, D**; **Supplementary Tables S8**, **S9**). From 1990 to 2021, Egypt, Greenland, Mauritius, and Lesotho experienced the largest increases in ASIR, ASPR, ASMR, and ASDR, respectively, while Cyprus was the only country to report a decline in ASIR and the largest reduction in ASDR (**Figures 4E–H**; **Supplementary Tables S6–S9**). Singapore demonstrated the most significant decrease in ASMR. In 2021, China, India, and the USA significantly outpaced other countries in the total number of T2DM cases across all indicators (**Figures 4I–L**; **Supplementary Tables S6–S9**), highlighting the major challenges these countries face in addressing T2DM.

### 3.4 Decomposition analysis of T2DM burden

From 1990 to 2021, all four absolute burden indicators for T2DM globally increased, with the highest increases in incidence, deaths, and DALYs observed in the middle-SDI region, and the largest increase in prevalence in the low-middle-SDI region.

**Figure 5:**
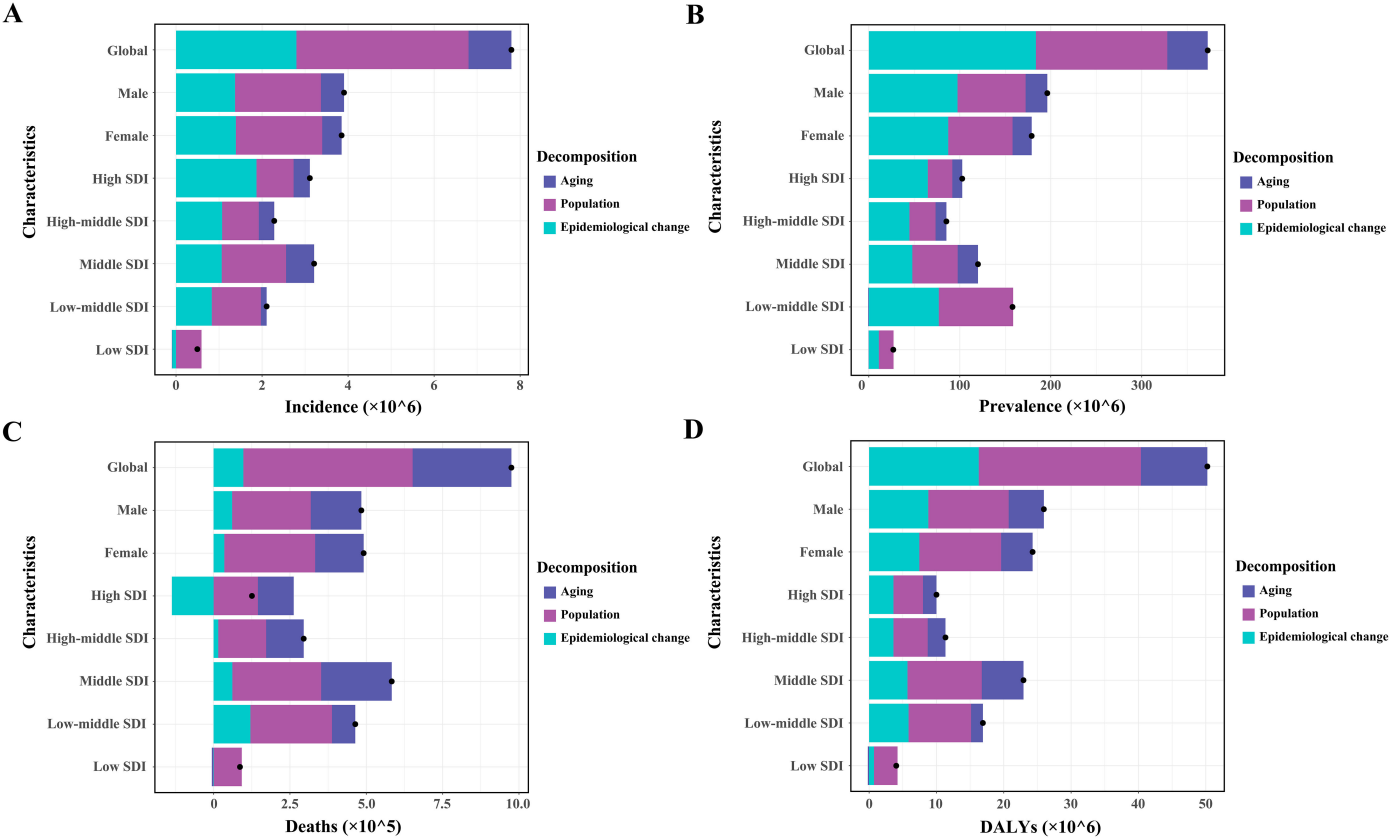
Patterns of change in incidence **(A)**, prevalence **(B)**, deaths **(C)**, and DALYs **(D)** of type 2 diabetes mellitus, driven by aging, population growth, and epidemiological changes from 1990 to 2021, shown globally, by sex, and across various SDI regions. Black dots represent the overall change attributed to these components. Positive values indicate contributions to an increase in T2DM burden, whereas negative values indicate contributions to its decrease. SDI, sociodemographic index.

Globally, population growth was the primary contributor to the increasing burden of incidence, deaths, and DALYs, accounting for 51.31%, 56.84%, and 47.94%, respectively (**Figures 5A, C, D**; **Supplementary Tables S10**, **S12**, **S13**). The impact of population growth was consistent across genders but varied by SDI region, being most pronounced in low-SDI regions, with contributions of 119.52%, 59.96%, 106.75%, and 86.79% for incidence, prevalence, deaths, and DALYs, respectively (**Figures 5A–D**; **Supplementary Tables S10**-**S13**). Epidemiological changes contributed the largest share to global prevalence (49.31%), with a similar distribution across genders and peaking in high-SDI regions (63.16%) (**Figure 5B**; **Supplementary Table S11**). While this factor had the least impact on global mortality, it substantially reduced T2DM mortality in high-SDI regions (− 108.87%). Aging contributed minimally to global incidence, prevalence, and DALYs, showing similar patterns across genders and SDI regions (**Figures 5A, B, D**; **Supplementary Tables S10**, **S11**, **S13**). However, it played a significant role in the rise of global T2DM deaths, particularly in high-SDI regions (93.37%) (**Figure 5C**; **Supplementary Table S12**).

The contributions of these three determinants to changes in the T2DM burden varied significantly across the 21 GBD regions and 204 countries (**Supplementary Tables S10**-**S13**). Population growth was the main driver of the increased T2DM burden in the Oceania region and India, while epidemiological changes were the dominant factor in the USA. In China, the T2DM burden increase was driven by a combination of all three determinants.

### 3.5 Analysis of attributable risk factors for T2DM

**Figure 6:**
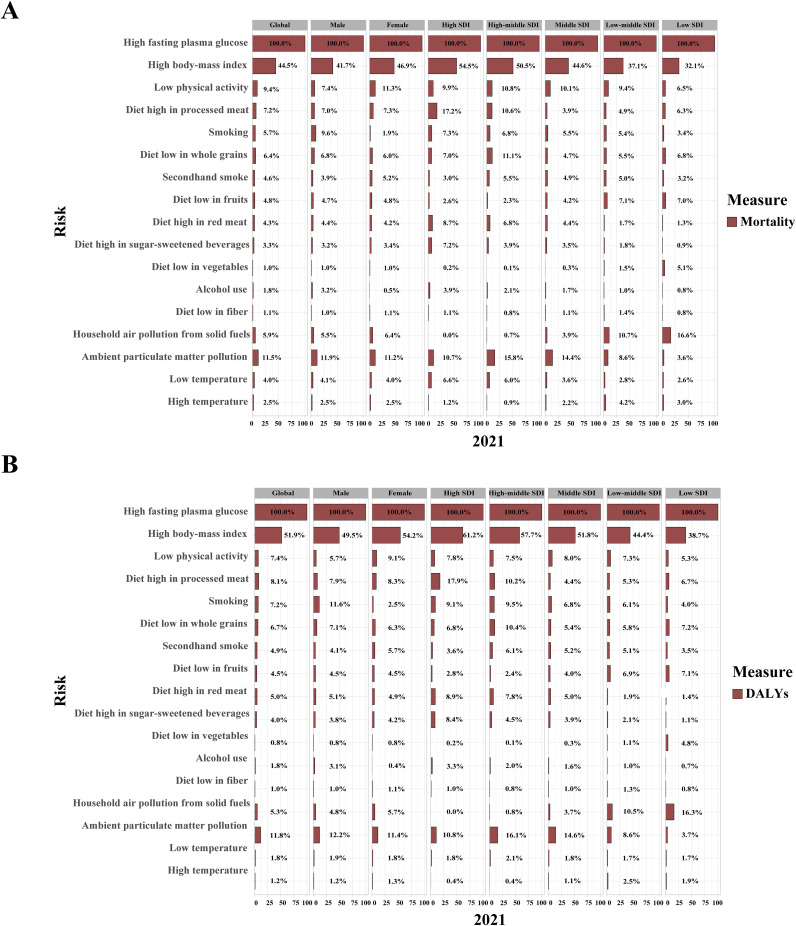
Contribution of 17 risk factors to age-standardized mortality **(A)** and DALY rates **(B)** related to type 2 diabetes mellitus, by sex and SDI regions in 2021. DALYs, disability-adjusted life years; SDI, sociodemographic index.

Over the past 30 years, metabolic risk factors have been the primary contributors to T2DM-related ASMR and ASDR, followed by behavioral and environmental/occupational risks (**Supplementary Figure S3A, B**). High fasting plasma glucose (FPG) has remained the leading metabolic risk factor for T2DM-related ASMR (PAF, 100.0% [95% UI: 99.8 to 100.1]) and ASDR (PAF, 100.0% [95% UI: 99.9 to 100.1]) globally (**Figures 6A, B**; **Supplementary Tables S14**, **S15**). High body mass index (BMI) was responsible for 44.5% of T2DM-related ASMR and 51.9% of ASDR, with a more pronounced effect in women than in men. Its impact also increased with higher SDI quintiles (**Figures 6A, B**; **Supplementary Tables S14**, **S15**). From 1990 to 2021, the contribution of high BMI to both ASMR and ASDR showed an increasing trend in both genders (**Supplementary Tables S14**, **S15**).

Key behavioral risk factors for ASMR and ASDR in 2021 included LPA, high-processed meat diets, low whole grain diets, and smoking, each with gender-specific effects and varying contributions across SDI regions (**Figures 6A, B**). LPA and a diet high in processed meat had a greater impact on women, while a low whole grain diet and smoking were more influential in men. In high and high-middle SDI regions, smoking and dietary risks such as processed meat, red meat, and sugar-sweetened beverages (SSBs) contributed more to ASMR and ASDR, while diets low in fruits and vegetables were more impactful in low SDI regions. The contributions of high SSB consumption and alcohol use to ASMR and ASDR increased globally from 1990 to 2021, particularly in low SDI regions (**Supplementary Tables S14**, **S15**).

Ambient particulate matter (PM) pollution was the leading environmental/occupational risk factor for T2DM-related ASMR (PAF, 11.5% [95% UI: 6.7 to 17.0]) and ASDR (PAF, 11.8% [95% UI: 6.8 to 17.3]) in 2021, with similar effects across genders (**Figure 6A, B**; **Supplementary Tables S14**, **S15**). In high and high-middle SDI regions, ambient PM pollution and low temperatures contributed more to ASMR and ASDR, while in low SDI regions, household air pollution from solid fuels and high temperatures had a greater impact. From 1990 to 2021, the contributions of household air pollution from solid fuels and low temperatures decreased, while the impacts of ambient PM pollution and high temperatures increased (**Supplementary Tables S14**, **S15**).

### 3.6 Prediction of ASMR and ASDR for T2DM by 2050 based on important risk factors

This study identified the critical T2DM risk factors—those that dominate across three major categories or exhibit continuous upward trends from 1990 to 2021—and predicted their future impact using the BAPC model.

**Figure 7:**
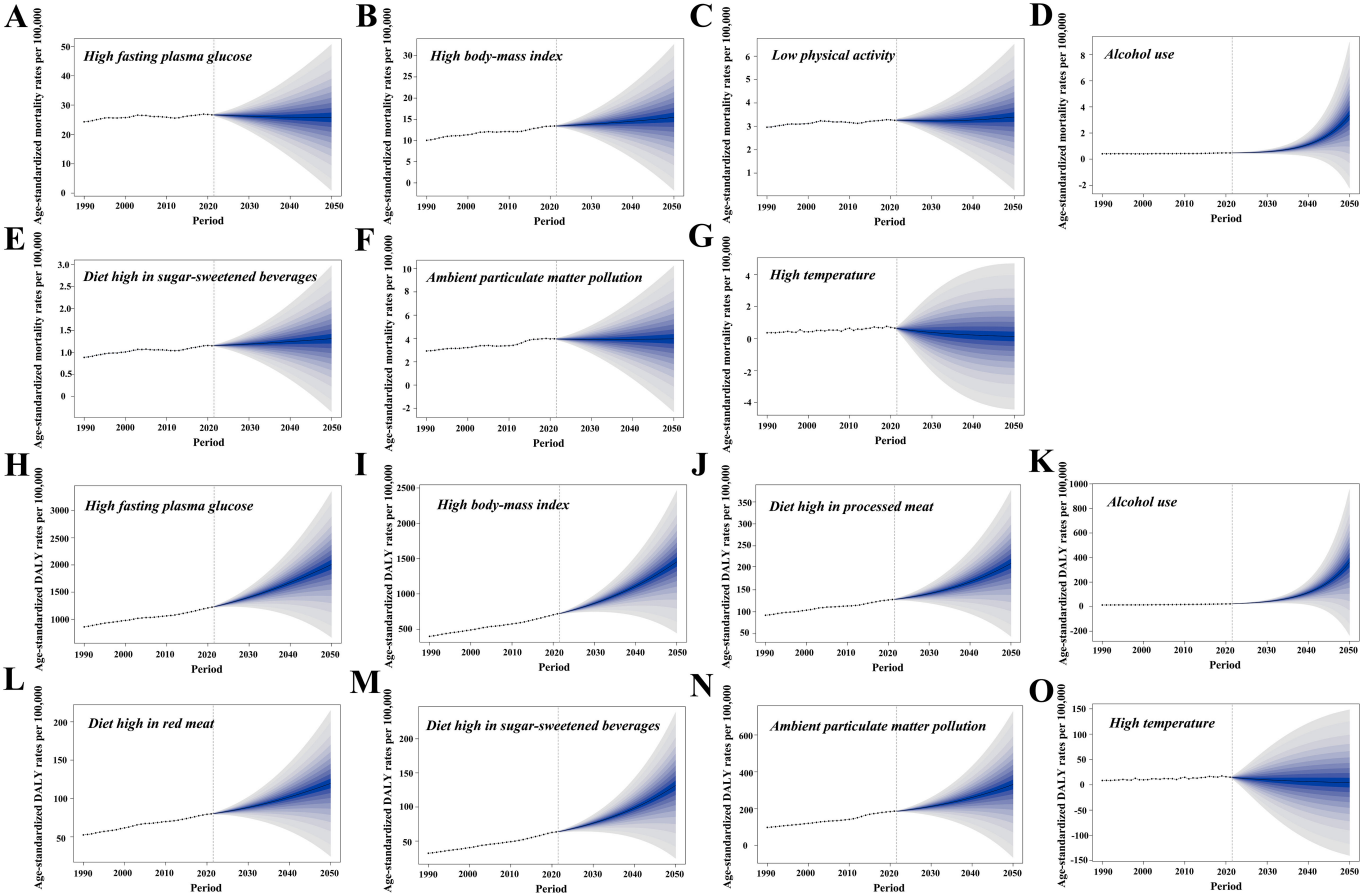
The Bayesian age–period–cohort model was utilized to forecast the age-standardized mortality (ASMR) and DALY rates (ASDR) associated with type 2 diabetes mellitus (T2DM) attributable to various major risk factors by 2050. **(A**–**G)** Contributions of attributable risk factors shaping the future patterns of T2DM-related ASMR changes. **(H**–**O**) Contributions of risk factors to future patterns of T2DM-related ASDR changes. DALYs, disability-adjusted life years.

In 2021, high FPG resulted in a T2DM-related ASMR of 26.7 and an ASDR of 1,222.4 per 100,000 population. The T2DM-related ASDR attributable to high FPG is projected to rise to 2,008.0 per 100,000 population by 2050, while the ASMR is expected to decrease by 3.4% (**Figures 7A, H**). High BMI is anticipated to dramatically increase the T2DM-related ASMR from 13.4 to 15.5 per 100,000 population and the T2DM-related ASDR from 718.1 to 1,454.3 per 100,000 population (**Figures 7B, I**).

In 2021, the T2DM-related ASMR due to LPA was 3.3 per 100,000 population, with a modest increase of 3.0% expected by 2050. In contrast, the T2DM-related ASDR due to high consumption of processed meats in 2021 stood at 127.8 per 100,000 population, with a projected rise of 64.2% by 2050 (**Figures 7C, J**). Alcohol use is forecast to experience the most substantial increase, with T2DM-related ASMR rising by 580.0% to 3.4 per 100,000 population and T2DM-related ASDR increasing by 1,492.0% to 360.0 per 100,000 population (**Figures 7D, K**). Consumption of red meat and SSBs will also contribute to higher ASMR and ASDR, with SSBs expected to raise ASDR by 107.7% (**Figures 7E, L, M**).

In 2021, ambient PM pollution contributed to T2DM-related ASMR and ASDR rates of 4.0 and 186.6 per 100,000 population, respectively. Ambient PM pollution is projected to increase T2DM-related ASDR by 77.5%, while ASMR is expected to remain stable (**Figures 7F, N**). In contrast, high temperatures are expected to further decrease T2DM-related ASMR and ASDR by 2050 (**Figures 7G, O**).

## 4 Discussion

T2DM remains a critical global health issue. The findings of this study reveal significant increases in its absolute burden, relative burden, and both fatal and nonfatal burdens since 1990, underscoring the urgent need for effective early prevention and management strategies.

In line with the GBD 2019 study (29), this study observed that in 2021, the ASIR, ASMR, and ASDR for male patients with T2DM were higher than for female patients. This may be attributed to the earlier diagnosis and lower body fat mass observed in male patients (30). However, biological, psychosocial, behavioral, pathophysiological, and comorbid factors all contribute to the observed sex differences in the T2DM burden (31). Furthermore, this study identified that the number of T2DM-related deaths among women surpassed that of men, potentially linked to the significantly increased relative risk of fatal coronary heart disease associated with diabetes in women (32).

The relative burden of T2DM in 2021 was most pronounced in older populations, though the impact on younger individuals has notably increased over the past three decades. Several factors likely contribute to this trend: urbanization has led to greater consumption of processed and fast foods among younger people, fostering a sedentary lifestyle and rising obesity rates in adolescents and children, which, in turn, drive up T2DM incidence (33–35). Early-onset T2DM, particularly in individuals under 40, often presents with more aggressive disease characteristics, such as heightened insulin resistance and severe beta-cell failure (36). Younger patients are at a heightened risk of earlier cardiovascular complications and increased mortality due to prolonged disease exposure and inadequate blood sugar control, which results in poorer long-term outcomes (37).

Distinct socioeconomic and geographic disparities exist in the burden of T2DM. Globally, in 2021, increasing SDI was associated with higher ASIR and ASPR, while ASMR and ASDR decreased. This trend likely reflects the benefits of increased funding and advanced medical technology in high-SDI regions, which facilitate early detection and treatment, reducing the risk of severe complications. Conversely, low-SDI regions, constrained by financial and technological limitations, face higher rates of undiagnosed cases and poorer outcomes due to inadequate disease management. Notably, Oceania, a low-SDI region, exhibited the highest relative burden across all four indicators, with the Marshall Islands and Fiji standing out, further reinforcing findings from GBD 2019. Faith-based health checks (38) and community participatory screening (39) could enhance early T2DM detection in the Marshall Islands. In Fiji, the rising T2DM prevalence is compounded by poor blood sugar control, low patient awareness, poor treatment adherence, and limited resources, leading to high mortality and hospitalization rates (40). Drawing from Singapore’s experience in strengthening T2DM screening and exploring personalized treatment strategies may provide an effective approach to reducing the T2DM burden in high-burden regions (41, 42).

This decomposition analysis enhances our understanding of the evolving trends in the absolute burden of T2DM across different economic regions. In low-SDI regions, increases in T2DM incidence and DALYs are primarily driven by population growth, followed by epidemiological changes. This outcome reflects the combined challenges of insufficient disease prevention and management amidst a large population base. In contrast, epidemiological changes are the leading contributor to the global rise in T2DM prevalence, especially in high-SDI regions, where rising obesity rates, high-calorie diets, and sedentary behaviors have amplified the economic burden associated with T2DM and its complications (43). Aging, a significant factor, is responsible for the increased mortality from T2DM across all income regions, likely due to the higher risks of fatal diseases in longer-lived patients with T2DM.

Additionally, this study refines the analysis of modifiable risk factors influencing T2DM mortality and DALY rates using GBD 2021, categorizing 17 risk factors into metabolic, behavioral, and environmental/occupational categories. High FPG remains the foremost risk factor for T2DM, crucial for diagnosis, treatment evaluation, and mortality in patients with T2DM, particularly following COVID-19 infection (44–46). High BMI, the second most significant risk, exacerbates the likelihood of heart failure and atrial fibrillation in patients with T2DM (47, 48). It also predicts all-cause and cardiovascular mortality when combined with high HbA1c levels (49). Ambient PM pollution ranks as the third-largest risk, with each 1 μg/m^3^ increase in PM_2.5_ elevating T2DM mortality risk by 3.6% (50). LPA and processed meat intake are key behavioral risks, with LPA reducing macrovascular and microvascular event risks (51), while processed meat consumption, being less than twice a week, is linked to slightly reduced risks of T2DM, cardiovascular diseases, and colorectal cancer (52).

Over the past 30 years, the PAFs of T2DM-related ASMR and ASDR due to alcohol use have risen by 6.4% and 21.7%, respectively. By 2050, alcohol consumption is projected to become the third leading risk factor for T2DM-related ASDR. The stem cell differentiation defects and tissue inflammation caused by ethanol and its metabolites may increase the cancer risk in patients with T2DM, contributing to a worse prognosis (53). High SSB consumption has also significantly increased T2DM risk. In 2020, SSB consumption resulted in 2.2 million T2DM cases and 7,000 deaths in four Latin American and Caribbean countries, accounting for 19% of total cases and 23% of deaths (54). However, implementing SSB taxes has proven effective in reducing the T2DM burden. For instance, after a 10% SSB tax was introduced in Hong Kong, obesity and T2DM prevalence showed significant improvement (55).

Despite these significant findings, some limitations should be acknowledged. GBD 2021 primarily relies on population-level data, which may not fully capture the individual health status of patients with T2DM. Additionally, variations in the definition and classification of T2DM across regions may affect the overall analysis results.

## 5 Conclusion

This study draws on the latest GBD 2021 data to robustly demonstrate the substantial global burden of T2DM and identify key populations in need of focused attention. Moving forward, targeting critical risk factors such as high BMI, alcohol consumption, and SSBs in these populations or regions is anticipated to mitigate the global T2DM burden and enhance the overall quality of life for affected individuals.

## Data Availability

The original contributions presented in the study are included in the article/**Supplementary Material**. Further inquiries can be directed to the corresponding author.
