# Supplementary material for: Global burden and risk factors of type 2 diabetes mellitus from 1990 to 2021, with forecasts to 2050
Source: Front Endocrinol (Lausanne). 2025 Aug 14;16:1538143. doi: 10.3389/fendo.2025.1538143 (PMC12390814; doi:10.3389/fendo.2025.1538143)
Supplement: Supplementary file 2 [file Supplementaryfile1.docx]

Supplementary Material

# Supplementary Figures and Tables

## Supplementary Figures


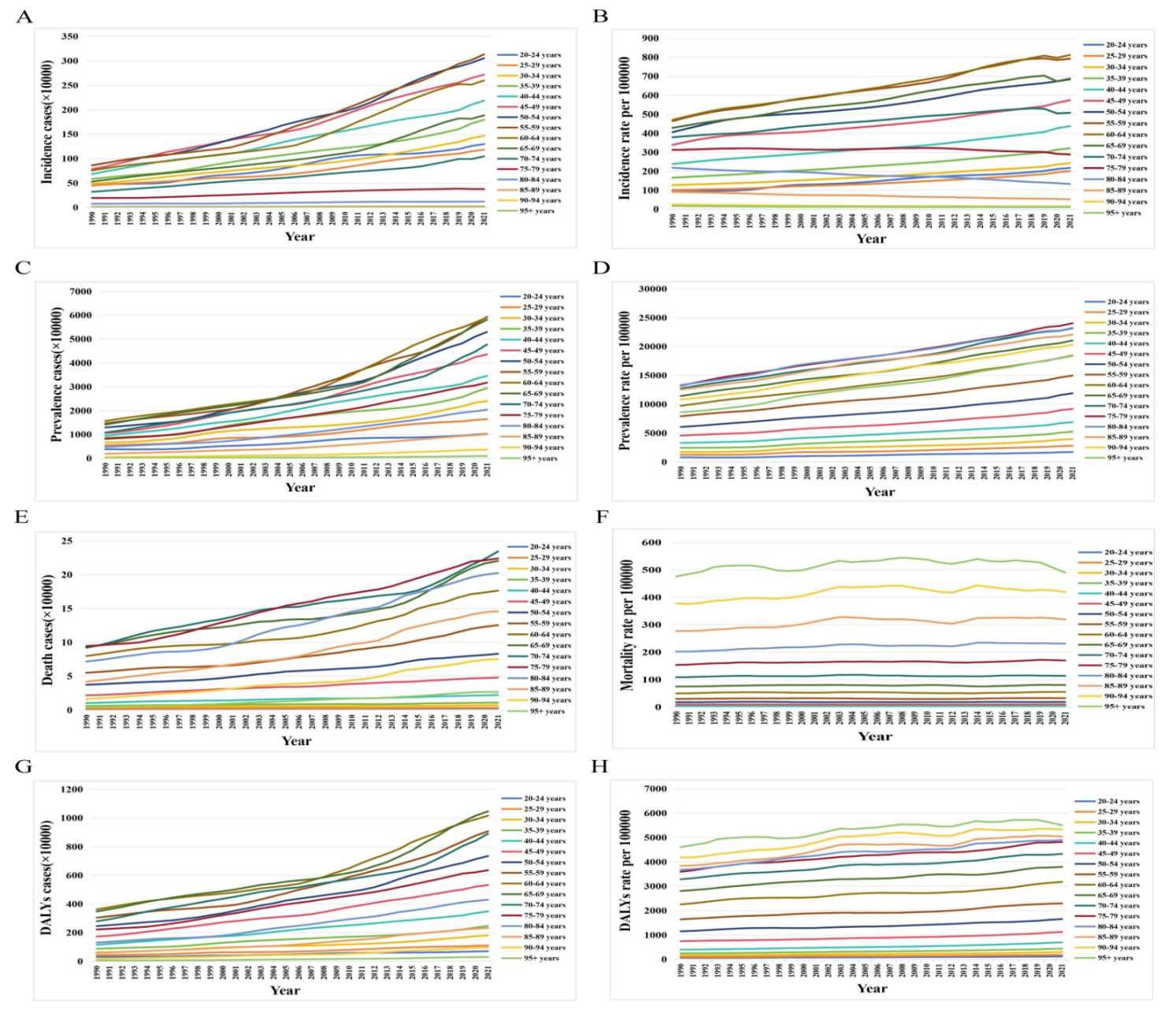


**Supplementary Figure 1. Temporal trends in the burden of type 2 diabetes mellitus in different age groups over 20 years old. (A and B)** Incidence counts and incidence rates for all age groups aged 20 and above from 1990 to 2021, as well as **(C and D)** Prevalence counts and prevalence rates, **(E and F)** Death counts and mortality rates, **(G and H)** DALYs counts and DALYs rates. DALYs, disability-adjusted life years.


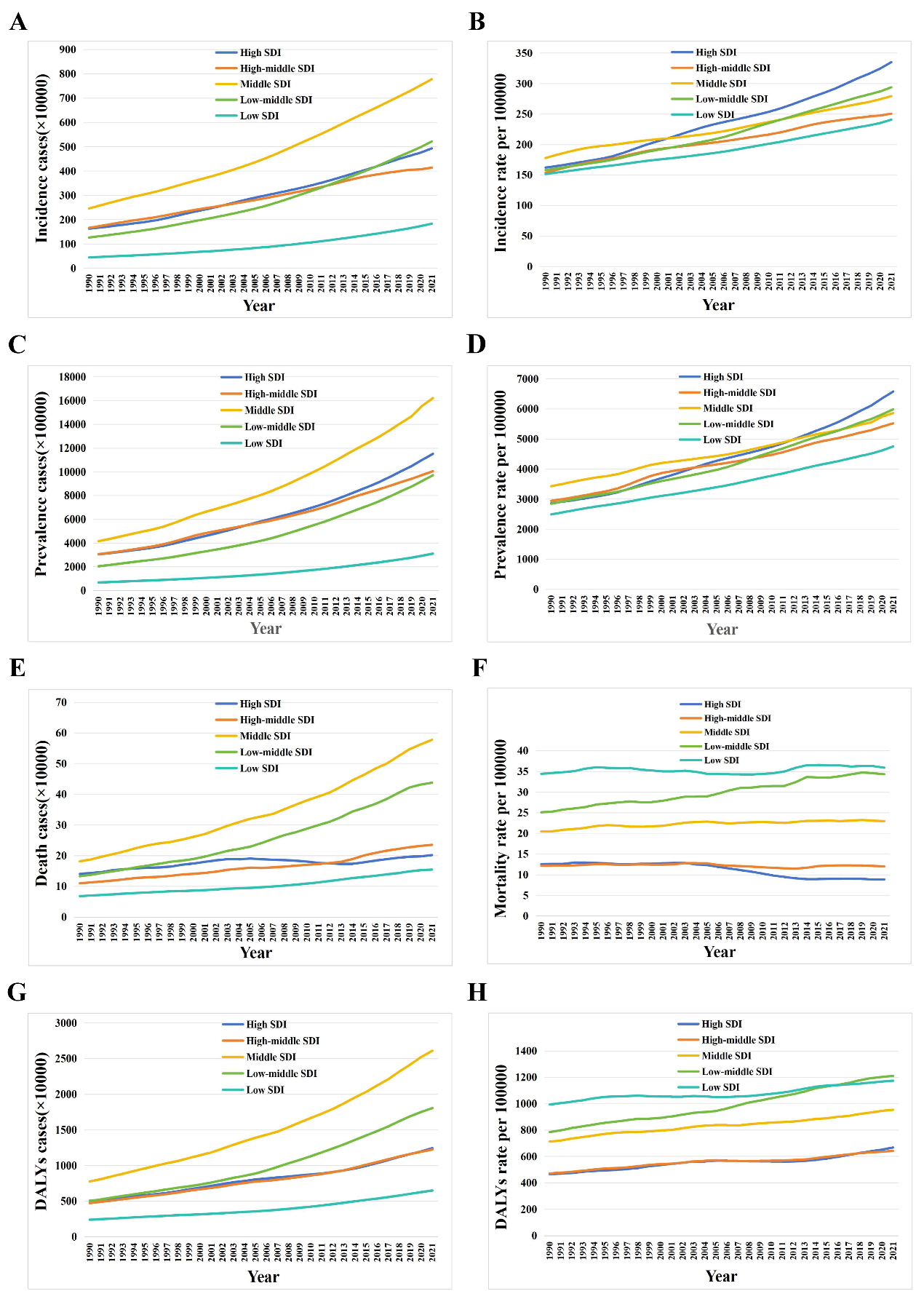


**Supplementary Figure 2.**  **Trends in absolute and relative burden of type 2 diabetes mellitus worldwide by SDI regions (1990-2021). (A and B)** Incidence counts and age-standardized incidence rates. **(C and D)** Prevalence counts and age-standardized prevalence rates. **(E and F)** Death counts and age-standardized mortality rates. **(G and H)** Disability-adjusted life years (DALYs) and age-standardized DALYs rates. SDI, sociodemographic index.


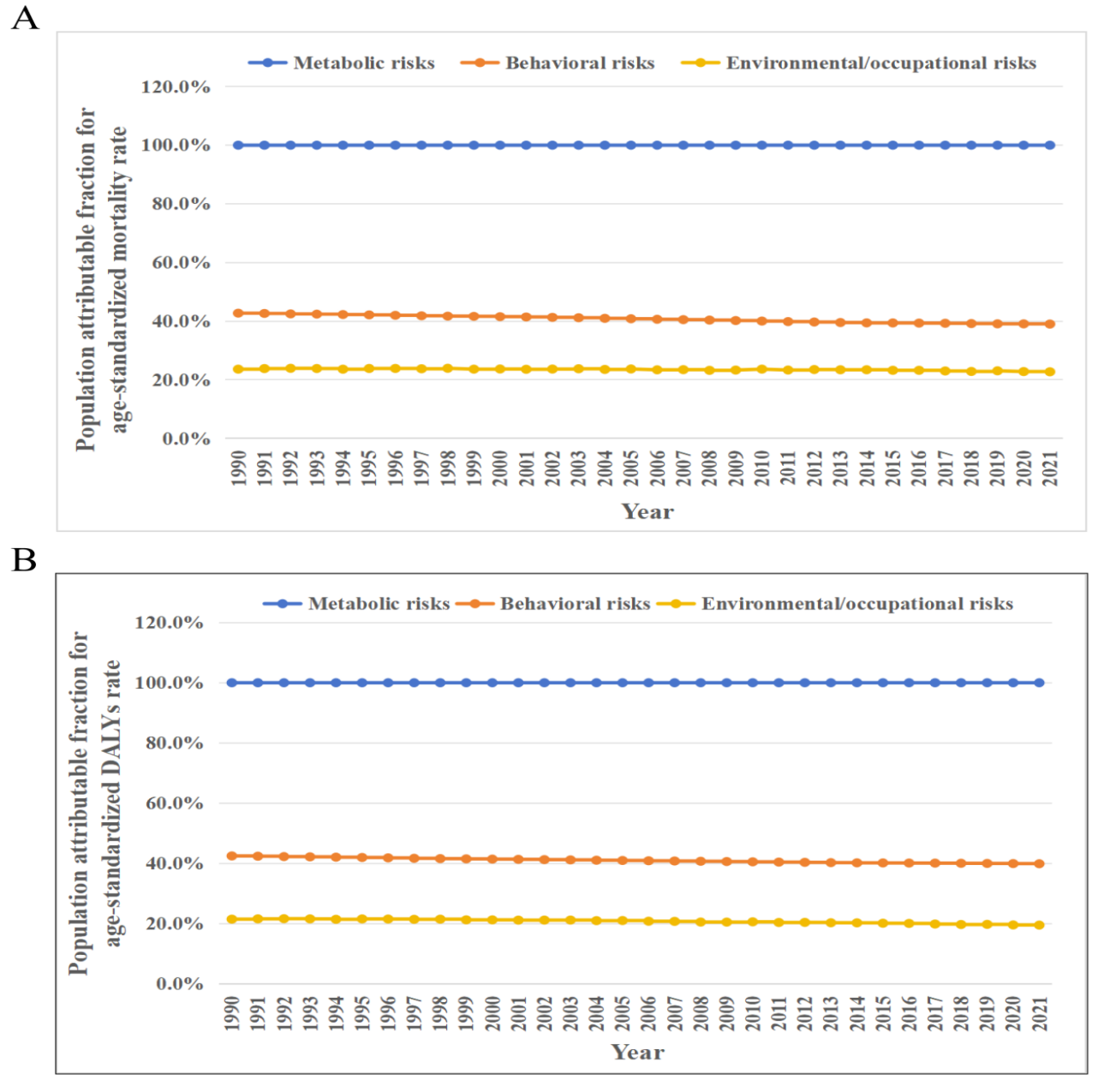


**Supplementary Figure 3.** **The trend analysis of the impact of the three major attributable risk factors on the relative burden of type 2 diabetes mellitus.** The Impact of metabolic risks, behavioral risks, and environmental/occupational risks on age-standardized mortality rates **(A)** and age-standardized DALYs **(B)** from 1990 to 2021. DALYs, disability-adjusted life years.

## Supplementary Tables

# Supplementary Table 1. Attributable risk factors for type 2 diabetes mellitus in GBD 2021.

| **Level 1** | **Level 2** | **Level 3** | **Level 4** |
| --- | --- | --- | --- |
| **Metabolic risks** | High fasting plasma glucose | High fasting plasma glucose | High fasting plasma glucose |
|  | High body-mass index | High body-mass index | High body-mass index |
| **Behavioral risks** | Dietary risks | Diet high in processed meat | Diet high in processed meat |
|  |  | Diet high in red meat | Diet high in red meat |
|  |  | Diet low in whole grains | Diet low in whole grains |
|  |  | Diet low in fruits | Diet low in fruits |
|  |  | Diet high in sugar-sweetened beverages | Diet high in sugar-sweetened beverages |
|  |  | Diet low in vegetables | Diet low in vegetables |
|  |  | Diet low in fiber | Diet low in fiber |
|  | Tobacco | Smoking | Smoking |
|  |  | Secondhand smoke | Secondhand smoke |
|  | Low physical activity | Low physical activity | Low physical activity |
|  | Alcohol use | Alcohol use | Alcohol use |
| **Environmental/occupational risks** | Air pollution | Particulate matter pollution | Household air pollution from solid fuels |
|  |  |  | Ambient particulate matter pollution |
|  | Non-optimal temperature | High temperature | High temperature |
|  |  |  | Low temperature |

GBD, Global Burden of Diseases.

# Supplementary Table 2. The incidence cases and age-specific incidence rate of type 2 diabetes mellitus in 1990 and 2021, and its temporal trends from 1990 to 2021, by sex and age groups.

| **Sex** | **Age groups** | **Number of incidence cases in 1990**  **(95%UI)** | **Number of incidence cases in 2021**  **(95%UI)** | **Percentage change in incidence counts, 1990-2021（95%UI）** | **1990 IR per 100000**  **(95%UI)** | **2021 IR per 100000**  **(95%UI)** | **Percentage change in IR, 1990-2021**  **(95%UI)** |
| --- | --- | --- | --- | --- | --- | --- | --- |
| **Both** | **20-24 years** | 488086 (344553 to 673150) | 1295409 (972911 to 1664116) | 165.4% (146.2 to 194.4) | 99.2  (70.0 to 136.8) | 216.9 (162.9 to 278.7) | 118.7% (102.8 to 142.6) |
|  | **25-29 years** | 447376 (295390 to 622401) | 1180775 (799979 to 1598495) | 163.9% (150.9 to 181.2) | 101.1 (66.7 to 140.6) | 200.7 (136.0 to 271.7) | 98.6%  (88.7 to 111.5) |
|  | **30-34 years** | 486564 (338585 to 641997) | 1465178 (1056843 to 1887287) | 201.1% (187.7 to 219.0) | 126.2 (87.8 to 166.6) | 242.4 (174.8 to 312.2) | 92.0%  (83.4 to 103.4) |
|  | **35-39 years** | 582725 (409231 to 809303) | 1793147 (1304812 to 2360336) | 207.7% (188.7 to 235.8) | 165.4 (116.2 to 229.8) | 319.7 (232.6 to 420.8) | 93.3%  (81.3 to 110.9) |
|  | **40-44 years** | 681193 (537986 to 843514) | 2185391 (1752811 to 2664680) | 220.8% (206.4 to 238.1) | 237.8 (187.8 to 294.4) | 436.9 (350.4 to 532.7) | 83.7%  (75.4 to 93.6) |
|  | **45-49 years** | 785524 (572713 to 1071798) | 2715301 (2041298 to 3601803) | 245.7% (228.8 to 265.7) | 338.3 (246.7 to 461.6) | 573.4 (431.1 to 760.7) | 69.5%  (61.2 to 79.3) |
|  | **50-54 years** | 862143 (680585 to 1081783) | 3054072 (2470039 to 3706140) | 254.2% (239.1 to 270.7) | 405.6 (320.2 to 508.9) | 686.4 (555.2 to 833.0) | 69.2%  (62.0 to 77.1) |
|  | **55-59 years** | 858432 (664875 to 1073446) | 3132299 (2473933 to 3824003) | 264.9% (246.9 to 284.5) | 463.5 (359.0 to 579.6) | 791.5 (625.2 to 966.3) | 70.8%  (62.3 to 79.9) |
|  | **60-64 years** | 754900 (581311 to 934926) | 2596907 (2059130 to 3145711) | 244.0% (228.2 to 262.3) | 470.0 (361.9 to 582.1) | 811.4 (643.4 to 982.9) | 72.6%  (64.7 to 81.8) |
|  | **65-69 years** | 528944 (406990 to 663673) | 1883548 (1514894 to 2300504) | 256.1% (238.2 to 278.3) | 427.9 (329.3 to 536.9) | 682.8 (549.2 to 834.0) | 59.6%  (51.5 to 69.5) |
|  | **70-74 years** | 320257 (241764 to 413616) | 1043812 (782143 to 1351352) | 225.9% (210.8 to 240.9) | 378.3 (285.6 to 488.6) | 507.1 (380.0 to 656.5) | 34.1%  (27.8 to 40.2) |
|  | **75-79 years** | 192153 (155933 to 239694) | 379697 (299616 to 479688) | 97.6% (80.9 to 113.1) | 312.2 (253.3 to 389.4) | 287.9 (227.2 to 363.7) | -7.8%  (-15.6 to -0.5) |
|  | **80-84 years** | 76953 (58026 to 102150) | 116148 (75223 to 170393) | 50.9% (27.8 to 71.3) | 217.5 (164.0 to 288.8) | 132.6  (85.9 to 194.6) | -39.0% (-48.4 to -30.8) |
|  | **85-89 years** | 13661 (10638 to 17710) | 23803 (16517 to 34254) | 74.2% (49.7 to 94.9) | 90.4  (70.4 to 117.2) | 52.1  (36.1 to 74.9) | -42.4% (-50.5 to -35.6) |
|  | **90-94 years** | 1040  (506 to 1746) | 2531  (1536 to 4067) | 143.4% (119.9 to 224.3) | 24.3  (11.8 to 40.7) | 14.1  (8.6 to 22.7) | -41.7% (-47.3 to -22.3) |
|  | **95+ years** | 165  (81 to 290) | 601  (392 to 923) | 263.4% (198.3 to 529.7) | 16.2  (8.0 to 28.5) | 11.0  (7.2 to 16.9) | -32.1% (-44.3 to 17.6) |
| **Male** | **20-24 years** | 263911 (184103 to 364229) | 709918 (535809 to 904480) | 169.0% (147.5 to 202.2) | 106.4 (74.2 to 146.9) | 234.0 (176.6 to 298.1) | 119.8% (102.2 to 147.0) |
|  | **25-29 years** | 248299 (164361 to 344364) | 640889 (432772 to 871079) | 158.1% (145.5 to 174.5) | 111.6 (73.9 to 154.8) | 215.5 (145.5 to 292.9) | 93.2%  (83.7 to 105.4) |
|  | **30-34 years** | 273757 (191942 to 360376) | 815111 (589912 to 1042982) | 197.7% (185.0 to 216.5) | 140.2 (98.3 to 184.5) | 266.8 (193.1 to 341.3) | 90.3%  (82.2 to 102.3) |
|  | **35-39 years** | 326338 (229886 to 449019) | 1009917 (741590 to 1316017) | 209.5% (188.6 to 236.6) | 182.5 (128.6 to 251.1) | 356.8 (262.0 to 464.9) | 95.5%  (82.3 to 112.6) |
|  | **40-44 years** | 368069 (290399 to 452361) | 1192752 (953601 to 1452633) | 224.1% (206.8 to 243.6) | 251.7 (198.6 to 309.3) | 473.0 (378.2 to 576.1) | 88.0%  (77.9 to 99.3) |
|  | **45-49 years** | 406060 (298298 to 551622) | 1419597 (1073563 to 1866705) | 249.6% (231.0 to 271.5) | 343.0 (252.0 to 465.9) | 596.8 (451.3 to 784.8) | 74.0%  (64.8 to 84.9) |
|  | **50-54 years** | 432416 (340093 to 546990) | 1536059 (1236774 to 1879124) | 255.2% (237.9 to 275.0) | 401.7 (315.9 to 508.1) | 692.0 (557.2 to 846.5) | 72.3%  (63.9 to 81.9) |
|  | **55-59 years** | 418982 (321640 to 530037) | 1511720 (1172461 to 1852315) | 260.8% (243.3 to 282.0) | 451.1 (346.3 to 570.6) | 776.3 (602.1 to 951.2) | 72.1%  (63.8 to 82.2) |
|  | **60-64 years** | 368245 (283482 to 455200) | 1273895 (1017931 to 1545146) | 245.9% (229.2 to 266.8) | 468.8 (360.9 to 579.5) | 819.0 (654.5 to 993.4) | 74.7%  (66.3 to 85.2) |
|  | **65-69 years** | 260996 (201475 to 329318) | 968338 (784505 to 1178673) | 271.0% (249.4 to 294.4) | 455.2 (351.4 to 574.4) | 734.5 (595.1 to 894.1) | 61.3%  (51.9 to 71.5) |
|  | **70-74 years** | 156062 (118069 to 200970) | 537029 (405906 to 688887) | 244.1% (226.6 to 262.1) | 414.9 (313.9 to 534.2) | 557.1 (421.1 to 714.7) | 34.3%  (27.4 to 41.3) |
|  | **75-79 years** | 84554 (69202 to 104889) | 177214 (140414 to 222988) | 109.6% (92.0 to 127.1) | 335.1 (274.3 to 415.7) | 296.4 (234.9 to 373.0) | -11.5% (-19.0 to -4.1) |
|  | **80-84 years** | 30336 (22606 to 40835) | 46159 (29977 to 68952) | 52.2% (26.4 to 75.3) | 228.4 (170.2 to 307.4) | 125.9  (81.8 to 188.1) | -44.9% (-54.2 to -36.5) |
|  | **85-89 years** | 4880 (3803 to 6311) | 8915  (6175 to 12830) | 82.7% (56.7 to 104.9) | 96.4  (75.1 to 124.6) | 51.7  (35.8 to 74.4) | -46.4% (-54.0 to -39.9) |
|  | **90-94 years** | 321  (165 to 528) | 919  (548 to 1461) | 186.4% (164.0 to 253.2) | 25.5  (13.1 to 41.9) | 15.8  (9.4 to 25.1) | -38.1% (-43.0 to -23.7) |
|  | **95+ years** | 45  (24 to 76) | 185  (116 to 294) | 311.6% (260.5 to 466.6) | 17.3  (9.2 to 29.2) | 12.3  (7.7 to 19.4) | -29.2% (-38.0 to -2.5) |
| **Female** | **20-24 years** | 224175 (157821 to 309075) | 585491 (435713 to 761869) | 161.2% (143.4 to 186.0) | 91.8  (64.6 to 126.6) | 199.3 (148.3 to 259.4) | 117.1% (102.2 to 137.7) |
|  | **25-29 years** | 199077 (131396 to 278620) | 539886 (365482 to 732247) | 171.2% (155.9 to 191.3) | 90.4  (59.7 to 126.6) | 185.5 (125.6 to 251.6) | 105.1% (93.6 to 120.3) |
|  | **30-34 years** | 212806 (146591 to 283171) | 650067 (466931 to 845773) | 205.5% (191.0 to 224.7) | 111.9 (77.1 to 149.0) | 217.5 (156.2 to 282.9) | 94.3%  (85.1 to 106.5) |
|  | **35-39 years** | 256387 (178711 to 360826) | 783230 (567536 to 1037492) | 205.5% (186.8 to 235.3) | 147.8 (103.0 to 208.0) | 281.9 (204.3 to 373.5) | 90.7%  (79.1 to 109.3) |
|  | **40-44 years** | 313124 (247652 to 390342) | 992639 (803063 to 1208894) | 217.0% (201.5 to 232.9) | 223.3 (176.6 to 278.4) | 400.1 (323.7 to 487.3) | 79.2%  (70.4 to 88.2) |
|  | **45-49 years** | 379465 (274180 to 521959) | 1295703 (967052 to 1732789) | 241.5% (223.4 to 261.0) | 333.4 (240.9 to 458.7) | 549.9 (410.4 to 735.3) | 64.9%  (56.2 to 74.3) |
|  | **50-54 years** | 429727 (342455 to 533118) | 1518013 (1236081 to 1836246) | 253.3% (239.0 to 269.4) | 409.6 (326.4 to 508.1) | 680.9 (554.4 to 823.6) | 66.2%  (59.5 to 73.8) |
|  | **55-59 years** | 439450 (341327 to 546388) | 1620579 (1284975 to 1964403) | 268.8% (250.9 to 287.3) | 476.0 (369.8 to 591.9) | 806.2 (639.3 to 977.3) | 69.4%  (61.1 to 77.9) |
|  | **60-64 years** | 386655 (296960 to 476729) | 1323013 (1041185 to 1598138) | 242.2% (225.9 to 261.3) | 471.2 (361.9 to 580.9) | 804.2 (632.9 to 971.4) | 70.7%  (62.6 to 80.2) |
|  | **65-69 years** | 267947 (205810 to 335354) | 915210 (734120 to 1113297) | 241.6% (226.3 to 262.4) | 404.3 (310.5 to 506.0) | 635.5 (509.8 to 773.1) | 57.2%  (50.2 to 66.8) |
|  | **70-74 years** | 164195 (124249 to 212628) | 506783 (375979 to 664850) | 208.6% (193.4 to 222.9) | 349.0 (264.1 to 452.0) | 463.0 (343.5 to 607.5) | 32.7%  (26.1 to 38.8) |
|  | **75-79 years** | 107599 (86697 to 133775) | 202483 (158867 to 257259) | 88.2% (71.3 to 103.5) | 296.2 (238.7 to 368.3) | 280.8 (220.3 to 356.8) | -5.2%  (-13.7 to 2.5) |
|  | **80-84 years** | 46617 (35260 to 61400) | 69989 (45842 to 102034) | 50.1% (27.5 to 70.2) | 211.0 (159.6 to 277.9) | 137.4  (90.0 to 200.3) | -34.9% (-44.7 to -26.2) |
|  | **85-89 years** | 8781 (6774 to 11413) | 14889 (10288 to 21454) | 69.5% (46.1 to 90.0) | 87.4  (67.4 to 113.6) | 52.3  (36.1 to 75.4) | -40.2% (-48.4 to -32.9) |
|  | **90-94 years** | 719  (346 to 1219) | 1612  (977 to 2613) | 124.2% (99.5 to 211.3) | 23.8  (11.4 to 40.3) | 13.4  (8.1 to 21.7) | -43.8% (-49.9 to -21.9) |
|  | **95+ years** | 120  (58 to 214) | 416  (271 to 634) | 245.4% (173.9 to 557.2) | 15.9  (7.6 to 28.3) | 10.6  (6.9 to 16.1) | -33.5% (-47.3 to 26.5) |

IR, age-specific incidence rate; UI, uncertainty interval.

# Supplementary Table 3. The prevalence cases and age-specific prevalence rate of type 2 diabetes mellitus in 1990 and 2021, and its temporal trends from 1990 to 2021, by sex and age groups.

| **Sex** | **Age groups** | **Number of prevalence cases in 1990**  **(95%UI)** | **Number of prevalence cases in 2021**  **(95%UI)** | **Percentage change in prevalence counts, 1990-2021(95%UI)** | **1990 PR per 100000**  **(95%UI)** | **2021 PR per 100000**  **(95%UI)** | **Percentage change in PR, 1990-2021**  **(95%UI)** |
| --- | --- | --- | --- | --- | --- | --- | --- |
| **Both** | **20-24 years** | 3803991 (2852876 to 4950387) | 10215750 (8242829 to 12542924) | 168.6% (149.7 to 195.2) | 773.0 (579.7 to 1006.0) | 1710.7 (1380.3 to 2100.4) | 121.3% (105.7 to 143.3) |
|  | **25-29 years** | 5432323 (4254082 to 6769295) | 16374467 (13650329 to 19374934) | 201.4% (184.2 to 223.5) | 1227.3 (961.1 to 1529.4) | 2783.1 (2320.1 to 3293.1) | 126.8% (113.8 to 143.4) |
|  | **30-34 years** | 6640640 (5395767 to 7994376) | 23978508 (20353652 to 27962557) | 261.1% (244.0 to 282.9) | 1723.0 (1400.0 to 2074.2) | 3966.8 (3367.1 to 4625.9) | 130.2% (119.3 to 144.2) |
|  | **35-39 years** | 8563234 (7051127 to 10062518) | 29377055 (25252585 to 33622680) | 243.1% (228.5 to 260.7) | 2431.0 (2001.8 to 2856.7) | 5237.8 (4502.4 to 5994.8) | 115.5% (106.3 to 126.5) |
|  | **40-44 years** | 9425482 (7927069 to 10964343) | 34530671 (29955397 to 39083869) | 266.4% (251.5 to 284.3) | 3290.1 (2767.0 to 3827.2) | 6902.7 (5988.1 to 7812.9) | 109.8% (101.3 to 120.1) |
|  | **45-49 years** | 10573647 (9211997 to 12122703) | 43529486 (38884726 to 49011245) | 311.7% (296.9 to 328.9) | 4553.8 (3967.3 to 5220.9) | 9193.0 (8212.1 to 10350.7) | 101.9% (94.6 to 110.3) |
|  | **50-54 years** | 12897409 (11186944 to 14998075) | 52917971 (47355503 to 59679201) | 310.3% (293.8 to 327.7) | 6067.3 (5262.7 to 7055.5) | 11893.7 (10643.5 to 13413.4) | 96.0% (88.2 to 104.3) |
|  | **55-59 years** | 14656245 (12904520 to 16853457) | 59224759 (53399647 to 65781350) | 304.1% (289.3 to 319.3) | 7913.7 (6967.9 to 9100.1) | 14966.0 (13494.0 to 16622.9) | 89.1% (82.2 to 96.2) |
|  | **60-64 years** | 15627051 (13764367 to 17504455) | 59035405 (53410095 to 64638871) | 277.8% (265.7 to 290.3) | 9729.9 (8570.1 to 10898.8) | 18445.8 (16688.2 to 20196.6) | 89.6% (83.5 to 95.8) |
|  | **65-69 years** | 14124587 (12600953 to 15739460) | 58061945 (52943606 to 63488762) | 311.1% (299.6 to 324.0) | 11426.8 (10194.2 to 12733.2) | 21049.0 (19193.4 to 23016.3) | 84.2% (79.0 to 90.0) |
|  | **70-74 years** | 10792586 (9612884 to 12006598) | 47653407 (43525411 to 51948594) | 341.5% (329.5 to 356.0) | 12748.0 (11354.5 to 14181.9) | 23150.8 (21145.3 to 25237.5) | 81.6% (76.7 to 87.6) |
|  | **75-79 years** | 8151266 (7332420 to 9059955) | 31693480 (29076101 to 34490007) | 288.8% (280.0 to 298.9) | 13242.1 (11911.9 to 14718.3) | 24031.3 (22046.7 to 26151.7) | 81.5% (77.3 to 86.2) |
|  | **80-84 years** | 4719714 (4276343 to 5183223) | 20340480 (18745722 to 22163618) | 331.0% (319.7 to 341.7) | 13341.6 (12088.3 to 14651.8) | 23224.2 (21403.4 to 25305.8) | 74.1% (69.5 to 78.4) |
|  | **85-89 years** | 1895226 (1720073 to 2077952) | 10091818 (9284709 to 11037642) | 432.5% (416.8 to 447.4) | 12542.0 (11382.9 to 13751.2) | 22072.2 (20307.0 to 24140.9) | 76.0% (70.8 to 80.9) |
|  | **90-94 years** | 464537 (418443 to 515160) | 3634933 (3327325 to 3994832) | 682.5% (654.4 to 710.7) | 10840.5 (9764.9 to 12021.9) | 20318.9 (18599.5 to 22330.8) | 87.4% (80.7 to 94.2) |
|  | **95+ years** | 87714 (76942 to 99777) | 1000399 (906124 to 1112512) | 1040.5% (988.9 to 1100.3) | 8615.5 (7557.5 to 9800.5) | 18354.9 (16625.2 to 20411.9) | 113.0% (103.4 to 124.2) |
| **Male** | **20-24 years** | 1976360 (1477920 to 2559595) | 5477653 (4402286 to 6696087) | 177.2% (155.7 to 208.8) | 797.1 (596.0 to 1032.3) | 1805.4 (1451.0 to 2207.0) | 126.5% (109.0 to 152.4) |
|  | **25-29 years** | 2881128 (2252386 to 3602809) | 8810222 (7381908 to 10397870) | 205.8% (186.4 to 232.2) | 1294.8 (1012.2 to 1619.1) | 2962.9 (2482.5 to 3496.8) | 128.8% (114.3 to 148.6) |
|  | **30-34 years** | 3614892 (2940861 to 4342362) | 13064251 (11108328 to 15095040) | 261.4% (242.4 to 285.2) | 1850.8 (1505.7 to 2223.3) | 4275.7 (3635.5 to 4940.3) | 131.0% (118.9 to 146.2) |
|  | **35-39 years** | 4726220 (3881068 to 5567625) | 16101713 (13840554 to 18415459) | 240.7% (225.6 to 259.5) | 2643.4 (2170.7 to 3114.0) | 5688.4 (4889.6 to 6505.8) | 115.2% (105.7 to 127.1) |
|  | **40-44 years** | 5240512 (4390395 to 6074093) | 19003358 (16527454 to 21525261) | 262.6% (247.8 to 282.4) | 3583.1 (3001.9 to 4153.1) | 7536.2 (6554.3 to 8536.3) | 110.3% (101.7 to 121.8) |
|  | **45-49 years** | 5784515 (5040052 to 6621035) | 23789830 (21268548 to 26686376) | 311.3% (296.1 to 327.9) | 4885.8 (4257.0 to 5592.3) | 10001.5 (8941.5 to 11219.3) | 104.7% (97.1 to 113.0) |
|  | **50-54 years** | 6886702 (6043971 to 8021392) | 28410190 (25586475 to 31803528) | 312.5% (295.8 to 330.7) | 6397.0 (5614.2 to 7451.0) | 12798.5 (11526.5 to 14327.2) | 100.1% (92.0 to 108.9) |
|  | **55-59 years** | 7623381 (6716839 to 8724384) | 30881486 (27912943 to 34233071) | 305.1% (289.6 to 321.2) | 8207.1 (7231.1 to 9392.4) | 15859.1 (14334.6 to 17580.3) | 93.2% (85.8 to 100.9) |
|  | **60-64 years** | 7874176 (6945587 to 8820189) | 30072752 (27179204 to 32909091) | 281.9% (268.9 to 295.0) | 10024.9 (8842.7 to 11229.3) | 19334.8 (17474.4 to 21158.4) | 92.9% (86.3 to 99.5) |
|  | **65-69 years** | 6814588 (6088290 to 7564177) | 29112572 (26585515 to 31722333) | 327.2% (314.5 to 340.7) | 11886.3 (10619.5 to 13193.8) | 22082.9 (20166.0 to 24062.5) | 85.8% (80.2 to 91.6) |
|  | **70-74 years** | 5044234 (4495950 to 5608430) | 23716005 (21728042 to 25758908) | 370.2% (357.0 to 385.8) | 13408.9 (11951.4 to 14908.7) | 24603.9 (22541.5 to 26723.2) | 83.5% (78.3 to 89.6) |
|  | **75-79 years** | 3582243 (3218928 to 3978829) | 15323007 (14091278 to 16640721) | 327.7% (317.4 to 339.2) | 14196.8 (12756.9 to 15768.5) | 25629.4 (23569.2 to 27833.5) | 80.5% (76.1 to 85.4) |
|  | **80-84 years** | 1919436 (1739759 to 2110083) | 9162993 (8456164 to 9970897) | 377.4% (365.0 to 389.7) | 14449.8 (13097.2 to 15885.1) | 25000.1 (23071.6 to 27204.3) | 73.0% (68.5 to 77.5) |
|  | **85-89 years** | 698638 (635554 to 767979) | 4161283 (3830747 to 4540123) | 495.6% (477.4 to 513.9) | 13796.0 (12550.3 to 15165.3) | 24119.7 (22203.8 to 26315.5) | 74.8% (69.5 to 80.2) |
|  | **90-94 years** | 153240 (138489 to 170098) | 1328253 (1217409 to 1458075) | 766.8% (735.0 to 799.8) | 12171.9 (11000.3 to 13511.0) | 22788.9 (20887.2 to 25016.3) | 87.2% (80.4 to 94.4) |
|  | **95+ years** | 26275 (23208 to 29798) | 322294 (292171 to 356441) | 1126.6% (1070.5 to 1193.5) | 10096.7 (8918.0 to 11450.5) | 21315.2 (19322.9 to 23573.5) | 111.1% (101.4 to 122.6) |
| **Female** | **20-24 years** | 1827630 (1373632 to 2394879) | 4738097 (3810163 to 5864748) | 159.2% (142.3 to 182.5) | 748.6 (562.7 to 981.0) | 1613.0 (1297.1 to 1996.5) | 115.5% (101.4 to 134.8) |
|  | **25-29 years** | 2551195 (2009906 to 3176484) | 7564245 (6310641 to 8986320) | 196.5% (179.7 to 216.5) | 1159.1 (913.2 to 1443.2) | 2599.5 (2168.7 to 3088.2) | 124.3% (111.6 to 139.4) |
|  | **30-34 years** | 3025748 (2464518 to 3652219) | 10914257 (9290751 to 12698935) | 260.7% (243.6 to 281.0) | 1591.6 (1296.4 to 1921.1) | 3651.1 (3108.0 to 4248.1) | 129.4% (118.5 to 142.3) |
|  | **35-39 years** | 3837015 (3155867 to 4526687) | 13275343 (11437918 to 15199468) | 246.0% (232.0 to 264.2) | 2212.1 (1819.4 to 2609.7) | 4778.7 (4117.3 to 5471.3) | 116.0% (107.3 to 127.4) |
|  | **40-44 years** | 4184970 (3531715 to 4882879) | 15527313 (13427149 to 17629009) | 271.0% (256.6 to 289.2) | 2984.5 (2518.6 to 3482.2) | 6258.7 (5412.2 to 7105.9) | 109.7% (101.6 to 120.0) |
|  | **45-49 years** | 4789132 (4179121 to 5507300) | 19739655 (17616178 to 22243549) | 312.2% (297.1 to 328.5) | 4208.4 (3672.3 to 4839.4) | 8376.9 (7475.8 to 9439.5) | 99.1% (91.8 to 106.9) |
|  | **50-54 years** | 6010707 (5191400 to 7043863) | 24507781 (21805378 to 27743079) | 307.7% (290.5 to 325.2) | 5729.0 (4948.1 to 6713.8) | 10992.9 (9780.7 to 12444.0) | 91.9% (83.8 to 100.1) |
|  | **55-59 years** | 7032864 (6185180 to 8125209) | 28343274 (25420563 to 31678847) | 303.0% (288.4 to 318.1) | 7618.6 (6700.3 to 8801.9) | 14100.9 (12646.8 to 15760.3) | 85.1% (78.4 to 92.0) |
|  | **60-64 years** | 7752875 (6818311 to 8730948) | 28962653 (26140737 to 31788519) | 273.6% (261.3 to 286.2) | 9447.5 (8308.6 to 10639.3) | 17605.3 (15890.0 to 19323.0) | 86.3% (80.2 to 92.7) |
|  | **65-69 years** | 7310000 (6503955 to 8146592) | 28949373 (26297423 to 31775337) | 296.0% (284.6 to 308.6) | 11029.3 (9813.1 to 12291.5) | 20102.5 (18261.0 to 22064.8) | 82.3% (77.0 to 88.1) |
|  | **70-74 years** | 5748352 (5115476 to 6406517) | 23937401 (21855814 to 26153604) | 316.4% (305.5 to 330.1) | 12219.4 (10874.1 to 13618.5) | 21871.1 (19969.2 to 23896.0) | 79.0% (74.3 to 84.8) |
|  | **75-79 years** | 4569022 (4109164 to 5073048) | 16370473 (14989276 to 17821428) | 258.3% (249.5 to 268.3) | 12579.0 (11312.9 to 13966.6) | 22706.0 (20790.2 to 24718.4) | 80.5% (76.1 to 85.6) |
|  | **80-84 years** | 2800278 (2538242 to 3071560) | 11177486 (10283659 to 12191395) | 299.2% (288.1 to 309.6) | 12675.3 (11489.2 to 13903.2) | 21946.2 (20191.3 to 23937.0) | 73.1% (68.3 to 77.7) |
|  | **85-89 years** | 1196588 (1082913 to 1309996) | 5930535 (5459183 to 6491258) | 395.6% (380.5 to 410.0) | 11909.9 (10778.5 to 13038.7) | 20831.5 (19175.8 to 22801.0) | 74.9% (69.6 to 80.0) |
|  | **90-94 years** | 311297 (279999 to 345512) | 2306680 (2107880 to 2543148) | 641.0% (615.0 to 668.4) | 10286.6 (9252.4 to 11417.2) | 19125.3 (17477.0 to 21085.9) | 85.9% (79.4 to 92.8) |
|  | **95+ years** | 61439 (53797 to 69923) | 678105 (613426 to 755774) | 1003.7% (951.1 to 1063.5) | 8107.0 (7098.5 to 9226.4) | 17218.3 (15576.0 to 19190.5) | 112.4% (102.3 to 123.9) |

PR, age-specific prevalence rate; UI, uncertainty interval.

# Supplementary Table 4. The death cases and age-specific mortality rate of type 2 diabetes mellitus in 1990 and 2021, and its temporal trends from 1990 to 2021, by sex and age groups.

| **Sex** | **Age groups** | **Number of death cases in 1990**  **(95%UI)** | **Number of death cases in 2021**  **(95%UI)** | **Percentage change in death counts, 1990-2021(95%UI)** | **1990 MR per 100000**  **(95%UI)** | **2021 MR per 100000**  **(95%UI)** | **Percentage change in MR, 1990-2021**  **(95%UI)** |
| --- | --- | --- | --- | --- | --- | --- | --- |
| **Both** | **20-24 years** | 1679 (1456 to 1876) | 2394 (2058 to 2748) | 42.6% (24.2 to 63.9) | 0.3  (0.3 to 0.4) | 0.4  (0.3 to 0.5) | 17.5%  (2.4 to 35.0) |
|  | **25-29 years** | 2151 (1893 to 2388) | 3267 (2866 to 3798) | 51.9% (32.8 to 70.6) | 0.5  (0.4 to 0.5) | 0.6  (0.5 to 0.6) | 14.2% (-0.1 to 28.4) |
|  | **30-34 years** | 4143 (3779 to 4463) | 7129 (6408 to 7931) | 72.1% (53.3 to 89.1) | 1.1  (1.0 to 1.2) | 1.2  (1.1 to 1.3) | 9.7%  (-2.3 to 20.6) |
|  | **35-39 years** | 6145 (5646 to 6593) | 11004 (9995 to 12152) | 79.1% (62.1 to 96.4) | 1.7  (1.6 to 1.9) | 2.0  (1.8 to 2.2) | 12.5%  (1.8 to 23.3) |
|  | **40-44 years** | 10270 (9486 to 10888) | 22192 (20033 to 24336) | 116.1% (95.2 to 138.1) | 3.6  (3.3 to 3.8) | 4.4  (4.0 to 4.9) | 23.7% (11.8 to 36.4) |
|  | **45-49 years** | 21669 (20615 to 22998) | 47686 (44062 to 51557) | 120.1% (97.9 to 138.7) | 9.3  (8.9 to 9.9) | 10.1  (9.3 to 10.9) | 7.9%  (-2.9 to 17.0) |
|  | **50-54 years** | 37268 (35348 to 39362) | 82890 (76904 to 88725) | 122.4% (101.5 to 139.8) | 17.5  (16.6 to 18.5) | 18.6  (17.3 to 19.9) | 6.3%  (-3.7 to 14.6) |
|  | **55-59 years** | 54834 (51956 to 58165) | 125279 (116720 to 134270) | 128.5% (107.1 to 147.1) | 29.6  (28.1 to 31.4) | 31.7  (29.5 to 33.9) | 6.9%  (-3.1 to 15.7) |
|  | **60-64 years** | 79632 (75753 to 83824) | 176241 (165146 to 187136) | 121.3% (102.5 to 136.9) | 49.6  (47.2 to 52.2) | 55.1  (51.6 to 58.5) | 11.1%  (1.6 to 18.9) |
|  | **65-69 years** | 92364 (87205 to 96845) | 220127 (206171 to 234391) | 138.3% (118.2 to 155.5) | 74.7  (70.5 to 78.3) | 79.8  (74.7 to 85.0) | 6.8%  (-2.2 to 14.5) |
|  | **70-74 years** | 91929 (86841 to 96974) | 234174 (218953 to 248827) | 154.7% (134.1 to 173.5) | 108.6 (102.6 to 114.5) | 113.8  (106.4 to 120.9) | 4.8%  (-3.7 to 12.5) |
|  | **75-79 years** | 94391 (88181 to 98786) | 223852 (206870 to 237388) | 137.2% (119.6 to 153.6) | 153.3 (143.3 to 160.5) | 169.7  (156.9 to 180.0) | 10.7%  (2.5 to 18.4) |
|  | **80-84 years** | 71525 (64710 to 75861) | 202286 (181530 to 216233) | 182.8% (163.0 to 200.2) | 202.2 (182.9 to 214.4) | 231.0  (207.3 to 246.9) | 14.2%  (6.2 to 21.2) |
|  | **85-89 years** | 41866 (36657 to 44765) | 145795 (124616 to 157898) | 248.2% (227.6 to 269.5) | 277.1 (242.6 to 296.2) | 318.9  (272.6 to 345.3) | 15.1%  (8.3 to 22.1) |
|  | **90-94 years** | 16173 (13689 to 17661) | 74981 (60785 to 82366) | 363.6% (340.6 to 386.3) | 377.4 (319.4 to 412.1) | 419.1  (339.8 to 460.4) | 11.1%  (5.5 to 16.5) |
|  | **95+ years** | 4843 (3745 to 5381) | 26694 (19947 to 30271) | 451.2% (423.8 to 477.4) | 475.7 (367.9 to 528.6) | 489.8  (366.0 to 555.4) | 3.0%  (-2.2 to 7.9) |
| **Male** | **20-24 years** | 654 (540 to 768) | 1048 (840 to 1292) | 60.4% (33.6 to 89.6) | 0.3  (0.2 to 0.3) | 0.3  (0.3 to 0.4) | 31.1%  (9.2 to 55.0) |
|  | **25-29 years** | 861 (735 to 985) | 1517 (1266 to 1832) | 76.2% (50.4 to 104.2) | 0.4  (0.3 to 0.4) | 0.5  (0.4 to 0.6) | 31.8% (12.5 to 52.8) |
|  | **30-34 years** | 2271 (2042 to 2487) | 4263 (3736 to 4887) | 87.7% (61.9 to 114.2) | 1.2  (1.0 to 1.3) | 1.4  (1.2 to 1.6) | 20.0%  (3.5 to 36.9) |
|  | **35-39 years** | 3288 (2942 to 3616) | 6151 (5446 to 6990) | 87.1% (62.5 to 111.6) | 1.8  (1.6 to 2.0) | 2.2  (1.9 to 2.5) | 18.2%  (2.6 to 33.7) |
|  | **40-44 years** | 5645 (5143 to 6161) | 12708 (11263 to 14264) | 125.1% (93.4 to 154.2) | 3.9  (3.5 to 4.2) | 5.0  (4.5 to 5.7) | 30.6% (12.2 to 47.4) |
|  | **45-49 years** | 12041 (11226 to 13049) | 27061 (24573 to 29709) | 124.7% (92.1 to 153.9) | 10.2  (9.5 to 11.0) | 11.4  (10.3 to 12.5) | 11.9% (-4.4 to 26.4) |
|  | **50-54 years** | 19910 (18552 to 21507) | 45933 (41699 to 49917) | 130.7% (96.9 to 157.6) | 18.5  (17.2 to 20.0) | 20.7  (18.8 to 22.5) | 11.9% (-4.5 to 24.9) |
|  | **55-59 years** | 28348 (26544 to 30662) | 67208 (61374 to 72796) | 137.1% (104.8 to 166.0) | 30.5  (28.6 to 33.0) | 34.5  (31.5 to 37.4) | 13.1% (-2.3 to 26.9) |
|  | **60-64 years** | 38704 (36010 to 41696) | 89899 (82667 to 97066) | 132.3% (102.1 to 159.1) | 49.3  (45.8 to 53.1) | 57.8  (53.1 to 62.4) | 17.3%  (2.0 to 30.9) |
|  | **65-69 years** | 43244 (40156 to 46399) | 108733 (99572 to 117400) | 151.4% (119.9 to 180.5) | 75.4  (70.0 to 80.9) | 82.5  (75.5 to 89.1) | 9.3%  (-4.4 to 22.0) |
|  | **70-74 years** | 41701 (38696 to 44765) | 115177 (105516 to 125299) | 176.2% (142.5 to 209.1) | 110.9 (102.9 to 119.0) | 119.5  (109.5 to 130.0) | 7.8%  (-5.3 to 20.6) |
|  | **75-79 years** | 39969 (37218 to 42514) | 105429 (96594 to 113804) | 163.8% (134.1 to 194.9) | 158.4 (147.5 to 168.5) | 176.3  (161.6 to 190.3) | 11.3% (-1.2 to 24.5) |
|  | **80-84 years** | 27936 (25725 to 29958) | 86361 (77585 to 93103) | 209.1% (174.9 to 240.5) | 210.3 (193.7 to 225.5) | 235.6  (211.7 to 254.0) | 12.0% (-0.4 to 23.4) |
|  | **85-89 years** | 14964 (13381 to 16244) | 62189 (55196 to 67587) | 315.6% (272.1 to 358.2) | 295.5 (264.2 to 320.8) | 360.5  (319.9 to 391.7) | 22.0%  (9.2 to 34.5) |
|  | **90-94 years** | 5117 (4467 to 5578) | 28207 (24350 to 30781) | 451.3% (401.1 to 505.5) | 406.4 (354.8 to 443.1) | 483.9  (417.8 to 528.1) | 19.1%  (8.2 to 30.8) |
|  | **95+ years** | 1249 (1031 to 1385) | 7765 (6135 to 8657) | 521.6% (470.8 to 578.6) | 480.0 (396.1 to 532.3) | 513.5  (405.8 to 572.5) | 7.0%  (-1.8 to 16.8) |
| **Female** | **20-24 years** | 1025 (849 to 1166) | 1346 (1126 to 1530) | 31.3% (12.9 to 56.1) | 0.4  (0.3 to 0.5) | 0.5  (0.4 to 0.5) | 9.1%  (-6.1 to 29.8) |
|  | **25-29 years** | 1290 (1090 to 1477) | 1750 (1482 to 2017) | 35.6% (16.9 to 57.4) | 0.6  (0.5 to 0.7) | 0.6  (0.5 to 0.7) | 2.6% (-11.6 to 19.0) |
|  | **30-34 years** | 1873 (1661 to 2081) | 2866 (2524 to 3200) | 53.1% (35.0 to 72.4) | 1.0  (0.9 to 1.1) | 1.0  (0.8 to 1.1) | -2.7% (-14.2 to 9.6) |
|  | **35-39 years** | 2858 (2524 to 3160) | 4853 (4338 to 5360) | 69.8% (51.2 to 90.5) | 1.6  (1.5 to 1.8) | 1.7  (1.6 to 1.9) | 6.0%  (-5.6 to 18.9) |
|  | **40-44 years** | 4626 (4170 to 5034) | 9484 (8470 to 10451) | 105.0% (83.9 to 130.0) | 3.3  (3.0 to 3.6) | 3.8  (3.4 to 4.2) | 15.9%  (3.9 to 30.0) |
|  | **45-49 years** | 9628 (9032 to 10386) | 20625 (18916 to 22495) | 114.2% (91.9 to 134.6) | 8.5  (7.9 to 9.1) | 8.8  (8.0 to 9.5) | 3.5%  (-7.3 to 13.3) |
|  | **50-54 years** | 17359 (16240 to 18643) | 36957 (33822 to 40095) | 112.9% (92.5 to 132.9) | 16.5  (15.5 to 17.8) | 16.6  (15.2 to 18.0) | 0.2%  (-9.4 to 9.6) |
|  | **55-59 years** | 26486 (24825 to 28529) | 58071 (53381 to 63110) | 119.3% (99.0 to 141.7) | 28.7  (26.9 to 30.9) | 28.9  (26.6 to 31.4) | 0.7%  (-8.6 to 11.0) |
|  | **60-64 years** | 40927 (38385 to 43567) | 86341 (80211 to 92787) | 111.0% (90.6 to 130.2) | 49.9  (46.8 to 53.1) | 52.5  (48.8 to 56.4) | 5.2%  (-4.9 to 14.8) |
|  | **65-69 years** | 49119 (45760 to 52275) | 111395 (103701 to 119226) | 126.8% (108.0 to 146.4) | 74.1  (69.0 to 78.9) | 77.4  (72.0 to 82.8) | 4.4%  (-4.3 to 13.4) |
|  | **70-74 years** | 50228 (46820 to 53591) | 118998 (110273 to 127680) | 136.9% (114.1 to 159.0) | 106.8 (99.5 to 113.9) | 108.7  (100.8 to 116.7) | 1.8%  (-8.0 to 11.3) |
|  | **75-79 years** | 54421 (50254 to 57830) | 118423 (108317 to 126765) | 117.6% (98.9 to 132.8) | 149.8 (138.4 to 159.2) | 164.3  (150.2 to 175.8) | 9.6%  (0.2 to 17.3) |
|  | **80-84 years** | 43588 (38673 to 46473) | 115925 (102233 to 125199) | 166.0% (146.3 to 184.2) | 197.3 (175.0 to 210.4) | 227.6  (200.7 to 245.8) | 15.4%  (6.8 to 23.3) |
|  | **85-89 years** | 26902 (22896 to 29065) | 83606 (69506 to 91868) | 210.8% (190.0 to 231.5) | 267.8 (227.9 to 289.3) | 293.7  (244.1 to 322.7) | 9.7%  (2.3 to 17.0) |
|  | **90-94 years** | 11056 (9035 to 12195) | 46775 (36674 to 52297) | 323.1% (299.3 to 343.8) | 365.3 (298.6 to 403.0) | 387.8  (304.1 to 433.6) | 6.2%  (0.2 to 11.4) |
|  | **95+ years** | 3594 (2706 to 4031) | 18929 (13840 to 21671) | 426.7% (399.5 to 452.9) | 474.2 (357.0 to 531.9) | 480.7  (351.4 to 550.3) | 1.4%  (-3.9 to 6.4) |

MR, age-specific mortality rate; UI, uncertainty interval.

# Supplementary Table 5. The DALYs cases and age-specific DALYs rate of type 2 diabetes mellitus in 1990 and 2021, and its temporal trends from 1990 to 2021, by sex and age groups.

| **Sex** | **Age groups** | **Number of DALYs cases in 1990(95%UI)** | **Number of DALYs cases in 2021**  **(95%UI)** | **Percentage change in DALYs counts, 1990-2021(95%UI)** | **1990 DR per 100000**  **(95%UI)** | **2021 DR per 100000**  **(95%UI)** | **Percentage change in DR, 1990-2021**  **(95%UI)** |
| --- | --- | --- | --- | --- | --- | --- | --- |
| **Both** | **20-24 years** | 311950 (232200 to 419637) | 699556 (494536 to 960375) | 124.3% (104.4 to 142.9) | 63.4  (47.2 to 85.3) | 117.1 (82.8 to 160.8) | 84.8%  (68.5 to 100.2) |
|  | **25-29 years** | 427583 (313238 to 565446) | 1094361 (761010 to 1489425) | 155.9% (136.9 to 173.3) | 96.6  (70.8 to 127.7) | 186.0 (129.3 to 253.2) | 92.5%  (78.2 to 105.6) |
|  | **30-34 years** | 622784 (482997 to 795333) | 1798233 (1301110 to 2423923) | 188.7% (166.0 to 208.0) | 161.6 (125.3 to 206.4) | 297.5 (215.2 to 401.0) | 84.1%  (69.6 to 96.4) |
|  | **35-39 years** | 866048 (675756 to 1096138) | 2455955 (1823923 to 3261662) | 183.6% (165.1 to 201.1) | 245.9 (191.8 to 311.2) | 437.9 (325.2 to 581.5) | 78.1%  (66.5 to 89.1) |
|  | **40-44 years** | 1142617 (913629 to 1418711) | 3473403 (2662960 to 4446035) | 204.0% (184.1 to 222.2) | 398.8 (318.9 to 495.2) | 694.3 (532.3 to 888.8) | 74.1%  (62.7 to 84.5) |
|  | **45-49 years** | 1720855 (1456731 to 2037263) | 5311436 (4289811 to 6623686) | 208.7% (185.7 to 229.4) | 741.1 (627.4 to 877.4) | 1121.7 (906.0 to 1398.9) | 51.4%  (40.1 to 61.6) |
|  | **50-54 years** | 2440548 (2111573 to 2878498) | 7341934 (6024155 to 9118588) | 200.8% (178.6 to 220.1) | 1148.1 (993.3 to 1354.1) | 1650.2 (1354.0 to 2049.5) | 43.7%  (33.1 to 52.9) |
|  | **55-59 years** | 3032032 (2652111 to 3529476) | 9070985 (7599146 to 11114912) | 199.2% (178.4 to 218.4) | 1637.2 (1432.0 to 1905.8) | 2292.2 (1920.3 to 2808.7) | 40.0%  (30.3 to 49.0) |
|  | **60-64 years** | 3616336 (3203962 to 4201045) | 10162374 (8602076 to 12240773) | 181.0% (164.3 to 199.0) | 2251.6 (1994.9 to 2615.7) | 3175.3 (2687.7 to 3824.7) | 41.0%  (32.6 to 50.0) |
|  | **65-69 years** | 3464218 (3084757 to 3940828) | 10459744 (8838210 to 12461495) | 201.9% (183.2 to 222.1) | 2802.6 (2495.6 to 3188.1) | 3791.9 (3204.1 to 4517.6) | 35.3%  (26.9 to 44.3) |
|  | **70-74 years** | 2778303 (2482715 to 3144058) | 8901300 (7543340 to 10517070) | 220.4% (200.6 to 241.4) | 3281.7 (2932.5 to 3713.7) | 4324.4 (3664.7 to 5109.4) | 31.8%  (23.7 to 40.4) |
|  | **75-79 years** | 2211294 (1988954 to 2478022) | 6349261 (5488615 to 7315347) | 187.1% (170.0 to 204.2) | 3592.4 (3231.2 to 4025.7) | 4814.3 (4161.7 to 5546.8) | 34.0%  (26.0 to 42.0) |
|  | **80-84 years** | 1300064 (1160573 to 1457374) | 4285186 (3759117 to 4954364) | 229.6% (211.8 to 247.4) | 3675.0 (3280.7 to 4119.7) | 4892.7 (4292.1 to 5656.8) | 33.1%  (25.9 to 40.3) |
|  | **85-89 years** | 578506 (511672 to 643928) | 2306135 (1997213 to 2631398) | 298.6% (278.7 to 320.7) | 3828.4 (3386.1 to 4261.3) | 5043.8 (4368.2 to 5755.2) | 31.7%  (25.1 to 39.0) |
|  | **90-94 years** | 179098 (154452 to 197626) | 953505 (812635 to 1082293) | 432.4% (404.2 to 460.9) | 4179.5 (3604.3 to 4611.8) | 5330.0 (4542.6 to 6049.9) | 27.5%  (20.8 to 34.4) |
|  | **95+ years** | 46869 (37595 to 52169) | 299831 (242944 to 342925) | 539.7% (509.0 to 577.6) | 4603.7 (3692.7 to 5124.2) | 5501.2 (4457.4 to 6291.8) | 19.5%  (13.8 to 26.6) |
| **Male** | **20-24 years** | 146236 (105247 to 200303) | 356240 (250019 to 496552) | 143.6% (123.7 to 165.7) | 59.0  (42.4 to 80.8) | 117.4 (82.4 to 163.7) | 99.1%  (82.8 to 117.2) |
|  | **25-29 years** | 207072 (144717 to 281366) | 567227 (392210 to 780989) | 173.9% (156.5 to 191.9) | 93.1  (65.0 to 126.4) | 190.8 (131.9 to 262.6) | 105.0% (91.9 to 118.5) |
|  | **30-34 years** | 337200 (260759 to 431403) | 992484 (724640 to 1340344) | 194.3% (170.2 to 215.1) | 172.6 (133.5 to 220.9) | 324.8 (237.2 to 438.7) | 88.1%  (72.7 to 101.4) |
|  | **35-39 years** | 470258 (364774 to 599317) | 1344716 (992605 to 1792590) | 186.0% (166.4 to 203.8) | 263.0 (204.0 to 335.2) | 475.1 (350.7 to 633.3) | 80.6%  (68.3 to 91.9) |
|  | **40-44 years** | 630602 (502954 to 782383) | 1929202 (1470420 to 2479677) | 205.9% (184.1 to 226.7) | 431.2 (343.9 to 534.9) | 765.1 (583.1 to 983.4) | 77.4%  (64.8 to 89.5) |
|  | **45-49 years** | 949029 (797972 to 1124825) | 2940890 (2371149 to 3655085) | 209.9% (181.7 to 232.5) | 801.6 (674.0 to 950.1) | 1236.4 (996.9 to 1536.6) | 54.2%  (40.2 to 65.5) |
|  | **50-54 years** | 1303374 (1130291 to 1534114) | 3992729 (3277307 to 4936971) | 206.3% (178.5 to 229.0) | 1210.7 (1049.9 to 1425.0) | 1798.7 (1476.4 to 2224.1) | 48.6%  (35.1 to 59.6) |
|  | **55-59 years** | 1571040 (1372305 to 1825006) | 4788446 (4018056 to 5838336) | 204.8% (177.3 to 227.2) | 1691.3 (1477.4 to 1964.7) | 2459.1 (2063.5 to 2998.3) | 45.4%  (32.3 to 56.1) |
|  | **60-64 years** | 1780840 (1566055 to 2065000) | 5173180 (4350530 to 6222637) | 190.5% (165.0 to 213.0) | 2267.3 (1993.8 to 2629.0) | 3326.0 (2797.1 to 4000.7) | 46.7%  (33.8 to 58.1) |
|  | **65-69 years** | 1640535 (1445189 to 1863867) | 5200570 (4336525 to 6168767) | 217.0% (188.3 to 243.7) | 2861.5 (2520.8 to 3251.1) | 3944.8 (3289.4 to 4679.2) | 37.9%  (25.4 to 49.5) |
|  | **70-74 years** | 1276353 (1128522 to 1446120) | 4398482 (3704621 to 5198945) | 244.6% (212.4 to 274.8) | 3392.9 (2999.9 to 3844.2) | 4563.1 (3843.3 to 5393.6) | 34.5%  (21.9 to 46.3) |
|  | **75-79 years** | 951207 (853523 to 1071399) | 3027007 (2610365 to 3551020) | 218.2% (192.4 to 245.0) | 3769.7 (3382.6 to 4246.1) | 5063.0 (4366.1 to 5939.5) | 34.3%  (23.4 to 45.6) |
|  | **80-84 years** | 517562 (461471 to 585359) | 1873378 (1627042 to 2175325) | 262.0% (233.3 to 287.9) | 3896.3 (3474.0 to 4406.7) | 5111.3 (4439.2 to 5935.1) | 31.2%  (20.8 to 40.6) |
|  | **85-89 years** | 210220 (187110 to 235799) | 974677 (856519 to 1115211) | 363.6% (328.3 to 396.7) | 4151.2 (3694.9 to 4656.3) | 5649.4 (4964.6 to 6464.0) | 36.1%  (25.7 to 45.8) |
|  | **90-94 years** | 57560 (51155 to 63663) | 357504 (314580 to 404959) | 521.1% (472.4 to 567.7) | 4572.1 (4063.2 to 5056.8) | 6133.7 (5397.3 to 6947.9) | 34.2%  (23.6 to 44.2) |
|  | **95+ years** | 12475 (10472 to 13831) | 90918 (76983 to 104134) | 628.8% (570.4 to 689.2) | 4793.9 (4024.2 to 5314.8) | 6012.9 (5091.3 to 6887.0) | 25.4%  (15.4 to 35.8) |
| **Female** | **20-24 years** | 165714 (122924 to 218153) | 343316 (245963 to 464476) | 107.2% (85.4 to 126.1) | 67.9 (50.4 to 89.4) | 116.9 (83.7 to 158.1) | 72.2%  (54.1 to 87.9) |
|  | **25-29 years** | 220511 (167284 to 289453) | 527135 (372123 to 707421) | 139.1% (116.5 to 159.6) | 100.2 (76.0 to 131.5) | 181.2 (127.9 to 243.1) | 80.8%  (63.7 to 96.4) |
|  | **30-34 years** | 285584 (224686 to 365834) | 805749 (582384 to 1084579) | 182.1% (156.8 to 205.2) | 150.2 (118.2 to 192.4) | 269.5 (194.8 to 362.8) | 79.4%  (63.3 to 94.1) |
|  | **35-39 years** | 395789 (311037 to 502570) | 1111239 (826086 to 1472621) | 180.8% (158.5 to 200.4) | 228.2 (179.3 to 289.7) | 400.0 (297.4 to 530.1) | 75.3%  (61.4 to 87.6) |
|  | **40-44 years** | 512015 (410991 to 634474) | 1544200 (1182943 to 1978941) | 201.6% (179.9 to 222.3) | 365.1 (293.1 to 452.5) | 622.4 (476.8 to 797.7) | 70.5%  (58.2 to 82.2) |
|  | **45-49 years** | 771826 (654184 to 914856) | 2370546 (1883038 to 2980088) | 207.1% (184.0 to 229.7) | 678.2 (574.9 to 803.9) | 1006.0 (799.1 to 1264.7) | 48.3%  (37.2 to 59.2) |
|  | **50-54 years** | 1137174 (978622 to 1342673) | 3349206 (2730678 to 4147452) | 194.5% (170.1 to 216.5) | 1083.9 (932.8 to 1279.8) | 1502.3 (1224.8 to 1860.3) | 38.6%  (27.1 to 49.0) |
|  | **55-59 years** | 1460992 (1265431 to 1707233) | 4282539 (3570736 to 5250157) | 193.1% (171.0 to 214.0) | 1582.7 (1370.8 to 1849.4) | 2130.6 (1776.5 to 2612.0) | 34.6%  (24.4 to 44.2) |
|  | **60-64 years** | 1835496 (1629577 to 2104962) | 4989195 (4217994 to 6056328) | 171.8% (152.5 to 190.8) | 2236.7 (1985.8 to 2565.1) | 3032.7 (2564.0 to 3681.4) | 35.6%  (26.0 to 45.1) |
|  | **65-69 years** | 1823683 (1614977 to 2082557) | 5259174 (4480374 to 6290970) | 188.4% (168.1 to 208.5) | 2751.6 (2436.7 to 3142.1) | 3652.0 (3111.2 to 4368.5) | 32.7%  (23.4 to 42.0) |
|  | **70-74 years** | 1501950 (1335227 to 1702371) | 4502818 (3835344 to 5379469) | 199.8% (178.3 to 220.4) | 3192.7 (2838.3 to 3618.8) | 4114.1 (3504.3 to 4915.1) | 28.9%  (19.6 to 37.7) |
|  | **75-79 years** | 1260087 (1132853 to 1400729) | 3322254 (2889229 to 3864917) | 163.7% (146.0 to 180.5) | 3469.1 (3118.9 to 3856.3) | 4608.0 (4007.4 to 5360.7) | 32.8%  (23.9 to 41.3) |
|  | **80-84 years** | 782502 (693616 to 873947) | 2411808 (2110679 to 2773961) | 208.2% (189.8 to 226.3) | 3541.9 (3139.6 to 3955.9) | 4735.4 (4144.2 to 5446.5) | 33.7%  (25.7 to 41.5) |
|  | **85-89 years** | 368286 (323701 to 408704) | 1331458 (1137528 to 1531696) | 261.5% (243.0 to 281.6) | 3665.6 (3221.9 to 4067.9) | 4676.8 (3995.7 to 5380.2) | 27.6%  (21.1 to 34.7) |
|  | **90-94 years** | 121537 (102787 to 134710) | 596001 (497761 to 684457) | 390.4% (364.3 to 418.6) | 4016.1 (3396.5 to 4451.4) | 4941.6 (4127.1 to 5675.0) | 23.0%  (16.5 to 30.1) |
|  | **95+ years** | 34394 (26847 to 38501) | 208913 (165193 to 239074) | 507.4% (477.7 to 542.8) | 4538.3 (3542.6 to 5080.3) | 5304.7 (4194.5 to 6070.5) | 16.9%  (11.2 to 23.7) |

DR, age-specific DALYs rate; UI, uncertainty interval.

# Supplementary Table 6. The incidence cases and age-standardized incidence rate of type 2 diabetes mellitus in 1990 and 2021, and its temporal trends from 1990 to 2021 in 204 countries/territories.

| **Countries/territories** | **Number of incidence cases in 1990**  **(95%UI)** | **Number of incidence cases in 2021**  **(95%UI)** | **Percentage change in incidence counts, 1990-2021**  **(95%UI)** | **1990 ASIR per 100000**  **(95%UI)** | **2021 ASIR per 100000**  **(95%UI)** | **1990-2021 EAPC (%)**  **(95%CI)** |
| --- | --- | --- | --- | --- | --- | --- |
| **Afghanistan** | 20910 (18785 to 23018) | 135307 (123786 to 147913) | 547.1% (494.4 to 608.4) | 272.4 (246.7 to 300.7) | 654.2 (604.0 to 705.8) | 2.91 (2.87 to 2.95) |
| **Albania** | 3254 (2951 to 3609) | 6577 (5822 to 7424) | 102.1% (87.0 to 118.3) | 127.3 (114.8 to 141.1) | 181.4 (161.3 to 203.8) | 1.17 (1.09 to 1.25) |
| **Algeria** | 32729 (29736 to 35986) | 209799 (192595 to 226849) | 541.0% (492.4 to 598.3) | 207.3 (186.6 to 230.9) | 478.5 (443.0 to 516.4) | 2.77 (2.74 to 2.79) |
| **American Samoa** | 159 (147 to 173) | 453 (419 to 488) | 185.6% (166.4 to 202.8) | 451.1 (419.7 to 486.1) | 863.4 (803.3 to 926.7) | 1.96 (1.82 to 2.09) |
| **Andorra** | 66 (59 to 72) | 261 (234 to 292) | 296.8% (266.3 to 332.0) | 106.4 (95.2 to 117.4) | 211.9 (192.7 to 234.9) | 2.26 (2.22 to 2.31) |
| **Angola** | 10714 (9815 to 11799) | 60603 (55422 to 66669) | 465.6% (428.6 to 503.1) | 195.4 (179.9 to 212.7) | 301.4 (275.9 to 327.5) | 1.47 (1.43 to 1.50) |
| **Antigua and Barbuda** | 167 (157 to 179) | 586 (537 to 641) | 250.4% (228.1 to 280.9) | 331.5 (310.1 to 358.0) | 517.3 (477.0 to 562.6) | 1.42 (1.40 to 1.45) |
| **Argentina** | 53613 (50626 to 57456) | 149119 (135324 to 164155) | 178.1% (156.4 to 199.7) | 165.0 (155.8 to 176.8) | 291.6 (264.8 to 321.0) | 1.75 (1.67 to 1.82) |
| **Armenia** | 4931 (4539 to 5451) | 8895 (7982 to 9932) | 80.4% (66.6 to 95.4) | 152.5 (141.4 to 167.8) | 235.3 (213.8 to 259.5) | 1.27 (1.05 to 1.50) |
| **Australia** | 19688 (18152 to 21500) | 63634 (56848 to 69985) | 223.2% (203.3 to 247.5) | 104.9 (96.6 to 114.4) | 184.9 (165.3 to 201.8) | 1.84 (1.79 to 1.90) |
| **Austria** | 7633 (7154 to 8096) | 17784 (16141 to 19207) | 133.0% (118.3 to 150.3) | 74.2 (69.2 to 79.6) | 141.1 (128.9 to 152.4) | 2.06 (1.97 to 2.14) |
| **Azerbaijan** | 6101 (5566 to 6658) | 28458 (26190 to 31300) | 366.5% (330.4 to 404.1) | 99.5 (91.1 to 108.4) | 227.0 (210.5 to 247.2) | 2.79 (2.66 to 2.92) |
| **Bahrain** | 1234 (1119 to 1345) | 13523 (12547 to 14628) | 995.7% (916.2 to 1074.4) | 399.1 (371.6 to 425.0) | 766.7 (725.2 to 812.1) | 2.10 (1.97 to 2.22) |
| **Bangladesh** | 107334 (99143 to 116023) | 493818 (462045 to 531970) | 360.1% (334.8 to 388.3) | 160.3 (149.3 to 171.4) | 303.1 (283.9 to 324.8) | 2.10 (2.06 to 2.15) |
| **Barbados** | 852 (809 to 900) | 2037 (1920 to 2173) | 139.0% (126.9 to 153.6) | 340.8 (320.4 to 364.4) | 487.5 (458.8 to 518.7) | 1.01 (0.96 to 1.07) |
| **Belarus** | 9800 (8809 to 11023) | 16774 (14915 to 18744) | 71.2% (55.7 to 86.1) | 79.4 (72.4 to 88.6) | 129.5 (116.4 to 141.7) | 1.39 (1.31 to 1.47) |
| **Belgium** | 14962 (13830 to 16342) | 32471 (28655 to 36566) | 117.0% (97.8 to 140.9) | 117.9 (108.5 to 129.0) | 217.4 (194.0 to 241.5) | 1.91 (1.84 to 1.98) |
| **Belize** | 267 (250 to 287) | 1657 (1522 to 1826) | 520.6% (474.2 to 577.1) | 243.4 (228.3 to 261.6) | 422.2 (388.8 to 464.0) | 1.83 (1.77 to 1.89) |
| **Benin** | 4165 (3797 to 4558) | 25731 (23498 to 28311) | 517.8% (482.7 to 556.0) | 165.3 (149.6 to 180.7) | 307.0 (280.9 to 337.4) | 1.92 (1.83 to 2.00) |
| **Bermuda** | 122 (112 to 132) | 290 (263 to 316) | 137.2% (119.0 to 156.9) | 184.9 (170.0 to 199.6) | 297.6 (271.1 to 323.3) | 1.45 (1.41 to 1.50) |
| **Bhutan** | 542 (490 to 594) | 1825 (1684 to 1961) | 236.6% (215.9 to 257.2) | 139.4 (127.6 to 152.3) | 242.3 (224.4 to 260.7) | 1.89 (1.84 to 1.94) |
| **Bolivia** | 7025 (6496 to 7592) | 35942 (32956 to 39201) | 411.6% (384.3 to 441.2) | 176.5 (163.5 to 190.5) | 337.0 (309.6 to 367.5) | 2.15 (2.12 to 2.19) |
| **Bosnia and Herzegovina** | 8897 (8012 to 9765) | 18772 (17081 to 20419) | 111.0% (94.1 to 130.5) | 181.9 (165.0 to 198.6) | 373.3 (342.5 to 401.6) | 2.61 (2.42 to 2.79) |
| **Botswana** | 1164 (1071 to 1263) | 5649 (5251 to 6062) | 385.2% (356.8 to 415.3) | 175.2 (161.0 to 189.5) | 295.6 (277.0 to 314.3) | 1.79 (1.75 to 1.83) |
| **Brazil** | 242405 (219037 to 266021) | 718517 (646384 to 799051) | 196.4% (184.6 to 208.8) | 216.1 (194.9 to 237.7) | 279.1 (251.6 to 309.3) | 0.90 (0.85 to 0.96) |
| **Brunei** | 548 (510 to 585) | 3430 (3185 to 3658) | 525.8% (489.0 to 566.4) | 366.1 (346.9 to 387.7) | 676.6 (632.2 to 721.3) | 1.71 (1.60 to 1.82) |
| **Bulgaria** | 20451 (18227 to 23037) | 28995 (26344 to 31708) | 41.8% (29.9 to 56.7) | 174.2 (157.9 to 193.3) | 289.6 (264.9 to 311.7) | 1.70 (1.54 to 1.86) |
| **Burkina Faso** | 6649 (6173 to 7187) | 30212 (27648 to 32620) | 354.4% (332.1 to 382.5) | 132.8 (123.9 to 142.2) | 227.2 (208.6 to 243.6) | 1.75 (1.66 to 1.85) |
| **Burundi** | 3717 (3470 to 3996) | 12091 (11144 to 13030) | 225.3% (206.6 to 243.9) | 132.8 (124.1 to 142.8) | 169.5 (156.6 to 182.3) | 0.72 (0.68 to 0.75) |
| **Cambodia** | 7277 (6740 to 7880) | 43678 (40029 to 47511) | 500.2% (461.1 to 546.9) | 129.0 (119.8 to 138.7) | 294.2 (267.9 to 320.1) | 2.77 (2.74 to 2.81) |
| **Cameroon** | 8316 (7601 to 9044) | 50577 (46784 to 54823) | 508.2% (472.7 to 549.0) | 149.0 (138.2 to 160.8) | 258.5 (240.9 to 277.7) | 1.88 (1.79 to 1.96) |
| **Canada** | 38425 (35510 to 41666) | 180342 (160672 to 201112) | 369.3% (327.2 to 414.5) | 123.0 (113.5 to 133.4) | 326.7 (292.9 to 361.2) | 2.93 (2.86 to 3.00) |
| **Cape Verde** | 370 (337 to 405) | 1770 (1616 to 1936) | 378.8% (344.6 to 412.6) | 152.4 (139.0 to 167.1) | 331.2 (302.7 to 361.0) | 2.74 (2.63 to 2.86) |
| **Central African Republic** | 3363 (3083 to 3685) | 13095 (11931 to 14248) | 289.3% (267.7 to 313.7) | 212.7 (195.2 to 232.7) | 335.1 (307.2 to 362.8) | 1.51 (1.47 to 1.55) |
| **Chad** | 4626 (4247 to 5041) | 22473 (20485 to 24504) | 385.8% (358.3 to 411.8) | 136.0 (124.7 to 148.6) | 241.9 (220.4 to 263.2) | 1.84 (1.70 to 1.98) |
| **Chile** | 18242 (16635 to 20102) | 84065 (75356 to 93282) | 360.8% (324.5 to 397.0) | 160.3 (145.6 to 176.9) | 352.9 (319.7 to 391.4) | 2.64 (2.53 to 2.75) |
| **China** | 1814230 (1625010 to 2028169) | 3971486 (3572723 to 4410205) | 118.9% (104.1 to 133.5) | 161.5 (143.0 to 180.1) | 241.9 (221.0 to 263.7) | 1.10 (1.01 to 1.19) |
| **Colombia** | 50680 (46686 to 55766) | 155547 (144776 to 166483) | 206.9% (182.5 to 231.6) | 209.9 (193.2 to 230.9) | 284.3 (264.2 to 304.7) | 0.67 (0.56 to 0.78) |
| **Comoros** | 375 (348 to 409) | 1423 (1318 to 1541) | 279.0% (257.4 to 304.1) | 160.0 (148.3 to 174.7) | 236.1 (219.1 to 255.9) | 1.28 (1.25 to 1.31) |
| **Congo** | 2482 (2283 to 2681) | 12047 (11037 to 13159) | 385.4% (351.5 to 421.5) | 184.6 (172.2 to 196.9) | 294.8 (273.6 to 318.2) | 1.58 (1.54 to 1.62) |
| **Cook Islands** | 80 (75 to 86) | 160 (149 to 172) | 100.1% (89.0 to 112.7) | 513.0 (479.7 to 548.7) | 806.4 (752.3 to 857.5) | 1.25 (1.15 to 1.34) |
| **Costa Rica** | 4553 (4110 to 4975) | 18534 (16872 to 20108) | 307.1% (279.4 to 337.8) | 205.4 (187.1 to 226.8) | 343.6 (314.6 to 372.5) | 1.66 (1.57 to 1.74) |
| **Cote d'Ivoire** | 9492 (8610 to 10382) | 48818 (44501 to 53314) | 414.3% (384.8 to 448.8) | 159.0 (146.1 to 172.3) | 274.2 (251.5 to 301.2) | 1.81 (1.76 to 1.85) |
| **Croatia** | 11620 (10290 to 12881) | 17066 (15428 to 18625) | 46.9% (36.9 to 57.7) | 182.7 (162.9 to 202.7) | 270.1 (245.2 to 296.5) | 1.25 (1.14 to 1.37) |
| **Cuba** | 19770 (18323 to 21593) | 45250 (40552 to 49405) | 128.9% (109.5 to 147.1) | 182.8 (168.7 to 201.0) | 288.1 (262.0 to 314.0) | 1.34 (1.28 to 1.41) |
| **Cyprus** | 2449 (2334 to 2577) | 5380 (4986 to 5864) | 119.7% (106.6 to 133.1) | 290.5 (275.1 to 308.4) | 303.1 (280.3 to 331.2) | -0.07 (-0.17 to 0.03) |
| **Czech Republic** | 22052 (19858 to 24481) | 45371 (40970 to 49486) | 105.8% (90.1 to 122.4) | 174.3 (157.5 to 193.1) | 291.7 (264.5 to 317.3) | 1.80 (1.77 to 1.84) |
| **Democratic Republic of the Congo** | 31633 (29126 to 34406) | 140622 (128003 to 153553) | 344.5% (319.0 to 372.1) | 156.8 (144.7 to 169.3) | 239.2 (218.8 to 258.3) | 1.39 (1.34 to 1.44) |
| **Denmark** | 5379 (5026 to 5764) | 14314 (12989 to 16100) | 166.1% (146.7 to 189.7) | 83.5 (77.6 to 89.7) | 183.0 (166.3 to 203.0) | 2.57 (2.55 to 2.59) |
| **Djibouti** | 205 (188 to 224) | 1619 (1469 to 1766) | 690.3% (643.7 to 744.8) | 115.0 (106.9 to 124.8) | 186.4 (173.0 to 200.3) | 1.61 (1.59 to 1.64) |
| **Dominica** | 197 (186 to 210) | 432 (397 to 468) | 119.1% (106.6 to 134.1) | 338.8 (318.2 to 364.7) | 532.4 (490.2 to 578.3) | 1.40 (1.37 to 1.43) |
| **Dominican Republic** | 10102 (9218 to 11075) | 46798 (42506 to 51134) | 363.2% (330.8 to 395.7) | 205.0 (186.8 to 225.2) | 424.3 (386.6 to 463.7) | 2.45 (2.40 to 2.51) |
| **Ecuador** | 11548 (10660 to 12697) | 67077 (61911 to 72349) | 480.9% (443.7 to 523.4) | 175.6 (162.2 to 193.1) | 385.0 (355.4 to 416.4) | 2.54 (2.41 to 2.67) |
| **Egypt** | 44834 (41382 to 48609) | 378556 (342012 to 416907) | 744.4% (691.7 to 798.6) | 120.7 (111.1 to 130.7) | 420.5 (381.5 to 462.9) | 4.30 (4.21 to 4.40) |
| **El Salvador** | 5959 (5487 to 6560) | 19939 (18241 to 21869) | 234.6% (214.7 to 256.5) | 161.1 (148.0 to 177.5) | 321.0 (293.7 to 352.7) | 2.12 (2.02 to 2.22) |
| **Equatorial Guinea** | 429 (395 to 465) | 3048 (2782 to 3338) | 611.2% (567.1 to 659.3) | 176.2 (163.6 to 190.6) | 331.5 (308.6 to 356.2) | 2.28 (2.20 to 2.36) |
| **Eritrea** | 2213 (2032 to 2425) | 9513 (8728 to 10308) | 329.9% (305.1 to 356.8) | 145.8 (134.4 to 158.0) | 220.9 (204.1 to 238.0) | 1.36 (1.34 to 1.38) |
| **Estonia** | 1941 (1762 to 2152) | 3582 (3211 to 3947) | 84.6% (71.8 to 99.6) | 102.9 (93.4 to 114.5) | 206.2 (184.6 to 227.1) | 2.21 (2.13 to 2.29) |
| **Ethiopia** | 42886 (39190 to 46896) | 118170 (107637 to 129257) | 175.5% (164.5 to 186.0) | 170.9 (156.4 to 187.0) | 187.8 (171.5 to 205.6) | 0.15 (0.08 to 0.23) |
| **Federated States of Micronesia** | 236 (221 to 252) | 664 (626 to 704) | 181.4% (166.2 to 197.6) | 351.9 (331.7 to 372.8) | 661.9 (628.4 to 697.3) | 2.16 (2.01 to 2.31) |
| **Fiji** | 2546 (2388 to 2714) | 7746 (7380 to 8112) | 204.2% (187.7 to 222.9) | 506.2 (482.9 to 531.2) | 828.8 (794.1 to 863.2) | 1.43 (1.32 to 1.53) |
| **Finland** | 9328 (8481 to 10344) | 19395 (17626 to 21594) | 107.9% (93.0 to 125.7) | 153.4 (140.1 to 169.1) | 277.6 (254.3 to 304.5) | 1.83 (1.72 to 1.94) |
| **France** | 66580 (62014 to 72003) | 139495 (123967 to 153120) | 109.5% (93.9 to 124.5) | 95.9 (89.6 to 103.6) | 162.3 (146.9 to 178.9) | 1.77 (1.70 to 1.83) |
| **Gabon** | 1361 (1261 to 1477) | 5110 (4743 to 5481) | 275.4% (255.0 to 298.9) | 208.6 (194.2 to 224.6) | 358.4 (335.2 to 382.8) | 1.84 (1.77 to 1.91) |
| **Georgia** | 6972 (6292 to 7704) | 11481 (10438 to 12603) | 64.7% (51.4 to 76.9) | 112.9 (102.1 to 125.2) | 260.7 (238.1 to 284.2) | 2.87 (2.77 to 2.97) |
| **Germany** | 108981 (101719 to 116573) | 261658 (237913 to 294191) | 140.1% (122.0 to 162.3) | 99.3 (92.4 to 106.4) | 218.6 (200.0 to 241.8) | 2.49 (2.31 to 2.68) |
| **Ghana** | 12096 (11015 to 13226) | 64874 (58903 to 70351) | 436.3% (404.7 to 470.3) | 143.4 (131.8 to 155.9) | 265.8 (243.8 to 289.6) | 2.02 (1.85 to 2.19) |
| **Greece** | 18353 (16456 to 20290) | 28422 (25464 to 31385) | 54.9% (42.6 to 66.3) | 140.2 (128.0 to 154.6) | 219.9 (198.8 to 241.2) | 1.33 (1.25 to 1.40) |
| **Greenland** | 26 (24 to 29) | 149 (133 to 165) | 463.4% (404.5 to 527.9) | 59.4 (54.7 to 63.9) | 195.8 (176.9 to 215.8) | 4.03 (3.80 to 4.25) |
| **Grenada** | 223 (209 to 237) | 684 (627 to 742) | 207.6% (185.5 to 228.4) | 346.8 (322.2 to 372.3) | 560.6 (516.4 to 603.9) | 1.52 (1.47 to 1.58) |
| **Guam** | 265 (244 to 289) | 653 (592 to 721) | 146.9% (128.6 to 166.9) | 234.2 (217.5 to 254.5) | 365.8 (333.1 to 403.3) | 1.32 (1.24 to 1.40) |
| **Guatemala** | 9032 (8345 to 9719) | 60781 (56580 to 65239) | 572.9% (532.7 to 619.9) | 180.7 (167.4 to 195.3) | 444.5 (414.0 to 478.9) | 3.14 (2.89 to 3.39) |
| **Guinea** | 4956 (4566 to 5391) | 18060 (16698 to 19586) | 264.4% (242.5 to 287.9) | 131.6 (121.6 to 143.3) | 224.8 (207.5 to 243.1) | 1.59 (1.50 to 1.67) |
| **Guinea-Bissau** | 934 (847 to 1016) | 3653 (3341 to 3985) | 290.9% (271.5 to 315.7) | 173.9 (159.2 to 189.0) | 292.8 (269.9 to 317.6) | 1.72 (1.68 to 1.75) |
| **Guyana** | 2422 (2233 to 2649) | 5512 (5148 to 5946) | 127.6% (113.8 to 142.8) | 455.1 (421.1 to 494.6) | 713.8 (667.2 to 769.3) | 1.40 (1.34 to 1.46) |
| **Haiti** | 14706 (13560 to 15820) | 56885 (51876 to 62644) | 286.8% (264.6 to 310.5) | 341.4 (316.2 to 367.0) | 524.5 (479.5 to 576.6) | 1.34 (1.32 to 1.36) |
| **Honduras** | 5981 (5432 to 6566) | 33571 (30622 to 37277) | 461.3% (428.4 to 498.8) | 208.3 (188.3 to 227.2) | 391.0 (356.0 to 435.1) | 2.12 (2.03 to 2.21) |
| **Hungary** | 25021 (22175 to 27477) | 38602 (34285 to 42786) | 54.3% (41.5 to 64.4) | 186.5 (167.9 to 204.0) | 274.7 (246.9 to 303.2) | 1.21 (0.98 to 1.44) |
| **Iceland** | 247 (225 to 271) | 878 (803 to 961) | 255.8% (232.7 to 285.4) | 93.4 (85.1 to 102.8) | 205.1 (188.3 to 225.3) | 2.55 (2.51 to 2.59) |
| **India** | 998569 (903408 to 1106291) | 3719320 (3367369 to 4097805) | 272.5% (262.7 to 284.9) | 157.8 (143.3 to 174.1) | 258.7 (234.9 to 283.7) | 1.57 (1.52 to 1.62) |
| **Indonesia** | 185427 (168257 to 204492) | 795495 (714514 to 879985) | 329.0% (310.5 to 347.5) | 145.8 (132.1 to 161.1) | 271.1 (244.9 to 299.7) | 2.08 (2.00 to 2.17) |
| **Iran** | 51961 (46518 to 57544) | 326417 (290596 to 363434) | 528.2% (501.1 to 558.2) | 148.2 (132.8 to 164.0) | 343.7 (307.7 to 381.1) | 2.89 (2.83 to 2.94) |
| **Iraq** | 37935 (34842 to 41016) | 257868 (237549 to 281145) | 579.8% (539.2 to 629.2) | 341.3 (311.1 to 371.9) | 694.1 (641.2 to 750.7) | 2.40 (2.35 to 2.46) |
| **Ireland** | 4200 (3810 to 4650) | 11435 (10311 to 12530) | 172.2% (150.9 to 196.9) | 113.7 (102.6 to 125.7) | 185.5 (167.3 to 203.8) | 1.66 (1.58 to 1.74) |
| **Israel** | 8290 (7765 to 8835) | 27433 (25347 to 29559) | 230.9% (208.9 to 248.9) | 182.6 (169.9 to 196.4) | 271.3 (248.8 to 294.5) | 1.28 (1.07 to 1.49) |
| **Italy** | 119784 (106903 to 133829) | 189382 (165658 to 217555) | 58.1% (49.9 to 68.3) | 159.5 (143.2 to 177.2) | 211.2 (186.2 to 240.7) | 0.83 (0.72 to 0.94) |
| **Jamaica** | 4844 (4566 to 5127) | 13169 (12317 to 14183) | 171.8% (157.2 to 186.9) | 279.7 (262.4 to 297.7) | 426.3 (400.2 to 458.8) | 1.27 (1.21 to 1.33) |
| **Japan** | 304565 (269645 to 341417) | 507606 (450603 to 570203) | 66.7% (57.0 to 77.2) | 186.8 (165.8 to 209.3) | 278.8 (249.2 to 312.0) | 0.94 (0.80 to 1.07) |
| **Jordan** | 7570 (7084 to 8118) | 74940 (69478 to 81287) | 890.0% (828.9 to 950.2) | 380.4 (357.2 to 405.2) | 653.8 (609.6 to 702.4) | 1.81 (1.75 to 1.88) |
| **Kazakhstan** | 19593 (17547 to 22208) | 57084 (52686 to 61496) | 191.4% (161.4 to 220.3) | 131.3 (117.4 to 149.1) | 281.9 (261.0 to 302.8) | 2.65 (2.59 to 2.71) |
| **Kenya** | 10151 (9283 to 11086) | 39803 (36532 to 43543) | 292.1% (279.0 to 305.7) | 102.2 (93.9 to 112.1) | 134.6 (123.5 to 147.5) | 0.86 (0.80 to 0.92) |
| **Kiribati** | 225 (210 to 241) | 736 (692 to 785) | 227.6% (211.9 to 242.7) | 423.2 (398.6 to 447.6) | 687.1 (650.2 to 725.5) | 1.55 (1.43 to 1.67) |
| **Kuwait** | 4428 (3997 to 4894) | 39969 (35993 to 44292) | 802.6% (717.6 to 899.3) | 368.5 (338.5 to 400.7) | 703.2 (645.8 to 767.0) | 2.25 (2.19 to 2.32) |
| **Kyrgyzstan** | 3123 (2833 to 3392) | 11680 (10590 to 13151) | 274.0% (246.5 to 309.1) | 89.1 (81.1 to 96.8) | 182.1 (164.9 to 205.4) | 2.39 (2.29 to 2.50) |
| **Laos** | 4264 (3943 to 4610) | 20659 (19059 to 22406) | 384.5% (356.7 to 412.4) | 169.0 (156.2 to 182.8) | 350.4 (322.5 to 382.8) | 2.37 (2.36 to 2.38) |
| **Latvia** | 3080 (2777 to 3363) | 5160 (4609 to 5821) | 67.5% (55.1 to 81.1) | 95.3 (86.8 to 103.7) | 204.6 (185.9 to 226.0) | 2.51 (2.41 to 2.61) |
| **Lebanon** | 6757 (6145 to 7394) | 30574 (28117 to 33038) | 352.5% (322.6 to 382.6) | 266.6 (242.4 to 291.3) | 527.1 (484.5 to 569.6) | 2.29 (2.24 to 2.34) |
| **Lesotho** | 1308 (1216 to 1407) | 4187 (3931 to 4448) | 220.2% (200.0 to 237.9) | 141.8 (132.7 to 152.3) | 316.0 (297.7 to 334.4) | 2.89 (2.80 to 2.97) |
| **Liberia** | 2189 (2009 to 2394) | 10021 (9071 to 10969) | 357.8% (326.7 to 392.7) | 153.8 (140.9 to 168.3) | 278.4 (253.9 to 303.4) | 2.02 (1.99 to 2.06) |
| **Libya** | 5115 (4666 to 5638) | 39276 (35678 to 43794) | 667.9% (615.8 to 747.2) | 198.4 (179.9 to 218.8) | 499.7 (457.2 to 549.5) | 3.20 (3.07 to 3.32) |
| **Lithuania** | 3681 (3315 to 4102) | 6329 (5681 to 7082) | 71.9% (57.1 to 87.5) | 86.4 (78.3 to 95.7) | 171.0 (155.9 to 187.6) | 2.11 (2.03 to 2.18) |
| **Luxembourg** | 565 (521 to 617) | 1739 (1577 to 1928) | 208.0% (185.7 to 228.4) | 117.3 (107.9 to 128.3) | 207.2 (188.6 to 228.2) | 1.83 (1.78 to 1.88) |
| **Macedonia** | 4624 (4177 to 5101) | 11914 (10719 to 13014) | 157.6% (138.7 to 177.2) | 220.9 (199.2 to 243.0) | 373.7 (337.2 to 407.3) | 1.77 (1.63 to 1.90) |
| **Madagascar** | 6615 (6105 to 7165) | 26038 (23822 to 28162) | 293.6% (270.2 to 316.4) | 110.2 (102.3 to 119.5) | 155.5 (143.1 to 167.6) | 1.10 (1.08 to 1.11) |
| **Malawi** | 5002 (4662 to 5383) | 13991 (13074 to 15048) | 179.7% (164.4 to 196.2) | 114.5 (107.9 to 122.1) | 142.3 (133.3 to 151.6) | 0.63 (0.60 to 0.66) |
| **Malaysia** | 28340 (26316 to 30872) | 130888 (119991 to 141643) | 361.8% (331.6 to 391.8) | 241.6 (224.5 to 260.2) | 406.6 (371.7 to 441.0) | 1.65 (1.61 to 1.70) |
| **Maldives** | 206 (187 to 227) | 1414 (1280 to 1571) | 588.0% (533.4 to 651.1) | 179.3 (162.9 to 197.2) | 309.9 (280.4 to 339.5) | 1.77 (1.71 to 1.83) |
| **Mali** | 11544 (10394 to 12684) | 57448 (52474 to 63655) | 397.6% (365.2 to 431.3) | 224.9 (203.3 to 246.3) | 405.1 (370.9 to 444.1) | 1.96 (1.89 to 2.03) |
| **Malta** | 578 (537 to 624) | 1883 (1692 to 2068) | 225.7% (202.5 to 248.8) | 138.2 (128.5 to 149.4) | 313.7 (283.2 to 341.6) | 2.65 (2.35 to 2.95) |
| **Marshall Islands** | 137 (126 to 148) | 501 (465 to 545) | 266.3% (243.5 to 288.9) | 484.6 (450.4 to 522.5) | 877.7 (819.3 to 947.0) | 1.96 (1.88 to 2.04) |
| **Mauritania** | 1543 (1431 to 1666) | 5199 (4902 to 5462) | 236.8% (217.7 to 254.4) | 129.3 (120.3 to 139.4) | 184.0 (174.1 to 192.6) | 1.09 (0.96 to 1.22) |
| **Mauritius** | 2723 (2550 to 2920) | 11383 (10643 to 12185) | 318.1% (295.1 to 344.4) | 307.3 (286.5 to 330.6) | 629.4 (591.1 to 672.3) | 2.38 (2.23 to 2.52) |
| **Mexico** | 229138 (210184 to 250088) | 597882 (544430 to 652781) | 160.9% (152.9 to 169.6) | 389.0 (353.4 to 427.2) | 430.6 (393.8 to 467.1) | 0.16 (0.10 to 0.21) |
| **Moldova** | 5878 (5348 to 6570) | 11378 (10075 to 12624) | 93.6% (78.5 to 108.6) | 125.3 (114.0 to 139.2) | 229.3 (205.6 to 251.8) | 1.85 (1.74 to 1.96) |
| **Mongolia** | 1105 (1007 to 1214) | 5605 (5113 to 6136) | 407.5% (371.8 to 443.2) | 75.4 (68.6 to 83.6) | 170.1 (155.8 to 185.4) | 2.76 (2.68 to 2.85) |
| **Montenegro** | 1351 (1200 to 1503) | 2612 (2355 to 2887) | 93.4% (78.3 to 109.3) | 203.3 (181.6 to 224.7) | 306.1 (277.2 to 337.0) | 1.32 (1.25 to 1.40) |
| **Morocco** | 43316 (39460 to 47809) | 250420 (229830 to 273554) | 478.1% (441.7 to 514.6) | 243.5 (220.8 to 269.9) | 632.7 (581.3 to 690.8) | 3.20 (3.14 to 3.25) |
| **Mozambique** | 7880 (7343 to 8440) | 31562 (28923 to 34281) | 300.5% (276.8 to 327.1) | 119.4 (112.7 to 127.2) | 193.9 (179.9 to 207.1) | 1.67 (1.63 to 1.72) |
| **Myanmar** | 65974 (60707 to 71661) | 250725 (230675 to 272578) | 280.0% (255.4 to 305.1) | 234.2 (215.3 to 253.5) | 450.5 (413.5 to 490.8) | 2.09 (2.07 to 2.11) |
| **Namibia** | 1311 (1212 to 1427) | 4279 (3907 to 4666) | 226.4% (207.8 to 247.6) | 173.8 (161.5 to 187.4) | 242.5 (222.6 to 263.6) | 1.08 (1.06 to 1.09) |
| **Nepal** | 21237 (19377 to 23380) | 88932 (81805 to 96556) | 318.8% (294.1 to 346.1) | 159.8 (146.0 to 175.0) | 302.0 (277.8 to 327.5) | 2.10 (2.01 to 2.19) |
| **Netherlands** | 22514 (20999 to 24162) | 43792 (39325 to 49272) | 94.5% (75.6 to 114.4) | 124.6 (116.2 to 134.0) | 184.5 (166.7 to 204.7) | 1.17 (1.12 to 1.22) |
| **New Zealand** | 5317 (4772 to 5924) | 14579 (13783 to 15413) | 174.2% (152.8 to 200.4) | 148.3 (132.4 to 165.5) | 217.8 (206.7 to 228.8) | 1.63 (1.37 to 1.90) |
| **Nicaragua** | 5292 (4843 to 5783) | 24825 (22934 to 27029) | 369.1% (338.1 to 401.0) | 243.7 (225.3 to 267.2) | 408.9 (377.7 to 444.4) | 1.68 (1.63 to 1.73) |
| **Niger** | 5507 (4998 to 6009) | 31326 (28433 to 34496) | 468.9% (435.0 to 510.3) | 141.1 (128.5 to 153.1) | 238.8 (217.4 to 261.0) | 1.72 (1.71 to 1.74) |
| **Nigeria** | 72729 (66092 to 79798) | 267509 (243565 to 291800) | 267.8% (252.7 to 281.3) | 136.4 (124.1 to 150.6) | 203.1 (185.7 to 222.0) | 1.26 (1.22 to 1.30) |
| **North Korea** | 25328 (22778 to 28225) | 76288 (69463 to 84652) | 201.2% (182.4 to 224.8) | 125.2 (113.0 to 139.2) | 237.0 (217.4 to 261.2) | 2.04 (2.02 to 2.05) |
| **Northern Mariana Islands** | 92 (83 to 103) | 247 (224 to 271) | 169.3% (143.1 to 195.7) | 247.8 (228.2 to 269.5) | 429.1 (394.7 to 468.1) | 1.61 (1.55 to 1.68) |
| **Norway** | 7857 (7052 to 8780) | 13342 (11785 to 15060) | 69.8% (62.0 to 77.5) | 153.1 (136.9 to 171.6) | 192.1 (170.5 to 215.6) | 0.61 (0.49 to 0.73) |
| **Oman** | 2697 (2427 to 2958) | 18639 (16777 to 20930) | 591.1% (538.1 to 646.5) | 237.4 (218.8 to 255.4) | 452.4 (417.7 to 491.9) | 1.95 (1.91 to 1.99) |
| **Pakistan** | 121280 (110562 to 134071) | 635449 (576554 to 699928) | 424.0% (402.4 to 451.7) | 166.0 (151.7 to 184.0) | 341.1 (312.4 to 374.3) | 2.61 (2.42 to 2.79) |
| **Palestine** | 2740 (2545 to 2934) | 19092 (17666 to 20624) | 596.9% (553.6 to 641.1) | 259.6 (241.7 to 277.6) | 509.5 (472.9 to 548.3) | 2.32 (2.27 to 2.36) |
| **Panama** | 3539 (3236 to 3868) | 15232 (13881 to 16671) | 330.4% (301.9 to 367.4) | 193.8 (177.9 to 213.4) | 343.8 (313.2 to 375.9) | 1.91 (1.86 to 1.97) |
| **Papua New Guinea** | 8235 (7592 to 8871) | 46738 (43352 to 50339) | 467.5% (435.6 to 497.4) | 300.6 (280.6 to 320.9) | 536.8 (502.6 to 571.4) | 1.85 (1.78 to 1.93) |
| **Paraguay** | 5001 (4599 to 5440) | 23595 (21946 to 25568) | 371.8% (342.2 to 406.5) | 186.7 (171.3 to 202.9) | 353.4 (327.6 to 382.4) | 2.15 (2.03 to 2.26) |
| **Peru** | 15979 (14650 to 17587) | 70259 (64912 to 76216) | 339.7% (313.1 to 372.4) | 109.3 (99.7 to 120.4) | 197.5 (182.6 to 213.8) | 1.96 (1.93 to 2.00) |
| **Philippines** | 75422 (68899 to 83226) | 255789 (232375 to 281891) | 239.1% (225.2 to 254.4) | 210.0 (191.4 to 233.2) | 262.6 (238.4 to 290.8) | 0.79 (0.71 to 0.87) |
| **Poland** | 84669 (74881 to 94316) | 153426 (136163 to 172103) | 81.2% (73.9 to 89.4) | 195.8 (173.2 to 217.3) | 283.1 (251.8 to 317.5) | 1.25 (1.17 to 1.34) |
| **Portugal** | 21422 (19624 to 23215) | 50119 (45224 to 56247) | 134.0% (115.3 to 154.8) | 174.2 (161.0 to 189.0) | 338.6 (308.0 to 374.6) | 2.09 (1.94 to 2.24) |
| **Principality of Monaco** | 37 (33 to 41) | 96 (85 to 109) | 160.4% (139.7 to 182.6) | 84.1 (75.1 to 93.1) | 182.3 (164.6 to 203.1) | 2.54 (2.51 to 2.58) |
| **Puerto Rico** | 11943 (11141 to 12937) | 23128 (21260 to 25230) | 93.7% (82.7 to 107.8) | 334.0 (310.9 to 362.0) | 525.3 (486.6 to 574.5) | 1.44 (1.30 to 1.59) |
| **Qatar** | 1049 (929 to 1164) | 22561 (19653 to 25256) | 2051.4% (1849.7 to 2319.1) | 426.1 (399.2 to 455.8) | 778.1 (724.9 to 823.4) | 1.81 (1.70 to 1.93) |
| **Republic of Nauru** | 30 (28 to 33) | 68 (63 to 72) | 124.2% (112.1 to 138.2) | 424.9 (398.2 to 453.1) | 712.7 (672.2 to 755.1) | 1.54 (1.42 to 1.65) |
| **Republic of Niue** | 9 (8 to 9) | 14 (13 to 15) | 65.7% (56.3 to 75.2) | 414.6 (386.8 to 446.3) | 768.3 (713.5 to 825.0) | 1.92 (1.84 to 1.99) |
| **Republic of Palau** | 57 (53 to 61) | 172 (161 to 183) | 202.6% (181.4 to 225.1) | 432.8 (403.3 to 462.9) | 738.1 (695.8 to 779.5) | 1.70 (1.66 to 1.75) |
| **Republic of San Marino** | 30 (27 to 33) | 92 (82 to 104) | 209.6% (191.7 to 232.1) | 101.5 (91.6 to 111.7) | 203.9 (184.3 to 226.5) | 2.30 (2.25 to 2.35) |
| **Romania** | 34037 (29728 to 38820) | 49202 (44508 to 54651) | 44.6% (30.9 to 58.7) | 124.2 (109.7 to 140.9) | 184.2 (167.2 to 203.6) | 1.35 (1.27 to 1.42) |
| **Russian Federation** | 176154 (154590 to 197698) | 385982 (342412 to 432755) | 119.1% (111.1 to 127.7) | 98.7 (87.0 to 110.9) | 193.6 (172.7 to 216.3) | 2.18 (2.10 to 2.26) |
| **Rwanda** | 4233 (3921 to 4546) | 11919 (10965 to 12886) | 181.6% (164.1 to 200.7) | 128.5 (120.6 to 136.5) | 146.8 (136.3 to 157.1) | 0.28 (0.19 to 0.36) |
| **Saint Kitts and Nevis** | 110 (103 to 117) | 393 (358 to 439) | 258.3% (226.1 to 288.5) | 334.4 (311.4 to 358.1) | 499.4 (456.7 to 549.2) | 1.26 (1.23 to 1.29) |
| **Saint Lucia** | 421 (396 to 448) | 1324 (1221 to 1417) | 214.6% (195.8 to 234.1) | 442.9 (414.9 to 473.4) | 571.4 (531.1 to 609.1) | 0.76 (0.73 to 0.80) |
| **Saint Vincent and the Grenadines** | 310 (292 to 328) | 804 (746 to 865) | 159.0% (141.1 to 177.1) | 410.4 (386.5 to 436.3) | 590.0 (550.9 to 632.6) | 1.14 (1.13 to 1.16) |
| **Samoa** | 441 (409 to 476) | 1235 (1144 to 1342) | 180.0% (164.7 to 194.8) | 383.0 (355.5 to 413.4) | 665.6 (618.0 to 725.6) | 1.77 (1.71 to 1.84) |
| **Sao Tome and Principe** | 105 (96 to 116) | 435 (395 to 481) | 312.9% (288.6 to 343.2) | 140.5 (126.8 to 154.7) | 262.8 (237.8 to 289.5) | 2.05 (2.04 to 2.06) |
| **Saudi Arabia** | 26189 (23955 to 28314) | 216942 (196534 to 242083) | 728.4% (661.5 to 797.1) | 274.3 (252.6 to 296.9) | 526.3 (482.8 to 573.6) | 2.05 (2.00 to 2.10) |
| **Senegal** | 8261 (7513 to 9044) | 38284 (35645 to 41046) | 363.4% (334.7 to 394.2) | 198.6 (179.2 to 217.3) | 361.5 (336.0 to 391.4) | 2.09 (1.97 to 2.21) |
| **Serbia** | 26553 (23839 to 29618) | 42593 (38240 to 47152) | 60.4% (45.1 to 79.3) | 218.6 (198.2 to 242.3) | 339.9 (307.9 to 380.3) | 1.41 (1.32 to 1.50) |
| **Seychelles** | 121 (111 to 133) | 752 (692 to 816) | 519.8% (474.2 to 572.3) | 206.0 (187.0 to 227.1) | 586.5 (543.5 to 633.6) | 3.45 (3.28 to 3.62) |
| **Sierra Leone** | 3497 (3194 to 3830) | 14294 (12991 to 15592) | 308.7% (284.8 to 334.2) | 139.1 (126.2 to 152.1) | 248.2 (227.3 to 271.1) | 1.94 (1.90 to 1.98) |
| **Singapore** | 8724 (8050 to 9447) | 28829 (26065 to 31511) | 230.5% (203.5 to 259.4) | 280.8 (259.6 to 304.8) | 365.1 (333.6 to 395.0) | 0.66 (0.59 to 0.73) |
| **Slovakia** | 9190 (8266 to 10142) | 16720 (14857 to 18311) | 81.9% (67.5 to 96.4) | 158.4 (142.6 to 175.1) | 214.5 (192.2 to 234.4) | 0.97 (0.91 to 1.03) |
| **Slovenia** | 4354 (3921 to 4869) | 6814 (6074 to 7538) | 56.5% (43.7 to 69.9) | 180.4 (163.0 to 201.2) | 218.0 (196.1 to 241.8) | 0.49 (0.35 to 0.64) |
| **Solomon Islands** | 508 (475 to 547) | 2522 (2368 to 2694) | 396.1% (369.2 to 426.3) | 252.8 (238.6 to 268.4) | 471.3 (449.1 to 495.2) | 2.09 (2.01 to 2.17) |
| **Somalia** | 4224 (3899 to 4585) | 18967 (17336 to 20666) | 349.0% (324.5 to 377.4) | 133.4 (124.2 to 144.2) | 184.4 (171.1 to 197.8) | 1.06 (1.04 to 1.08) |
| **South Africa** | 47440 (43331 to 52086) | 181422 (166529 to 198202) | 282.4% (262.8 to 302.6) | 193.6 (176.3 to 213.1) | 338.8 (311.4 to 368.9) | 1.95 (1.90 to 2.01) |
| **South Korea** | 88328 (81577 to 95166) | 345554 (323370 to 369348) | 291.2% (266.2 to 320.7) | 211.6 (195.6 to 228.4) | 462.3 (437.1 to 489.4) | 2.67 (2.56 to 2.78) |
| **South Sudan** | 3580 (3317 to 3892) | 8929 (8191 to 9698) | 149.4% (134.1 to 166.7) | 118.8 (110.5 to 128.8) | 167.6 (155.7 to 180.1) | 1.10 (1.08 to 1.11) |
| **Spain** | 100425 (91508 to 110419) | 205301 (185675 to 223612) | 104.4% (86.1 to 118.2) | 211.6 (196.2 to 229.5) | 319.1 (294.1 to 342.6) | 1.20 (1.05 to 1.35) |
| **Sri Lanka** | 35915 (32775 to 39869) | 157995 (145804 to 170674) | 339.9% (310.9 to 367.5) | 285.1 (259.8 to 316.4) | 575.1 (533.6 to 621.8) | 2.30 (2.19 to 2.41) |
| **Sudan** | 21233 (19468 to 23281) | 116952 (106798 to 127052) | 450.8% (417.9 to 489.2) | 174.7 (158.9 to 191.8) | 379.4 (345.8 to 412.5) | 2.52 (2.47 to 2.56) |
| **Suriname** | 898 (822 to 982) | 3611 (3326 to 3916) | 302.2% (274.0 to 329.6) | 288.4 (263.9 to 314.2) | 553.8 (515.2 to 597.1) | 2.25 (2.18 to 2.31) |
| **Swaziland** | 792 (735 to 860) | 2998 (2802 to 3209) | 278.5% (256.9 to 299.5) | 225.7 (210.8 to 243.0) | 408.3 (383.5 to 433.5) | 2.08 (1.97 to 2.19) |
| **Sweden** | 15436 (13691 to 17348) | 29634 (25942 to 33600) | 92.0% (79.9 to 104.8) | 140.8 (124.3 to 159.0) | 223.6 (198.1 to 252.5) | 1.55 (1.50 to 1.59) |
| **Switzerland** | 14957 (13639 to 16419) | 37652 (33683 to 41203) | 151.7% (131.6 to 178.3) | 173.9 (158.3 to 191.6) | 312.1 (283.0 to 339.5) | 1.75 (1.69 to 1.81) |
| **Syria** | 15030 (13710 to 16401) | 66015 (59725 to 72410) | 339.2% (308.0 to 374.2) | 207.5 (188.3 to 226.9) | 416.0 (380.1 to 453.3) | 2.25 (2.20 to 2.29) |
| **Taiwan (Province of China)** | 34551 (32752 to 36613) | 102811 (95783 to 111383) | 197.6% (178.8 to 215.3) | 183.2 (174.0 to 193.6) | 291.0 (272.8 to 312.4) | 1.35 (1.29 to 1.42) |
| **Tajikistan** | 3042 (2779 to 3309) | 17209 (15702 to 18796) | 465.6% (424.3 to 504.7) | 85.5 (78.1 to 93.1) | 196.8 (180.2 to 215.6) | 2.84 (2.75 to 2.94) |
| **Tanzania** | 12990 (12071 to 13898) | 53514 (49815 to 57368) | 312.0% (290.3 to 335.1) | 105.7 (99.5 to 112.1) | 162.3 (151.7 to 172.6) | 1.42 (1.38 to 1.46) |
| **Thailand** | 70114 (64239 to 76552) | 309126 (276640 to 345263) | 340.9% (299.7 to 386.4) | 160.2 (146.9 to 175.5) | 304.4 (275.1 to 336.5) | 2.02 (1.97 to 2.06) |
| **The Bahamas** | 567 (526 to 611) | 2086 (1903 to 2264) | 267.8% (240.9 to 294.2) | 282.8 (263.0 to 303.7) | 455.6 (417.9 to 493.6) | 1.54 (1.50 to 1.57) |
| **The Gambia** | 679 (616 to 740) | 3832 (3515 to 4188) | 464.5% (430.9 to 497.3) | 138.3 (125.8 to 151.4) | 260.7 (238.8 to 286.0) | 2.14 (2.10 to 2.19) |
| **Timor-Leste** | 473 (437 to 517) | 3375 (3047 to 3690) | 612.8% (559.6 to 665.3) | 119.1 (110.1 to 129.1) | 337.7 (303.1 to 369.8) | 3.51 (3.49 to 3.54) |
| **Togo** | 1983 (1803 to 2158) | 10525 (9742 to 11324) | 430.7% (401.4 to 468.8) | 115.5 (106.8 to 125.5) | 188.4 (173.9 to 202.5) | 1.60 (1.58 to 1.61) |
| **Tokelau** | 6 (5 to 6) | 10 (9 to 11) | 73.4% (63.2 to 84.3) | 435.4 (401.9 to 471.1) | 737.8 (685.5 to 788.9) | 1.63 (1.55 to 1.71) |
| **Tonga** | 257 (239 to 275) | 574 (537 to 620) | 123.6% (112.2 to 136.4) | 373.8 (348.6 to 399.3) | 625.6 (585.3 to 673.3) | 1.64 (1.62 to 1.66) |
| **Trinidad and Tobago** | 4611 (4372 to 4852) | 11520 (10724 to 12293) | 149.8% (131.3 to 168.4) | 486.7 (462.9 to 512.3) | 658.1 (614.1 to 700.0) | 0.93 (0.88 to 0.97) |
| **Tunisia** | 11458 (10372 to 12547) | 65600 (58872 to 72279) | 472.5% (431.5 to 510.2) | 186.8 (168.9 to 205.0) | 471.8 (426.6 to 518.9) | 2.87 (2.67 to 3.07) |
| **Turkey** | 67635 (63253 to 72342) | 326218 (300295 to 358620) | 382.3% (347.2 to 423.1) | 164.1 (153.7 to 175.9) | 333.2 (307.9 to 364.9) | 2.38 (2.19 to 2.56) |
| **Turkmenistan** | 2044 (1885 to 2210) | 9146 (8365 to 10080) | 347.4% (314.8 to 381.0) | 79.8 (73.6 to 86.4) | 177.6 (162.2 to 195.7) | 2.60 (2.51 to 2.69) |
| **Tuvalu** | 24 (22 to 26) | 63 (59 to 67) | 162.5% (148.5 to 177.1) | 306.3 (286.9 to 326.3) | 528.2 (496.8 to 561.5) | 1.71 (1.64 to 1.78) |
| **Uganda** | 9214 (8543 to 9920) | 36796 (33859 to 39633) | 299.3% (275.2 to 322.9) | 121.5 (113.1 to 130.8) | 175.1 (162.6 to 187.7) | 1.16 (1.13 to 1.20) |
| **Ukraine** | 59542 (52302 to 67148) | 100453 (87651 to 113785) | 68.7% (58.2 to 80.1) | 92.7 (82.0 to 103.4) | 169.7 (148.9 to 191.1) | 1.84 (1.76 to 1.91) |
| **United Arab Emirates** | 3052 (2722 to 3436) | 64755 (56881 to 73716) | 2021.5% (1825.4 to 2286.6) | 312.5 (292.9 to 333.1) | 488.8 (449.3 to 532.1) | 1.27 (1.19 to 1.36) |
| **United Kingdom** | 97863 (87456 to 108797) | 253037 (231405 to 276871) | 158.6% (148.8 to 168.9) | 139.9 (125.2 to 155.6) | 313.5 (287.7 to 341.0) | 2.48 (2.35 to 2.60) |
| **United States** | 500824 (457268 to 547177) | 1989165 (1856370 to 2117191) | 297.2% (273.6 to 321.6) | 185.5 (168.0 to 203.8) | 445.5 (416.8 to 472.7) | 3.02 (2.90 to 3.13) |
| **Uruguay** | 4413 (4044 to 4853) | 12048 (10861 to 13289) | 173.0% (153.5 to 194.2) | 127.1 (116.6 to 139.5) | 287.4 (258.8 to 315.3) | 2.84 (2.77 to 2.90) |
| **Uzbekistan** | 14198 (13109 to 15620) | 96296 (88031 to 106182) | 578.2% (523.9 to 644.1) | 97.4 (90.2 to 106.9) | 275.3 (252.7 to 303.3) | 3.72 (3.50 to 3.94) |
| **Vanuatu** | 246 (227 to 270) | 1324 (1232 to 1419) | 437.4% (405.5 to 469.1) | 255.5 (236.8 to 277.0) | 497.4 (464.9 to 534.2) | 2.12 (2.10 to 2.14) |
| **Venezuela** | 26765 (24807 to 28841) | 100542 (91973 to 110151) | 275.6% (246.1 to 305.6) | 203.7 (188.8 to 220.5) | 329.1 (302.0 to 359.1) | 1.61 (1.56 to 1.67) |
| **Vietnam** | 66901 (61676 to 73236) | 303192 (281778 to 327272) | 353.2% (325.4 to 383.5) | 149.8 (138.1 to 162.6) | 278.8 (259.2 to 302.0) | 2.09 (2.02 to 2.16) |
| **Virgin Islands, U.S.** | 367 (332 to 402) | 740 (678 to 806) | 101.4% (85.3 to 119.4) | 350.7 (320.8 to 380.4) | 626.4 (576.0 to 674.3) | 1.95 (1.87 to 2.02) |
| **Yemen** | 9873 (8921 to 10869) | 64410 (58606 to 71323) | 552.4% (511.8 to 595.6) | 140.4 (127.2 to 154.7) | 287.6 (262.0 to 319.0) | 2.41 (2.36 to 2.47) |
| **Zambia** | 6221 (5720 to 6727) | 27696 (25463 to 30173) | 345.2% (317.6 to 373.8) | 171.9 (159.3 to 186.5) | 244.9 (226.4 to 265.3) | 1.12 (1.10 to 1.14) |
| **Zimbabwe** | 7108 (6507 to 7704) | 23775 (21995 to 25727) | 234.5% (214.9 to 260.0) | 142.7 (132.5 to 155.4) | 249.0 (230.8 to 268.2) | 1.98 (1.93 to 2.03) |

ASIR, age-standardized incidence rate; UI, uncertainty interval; EAPC, estimated annual percentage change; CI, confidence interval.

# Supplementary Table 7. The prevalence cases and age-standardized prevalence rate of type 2 diabetes mellitus in 1990 and 2021, and its temporal trends from 1990 to 2021 in 204 countries/territories.

| **Countries/territories** | **Number of prevalence cases in 1990**  **(95%UI)** | **Number of prevalence cases in 2021**  **(95%UI)** | **Percentage change in prevalence counts, 1990-2021**  **(95%UI)** | **1990 ASPR per 100000**  **(95%UI)** | **2021 ASPR per 100000**  **(95%UI)** | **1990-2021 EAPC (%)**  **(95%CI)** |
| --- | --- | --- | --- | --- | --- | --- |
| **Afghanistan** | 369304 (332550 to 409622) | 2058223 (1888947 to 2234322) | 457.3% (414.4 to 503.2) | 5163.5 (4677.2 to 5690.1) | 14383.1 (13324.0 to 15520.0) | 3.41 (3.36 to 3.45) |
| **Albania** | 49555 (44542 to 54968) | 138139 (122531 to 155781) | 178.8% (160.5 to 199.4) | 2228.4 (2004.4 to 2471.8) | 3348.4 (2987.5 to 3779.0) | 1.34 (1.26 to 1.42) |
| **Algeria** | 526467 (474101 to 582049) | 3892013 (3591808 to 4171457) | 639.3% (583.5 to 709.2) | 3806.1 (3425.3 to 4203.1) | 9805.5 (9057.7 to 10519.5) | 3.10 (3.08 to 3.12) |
| **American Samoa** | 2665 (2446 to 2930) | 10805 (9983 to 11677) | 305.5% (279.6 to 331.5) | 8930.5 (8260.0 to 9756.7) | 21151.9 (19513.2 to 22776.8) | 2.65 (2.47 to 2.82) |
| **Andorra** | 1259 (1125 to 1400) | 5965 (5369 to 6577) | 373.7% (344.5 to 408.2) | 2059.5 (1843.8 to 2287.1) | 4319.4 (3904.0 to 4783.2) | 2.44 (2.40 to 2.48) |
| **Angola** | 156958 (142953 to 174230) | 1008288 (911815 to 1115010) | 542.4% (497.6 to 587.7) | 3129.4 (2863.9 to 3446.9) | 6025.3 (5499.4 to 6570.4) | 2.24 (2.17 to 2.30) |
| **Antigua and Barbuda** | 2983 (2742 to 3274) | 11092 (10139 to 12087) | 271.9% (244.0 to 300.7) | 5737.8 (5238.9 to 6350.8) | 10031.0 (9216.2 to 10927.1) | 1.77 (1.74 to 1.80) |
| **Argentina** | 852138 (793805 to 918409) | 2827812 (2566532 to 3113393) | 231.8% (202.8 to 257.5) | 2616.5 (2437.8 to 2818.4) | 5220.7 (4733.4 to 5747.2) | 2.19 (2.12 to 2.26) |
| **Armenia** | 80421 (73578 to 88975) | 186502 (168358 to 206619) | 131.9% (114.5 to 149.1) | 2737.8 (2513.8 to 3025.8) | 4514.7 (4082.7 to 5017.3) | 1.46 (1.24 to 1.69) |
| **Australia** | 324288 (294255 to 357815) | 1303062 (1160529 to 1422417) | 301.8% (276.1 to 334.0) | 1676.1 (1524.1 to 1853.7) | 3201.5 (2844.6 to 3506.3) | 2.13 (2.08 to 2.17) |
| **Austria** | 127082 (116912 to 139236) | 389115 (353335 to 424068) | 206.2% (188.3 to 227.5) | 1168.0 (1064.8 to 1285.9) | 2598.0 (2350.5 to 2834.7) | 2.57 (2.49 to 2.65) |
| **Azerbaijan** | 102522 (93451 to 112808) | 521623 (476193 to 570163) | 408.8% (371.2 to 449.8) | 1860.6 (1697.6 to 2046.1) | 4539.3 (4166.6 to 4952.8) | 2.98 (2.84 to 3.11) |
| **Bahrain** | 15526 (14018 to 17045) | 189233 (175437 to 205462) | 1118.8% (1034.7 to 1210.6) | 6639.2 (6058.8 to 7185.4) | 14706.1 (13797.7 to 15746.0) | 2.57 (2.43 to 2.70) |
| **Bangladesh** | 1738561 (1582662 to 1904260) | 10466962 (9766057 to 11264945) | 502.0% (467.7 to 539.3) | 2910.2 (2677.2 to 3151.6) | 6827.5 (6391.9 to 7338.1) | 2.87 (2.80 to 2.94) |
| **Barbados** | 15356 (14203 to 16683) | 44212 (40984 to 47442) | 187.9% (171.0 to 209.9) | 5591.7 (5117.9 to 6118.6) | 9385.9 (8653.3 to 10097.0) | 1.49 (1.41 to 1.56) |
| **Belarus** | 185790 (168219 to 207024) | 372312 (333790 to 406802) | 100.4% (81.3 to 116.4) | 1475.7 (1337.5 to 1642.9) | 2519.7 (2273.1 to 2755.6) | 1.52 (1.43 to 1.62) |
| **Belgium** | 306521 (279514 to 336817) | 820484 (727114 to 916875) | 167.7% (146.3 to 193.4) | 2226.4 (2022.2 to 2445.1) | 4495.7 (3994.6 to 4995.1) | 2.21 (2.16 to 2.27) |
| **Belize** | 4143 (3806 to 4529) | 28281 (25881 to 31365) | 582.7% (526.4 to 647.6) | 4102.2 (3765.7 to 4484.0) | 8201.1 (7513.5 to 9118.0) | 2.29 (2.23 to 2.36) |
| **Benin** | 66759 (60386 to 73249) | 459466 (421648 to 500582) | 588.2% (548.5 to 637.4) | 2802.6 (2530.5 to 3070.1) | 6408.7 (5865.7 to 6974.4) | 2.57 (2.44 to 2.70) |
| **Bermuda** | 2048 (1866 to 2247) | 6721 (6105 to 7367) | 228.1% (202.3 to 252.9) | 3166.8 (2884.9 to 3472.2) | 5865.6 (5340.2 to 6439.3) | 1.89 (1.84 to 1.95) |
| **Bhutan** | 8648 (7750 to 9588) | 34562 (31630 to 37273) | 299.6% (275.4 to 326.1) | 2625.5 (2368.9 to 2866.2) | 4998.8 (4586.2 to 5379.0) | 2.12 (2.06 to 2.19) |
| **Bolivia** | 98472 (90031 to 107266) | 582759 (531497 to 637603) | 491.8% (458.4 to 526.6) | 2750.1 (2507.8 to 2991.6) | 5936.8 (5396.5 to 6498.2) | 2.53 (2.48 to 2.58) |
| **Bosnia and Herzegovina** | 130629 (118446 to 143846) | 389627 (357411 to 420115) | 198.3% (175.0 to 219.9) | 3034.9 (2763.1 to 3330.2) | 6563.4 (6036.9 to 7077.5) | 2.75 (2.59 to 2.92) |
| **Botswana** | 13599 (12122 to 15147) | 79630 (72957 to 86817) | 485.6% (445.9 to 531.8) | 2166.7 (1961.7 to 2396.6) | 4629.0 (4250.3 to 4988.3) | 2.55 (2.53 to 2.58) |
| **Brazil** | 3727747 (3354878 to 4127699) | 12991915 (11658615 to 14490501) | 248.5% (236.5 to 262.5) | 3759.5 (3379.2 to 4171.9) | 5095.8 (4578.5 to 5678.2) | 1.04 (0.98 to 1.10) |
| **Brunei** | 6761 (6214 to 7352) | 57528 (53495 to 61758) | 750.9% (693.9 to 812.5) | 5504.2 (5094.9 to 5927.0) | 13428.2 (12516.5 to 14340.1) | 2.62 (2.52 to 2.72) |
| **Bulgaria** | 360871 (326120 to 401566) | 673546 (615185 to 724076) | 86.6% (71.0 to 106.5) | 2929.8 (2657.6 to 3245.2) | 5237.9 (4810.7 to 5614.0) | 1.96 (1.80 to 2.11) |
| **Burkina Faso** | 87465 (79298 to 95780) | 504098 (458182 to 549810) | 476.3% (442.0 to 513.7) | 1821.7 (1661.8 to 1988.3) | 4242.6 (3887.3 to 4576.5) | 2.79 (2.69 to 2.89) |
| **Burundi** | 42541 (38437 to 47146) | 173263 (156475 to 189310) | 307.3% (279.4 to 333.8) | 1595.8 (1463.0 to 1752.1) | 2709.1 (2467.3 to 2935.3) | 1.64 (1.61 to 1.67) |
| **Cambodia** | 89986 (81413 to 99027) | 644874 (591624 to 701597) | 616.6% (562.7 to 673.7) | 1781.2 (1624.5 to 1948.2) | 4890.4 (4487.2 to 5328.6) | 3.31 (3.29 to 3.33) |
| **Cameroon** | 119398 (107007 to 130696) | 839526 (766209 to 917448) | 603.1% (554.4 to 649.9) | 2252.0 (2023.7 to 2457.0) | 4857.8 (4489.8 to 5264.1) | 2.57 (2.47 to 2.68) |
| **Canada** | 519021 (470791 to 583909) | 3602183 (3201441 to 4004146) | 594.0% (525.7 to 675.2) | 1598.6 (1447.1 to 1804.2) | 5429.0 (4814.9 to 6038.4) | 3.67 (3.59 to 3.76) |
| **Cape Verde** | 6561 (5971 to 7153) | 34160 (31288 to 37045) | 420.7% (390.9 to 454.1) | 2774.4 (2532.7 to 3031.3) | 6813.4 (6271.2 to 7393.1) | 3.14 (2.99 to 3.29) |
| **Central African Republic** | 49778 (44799 to 54823) | 220553 (200395 to 240753) | 343.1% (317.6 to 369.5) | 3491.3 (3165.9 to 3823.5) | 6896.7 (6300.0 to 7480.0) | 2.28 (2.21 to 2.35) |
| **Chad** | 72264 (65293 to 79492) | 375720 (342547 to 409784) | 419.9% (389.2 to 452.2) | 2242.3 (2038.8 to 2450.7) | 4739.7 (4323.1 to 5156.6) | 2.38 (2.22 to 2.54) |
| **Chile** | 286581 (259670 to 317532) | 1613636 (1464745 to 1783647) | 463.1% (420.0 to 509.1) | 2733.3 (2474.7 to 3028.9) | 6417.8 (5837.4 to 7073.0) | 2.84 (2.71 to 2.97) |
| **China** | 34625427 (31011339 to 38301027) | 115845779 (106137170 to 126653487) | 234.6% (219.4 to 254.9) | 3519.8 (3138.3 to 3910.1) | 6055.5 (5510.1 to 6614.3) | 1.65 (1.56 to 1.75) |
| **Colombia** | 869191 (795481 to 961864) | 3302788 (3068862 to 3570593) | 280.0% (253.0 to 309.8) | 4110.3 (3765.0 to 4568.9) | 6004.2 (5579.7 to 6488.6) | 0.89 (0.76 to 1.02) |
| **Comoros** | 4717 (4280 to 5185) | 23315 (21532 to 25327) | 394.2% (363.0 to 432.3) | 2136.9 (1951.7 to 2344.3) | 4177.8 (3856.5 to 4539.2) | 2.23 (2.20 to 2.26) |
| **Congo** | 32454 (29044 to 35684) | 195317 (177084 to 213978) | 501.8% (454.9 to 546.5) | 2598.5 (2348.4 to 2849.5) | 5511.5 (5057.7 to 5989.3) | 2.48 (2.44 to 2.52) |
| **Cook Islands** | 1402 (1295 to 1516) | 4389 (4049 to 4692) | 213.1% (193.2 to 235.7) | 9817.8 (9061.3 to 10613.4) | 19105.9 (17668.9 to 20470.4) | 1.92 (1.76 to 2.07) |
| **Costa Rica** | 81375 (73845 to 88606) | 406741 (375949 to 444396) | 399.8% (369.7 to 432.4) | 4049.2 (3683.5 to 4420.7) | 7444.2 (6886.7 to 8128.1) | 1.93 (1.85 to 2.02) |
| **Cote d'Ivoire** | 142809 (128089 to 157133) | 832653 (756598 to 902930) | 483.1% (450.2 to 523.8) | 2584.1 (2324.3 to 2814.5) | 5351.9 (4901.2 to 5801.9) | 2.39 (2.33 to 2.46) |
| **Croatia** | 187263 (168221 to 206694) | 380447 (343530 to 420042) | 103.2% (87.7 to 117.3) | 3032.9 (2727.6 to 3352.9) | 4753.4 (4288.5 to 5273.1) | 1.44 (1.32 to 1.55) |
| **Cuba** | 369818 (342325 to 405781) | 1050177 (957964 to 1144176) | 184.0% (162.9 to 204.4) | 3514.4 (3253.2 to 3863.7) | 6033.1 (5495.7 to 6550.5) | 1.61 (1.54 to 1.67) |
| **Cyprus** | 36563 (34119 to 39115) | 108041 (99128 to 118376) | 195.5% (177.4 to 214.4) | 4277.2 (3987.9 to 4579.6) | 5416.6 (4968.1 to 5947.2) | 0.65 (0.55 to 0.75) |
| **Czech Republic** | 397024 (359521 to 441911) | 1001748 (907807 to 1088882) | 152.3% (135.4 to 171.7) | 2958.7 (2682.4 to 3293.0) | 5151.1 (4645.0 to 5610.9) | 1.90 (1.87 to 1.94) |
| **Democratic Republic of the Congo** | 447437 (403661 to 495430) | 2302575 (2092791 to 2517222) | 414.6% (384.1 to 448.7) | 2398.0 (2184.1 to 2633.3) | 4600.9 (4203.8 to 4996.3) | 2.08 (2.04 to 2.12) |
| **Denmark** | 104831 (96658 to 113576) | 334266 (302071 to 370014) | 218.9% (197.8 to 244.2) | 1482.9 (1353.4 to 1616.1) | 3513.1 (3164.7 to 3904.5) | 2.80 (2.77 to 2.83) |
| **Djibouti** | 2326 (2010 to 2628) | 22777 (20678 to 24971) | 879.4% (810.8 to 957.5) | 1374.0 (1234.9 to 1522.2) | 2852.9 (2609.0 to 3100.5) | 2.42 (2.40 to 2.44) |
| **Dominica** | 3519 (3263 to 3843) | 8861 (8070 to 9672) | 151.8% (134.9 to 167.8) | 5992.2 (5558.2 to 6585.4) | 10674.3 (9774.7 to 11658.5) | 1.80 (1.75 to 1.85) |
| **Dominican Republic** | 166306 (151949 to 183050) | 913460 (832778 to 998474) | 449.3% (408.4 to 489.1) | 3841.6 (3516.5 to 4236.6) | 8696.9 (7932.2 to 9495.1) | 2.74 (2.69 to 2.80) |
| **Ecuador** | 179032 (163900 to 196395) | 1213279 (1123790 to 1304282) | 577.7% (533.4 to 628.3) | 3025.0 (2768.3 to 3335.2) | 7190.8 (6650.4 to 7740.2) | 2.84 (2.73 to 2.95) |
| **Egypt** | 624413 (565866 to 689714) | 6092763 (5534595 to 6721537) | 875.8% (811.1 to 949.4) | 1982.2 (1813.5 to 2167.1) | 8139.0 (7408.4 to 8941.9) | 4.85 (4.75 to 4.95) |
| **El Salvador** | 103938 (94582 to 115252) | 401689 (367198 to 441254) | 286.5% (262.8 to 314.2) | 3105.6 (2825.5 to 3446.7) | 6532.8 (5961.5 to 7182.8) | 2.26 (2.16 to 2.36) |
| **Equatorial Guinea** | 6197 (5614 to 6793) | 47595 (43346 to 52232) | 668.0% (625.0 to 712.9) | 2746.2 (2502.3 to 3006.8) | 6209.1 (5659.0 to 6725.6) | 2.82 (2.76 to 2.89) |
| **Eritrea** | 27193 (24061 to 30499) | 146045 (132363 to 161375) | 437.1% (405.7 to 478.3) | 1905.9 (1727.9 to 2092.2) | 3824.2 (3494.1 to 4143.0) | 2.29 (2.26 to 2.32) |
| **Estonia** | 36960 (33609 to 41039) | 88175 (79291 to 97181) | 138.6% (120.8 to 155.9) | 1869.4 (1698.8 to 2078.4) | 3983.7 (3575.6 to 4396.4) | 2.43 (2.34 to 2.52) |
| **Ethiopia** | 533664 (474221 to 593237) | 1775220 (1582274 to 1963889) | 232.6% (218.7 to 247.5) | 2261.3 (2016.8 to 2498.7) | 3132.2 (2813.4 to 3463.6) | 0.87 (0.82 to 0.93) |
| **Federated States of Micronesia** | 3599 (3306 to 3916) | 12361 (11443 to 13335) | 243.4% (224.0 to 266.1) | 6073.8 (5631.5 to 6537.5) | 13942.6 (12986.4 to 14986.7) | 2.87 (2.66 to 3.07) |
| **Fiji** | 33978 (31085 to 37157) | 136901 (126402 to 146404) | 302.9% (271.8 to 337.0) | 7420.4 (6851.0 to 8017.4) | 15952.9 (14836.6 to 16972.9) | 2.29 (2.20 to 2.38) |
| **Finland** | 186299 (169675 to 205895) | 517093 (470099 to 569380) | 177.6% (159.0 to 198.8) | 2810.4 (2557.3 to 3102.1) | 5528.3 (4966.3 to 6078.8) | 2.14 (2.05 to 2.24) |
| **France** | 1242470 (1149016 to 1338789) | 3405205 (3054161 to 3750608) | 174.1% (155.6 to 192.7) | 1630.0 (1500.0 to 1762.9) | 3167.3 (2844.2 to 3518.1) | 2.35 (2.28 to 2.42) |
| **Gabon** | 19120 (17416 to 20930) | 83415 (76812 to 91274) | 336.3% (310.6 to 367.7) | 3083.8 (2824.4 to 3375.6) | 6591.5 (6105.6 to 7121.8) | 2.51 (2.43 to 2.59) |
| **Georgia** | 133450 (120941 to 146671) | 282347 (259899 to 309507) | 111.6% (94.4 to 128.0) | 2156.3 (1959.9 to 2364.7) | 5388.4 (4962.5 to 5897.6) | 3.12 (2.99 to 3.25) |
| **Germany** | 1883616 (1728198 to 2050391) | 6420799 (5843383 to 7146057) | 240.9% (216.4 to 268.8) | 1631.3 (1492.6 to 1782.2) | 4261.0 (3860.5 to 4722.6) | 3.08 (2.91 to 3.26) |
| **Ghana** | 189542 (171213 to 209482) | 1126967 (1017344 to 1222856) | 494.6% (459.6 to 529.7) | 2416.3 (2190.2 to 2636.6) | 5174.1 (4728.8 to 5615.5) | 2.50 (2.37 to 2.64) |
| **Greece** | 380057 (346368 to 417875) | 828917 (748714 to 909492) | 118.1% (103.3 to 133.6) | 2718.4 (2481.4 to 3003.8) | 4828.5 (4329.2 to 5331.1) | 1.77 (1.71 to 1.83) |
| **Greenland** | 243 (207 to 281) | 2481 (2234 to 2730) | 922.9% (813.1 to 1063.8) | 683.5 (595.3 to 773.6) | 3435.2 (3109.6 to 3779.2) | 5.48 (5.33 to 5.63) |
| **Grenada** | 4191 (3865 to 4529) | 13156 (11961 to 14389) | 213.9% (191.9 to 234.3) | 6177.8 (5663.0 to 6715.1) | 11086.5 (10097.9 to 12082.7) | 1.84 (1.79 to 1.89) |
| **Guam** | 4649 (4229 to 5074) | 16376 (14963 to 17936) | 252.2% (228.1 to 282.7) | 4582.5 (4221.7 to 4996.9) | 8500.5 (7762.1 to 9278.0) | 1.88 (1.83 to 1.94) |
| **Guatemala** | 146429 (134869 to 158718) | 1100980 (1016847 to 1191172) | 651.9% (600.7 to 707.3) | 3427.6 (3153.4 to 3697.2) | 8967.7 (8301.4 to 9715.7) | 3.35 (3.13 to 3.57) |
| **Guinea** | 74881 (67879 to 81903) | 305678 (280103 to 331188) | 308.2% (282.4 to 335.7) | 2065.7 (1876.6 to 2268.5) | 4241.9 (3906.5 to 4601.4) | 2.19 (2.12 to 2.26) |
| **Guinea-Bissau** | 14181 (12739 to 15645) | 61090 (56074 to 66760) | 330.8% (307.2 to 359.7) | 2853.1 (2572.5 to 3120.8) | 5767.9 (5317.7 to 6274.1) | 2.28 (2.23 to 2.34) |
| **Guyana** | 37380 (33826 to 41317) | 107783 (99819 to 116707) | 188.3% (169.2 to 209.8) | 8385.4 (7625.9 to 9255.1) | 15148.0 (14073.0 to 16382.4) | 1.89 (1.82 to 1.96) |
| **Haiti** | 224820 (203818 to 245474) | 948297 (862452 to 1044772) | 321.8% (296.1 to 350.6) | 6024.4 (5524.9 to 6572.3) | 10589.9 (9674.6 to 11701.5) | 1.78 (1.75 to 1.80) |
| **Honduras** | 103678 (93742 to 113741) | 619199 (566655 to 679553) | 497.2% (464.4 to 534.9) | 4206.9 (3821.5 to 4600.1) | 8298.0 (7602.7 to 9122.0) | 2.26 (2.18 to 2.34) |
| **Hungary** | 457608 (412355 to 502291) | 863044 (778437 to 959122) | 88.6% (75.5 to 101.2) | 3198.8 (2887.3 to 3511.3) | 4927.9 (4433.5 to 5462.7) | 1.35 (1.12 to 1.59) |
| **Iceland** | 5110 (4592 to 5684) | 21068 (19271 to 23194) | 312.3% (284.5 to 339.9) | 1882.0 (1686.0 to 2099.1) | 4315.2 (3935.6 to 4744.9) | 2.71 (2.67 to 2.75) |
| **India** | 16915529 (15103782 to 18815277) | 75141589 (67534099 to 82889789) | 344.2% (331.3 to 358.2) | 2984.8 (2678.0 to 3307.7) | 5620.7 (5048.8 to 6195.5) | 2.01 (1.96 to 2.05) |
| **Indonesia** | 2453527 (2172274 to 2740530) | 11479437 (10328432 to 12838858) | 367.9% (345.7 to 390.5) | 2186.1 (1953.7 to 2436.5) | 4359.2 (3925.8 to 4821.9) | 1.71 (1.41 to 2.00) |
| **Iran** | 796136 (709722 to 888310) | 5702547 (5105371 to 6319369) | 616.3% (587.7 to 645.5) | 2693.7 (2411.4 to 3004.5) | 6684.4 (5989.8 to 7369.3) | 3.00 (2.94 to 3.06) |
| **Iraq** | 623420 (572791 to 676596) | 4510722 (4168875 to 4909146) | 623.5% (582.0 to 674.2) | 6678.1 (6130.0 to 7282.2) | 15052.7 (13975.5 to 16271.6) | 2.77 (2.68 to 2.85) |
| **Ireland** | 82219 (75050 to 90892) | 247580 (221018 to 273949) | 201.1% (178.6 to 225.5) | 2133.0 (1939.0 to 2366.7) | 3526.3 (3137.1 to 3900.1) | 1.75 (1.67 to 1.83) |
| **Israel** | 154416 (143556 to 167974) | 561681 (511422 to 611464) | 263.7% (234.0 to 286.6) | 3225.7 (2998.9 to 3515.9) | 4980.5 (4506.4 to 5427.9) | 1.42 (1.26 to 1.59) |
| **Italy** | 2263362 (2004130 to 2566486) | 4407680 (3850537 to 5050375) | 94.7% (85.4 to 106.6) | 2743.7 (2420.7 to 3113.3) | 3802.8 (3269.4 to 4331.6) | 1.06 (0.91 to 1.22) |
| **Jamaica** | 77101 (71317 to 82967) | 235955 (217481 to 255453) | 206.0% (186.4 to 228.6) | 4360.9 (4039.5 to 4718.0) | 7649.1 (7057.1 to 8282.1) | 1.65 (1.57 to 1.73) |
| **Japan** | 5845738 (5244697 to 6549792) | 14497687 (13093785 to 16119514) | 148.0% (137.0 to 157.7) | 3501.0 (3145.9 to 3915.1) | 5636.9 (5029.7 to 6212.3) | 1.22 (1.11 to 1.33) |
| **Jordan** | 103413 (95533 to 112125) | 1211267 (1117477 to 1308982) | 1071.3% (997.6 to 1147.6) | 6601.1 (6137.9 to 7108.9) | 13213.3 (12340.7 to 14178.3) | 2.36 (2.27 to 2.45) |
| **Kazakhstan** | 368657 (330789 to 427085) | 1170282 (1086768 to 1254330) | 217.4% (184.0 to 247.6) | 2689.9 (2414.3 to 3101.2) | 6071.2 (5632.5 to 6494.3) | 2.81 (2.73 to 2.89) |
| **Kenya** | 116850 (102889 to 130222) | 509748 (454635 to 559720) | 336.2% (321.3 to 352.1) | 1222.5 (1091.9 to 1350.5) | 1858.5 (1674.3 to 2051.8) | 1.27 (1.21 to 1.33) |
| **Kiribati** | 3324 (3049 to 3648) | 13053 (12010 to 14111) | 292.8% (269.9 to 316.4) | 7054.9 (6490.2 to 7677.8) | 14170.0 (13143.1 to 15242.8) | 2.26 (2.13 to 2.40) |
| **Kuwait** | 61928 (55845 to 68255) | 633142 (575509 to 693987) | 922.4% (838.3 to 1004.7) | 6967.0 (6395.3 to 7659.0) | 14772.5 (13567.1 to 16051.1) | 2.61 (2.56 to 2.67) |
| **Kyrgyzstan** | 57164 (51771 to 62376) | 218249 (197136 to 241898) | 281.8% (253.8 to 318.7) | 1773.7 (1616.2 to 1928.0) | 3877.8 (3520.8 to 4333.4) | 2.61 (2.49 to 2.73) |
| **Laos** | 54784 (49787 to 60334) | 304217 (279356 to 329150) | 455.3% (420.9 to 492.4) | 2427.8 (2202.7 to 2653.7) | 5950.6 (5471.4 to 6454.5) | 2.85 (2.81 to 2.88) |
| **Latvia** | 61018 (55609 to 66632) | 131501 (119134 to 145232) | 115.5% (99.5 to 132.9) | 1782.6 (1621.7 to 1947.0) | 4029.1 (3652.9 to 4442.4) | 2.69 (2.58 to 2.80) |
| **Lebanon** | 116030 (106681 to 127446) | 647936 (601074 to 696828) | 458.4% (426.1 to 495.0) | 4994.2 (4593.4 to 5479.0) | 10910.3 (10094.6 to 11738.7) | 2.65 (2.59 to 2.71) |
| **Lesotho** | 14541 (12982 to 16294) | 56158 (51485 to 61242) | 286.2% (256.3 to 318.7) | 1633.2 (1461.6 to 1821.9) | 4646.6 (4275.0 to 5048.6) | 3.67 (3.59 to 3.76) |
| **Liberia** | 34441 (31258 to 37716) | 174582 (157860 to 192967) | 406.9% (374.8 to 446.7) | 2554.6 (2345.3 to 2797.7) | 5638.0 (5144.9 to 6125.5) | 2.68 (2.61 to 2.75) |
| **Libya** | 82048 (74390 to 90652) | 659735 (601820 to 724071) | 704.1% (649.1 to 777.2) | 3764.8 (3406.9 to 4168.4) | 10370.1 (9501.8 to 11375.5) | 3.51 (3.36 to 3.67) |
| **Lithuania** | 70075 (62981 to 77838) | 159731 (145704 to 174966) | 127.9% (110.0 to 146.5) | 1597.9 (1435.9 to 1778.6) | 3376.2 (3081.1 to 3686.9) | 2.35 (2.26 to 2.43) |
| **Luxembourg** | 11288 (10270 to 12346) | 39964 (36227 to 43963) | 254.0% (229.2 to 275.7) | 2210.7 (2003.6 to 2418.9) | 4254.1 (3858.6 to 4698.7) | 2.11 (2.07 to 2.16) |
| **Macedonia** | 70183 (63445 to 77556) | 222321 (199030 to 242510) | 216.8% (195.2 to 240.4) | 3630.7 (3288.6 to 3994.4) | 6574.1 (5890.7 to 7172.0) | 1.99 (1.85 to 2.13) |
| **Madagascar** | 81523 (73169 to 90797) | 379425 (343883 to 418812) | 365.4% (335.2 to 397.8) | 1411.7 (1284.1 to 1553.6) | 2561.9 (2343.9 to 2797.7) | 1.90 (1.88 to 1.92) |
| **Malawi** | 49025 (43549 to 54916) | 171252 (155280 to 187645) | 249.3% (224.4 to 275.9) | 1184.7 (1079.7 to 1299.4) | 1883.1 (1723.3 to 2041.6) | 1.37 (1.31 to 1.44) |
| **Malaysia** | 416537 (384463 to 455829) | 2137115 (1987044 to 2309670) | 413.1% (380.9 to 445.1) | 4006.0 (3696.1 to 4379.0) | 7199.8 (6693.7 to 7742.6) | 1.86 (1.81 to 1.90) |
| **Maldives** | 2638 (2395 to 2925) | 21155 (19288 to 23436) | 702.0% (645.0 to 777.6) | 2678.1 (2434.4 to 2955.6) | 5252.7 (4781.0 to 5767.0) | 2.16 (2.11 to 2.21) |
| **Mali** | 195478 (177952 to 214438) | 1021548 (929191 to 1117226) | 422.6% (392.1 to 455.0) | 4182.3 (3814.9 to 4576.1) | 8614.8 (7885.4 to 9398.3) | 2.46 (2.32 to 2.60) |
| **Malta** | 9393 (8563 to 10369) | 47545 (42734 to 52210) | 406.2% (368.5 to 442.9) | 2202.2 (2003.0 to 2435.8) | 6098.0 (5488.3 to 6750.7) | 3.35 (3.09 to 3.61) |
| **Marshall Islands** | 2260 (2059 to 2483) | 10389 (9558 to 11396) | 359.6% (330.7 to 394.7) | 10056.7 (9233.0 to 10920.7) | 21942.7 (20398.2 to 23914.2) | 2.61 (2.50 to 2.72) |
| **Mauritania** | 22029 (20057 to 24265) | 84590 (78864 to 90257) | 284.0% (260.3 to 306.5) | 1930.6 (1761.2 to 2108.7) | 3278.9 (3065.1 to 3466.4) | 1.58 (1.44 to 1.73) |
| **Mauritius** | 39460 (36716 to 42916) | 196803 (182183 to 211340) | 398.7% (365.6 to 432.8) | 5045.5 (4707.8 to 5453.8) | 10869.3 (10114.0 to 11631.1) | 2.55 (2.42 to 2.68) |
| **Mexico** | 3725611 (3380569 to 4096794) | 11879654 (10808425 to 13056051) | 218.9% (210.7 to 227.8) | 7356.3 (6675.9 to 8106.4) | 8862.0 (8061.5 to 9732.4) | 0.43 (0.35 to 0.50) |
| **Moldova** | 107361 (97944 to 119453) | 259444 (232171 to 283527) | 141.7% (124.5 to 163.1) | 2383.0 (2174.0 to 2644.7) | 4662.6 (4162.3 to 5090.1) | 2.11 (1.97 to 2.24) |
| **Mongolia** | 19244 (17397 to 21109) | 109916 (100373 to 120525) | 471.2% (432.5 to 507.5) | 1537.5 (1402.8 to 1688.6) | 3780.5 (3463.9 to 4099.4) | 3.07 (2.99 to 3.15) |
| **Montenegro** | 21840 (19523 to 24363) | 52543 (47257 to 57929) | 140.6% (120.9 to 160.2) | 3441.9 (3079.2 to 3832.2) | 5508.7 (4943.6 to 6082.5) | 1.52 (1.43 to 1.60) |
| **Morocco** | 734668 (665480 to 812592) | 4991730 (4556137 to 5432476) | 579.5% (539.0 to 624.1) | 4668.0 (4238.8 to 5177.6) | 13494.6 (12351.9 to 14686.4) | 3.56 (3.50 to 3.62) |
| **Mozambique** | 83556 (74693 to 92808) | 437079 (393607 to 482393) | 423.1% (386.3 to 471.0) | 1318.8 (1193.0 to 1440.6) | 3037.9 (2759.9 to 3320.8) | 2.85 (2.81 to 2.90) |
| **Myanmar** | 875520 (794133 to 963652) | 3925453 (3603035 to 4225553) | 348.4% (316.6 to 381.3) | 3502.8 (3174.9 to 3849.2) | 7732.6 (7110.4 to 8340.2) | 2.43 (2.36 to 2.49) |
| **Namibia** | 15553 (13906 to 17360) | 62219 (56237 to 68591) | 300.1% (274.9 to 329.8) | 2177.8 (1966.1 to 2416.0) | 3903.3 (3543.8 to 4298.5) | 1.91 (1.88 to 1.94) |
| **Nepal** | 392437 (357074 to 432189) | 1899269 (1756840 to 2050248) | 384.0% (355.9 to 414.1) | 3346.6 (3063.0 to 3673.2) | 7120.6 (6594.1 to 7648.2) | 2.47 (2.31 to 2.63) |
| **Netherlands** | 390255 (359947 to 422586) | 1028841 (921767 to 1141912) | 163.6% (139.3 to 191.3) | 2049.4 (1886.9 to 2228.3) | 3592.8 (3215.6 to 4002.1) | 1.74 (1.70 to 1.78) |
| **New Zealand** | 96273 (85841 to 108960) | 306966 (291523 to 321613) | 218.8% (189.6 to 250.4) | 2540.8 (2257.4 to 2882.6) | 4077.5 (3866.3 to 4284.2) | 2.00 (1.68 to 2.32) |
| **Nicaragua** | 84508 (77182 to 92619) | 448525 (411888 to 490396) | 430.7% (397.7 to 466.6) | 4521.4 (4134.3 to 4967.5) | 8177.8 (7554.0 to 8945.7) | 1.89 (1.85 to 1.94) |
| **Niger** | 84977 (76893 to 93428) | 523260 (475399 to 573036) | 515.8% (481.5 to 553.6) | 2353.3 (2142.0 to 2568.6) | 4713.6 (4303.9 to 5169.2) | 2.27 (2.24 to 2.30) |
| **Nigeria** | 1042967 (929113 to 1149835) | 4108042 (3684496 to 4516683) | 293.9% (281.3 to 306.6) | 2067.1 (1860.1 to 2284.3) | 3529.3 (3212.3 to 3869.8) | 1.70 (1.66 to 1.73) |
| **North Korea** | 487661 (444520 to 539476) | 1749768 (1601483 to 1924131) | 258.8% (238.4 to 281.0) | 2645.0 (2413.1 to 2926.0) | 5323.7 (4870.7 to 5862.1) | 2.22 (2.20 to 2.24) |
| **Northern Mariana Islands** | 1533 (1378 to 1702) | 5475 (4967 to 5986) | 257.1% (227.0 to 288.5) | 4852.8 (4436.4 to 5294.4) | 9676.7 (8855.0 to 10543.6) | 2.08 (2.00 to 2.15) |
| **Norway** | 169863 (150466 to 190562) | 319539 (281649 to 357827) | 88.1% (82.4 to 94.1) | 2951.9 (2593.2 to 3319.7) | 3900.6 (3409.4 to 4387.1) | 0.70 (0.60 to 0.81) |
| **Oman** | 34368 (30830 to 38005) | 257977 (231951 to 288731) | 650.6% (591.5 to 713.9) | 3944.8 (3581.1 to 4323.2) | 8409.6 (7697.0 to 9245.7) | 2.15 (2.01 to 2.28) |
| **Pakistan** | 2005192 (1793739 to 2238510) | 10902779 (9856302 to 11978317) | 443.7% (419.8 to 473.2) | 3061.0 (2748.2 to 3405.9) | 6886.4 (6210.1 to 7575.8) | 2.88 (2.68 to 3.08) |
| **Palestine** | 39648 (36324 to 43091) | 287081 (263787 to 311348) | 624.1% (572.9 to 676.3) | 4202.2 (3866.0 to 4560.4) | 9456.7 (8755.2 to 10216.6) | 2.74 (2.70 to 2.78) |
| **Panama** | 61504 (56027 to 67141) | 309869 (281736 to 337555) | 403.8% (370.5 to 445.0) | 3673.8 (3342.0 to 4024.0) | 7019.9 (6382.6 to 7642.3) | 2.13 (2.08 to 2.18) |
| **Papua New Guinea** | 126172 (113851 to 138004) | 820934 (755122 to 895441) | 550.6% (512.3 to 588.4) | 5197.1 (4752.0 to 5652.0) | 11469.8 (10641.5 to 12395.5) | 2.50 (2.43 to 2.58) |
| **Paraguay** | 79731 (71873 to 87570) | 397477 (367015 to 431594) | 398.5% (365.3 to 437.4) | 3287.1 (2977.3 to 3595.9) | 6410.7 (5934.0 to 6971.3) | 2.25 (2.14 to 2.36) |
| **Peru** | 251703 (230355 to 277625) | 1203814 (1109776 to 1308052) | 378.3% (349.2 to 414.0) | 1885.2 (1725.3 to 2083.5) | 3482.4 (3212.9 to 3779.3) | 2.04 (1.99 to 2.09) |
| **Philippines** | 1067282 (953689 to 1180922) | 3674882 (3334548 to 4047836) | 244.3% (228.9 to 261.0) | 3160.0 (2844.7 to 3499.0) | 4206.9 (3826.5 to 4624.1) | 0.94 (0.82 to 1.06) |
| **Poland** | 1483579 (1321591 to 1663701) | 3389639 (3033975 to 3803230) | 128.5% (120.0 to 137.7) | 3397.8 (3033.0 to 3815.5) | 5166.3 (4618.0 to 5773.8) | 1.41 (1.34 to 1.49) |
| **Portugal** | 428807 (394696 to 466478) | 1313146 (1194972 to 1447089) | 206.2% (184.1 to 232.8) | 3294.3 (3030.4 to 3584.6) | 6920.6 (6276.0 to 7600.5) | 2.35 (2.23 to 2.48) |
| **Principality of Monaco** | 907 (807 to 1002) | 2685 (2431 to 2984) | 195.9% (176.8 to 216.3) | 1719.3 (1519.3 to 1906.1) | 3859.8 (3452.1 to 4324.7) | 2.67 (2.63 to 2.70) |
| **Puerto Rico** | 214492 (198980 to 233969) | 604291 (558114 to 665923) | 181.7% (163.5 to 203.7) | 5930.5 (5489.4 to 6462.9) | 10580.9 (9744.0 to 11752.7) | 1.87 (1.71 to 2.03) |
| **Qatar** | 11688 (10443 to 12971) | 273127 (242901 to 304070) | 2236.7% (2043.5 to 2483.8) | 6793.5 (6240.5 to 7425.4) | 15006.6 (13792.8 to 16076.5) | 2.43 (2.29 to 2.57) |
| **Republic of Nauru** | 471 (431 to 522) | 1213 (1132 to 1312) | 157.6% (139.8 to 176.5) | 7650.0 (7059.5 to 8403.4) | 15756.4 (14737.2 to 16914.2) | 2.09 (1.93 to 2.25) |
| **Republic of Niue** | 168 (155 to 181) | 367 (339 to 395) | 119.0% (105.4 to 131.8) | 7947.6 (7314.9 to 8631.3) | 18119.4 (16711.0 to 19556.7) | 2.66 (2.56 to 2.76) |
| **Republic of Palau** | 931 (849 to 1020) | 4059 (3762 to 4350) | 336.1% (305.8 to 373.9) | 7926.3 (7260.9 to 8652.5) | 16752.1 (15624.2 to 17985.0) | 2.42 (2.37 to 2.47) |
| **Republic of San Marino** | 621 (559 to 685) | 2380 (2155 to 2659) | 283.3% (261.6 to 307.6) | 1977.9 (1767.8 to 2192.4) | 4206.0 (3768.5 to 4687.7) | 2.49 (2.45 to 2.54) |
| **Romania** | 620929 (554532 to 707264) | 1141002 (1038650 to 1264690) | 83.8% (68.3 to 97.8) | 2228.1 (1984.2 to 2534.4) | 3435.0 (3126.6 to 3812.5) | 1.45 (1.39 to 1.52) |
| **Russian Federation** | 3225202 (2874136 to 3606415) | 8174228 (7306105 to 9137361) | 153.4% (144.9 to 161.9) | 1814.3 (1616.3 to 2026.2) | 3641.7 (3256.9 to 4068.3) | 2.26 (2.19 to 2.33) |
| **Rwanda** | 43439 (38799 to 48253) | 155725 (140368 to 171671) | 258.5% (234.4 to 285.8) | 1370.8 (1243.5 to 1500.9) | 2083.3 (1902.8 to 2274.4) | 1.12 (1.01 to 1.22) |
| **Saint Kitts and Nevis** | 2078 (1926 to 2236) | 7313 (6571 to 8060) | 251.9% (224.2 to 278.6) | 5875.0 (5406.1 to 6403.2) | 9740.3 (8848.6 to 10700.9) | 1.57 (1.54 to 1.59) |
| **Saint Lucia** | 7249 (6650 to 7875) | 27648 (25623 to 29626) | 281.4% (255.0 to 306.8) | 7940.4 (7247.7 to 8647.6) | 11618.1 (10788.0 to 12441.3) | 1.18 (1.14 to 1.22) |
| **Saint Vincent and the Grenadines** | 5203 (4798 to 5598) | 16717 (15485 to 17972) | 221.3% (199.0 to 242.8) | 7071.6 (6515.4 to 7602.2) | 11816.1 (10973.9 to 12717.6) | 1.61 (1.57 to 1.64) |
| **Samoa** | 7356 (6776 to 7988) | 25759 (23671 to 28242) | 250.2% (229.1 to 272.0) | 7303.8 (6747.2 to 7932.7) | 15578.4 (14353.0 to 17028.4) | 2.50 (2.38 to 2.62) |
| **Sao Tome and Principe** | 1823 (1651 to 2008) | 8276 (7609 to 9071) | 354.1% (326.5 to 383.2) | 2590.5 (2356.2 to 2840.1) | 5674.3 (5219.9 to 6169.3) | 2.58 (2.56 to 2.59) |
| **Saudi Arabia** | 369750 (335642 to 403002) | 3289161 (2976473 to 3642757) | 789.6% (726.1 to 852.0) | 4967.4 (4547.6 to 5381.3) | 10849.2 (9941.6 to 11869.7) | 2.49 (2.42 to 2.57) |
| **Senegal** | 133332 (121041 to 146139) | 653534 (607555 to 700136) | 390.2% (361.5 to 421.2) | 3460.0 (3151.2 to 3772.9) | 6971.7 (6508.8 to 7466.7) | 2.46 (2.33 to 2.59) |
| **Serbia** | 431245 (385715 to 478451) | 920558 (834271 to 1030927) | 113.5% (91.8 to 140.3) | 3701.3 (3325.3 to 4105.8) | 6026.0 (5466.2 to 6752.8) | 1.53 (1.44 to 1.62) |
| **Seychelles** | 1929 (1756 to 2126) | 12961 (11939 to 14024) | 571.8% (525.6 to 624.5) | 3341.6 (3043.2 to 3689.0) | 10804.4 (10024.6 to 11618.8) | 3.82 (3.63 to 4.01) |
| **Sierra Leone** | 55230 (49822 to 60917) | 250364 (227769 to 274434) | 353.3% (327.1 to 381.6) | 2307.1 (2080.8 to 2547.0) | 4939.6 (4498.1 to 5342.4) | 2.54 (2.48 to 2.59) |
| **Singapore** | 144809 (133914 to 156563) | 669052 (616681 to 723554) | 362.0% (329.6 to 394.3) | 5563.6 (5149.8 to 6009.8) | 7966.1 (7355.1 to 8594.7) | 0.98 (0.90 to 1.06) |
| **Slovakia** | 160309 (143828 to 177478) | 352175 (316844 to 384641) | 119.7% (102.1 to 136.7) | 2701.5 (2419.5 to 2994.2) | 3908.4 (3506.2 to 4286.8) | 1.17 (1.10 to 1.24) |
| **Slovenia** | 72254 (65210 to 80303) | 153583 (138795 to 171163) | 112.6% (98.6 to 129.3) | 2953.3 (2667.0 to 3275.7) | 3927.0 (3530.5 to 4363.5) | 0.79 (0.64 to 0.93) |
| **Solomon Islands** | 6864 (6259 to 7583) | 39655 (36617 to 43052) | 477.7% (438.7 to 520.3) | 3787.6 (3494.8 to 4153.4) | 8571.6 (7979.5 to 9221.0) | 2.62 (2.52 to 2.72) |
| **Somalia** | 48008 (41759 to 53886) | 257652 (231884 to 286196) | 436.7% (400.1 to 478.8) | 1571.0 (1419.6 to 1724.9) | 2921.4 (2655.4 to 3208.1) | 2.03 (2.01 to 2.04) |
| **South Africa** | 664019 (594636 to 734657) | 2816759 (2564649 to 3112714) | 324.2% (304.0 to 346.0) | 2846.4 (2548.3 to 3175.5) | 5643.5 (5138.4 to 6233.7) | 2.33 (2.29 to 2.37) |
| **South Korea** | 1392785 (1277396 to 1508230) | 8425169 (7963975 to 8862735) | 504.9% (466.9 to 551.3) | 3945.0 (3624.5 to 4271.2) | 10000.3 (9421.5 to 10544.8) | 3.28 (3.10 to 3.45) |
| **South Sudan** | 39346 (34854 to 43877) | 126190 (114662 to 138767) | 220.7% (199.5 to 242.7) | 1396.7 (1261.1 to 1548.6) | 2649.7 (2430.3 to 2878.1) | 2.07 (2.05 to 2.09) |
| **Spain** | 1936710 (1796364 to 2106985) | 4945224 (4582911 to 5302601) | 155.3% (137.9 to 171.6) | 3745.7 (3481.6 to 4076.3) | 6234.3 (5738.7 to 6710.2) | 1.46 (1.36 to 1.56) |
| **Sri Lanka** | 491713 (450683 to 543545) | 2702813 (2520055 to 2920556) | 449.7% (414.9 to 483.7) | 4328.8 (3953.3 to 4811.6) | 10034.5 (9365.4 to 10815.4) | 2.73 (2.60 to 2.87) |
| **Sudan** | 340052 (310279 to 372581) | 1889179 (1724529 to 2054375) | 455.6% (419.9 to 492.8) | 3246.9 (2961.9 to 3569.3) | 7668.5 (7009.3 to 8326.6) | 2.75 (2.70 to 2.81) |
| **Suriname** | 15311 (13952 to 16798) | 75247 (69863 to 81537) | 391.4% (356.7 to 432.8) | 5435.2 (4964.0 to 5958.9) | 11587.9 (10784.6 to 12530.8) | 2.62 (2.54 to 2.70) |
| **Swaziland** | 8772 (7899 to 9759) | 39873 (36736 to 43446) | 354.5% (321.1 to 386.0) | 2643.4 (2406.9 to 2938.5) | 6136.9 (5662.0 to 6668.0) | 2.93 (2.81 to 3.05) |
| **Sweden** | 333885 (293281 to 377178) | 740484 (651868 to 829132) | 121.8% (109.6 to 135.6) | 2640.5 (2304.9 to 2996.2) | 4406.5 (3856.0 to 4954.3) | 1.68 (1.63 to 1.73) |
| **Switzerland** | 310289 (282304 to 339943) | 933017 (848205 to 1013439) | 200.7% (180.3 to 225.2) | 3312.0 (3001.2 to 3631.1) | 6404.4 (5800.9 to 7003.5) | 2.02 (1.95 to 2.10) |
| **Syria** | 235275 (213239 to 257021) | 1195739 (1089024 to 1302914) | 408.2% (372.9 to 443.6) | 3861.6 (3517.7 to 4217.4) | 8370.4 (7658.7 to 9087.0) | 2.48 (2.44 to 2.52) |
| **Taiwan (Province of China)** | 581712 (540427 to 624281) | 2189494 (2040777 to 2371379) | 276.4% (250.4 to 300.6) | 3278.7 (3067.7 to 3514.1) | 5627.1 (5251.3 to 6062.7) | 1.62 (1.55 to 1.68) |
| **Tajikistan** | 50176 (45798 to 55021) | 283645 (257742 to 309693) | 465.3% (423.0 to 503.1) | 1609.6 (1465.7 to 1761.5) | 3883.0 (3535.0 to 4229.9) | 2.95 (2.85 to 3.04) |
| **Tanzania** | 129558 (113905 to 144806) | 724567 (661091 to 790160) | 459.3% (421.2 to 504.5) | 1124.4 (1011.1 to 1235.5) | 2382.0 (2195.9 to 2581.6) | 2.47 (2.43 to 2.51) |
| **Thailand** | 958665 (867026 to 1055544) | 5493290 (4965781 to 6010526) | 473.0% (423.0 to 526.3) | 2439.0 (2216.1 to 2688.9) | 5277.2 (4800.4 to 5765.2) | 2.46 (2.43 to 2.48) |
| **The Bahamas** | 8960 (8197 to 9726) | 40451 (36940 to 44452) | 351.5% (320.7 to 384.3) | 5093.6 (4675.3 to 5525.5) | 9289.0 (8502.4 to 10164.4) | 1.94 (1.90 to 1.99) |
| **The Gambia** | 10406 (9274 to 11518) | 65661 (60032 to 71790) | 531.0% (493.6 to 572.3) | 2274.7 (2065.4 to 2488.8) | 5057.6 (4661.4 to 5488.9) | 2.66 (2.61 to 2.72) |
| **Timor-Leste** | 5875 (5329 to 6533) | 52101 (46691 to 56697) | 786.8% (726.5 to 855.7) | 1687.8 (1550.1 to 1853.2) | 5749.4 (5153.8 to 6262.8) | 4.07 (4.04 to 4.09) |
| **Togo** | 28433 (25460 to 31449) | 170479 (156812 to 184670) | 499.6% (461.9 to 540.7) | 1761.3 (1592.3 to 1927.7) | 3432.4 (3191.8 to 3686.8) | 2.16 (2.12 to 2.19) |
| **Tokelau** | 115 (105 to 126) | 248 (231 to 267) | 115.4% (101.7 to 129.5) | 8749.8 (7984.1 to 9577.8) | 17398.3 (16176.3 to 18759.7) | 2.15 (2.04 to 2.26) |
| **Tonga** | 4250 (3898 to 4607) | 11727 (10901 to 12635) | 175.9% (161.1 to 194.0) | 6815.3 (6292.0 to 7362.8) | 13728.7 (12769.1 to 14762.1) | 2.27 (2.24 to 2.31) |
| **Trinidad and Tobago** | 72574 (67316 to 77894) | 245952 (227142 to 265079) | 238.9% (212.0 to 272.0) | 8194.0 (7623.9 to 8805.0) | 13043.8 (12050.8 to 14072.3) | 1.42 (1.37 to 1.46) |
| **Tunisia** | 191230 (173564 to 209961) | 1310979 (1184615 to 1442991) | 585.6% (537.8 to 628.4) | 3480.3 (3158.3 to 3832.8) | 9590.9 (8693.4 to 10543.0) | 3.14 (2.95 to 3.34) |
| **Turkey** | 970166 (904248 to 1040267) | 6125995 (5615026 to 6674158) | 531.4% (486.4 to 582.3) | 2571.8 (2400.5 to 2744.4) | 6372.4 (5846.9 to 6943.1) | 3.00 (2.77 to 3.23) |
| **Turkmenistan** | 32551 (29612 to 35324) | 171715 (157191 to 188073) | 427.5% (386.8 to 468.6) | 1467.3 (1339.7 to 1588.8) | 3689.7 (3382.0 to 4031.7) | 3.02 (2.92 to 3.13) |
| **Tuvalu** | 373 (343 to 408) | 1241 (1152 to 1345) | 232.6% (210.6 to 256.5) | 5052.1 (4657.6 to 5480.3) | 11053.7 (10269.7 to 11975.5) | 2.44 (2.35 to 2.52) |
| **Uganda** | 98911 (87940 to 109762) | 494853 (446769 to 545013) | 400.3% (365.1 to 434.3) | 1376.0 (1242.6 to 1514.5) | 2604.7 (2367.7 to 2844.2) | 2.01 (1.96 to 2.06) |
| **Ukraine** | 1219395 (1082765 to 1373104) | 2378550 (2103377 to 2668607) | 95.1% (84.0 to 107.1) | 1801.7 (1595.6 to 2021.9) | 3419.2 (3021.0 to 3823.3) | 1.96 (1.86 to 2.06) |
| **United Arab Emirates** | 33465 (29579 to 37653) | 775578 (693011 to 861153) | 2217.6% (2015.1 to 2449.9) | 4887.5 (4472.7 to 5331.6) | 9101.9 (8301.7 to 9962.5) | 1.81 (1.69 to 1.93) |
| **United Kingdom** | 2132364 (1914305 to 2377773) | 7205415 (6608088 to 7843794) | 237.9% (222.5 to 256.1) | 2714.9 (2433.2 to 3024.4) | 7314.2 (6655.4 to 7959.9) | 3.06 (2.94 to 3.17) |
| **United States** | 9748635 (8831917 to 10778381) | 44188569 (41776316 to 46822326) | 353.3% (325.2 to 381.4) | 3268.0 (2947.5 to 3621.1) | 8439.7 (7985.1 to 8947.3) | 3.27 (3.16 to 3.38) |
| **Uruguay** | 77824 (70319 to 86501) | 256809 (231448 to 281909) | 230.0% (204.8 to 256.9) | 2062.9 (1862.1 to 2299.1) | 5212.9 (4709.0 to 5714.6) | 3.25 (3.18 to 3.31) |
| **Uzbekistan** | 233517 (213598 to 257392) | 1633746 (1506209 to 1792649) | 599.6% (546.7 to 663.8) | 1813.3 (1664.9 to 1995.5) | 5320.5 (4887.9 to 5818.3) | 3.77 (3.56 to 3.99) |
| **Vanuatu** | 3877 (3528 to 4279) | 24523 (22727 to 26441) | 532.5% (491.9 to 574.1) | 4644.4 (4248.8 to 5093.4) | 10922.5 (10143.9 to 11718.0) | 2.70 (2.67 to 2.73) |
| **Venezuela** | 436491 (399507 to 471586) | 2029317 (1859356 to 2235696) | 364.9% (326.9 to 409.3) | 3763.7 (3471.7 to 4087.9) | 6663.1 (6118.6 to 7336.2) | 1.92 (1.87 to 1.97) |
| **Vietnam** | 885509 (800451 to 974208) | 4324015 (4032595 to 4640545) | 388.3% (358.5 to 420.8) | 2120.1 (1922.0 to 2329.8) | 4318.9 (4035.2 to 4631.2) | 2.30 (2.22 to 2.38) |
| **Virgin Islands, U.S.** | 6190 (5596 to 6796) | 20028 (18353 to 21656) | 223.6% (200.7 to 249.3) | 6616.8 (6027.9 to 7261.1) | 12906.6 (11823.4 to 13924.4) | 2.26 (2.16 to 2.36) |
| **Yemen** | 150484 (134734 to 166529) | 1016375 (922866 to 1117627) | 575.4% (538.1 to 613.9) | 2572.0 (2333.0 to 2853.2) | 5688.4 (5201.6 to 6290.2) | 2.41 (2.24 to 2.57) |
| **Zambia** | 79274 (71693 to 86782) | 437015 (397099 to 481908) | 451.3% (412.3 to 489.7) | 2316.9 (2127.9 to 2525.4) | 4391.8 (4039.0 to 4786.2) | 2.07 (2.05 to 2.10) |
| **Zimbabwe** | 91467 (81650 to 100562) | 350692 (322282 to 383338) | 283.4% (259.0 to 314.9) | 1939.6 (1746.5 to 2125.4) | 4164.3 (3836.4 to 4553.5) | 2.65 (2.60 to 2.70) |

ASPR, age-standardized prevalence rate; UI, uncertainty interval; EAPC, estimated annual percentage change; CI, confidence interval.

# Supplementary Table 8. The death cases and age-standardized mortality rate of type 2 diabetes mellitus in 1990 and 2021, and its temporal trends from 1990 to 2021 in 204 countries/territories

| **Countries/ territories** | **Number of death cases in 1990**  **(95%UI)** | **Number of death cases in 2021**  **(95%UI)** | **Percentage change in death counts, 1990-2021(95%UI)** | **1990 ASMR per 100000**  **(95%UI)** | **2021 ASMR per 100000**  **(95%UI)** | **1990-2021 EAPC (%)**  **(95%CI)** |
| --- | --- | --- | --- | --- | --- | --- |
| **Afghanistan** | 1614 (1214 to 2198) | 3352 (2543 to 4525) | 107.7% (49.9 to 175.6) | 24.4 (18.9 to 32.8) | 39.3 (30.1 to 51.4) | 1.72 (1.64 to 1.79) |
| **Albania** | 86 (70 to 104) | 203 (161 to 259) | 136.3% (73.4 to 231.5) | 4.8 (4.0 to 5.8) | 4.7 (3.7 to 5.9) | 0.07 (-0.19 to 0.34) |
| **Algeria** | 1101 (869 to 1456) | 5342 (4275 to 6573) | 385.3% (260.8 to 540.0) | 12.2 (9.9 to 15.9) | 18.5 (15.0 to 22.6) | 1.87 (1.67 to 2.06) |
| **American Samoa** | 18 (15 to 21) | 50 (42 to 60) | 171.2% (112.3 to 253.4) | 93.1 (78.6 to 106.4) | 112.8 (96.7 to 133.4) | 0.53 (0.18 to 0.87) |
| **Andorra** | 6 (5 to 8) | 14 (10 to 18) | 127.7% (53.3 to 237.1) | 12.2 (9.2 to 16.1) | 8.3 (6.0 to 10.8) | -0.94 (-1.19 to -0.69) |
| **Angola** | 1596 (1260 to 2042) | 4702 (3539 to 5974) | 194.5% (106.8 to 308.8) | 48.2 (38.9 to 60.2) | 48.2 (37.8 to 59.4) | -0.16 (-0.25 to -0.06) |
| **Antigua and Barbuda** | 36 (34 to 39) | 56 (52 to 60) | 54.4% (40.8 to 67.8) | 65.0 (60.0 to 69.8) | 57.2 (52.6 to 60.8) | -0.81 (-1.00 to -0.61) |
| **Argentina** | 7081 (6689 to 7420) | 9170 (8495 to 9745) | 29.5% (21.2 to 38.4) | 22.6 (21.3 to 23.8) | 16.0 (14.8 to 17.0) | -1.35 (-1.59 to -1.11) |
| **Armenia** | 453 (416 to 489) | 609 (540 to 692) | 34.5% (17.1 to 55.4) | 17.3 (15.8 to 18.8) | 14.1 (12.5 to 16.0) | -0.95 (-2.01 to 0.12) |
| **Australia** | 2122 (1935 to 2272) | 4281 (3695 to 4670) | 101.8% (84.4 to 118.9) | 11.1 (10.1 to 11.9) | 8.4 (7.4 to 9.1) | -1.21 (-1.50 to -0.91) |
| **Austria** | 1765 (1620 to 1871) | 1972 (1655 to 2166) | 11.7% (0.7 to 21.6) | 14.1 (13.0 to 14.9) | 9.1 (7.8 to 9.9) | -0.62 (-1.16 to -0.08) |
| **Azerbaijan** | 525 (419 to 656) | 1671 (1179 to 2184) | 218.1% (112.2 to 351.7) | 10.7 (8.6 to 13.4) | 16.9 (12.1 to 21.9) | 1.22 (0.80 to 1.64) |
| **Bahrain** | 126 (111 to 141) | 663 (535 to 772) | 425.6% (311.0 to 550.3) | 106.1 (92.7 to 119.0) | 126.2 (103.7 to 145.4) | 0.32 (-0.10 to 0.73) |
| **Bangladesh** | 10990 (9474 to 12637) | 38594 (31159 to 46586) | 251.2% (173.3 to 354.2) | 28.1 (24.0 to 32.1) | 35.1 (28.4 to 42.5) | 0.43 (-0.05 to 0.92) |
| **Barbados** | 227 (213 to 240) | 309 (245 to 375) | 36.5% (8.4 to 66.8) | 74.3 (69.8 to 78.8) | 59.4 (47.0 to 72.0) | -0.94 (-1.17 to -0.71) |
| **Belarus** | 488 (457 to 524) | 644 (529 to 756) | 31.8% (6.5 to 61.8) | 3.7 (3.5 to 4.0) | 4.0 (3.3 to 4.7) | -1.72 (-2.71 to -0.71) |
| **Belgium** | 1780 (1613 to 1919) | 1414 (1174 to 1563) | -20.6% (-28.5 to -13.7) | 11.2 (10.1 to 12.0) | 5.0 (4.3 to 5.5) | -2.66 (-2.87 to -2.45) |
| **Belize** | 44 (41 to 46) | 152 (134 to 171) | 248.4% (206.9 to 296.5) | 47.3 (44.0 to 50.2) | 54.0 (47.8 to 60.8) | 0.39 (-0.18 to 0.96) |
| **Benin** | 497 (419 to 585) | 1591 (1265 to 1970) | 220.3% (143.7 to 314.7) | 27.4 (23.1 to 32.1) | 36.0 (29.4 to 43.6) | 0.88 (0.70 to 1.05) |
| **Bermuda** | 21 (19 to 22) | 25 (21 to 30) | 18.6% (0.8 to 44.1) | 35.3 (32.9 to 37.5) | 16.6 (14.1 to 20.2) | -2.57 (-2.70 to -2.44) |
| **Bhutan** | 45 (35 to 59) | 173 (127 to 220) | 282.5% (188.1 to 415.7) | 23.0 (17.4 to 29.6) | 31.3 (23.1 to 40.0) | 1.03 (1.00 to 1.06) |
| **Bolivia** | 1163 (980 to 1405) | 3557 (2848 to 4619) | 205.9% (134.5 to 313.8) | 39.2 (33.3 to 47.3) | 42.3 (34.2 to 54.5) | 0.26 (0.24 to 0.29) |
| **Bosnia and Herzegovina** | 528 (455 to 616) | 1956 (1571 to 2416) | 270.3% (179.5 to 375.0) | 14.5 (12.5 to 17.0) | 30.1 (24.1 to 37.2) | 2.98 (2.63 to 3.32) |
| **Botswana** | 273 (207 to 354) | 726 (589 to 889) | 165.7% (92.3 to 274.9) | 57.2 (44.5 to 72.9) | 59.8 (49.3 to 73.0) | 0.44 (0.15 to 0.73) |
| **Brazil** | 25559 (24273 to 26424) | 63219 (57845 to 66623) | 147.3% (135.2 to 156.8) | 31.5 (29.5 to 32.8) | 25.8 (23.6 to 27.3) | -0.60 (-0.72 to -0.47) |
| **Brunei** | 72 (62 to 82) | 140 (122 to 161) | 94.9% (60.4 to 137.3) | 81.5 (70.1 to 93.4) | 49.9 (43.5 to 57.2) | -1.26 (-1.44 to -1.08) |
| **Bulgaria** | 1911 (1746 to 2075) | 2303 (2006 to 2607) | 20.5% (3.6 to 41.6) | 16.2 (15.0 to 17.4) | 15.6 (13.6 to 17.7) | -0.56 (-0.86 to -0.25) |
| **Burkina Faso** | 1257 (1042 to 1514) | 2649 (2099 to 3290) | 110.8% (56.3 to 176.4) | 34.6 (29.2 to 41.3) | 33.4 (27.3 to 40.4) | -0.12 (-0.19 to -0.05) |
| **Burundi** | 1089 (815 to 1426) | 1762 (1241 to 2628) | 61.8% (18.0 to 118.5) | 51.8 (39.0 to 68.1) | 44.2 (31.1 to 64.8) | -0.99 (-1.15 to -0.82) |
| **Cambodia** | 1183 (949 to 1438) | 3574 (2560 to 4717) | 202.1% (120.7 to 311.1) | 28.0 (22.8 to 33.8) | 32.0 (23.2 to 41.7) | 0.42 (0.28 to 0.56) |
| **Cameroon** | 1429 (1132 to 1789) | 5329 (3986 to 7061) | 273.0% (176.4 to 404.7) | 38.0 (30.3 to 47.1) | 50.3 (38.7 to 65.5) | 0.80 (0.56 to 1.04) |
| **Canada** | 4081 (3736 to 4355) | 5935 (5205 to 6465) | 45.4% (33.3 to 58.0) | 12.6 (11.5 to 13.5) | 7.5 (6.7 to 8.2) | -2.35 (-2.95 to -1.75) |
| **Cape Verde** | 26 (21 to 30) | 148 (120 to 177) | 478.8% (355.5 to 645.3) | 10.8 (9.1 to 12.7) | 35.5 (28.8 to 42.6) | 3.26 (2.75 to 3.77) |
| **Central African Republic** | 612 (510 to 717) | 1163 (869 to 1511) | 90.0% (43.0 to 147.6) | 61.2 (51.5 to 70.7) | 61.8 (47.9 to 77.2) | 0.03 (-0.05 to 0.11) |
| **Chad** | 578 (450 to 760) | 1753 (1328 to 2338) | 203.2% (125.5 to 300.8) | 22.7 (17.6 to 29.8) | 35.5 (27.3 to 46.4) | 1.41 (1.11 to 1.71) |
| **Chile** | 1493 (1418 to 1584) | 2868 (2553 to 3098) | 92.1% (75.0 to 109.5) | 15.6 (14.8 to 16.6) | 10.9 (9.7 to 11.8) | -0.99 (-1.39 to -0.59) |
| **China** | 66459 (57966 to 75921) | 174515 (144850 to 207117) | 162.6% (109.1 to 232.3) | 9.3 (8.2 to 10.5) | 8.7 (7.3 to 10.3) | -0.22 (-0.49 to 0.04) |
| **Colombia** | 3349 (3165 to 3505) | 7101 (5953 to 8332) | 112.0% (79.5 to 148.3) | 20.5 (19.2 to 21.6) | 12.8 (10.8 to 15.0) | -2.55 (-2.93 to -2.16) |
| **Comoros** | 73 (54 to 96) | 182 (125 to 239) | 150.0% (75.2 to 246.6) | 43.2 (32.4 to 56.2) | 43.0 (30.1 to 56.0) | -0.17 (-0.27 to -0.08) |
| **Congo** | 608 (495 to 730) | 1436 (1125 to 1834) | 136.3% (81.1 to 209.5) | 65.7 (54.0 to 78.5) | 63.5 (51.8 to 78.5) | -0.32 (-0.43 to -0.21) |
| **Cook Islands** | 17 (14 to 19) | 29 (24 to 34) | 72.5% (32.5 to 115.2) | 147.6 (127.5 to 171.2) | 112.5 (93.1 to 133.5) | -1.08 (-1.20 to -0.96) |
| **Costa Rica** | 246 (228 to 261) | 1020 (892 to 1152) | 315.2% (269.7 to 369.7) | 14.5 (13.5 to 15.4) | 18.6 (16.3 to 20.9) | -0.18 (-0.97 to 0.62) |
| **Cote d'Ivoire** | 1010 (835 to 1216) | 3762 (2899 to 4842) | 272.4% (181.0 to 397.8) | 31.7 (26.6 to 37.7) | 41.4 (33.0 to 52.2) | 0.78 (0.53 to 1.02) |
| **Croatia** | 709 (652 to 767) | 1522 (1322 to 1702) | 114.6% (83.7 to 147.9) | 12.7 (11.7 to 13.8) | 15.2 (13.3 to 17.0) | -0.21 (-0.70 to 0.28) |
| **Cuba** | 2143 (2025 to 2249) | 1981 (1728 to 2243) | -7.6% (-19.9 to 5.8) | 21.4 (20.2 to 22.4) | 9.7 (8.5 to 11.0) | -2.67 (-3.35 to -1.99) |
| **Cyprus** | 442 (378 to 508) | 508 (428 to 592) | 14.8% (-6.4 to 39.9) | 77.1 (65.9 to 87.6) | 28.4 (24.1 to 33.0) | -3.57 (-3.76 to -3.37) |
| **Czech Republic** | 1878 (1700 to 2086) | 4296 (3674 to 4988) | 128.8% (94.8 to 171.0) | 13.4 (12.2 to 14.9) | 18.2 (15.7 to 21.1) | 2.99 (2.08 to 3.90) |
| **Democratic Republic of the Congo** | 6392 (5019 to 7998) | 15147 (11478 to 19734) | 137.0% (69.3 to 229.3) | 49.8 (39.8 to 61.1) | 49.7 (38.0 to 63.7) | -0.05 (-0.16 to 0.06) |
| **Denmark** | 797 (743 to 846) | 1340 (1178 to 1458) | 68.0% (53.2 to 83.7) | 9.5 (8.9 to 10.0) | 10.0 (8.9 to 10.8) | 0.23 (-0.30 to 0.77) |
| **Djibouti** | 38 (29 to 52) | 227 (166 to 319) | 493.8% (318.8 to 739.3) | 35.7 (27.7 to 47.9) | 46.1 (34.8 to 62.8) | 0.80 (0.69 to 0.91) |
| **Dominica** | 42 (38 to 45) | 58 (50 to 68) | 38.5% (17.9 to 64.5) | 70.9 (65.0 to 76.7) | 73.1 (63.8 to 85.1) | -0.20 (-0.30 to -0.10) |
| **Dominican Republic** | 760 (660 to 862) | 2763 (2171 to 3462) | 263.5% (179.1 to 379.3) | 22.7 (19.6 to 25.6) | 28.0 (22.0 to 35.1) | 1.11 (0.94 to 1.29) |
| **Ecuador** | 1120 (1060 to 1178) | 4434 (3541 to 5446) | 295.8% (211.7 to 390.6) | 22.7 (21.4 to 23.8) | 28.4 (22.8 to 34.7) | 0.64 (0.19 to 1.10) |
| **Egypt** | 6469 (5837 to 7159) | 27199 (22225 to 32831) | 320.4% (238.5 to 432.1) | 29.1 (26.1 to 32.4) | 55.3 (46.6 to 65.6) | 3.04 (2.65 to 3.43) |
| **El Salvador** | 629 (570 to 694) | 2629 (2108 to 3220) | 318.1% (226.2 to 425.0) | 21.6 (19.5 to 23.8) | 41.9 (33.3 to 51.4) | 2.03 (1.81 to 2.24) |
| **Equatorial Guinea** | 95 (74 to 116) | 276 (194 to 376) | 191.7% (95.2 to 330.6) | 55.1 (43.5 to 66.0) | 64.5 (47.3 to 85.7) | 0.58 (0.32 to 0.85) |
| **Eritrea** | 464 (359 to 598) | 1189 (880 to 1549) | 156.3% (94.2 to 230.4) | 49.1 (38.2 to 62.2) | 54.2 (41.6 to 69.5) | 0.33 (0.25 to 0.40) |
| **Estonia** | 79 (72 to 86) | 292 (252 to 332) | 268.0% (215.4 to 324.7) | 3.9 (3.6 to 4.2) | 10.1 (8.7 to 11.4) | 2.03 (1.08 to 2.99) |
| **Ethiopia** | 11317 (9660 to 13181) | 13736 (11653 to 16047) | 21.4% (-4.9 to 50.7) | 65.3 (56.8 to 75.2) | 37.0 (31.6 to 43.1) | -2.31 (-2.50 to -2.11) |
| **Federated States of Micronesia** | 40 (32 to 50) | 78 (60 to 103) | 97.4% (46.1 to 172.5) | 87.2 (70.4 to 109.0) | 117.3 (91.0 to 150.1) | 0.92 (0.65 to 1.18) |
| **Fiji** | 626 (530 to 734) | 1810 (1411 to 2292) | 189.2% (112.2 to 283.8) | 192.0 (163.3 to 224.5) | 265.2 (213.0 to 328.3) | 0.86 (0.62 to 1.10) |
| **Finland** | 454 (412 to 490) | 553 (470 to 609) | 21.9% (10.8 to 33.5) | 6.2 (5.6 to 6.7) | 3.8 (3.3 to 4.1) | -1.88 (-2.26 to -1.50) |
| **France** | 7903 (7191 to 8529) | 12699 (10603 to 13842) | 60.7% (43.4 to 74.8) | 8.9 (8.2 to 9.6) | 6.9 (5.9 to 7.5) | -0.76 (-1.27 to -0.24) |
| **Gabon** | 333 (266 to 426) | 670 (503 to 871) | 101.2% (51.4 to 163.5) | 63.9 (50.7 to 81.9) | 74.5 (57.1 to 95.8) | 0.43 (0.28 to 0.57) |
| **Georgia** | 637 (564 to 724) | 1015 (878 to 1145) | 59.4% (35.4 to 86.2) | 10.1 (9.0 to 11.5) | 16.6 (14.3 to 18.8) | 2.75 (2.03 to 3.49) |
| **Germany** | 20186 (18387 to 21642) | 20885 (17635 to 22917) | 3.5% (-6.3 to 12.6) | 15.0 (13.7 to 16.0) | 9.0 (7.8 to 9.8) | -2.00 (-2.24 to -1.76) |
| **Ghana** | 1401 (1143 to 1756) | 6667 (5204 to 8261) | 375.8% (235.9 to 548.9) | 26.9 (22.2 to 32.9) | 47.3 (37.4 to 58.1) | 2.37 (2.04 to 2.71) |
| **Greece** | 1160 (1059 to 1253) | 1744 (1524 to 1907) | 50.4% (35.9 to 66.5) | 7.8 (7.1 to 8.4) | 6.2 (5.5 to 6.7) | -0.75 (-1.15 to -0.36) |
| **Greenland** | 4 (4 to 5) | 6 (5 to 7) | 30.9% (4.2 to 65.3) | 14.9 (13.0 to 17.1) | 8.9 (7.3 to 11.0) | -1.76 (-1.89 to -1.64) |
| **Grenada** | 64 (59 to 70) | 84 (73 to 93) | 30.2% (12.3 to 49.1) | 83.0 (75.8 to 90.0) | 81.6 (71.7 to 90.5) | 0.01 (-0.34 to 0.37) |
| **Guam** | 26 (24 to 29) | 40 (35 to 45) | 51.2% (28.5 to 76.1) | 43.0 (38.2 to 48.1) | 18.7 (16.5 to 21.2) | -2.25 (-2.49 to -2.01) |
| **Guatemala** | 556 (532 to 581) | 6656 (5771 to 7627) | 1096.5% (929.2 to 1281.3) | 19.1 (18.3 to 20.0) | 62.2 (54.2 to 70.9) | 2.64 (2.09 to 3.19) |
| **Guinea** | 846 (631 to 1096) | 1999 (1532 to 2560) | 136.3% (75.4 to 236.4) | 28.3 (20.9 to 36.5) | 40.3 (31.5 to 50.8) | 1.27 (1.05 to 1.48) |
| **Guinea-Bissau** | 145 (114 to 176) | 318 (249 to 386) | 119.1% (62.9 to 187.4) | 41.4 (33.0 to 50.1) | 53.5 (42.9 to 63.5) | 0.91 (0.74 to 1.07) |
| **Guyana** | 277 (250 to 304) | 503 (397 to 630) | 81.8% (37.4 to 128.7) | 77.3 (69.9 to 84.6) | 83.9 (66.6 to 104.0) | 0.07 (-0.38 to 0.52) |
| **Haiti** | 2440 (1928 to 3001) | 4845 (3534 to 6504) | 98.6% (44.2 to 182.7) | 83.5 (66.5 to 102.4) | 76.9 (57.1 to 102.6) | -0.17 (-0.22 to -0.12) |
| **Honduras** | 292 (246 to 337) | 1724 (1352 to 2268) | 490.8% (353.7 to 689.1) | 14.9 (12.6 to 17.1) | 29.2 (22.9 to 37.9) | 2.36 (2.14 to 2.57) |
| **Hungary** | 1789 (1657 to 1924) | 2719 (2406 to 3000) | 52.0% (35.5 to 71.2) | 12.1 (11.3 to 13.0) | 13.0 (11.6 to 14.3) | 0.48 (0.12 to 0.85) |
| **Iceland** | 16 (15 to 17) | 26 (22 to 29) | 62.5% (45.4 to 80.9) | 5.3 (4.8 to 5.7) | 4.0 (3.4 to 4.4) | -0.89 (-1.03 to -0.74) |
| **India** | 85586 (74092 to 95492) | 320278 (281785 to 359571) | 274.2% (207.5 to 346.2) | 22.0 (18.8 to 24.9) | 30.3 (26.8 to 34.0) | 1.21 (0.97 to 1.45) |
| **Indonesia** | 17890 (14733 to 20777) | 60336 (49609 to 70880) | 237.3% (171.1 to 318.0) | 19.7 (16.1 to 23.1) | 28.6 (23.6 to 33.1) | 1.25 (1.16 to 1.33) |
| **Iran** | 2684 (2307 to 3170) | 14278 (12642 to 15793) | 431.9% (318.3 to 534.6) | 13.7 (11.6 to 16.1) | 20.8 (18.3 to 23.0) | 1.89 (1.67 to 2.12) |
| **Iraq** | 2941 (2410 to 3639) | 9469 (7240 to 11624) | 222.0% (136.6 to 327.5) | 39.3 (32.3 to 48.5) | 47.9 (37.9 to 57.6) | 0.29 (0.09 to 0.50) |
| **Ireland** | 435 (404 to 468) | 410 (347 to 458) | -5.7% (-17.2 to 6.9) | 10.7 (9.8 to 11.5) | 4.8 (4.1 to 5.3) | -2.41 (-2.62 to -2.20) |
| **Israel** | 948 (879 to 1005) | 2412 (2039 to 2640) | 154.5% (127.4 to 177.3) | 20.4 (18.8 to 21.5) | 17.6 (15.0 to 19.1) | -1.08 (-1.93 to -0.22) |
| **Italy** | 17962 (16337 to 18824) | 19039 (15793 to 20852) | 6.0% (-3.7 to 12.9) | 19.5 (17.6 to 20.4) | 10.3 (8.7 to 11.1) | -1.82 (-1.93 to -1.70) |
| **Jamaica** | 1215 (1155 to 1281) | 2144 (1686 to 2685) | 76.5% (40.1 to 119.1) | 66.4 (63.1 to 70.0) | 66.6 (52.3 to 83.7) | -0.22 (-0.50 to 0.05) |
| **Japan** | 11465 (10599 to 11980) | 9975 (8077 to 11038) | -13.0% (-24.0 to -6.6) | 7.0 (6.4 to 7.4) | 2.1 (1.8 to 2.3) | -3.69 (-4.33 to -3.05) |
| **Jordan** | 585 (494 to 696) | 2195 (1754 to 2679) | 275.2% (176.4 to 388.6) | 53.7 (45.5 to 63.7) | 37.5 (30.2 to 45.2) | -1.68 (-2.10 to -1.25) |
| **Kazakhstan** | 793 (683 to 920) | 1392 (1146 to 1635) | 75.4% (41.0 to 121.8) | 6.4 (5.5 to 7.5) | 8.5 (7.0 to 9.9) | -0.34 (-0.83 to 0.15) |
| **Kenya** | 1899 (1522 to 2375) | 6961 (5692 to 8487) | 266.6% (196.2 to 357.8) | 27.0 (21.6 to 33.4) | 36.8 (30.2 to 44.6) | 1.19 (1.08 to 1.30) |
| **Kiribati** | 48 (40 to 57) | 118 (92 to 154) | 145.9% (84.6 to 243.2) | 141.4 (117.4 to 166.6) | 184.3 (143.3 to 231.8) | 0.78 (0.63 to 0.93) |
| **Kuwait** | 100 (91 to 108) | 553 (456 to 659) | 453.3% (355.4 to 567.8) | 21.4 (19.0 to 23.2) | 25.1 (20.6 to 30.0) | 0.11 (-0.47 to 0.68) |
| **Kyrgyzstan** | 147 (133 to 162) | 356 (299 to 419) | 142.3% (98.0 to 191.1) | 5.0 (4.5 to 5.5) | 7.5 (6.3 to 8.8) | 0.28 (-0.18 to 0.75) |
| **Laos** | 706 (542 to 900) | 1485 (1145 to 1898) | 110.2% (52.3 to 194.5) | 36.1 (28.3 to 45.3) | 35.7 (27.9 to 44.6) | -0.18 (-0.25 to -0.12) |
| **Latvia** | 192 (178 to 206) | 546 (479 to 605) | 184.9% (149.5 to 222.3) | 5.3 (5.0 to 5.7) | 13.1 (11.4 to 14.5) | 2.54 (1.87 to 3.22) |
| **Lebanon** | 701 (584 to 848) | 1873 (1368 to 2337) | 167.1% (88.7 to 247.4) | 36.9 (30.9 to 44.1) | 29.2 (21.4 to 36.6) | -0.43 (-0.61 to -0.26) |
| **Lesotho** | 350 (286 to 440) | 946 (713 to 1199) | 170.6% (95.5 to 270.4) | 44.7 (36.8 to 56.7) | 96.8 (74.4 to 120.9) | 3.45 (2.91 to 3.99) |
| **Liberia** | 307 (246 to 371) | 766 (542 to 1048) | 149.5% (82.8 to 238.9) | 30.7 (25.2 to 36.7) | 42.8 (31.2 to 57.4) | 1.15 (1.01 to 1.30) |
| **Libya** | 228 (179 to 280) | 1128 (816 to 1505) | 395.3% (246.1 to 584.8) | 13.2 (10.3 to 16.1) | 24.2 (17.6 to 31.9) | 2.82 (2.53 to 3.11) |
| **Lithuania** | 148 (138 to 157) | 541 (473 to 609) | 265.3% (222.8 to 314.5) | 3.3 (3.1 to 3.5) | 9.0 (7.9 to 10.2) | 2.30 (1.28 to 3.34) |
| **Luxembourg** | 59 (55 to 62) | 70 (61 to 78) | 19.7% (5.4 to 34.2) | 10.9 (10.2 to 11.6) | 5.9 (5.2 to 6.6) | -1.90 (-2.05 to -1.75) |
| **Macedonia** | 352 (293 to 412) | 922 (720 to 1128) | 161.9% (97.1 to 244.6) | 20.4 (16.9 to 23.9) | 31.6 (25.3 to 38.3) | 1.50 (1.06 to 1.95) |
| **Madagascar** | 1525 (1231 to 1873) | 3134 (2303 to 4150) | 105.5% (54.0 to 175.4) | 34.3 (27.8 to 42.2) | 34.7 (25.9 to 45.8) | 0.03 (-0.02 to 0.08) |
| **Malawi** | 1423 (1189 to 1708) | 3011 (2445 to 3606) | 111.5% (68.1 to 169.3) | 43.7 (36.3 to 51.5) | 47.1 (38.8 to 55.9) | -0.00 (-0.25 to 0.25) |
| **Malaysia** | 2282 (2044 to 2557) | 5054 (4447 to 5649) | 121.5% (86.3 to 165.0) | 26.3 (23.5 to 29.5) | 19.2 (16.8 to 21.6) | -1.34 (-1.57 to -1.10) |
| **Maldives** | 27 (23 to 32) | 52 (43 to 62) | 92.6% (45.2 to 145.9) | 34.0 (29.1 to 39.2) | 17.9 (14.5 to 21.4) | -2.41 (-2.58 to -2.23) |
| **Mali** | 1071 (896 to 1298) | 3025 (2373 to 3798) | 182.4% (113.9 to 268.8) | 32.5 (27.2 to 39.2) | 41.5 (33.4 to 51.4) | 0.89 (0.80 to 0.98) |
| **Malta** | 99 (91 to 107) | 147 (124 to 166) | 48.2% (31.3 to 67.5) | 24.5 (22.4 to 26.5) | 13.2 (11.3 to 15.0) | -1.98 (-2.33 to -1.63) |
| **Marshall Islands** | 16 (13 to 20) | 52 (35 to 77) | 228.6% (123.2 to 343.2) | 107.7 (88.6 to 135.9) | 165.9 (112.7 to 236.1) | 1.34 (1.08 to 1.60) |
| **Mauritania** | 262 (206 to 319) | 685 (503 to 923) | 161.3% (93.7 to 257.7) | 29.9 (23.7 to 36.3) | 36.9 (27.4 to 49.5) | 0.45 (0.36 to 0.54) |
| **Mauritius** | 358 (340 to 377) | 1886 (1767 to 1976) | 426.5% (382.0 to 461.3) | 50.3 (47.6 to 52.9) | 104.0 (97.0 to 109.0) | 3.96 (2.99 to 4.94) |
| **Mexico** | 27976 (27120 to 28667) | 82750 (73597 to 92137) | 195.8% (164.7 to 229.6) | 71.1 (68.6 to 73.0) | 67.6 (60.2 to 75.1) | -0.30 (-0.56 to -0.03) |
| **Moldova** | 279 (258 to 301) | 524 (466 to 588) | 88.0% (65.6 to 115.2) | 6.4 (6.0 to 6.9) | 8.7 (7.8 to 9.8) | -0.06 (-0.94 to 0.83) |
| **Mongolia** | 55 (42 to 73) | 191 (150 to 238) | 245.3% (141.9 to 406.6) | 5.4 (4.1 to 7.0) | 8.5 (6.7 to 10.7) | 1.60 (1.47 to 1.73) |
| **Montenegro** | 70 (58 to 83) | 164 (135 to 194) | 135.9% (76.4 to 203.6) | 11.6 (9.7 to 13.7) | 17.6 (14.5 to 20.7) | 1.42 (1.25 to 1.59) |
| **Morocco** | 1529 (1257 to 1855) | 7421 (5524 to 8918) | 385.4% (253.6 to 515.2) | 11.7 (9.7 to 13.9) | 23.4 (17.4 to 28.2) | 2.69 (2.53 to 2.86) |
| **Mozambique** | 1957 (1657 to 2358) | 4810 (3604 to 5971) | 145.8% (81.7 to 225.5) | 38.2 (33.0 to 45.0) | 49.2 (37.7 to 60.7) | 1.32 (1.15 to 1.49) |
| **Myanmar** | 12430 (9861 to 15627) | 24764 (20057 to 30708) | 99.2% (43.9 to 178.5) | 57.7 (46.3 to 71.6) | 55.9 (45.7 to 69.3) | -0.36 (-0.45 to -0.27) |
| **Namibia** | 333 (279 to 409) | 848 (645 to 1076) | 154.8% (86.5 to 237.9) | 59.0 (49.5 to 71.5) | 71.9 (55.5 to 89.8) | 0.41 (0.10 to 0.73) |
| **Nepal** | 1613 (1285 to 1996) | 6308 (4807 to 8014) | 291.0% (179.1 to 450.2) | 20.9 (16.6 to 25.8) | 31.7 (24.5 to 40.5) | 1.48 (1.24 to 1.71) |
| **Netherlands** | 3607 (3238 to 3870) | 3064 (2588 to 3376) | -15.0% (-22.2 to -7.4) | 17.6 (15.8 to 18.9) | 7.7 (6.6 to 8.5) | -2.81 (-3.15 to -2.46) |
| **New Zealand** | 403 (371 to 431) | 646 (565 to 698) | 60.5% (45.7 to 74.6) | 10.3 (9.5 to 11.1) | 7.3 (6.4 to 7.8) | -1.99 (-2.45 to -1.52) |
| **Nicaragua** | 348 (314 to 383) | 1389 (1177 to 1676) | 298.7% (232.7 to 386.5) | 24.1 (21.7 to 26.4) | 29.8 (25.1 to 35.9) | 0.73 (0.50 to 0.96) |
| **Niger** | 488 (372 to 630) | 1806 (1306 to 2429) | 269.7% (176.6 to 392.3) | 21.4 (16.0 to 27.4) | 27.1 (20.3 to 36.0) | 0.80 (0.68 to 0.91) |
| **Nigeria** | 13218 (10889 to 15917) | 27553 (22319 to 33873) | 108.5% (56.9 to 180.6) | 34.6 (28.8 to 40.8) | 37.2 (30.8 to 44.5) | 0.17 (0.12 to 0.23) |
| **North Korea** | 1899 (1406 to 2438) | 4408 (3422 to 5653) | 132.1% (72.2 to 221.6) | 13.3 (10.0 to 16.9) | 13.8 (10.8 to 17.7) | 0.30 (0.14 to 0.47) |
| **Northern Mariana Islands** | 10 (8 to 12) | 28 (24 to 32) | 196.2% (126.4 to 279.5) | 66.3 (55.2 to 81.8) | 62.1 (54.6 to 69.2) | -0.22 (-0.56 to 0.12) |
| **Norway** | 488 (440 to 514) | 600 (514 to 649) | 22.9% (14.3 to 29.6) | 6.6 (6.0 to 6.9) | 5.1 (4.5 to 5.5) | -0.97 (-1.48 to -0.45) |
| **Oman** | 226 (171 to 315) | 684 (539 to 834) | 202.8% (91.5 to 347.8) | 39.1 (29.8 to 53.9) | 46.3 (37.1 to 56.1) | 1.04 (0.82 to 1.26) |
| **Pakistan** | 13443 (10799 to 16847) | 44901 (35800 to 55974) | 234.0% (169.1 to 319.3) | 27.3 (21.9 to 34.2) | 44.9 (35.7 to 55.5) | 1.35 (1.08 to 1.62) |
| **Palestine** | 399 (318 to 497) | 1056 (901 to 1213) | 164.5% (100.8 to 245.3) | 53.8 (43.1 to 66.1) | 54.4 (46.6 to 63.1) | 0.10 (-0.12 to 0.32) |
| **Panama** | 292 (273 to 310) | 1237 (988 to 1481) | 323.0% (236.9 to 407.6) | 20.5 (19.1 to 21.8) | 27.6 (22.0 to 33.0) | 0.73 (0.46 to 1.00) |
| **Papua New Guinea** | 1448 (1050 to 1895) | 4020 (3119 to 5134) | 177.6% (90.4 to 305.4) | 89.7 (67.6 to 115.1) | 89.2 (69.5 to 112.7) | -0.02 (-0.05 to 0.01) |
| **Paraguay** | 533 (455 to 608) | 3134 (2462 to 3903) | 488.0% (345.7 to 670.7) | 25.5 (21.7 to 29.1) | 56.5 (44.4 to 70.1) | 2.93 (2.59 to 3.28) |
| **Peru** | 1597 (1354 to 1856) | 5857 (4580 to 7520) | 266.7% (177.5 to 375.7) | 14.0 (11.9 to 16.4) | 17.6 (13.8 to 22.7) | 0.54 (0.26 to 0.81) |
| **Philippines** | 7351 (6657 to 8060) | 29878 (25571 to 34442) | 306.5% (242.5 to 380.8) | 28.0 (25.6 to 30.6) | 39.0 (33.5 to 44.7) | 1.26 (1.20 to 1.32) |
| **Poland** | 5467 (5236 to 5671) | 9772 (8711 to 10586) | 78.7% (62.5 to 94.4) | 12.4 (11.9 to 12.9) | 12.9 (11.6 to 14.0) | 0.43 (-0.00 to 0.86) |
| **Portugal** | 2987 (2778 to 3158) | 3903 (3290 to 4285) | 30.7% (15.0 to 44.7) | 21.8 (20.2 to 23.1) | 12.6 (10.9 to 13.8) | -2.22 (-2.56 to -1.87) |
| **Principality of Monaco** | 3 (2 to 4) | 4 (3 to 5) | 47.5% (16.5 to 98.0) | 3.6 (2.8 to 4.4) | 3.6 (2.9 to 4.3) | 0.13 (0.09 to 0.16) |
| **Puerto Rico** | 1839 (1733 to 1930) | 3140 (2607 to 3679) | 70.8% (41.6 to 101.8) | 53.0 (49.8 to 55.6) | 39.7 (33.1 to 46.8) | -1.28 (-1.54 to -1.02) |
| **Qatar** | 55 (43 to 68) | 374 (286 to 486) | 581.0% (358.4 to 860.3) | 85.0 (69.3 to 104.4) | 76.8 (60.8 to 95.5) | -0.44 (-1.28 to 0.41) |
| **Republic of Nauru** | 5 (4 to 6) | 8 (6 to 10) | 58.1% (17.6 to 114.9) | 118.1 (93.7 to 146.4) | 146.9 (118.1 to 184.6) | 0.57 (0.41 to 0.73) |
| **Republic of Niue** | 2 (2 to 2) | 3 (2 to 3) | 35.0% (2.7 to 78.8) | 83.1 (68.7 to 100.4) | 121.8 (93.5 to 155.1) | 1.13 (0.97 to 1.29) |
| **Republic of Palau** | 8 (6 to 9) | 21 (16 to 26) | 174.3% (100.8 to 283.0) | 84.4 (67.7 to 102.3) | 106.4 (85.5 to 131.4) | 0.88 (0.73 to 1.04) |
| **Republic of San Marino** | 3 (2 to 3) | 4 (2 to 5) | 24.7% (-9.7 to 72.1) | 7.8 (6.6 to 9.1) | 3.7 (2.5 to 5.1) | -1.56 (-1.89 to -1.23) |
| **Romania** | 1912 (1787 to 2068) | 2669 (2377 to 3001) | 39.6% (23.2 to 60.0) | 7.0 (6.6 to 7.6) | 6.8 (6.1 to 7.6) | -0.04 (-0.41 to 0.33) |
| **Russian Federation** | 6719 (6500 to 6882) | 38282 (35246 to 41263) | 469.7% (427.7 to 513.0) | 3.7 (3.6 to 3.8) | 15.6 (14.4 to 16.9) | 3.73 (2.15 to 5.33) |
| **Rwanda** | 1406 (1013 to 1882) | 2134 (1329 to 3089) | 51.7% (9.1 to 111.0) | 56.9 (41.3 to 75.3) | 41.6 (26.6 to 58.5) | -1.94 (-2.28 to -1.60) |
| **Saint Kitts and Nevis** | 27 (26 to 29) | 30 (25 to 35) | 10.4% (-9.5 to 30.0) | 72.9 (68.0 to 77.7) | 51.6 (43.4 to 59.1) | -0.70 (-1.00 to -0.40) |
| **Saint Lucia** | 78 (74 to 82) | 133 (111 to 157) | 71.6% (42.9 to 103.2) | 97.4 (92.4 to 102.7) | 57.1 (47.8 to 67.2) | -2.73 (-3.05 to -2.41) |
| **Saint Vincent and the Grenadines** | 67 (61 to 71) | 94 (84 to 106) | 41.8% (24.0 to 63.6) | 98.0 (90.5 to 104.2) | 70.0 (62.4 to 78.1) | -1.49 (-1.77 to -1.21) |
| **Samoa** | 59 (49 to 71) | 124 (99 to 155) | 110.0% (66.3 to 174.8) | 75.8 (63.8 to 90.8) | 92.4 (74.4 to 114.9) | 0.57 (0.49 to 0.65) |
| **Sao Tome and Principe** | 7 (6 to 9) | 17 (14 to 20) | 127.6% (79.8 to 183.6) | 12.5 (10.9 to 14.4) | 17.9 (15.3 to 21.0) | 1.24 (1.11 to 1.37) |
| **Saudi Arabia** | 1188 (884 to 1599) | 4937 (3840 to 6104) | 315.4% (161.6 to 514.5) | 23.2 (17.4 to 30.8) | 29.3 (23.3 to 35.3) | 0.50 (0.35 to 0.65) |
| **Senegal** | 869 (720 to 1044) | 3024 (2329 to 3886) | 248.0% (166.1 to 358.0) | 30.8 (25.6 to 36.9) | 45.6 (35.6 to 58.1) | 1.26 (1.13 to 1.39) |
| **Serbia** | 1950 (1707 to 2249) | 3714 (3115 to 4454) | 90.4% (49.5 to 137.5) | 20.9 (18.1 to 24.1) | 21.2 (17.8 to 25.3) | -0.10 (-0.25 to 0.05) |
| **Seychelles** | 9 (8 to 10) | 26 (22 to 29) | 182.3% (137.4 to 237.0) | 16.2 (14.4 to 17.9) | 24.1 (21.1 to 27.8) | 1.64 (1.43 to 1.85) |
| **Sierra Leone** | 437 (343 to 537) | 1059 (816 to 1386) | 142.6% (83.9 to 225.8) | 23.8 (19.2 to 29.1) | 32.4 (25.6 to 41.9) | 1.17 (0.93 to 1.41) |
| **Singapore** | 352 (329 to 375) | 164 (144 to 180) | -53.4% (-58.7 to -48.8) | 17.6 (16.4 to 18.8) | 2.0 (1.7 to 2.2) | -7.04 (-8.10 to -5.97) |
| **Slovakia** | 677 (589 to 777) | 821 (662 to 987) | 21.4% (-4.5 to 50.6) | 11.3 (9.8 to 12.9) | 8.4 (6.8 to 10.1) | -0.89 (-0.97 to -0.82) |
| **Slovenia** | 260 (240 to 283) | 409 (351 to 470) | 57.5% (35.3 to 81.8) | 10.5 (9.7 to 11.4) | 8.2 (7.1 to 9.5) | -2.70 (-3.35 to -2.05) |
| **Solomon Islands** | 105 (69 to 148) | 362 (283 to 465) | 245.5% (141.3 to 424.3) | 84.3 (60.6 to 113.3) | 111.6 (89.2 to 139.1) | 0.95 (0.88 to 1.02) |
| **Somalia** | 1038 (781 to 1346) | 2716 (2001 to 3403) | 161.8% (94.5 to 251.9) | 51.3 (40.0 to 63.9) | 53.7 (40.6 to 65.5) | 0.22 (0.13 to 0.32) |
| **South Africa** | 8178 (7377 to 8908) | 32185 (29911 to 34448) | 293.6% (255.6 to 334.3) | 42.7 (38.4 to 46.6) | 77.3 (71.8 to 82.8) | 2.24 (1.77 to 2.72) |
| **South Korea** | 5553 (5038 to 6075) | 10552 (8750 to 12090) | 90.0% (58.8 to 125.3) | 20.7 (18.7 to 22.8) | 11.4 (9.4 to 13.0) | -2.50 (-3.12 to -1.88) |
| **South Sudan** | 1045 (757 to 1414) | 1834 (1353 to 2497) | 75.5% (30.8 to 149.2) | 45.8 (33.3 to 61.1) | 56.2 (41.9 to 76.0) | 0.58 (0.45 to 0.71) |
| **Spain** | 9723 (8699 to 10351) | 9088 (7448 to 10099) | -6.5% (-17.9 to 2.5) | 17.7 (15.7 to 18.8) | 7.0 (5.8 to 7.7) | -3.06 (-3.19 to -2.92) |
| **Sri Lanka** | 3370 (2906 to 3794) | 11953 (8316 to 15936) | 254.7% (140.0 to 399.1) | 38.0 (32.8 to 42.7) | 47.0 (33.1 to 62.1) | 1.50 (1.17 to 1.84) |
| **Sudan** | 1012 (802 to 1353) | 3109 (2335 to 4120) | 207.1% (120.7 to 320.4) | 12.0 (9.6 to 15.9) | 17.9 (13.8 to 23.4) | 1.52 (1.35 to 1.70) |
| **Suriname** | 95 (86 to 104) | 259 (202 to 323) | 171.1% (107.7 to 245.4) | 39.3 (35.6 to 43.0) | 41.7 (32.6 to 52.1) | 0.43 (0.24 to 0.62) |
| **Swaziland** | 197 (156 to 238) | 585 (433 to 787) | 197.5% (119.7 to 315.5) | 79.9 (64.6 to 97.5) | 119.1 (90.5 to 154.3) | 1.73 (1.08 to 2.39) |
| **Sweden** | 1433 (1278 to 1537) | 1921 (1603 to 2194) | 34.0% (19.2 to 50.8) | 8.7 (7.8 to 9.3) | 7.3 (6.1 to 8.2) | -0.45 (-0.72 to -0.19) |
| **Switzerland** | 1552 (1382 to 1670) | 1181 (970 to 1307) | -23.9% (-31.5 to -16.8) | 13.7 (12.2 to 14.7) | 5.2 (4.4 to 5.7) | -3.45 (-3.68 to -3.21) |
| **Syria** | 830 (673 to 1008) | 2160 (1608 to 2843) | 160.1% (81.5 to 291.6) | 18.1 (14.8 to 21.9) | 19.8 (15.0 to 25.7) | -0.17 (-0.39 to 0.05) |
| **Taiwan (Province of China)** | 4919 (4651 to 5140) | 10146 (8862 to 11027) | 106.3% (84.6 to 126.3) | 34.7 (32.3 to 36.3) | 23.2 (20.4 to 25.2) | -2.30 (-2.82 to -1.77) |
| **Tajikistan** | 322 (273 to 384) | 773 (586 to 993) | 139.7% (73.9 to 235.7) | 11.8 (10.1 to 13.9) | 13.6 (10.3 to 17.3) | 0.16 (-0.18 to 0.50) |
| **Tanzania** | 3412 (2876 to 4012) | 8272 (6533 to 10594) | 142.4% (83.2 to 222.3) | 36.8 (31.3 to 42.7) | 37.5 (30.1 to 47.3) | -0.14 (-0.23 to -0.04) |
| **Thailand** | 7750 (6325 to 9327) | 24159 (18261 to 30253) | 211.7% (125.2 to 328.6) | 23.6 (19.3 to 28.6) | 22.3 (16.9 to 27.9) | -0.83 (-1.07 to -0.59) |
| **The Bahamas** | 70 (65 to 75) | 135 (109 to 166) | 92.1% (53.3 to 139.7) | 48.0 (44.2 to 51.5) | 35.7 (29.3 to 43.5) | -1.42 (-1.67 to -1.18) |
| **The Gambia** | 79 (59 to 101) | 369 (277 to 473) | 366.4% (245.0 to 527.7) | 26.8 (20.8 to 33.7) | 43.4 (32.8 to 55.2) | 1.48 (1.34 to 1.63) |
| **Timor-Leste** | 45 (34 to 59) | 176 (134 to 233) | 287.9% (179.5 to 433.3) | 18.5 (13.8 to 23.9) | 22.1 (17.0 to 29.0) | 0.77 (0.58 to 0.97) |
| **Togo** | 252 (205 to 310) | 1088 (806 to 1461) | 331.4% (223.9 to 480.6) | 24.2 (19.5 to 29.8) | 35.2 (27.2 to 45.7) | 1.18 (0.98 to 1.38) |
| **Tokelau** | 1 (1 to 1) | 1 (1 to 2) | 26.5% (-5.4 to 63.0) | 75.9 (59.2 to 95.9) | 83.8 (66.8 to 104.6) | 0.25 (0.12 to 0.38) |
| **Tonga** | 47 (39 to 54) | 87 (70 to 108) | 85.4% (38.2 to 146.9) | 90.9 (76.1 to 105.9) | 111.3 (89.6 to 138.1) | 0.66 (0.51 to 0.81) |
| **Trinidad and Tobago** | 1011 (964 to 1061) | 1833 (1420 to 2279) | 81.3% (39.2 to 125.1) | 129.2 (123.5 to 135.4) | 95.7 (74.3 to 118.7) | -1.26 (-1.44 to -1.09) |
| **Tunisia** | 442 (368 to 526) | 2046 (1479 to 2778) | 363.1% (221.8 to 533.3) | 10.4 (8.7 to 12.2) | 16.5 (12.1 to 22.2) | 1.66 (1.55 to 1.78) |
| **Turkey** | 10643 (9116 to 12625) | 22751 (18275 to 27523) | 113.8% (63.2 to 174.8) | 35.8 (30.7 to 42.2) | 26.3 (21.3 to 32.0) | -0.59 (-0.97 to -0.21) |
| **Turkmenistan** | 180 (159 to 204) | 844 (649 to 1104) | 367.8% (251.6 to 513.8) | 9.5 (8.4 to 10.7) | 20.5 (15.8 to 26.5) | 1.77 (1.37 to 2.17) |
| **Tuvalu** | 5 (4 to 7) | 10 (8 to 12) | 80.8% (43.4 to 124.6) | 86.9 (71.2 to 108.6) | 100.2 (81.5 to 124.0) | 0.48 (0.38 to 0.58) |
| **Uganda** | 2254 (1567 to 3573) | 5680 (4027 to 8306) | 152.0% (79.8 to 247.9) | 40.9 (28.6 to 63.8) | 46.1 (33.0 to 64.5) | -0.03 (-0.26 to 0.20) |
| **Ukraine** | 2432 (2254 to 2595) | 2245 (1669 to 2861) | -7.7% (-30.8 to 17.5) | 3.3 (3.1 to 3.6) | 2.9 (2.1 to 3.7) | -1.99 (-2.51 to -1.47) |
| **United Arab Emirates** | 136 (103 to 187) | 807 (606 to 1012) | 495.2% (267.5 to 667.2) | 41.8 (32.1 to 55.3) | 42.8 (33.2 to 51.9) | 1.97 (1.28 to 2.67) |
| **United Kingdom** | 8264 (7724 to 8548) | 6182 (5408 to 6579) | -25.2% (-30.1 to -22.3) | 8.7 (8.1 to 9.0) | 4.1 (3.7 to 4.4) | -2.45 (-2.75 to -2.14) |
| **United States** | 46688 (42889 to 48885) | 70711 (64156 to 75054) | 51.5% (45.9 to 57.0) | 14.3 (13.2 to 14.9) | 11.8 (10.8 to 12.5) | -1.30 (-1.75 to -0.86) |
| **Uruguay** | 652 (609 to 694) | 952 (848 to 1023) | 46.0% (32.6 to 59.5) | 16.4 (15.3 to 17.4) | 15.7 (14.2 to 16.8) | -0.24 (-0.31 to -0.17) |
| **Uzbekistan** | 1044 (969 to 1130) | 6320 (5297 to 7409) | 505.5% (405.0 to 622.5) | 9.1 (8.4 to 9.8) | 24.0 (20.3 to 28.1) | 2.87 (2.38 to 3.37) |
| **Vanuatu** | 40 (30 to 59) | 140 (113 to 180) | 254.1% (160.6 to 388.1) | 71.5 (55.2 to 102.1) | 89.4 (73.0 to 111.6) | 0.65 (0.59 to 0.71) |
| **Venezuela** | 3061 (2885 to 3209) | 12398 (9626 to 15802) | 305.0% (212.3 to 410.8) | 33.8 (31.6 to 35.5) | 43.0 (33.5 to 54.7) | 0.58 (0.25 to 0.92) |
| **Vietnam** | 11034 (8870 to 13715) | 30787 (25035 to 37472) | 179.0% (109.4 to 274.4) | 29.8 (24.2 to 36.8) | 35.6 (29.0 to 42.7) | 0.70 (0.65 to 0.76) |
| **Virgin Islands, U.S.** | 32 (27 to 37) | 51 (40 to 62) | 60.1% (17.8 to 112.4) | 43.7 (37.5 to 51.0) | 28.3 (22.7 to 34.8) | -1.42 (-1.59 to -1.24) |
| **Yemen** | 501 (365 to 723) | 1749 (1207 to 2667) | 249.2% (150.8 to 404.0) | 11.8 (8.9 to 16.9) | 14.4 (10.2 to 21.5) | 0.76 (0.64 to 0.89) |
| **Zambia** | 1174 (984 to 1411) | 2737 (2043 to 3518) | 133.1% (69.4 to 214.9) | 47.6 (40.2 to 57.3) | 46.1 (35.7 to 57.7) | -0.54 (-0.76 to -0.33) |
| **Zimbabwe** | 1284 (1054 to 1527) | 3918 (3047 to 4931) | 205.1% (121.7 to 319.2) | 37.1 (30.6 to 43.9) | 65.7 (51.7 to 81.4) | 2.49 (1.90 to 3.09) |

ASMR, age-standardized mortality rate; UI, uncertainty interval; EAPC, estimated annual percentage change; CI, confidence interval.

# Supplementary Table 9. The DALYs cases and age-standardized DALYs rate of type 2 diabetes mellitus in 1990 and 2021, and its temporal trends from 1990 to 2021 in 204 countries/territories.

| **Countries /territories** | **Number of DALYs cases in 1990**  **(95%UI)** | **Number of DALYs cases in 2021**  **(95%UI)** | **Percentage change in DALYs counts, 1990-2021**  **(95%UI)** | **1990 ASDR per 100000**  **(95%UI)** | **2021 ASDR per 100000**  **(95%UI)** | **1990-2021 EAPC (%)**  **(95%CI)** |
| --- | --- | --- | --- | --- | --- | --- |
| **Afghanistan** | 71486 (57331 to 90215) | 240498 (183885 to 313326) | 236.4% (169.6 to 297.0) | 989.9 (797.4 to 1236.2) | 2024.0 (1591.7 to 2589.4) | 2.44 (2.40 to 2.49) |
| **Albania** | 6257 (4733 to 8209) | 16537 (12085 to 22093) | 164.3% (136.3 to 192.7) | 296.0 (226.1 to 382.9) | 389.4 (283.6 to 519.4) | 0.96 (0.87 to 1.05) |
| **Algeria** | 66896 (52939 to 85746) | 419929 (317651 to 546950) | 527.7% (438.2 to 600.4) | 535.6 (431.6 to 674.0) | 1121.5 (858.8 to 1447.5) | 2.56 (2.49 to 2.64) |
| **American Samoa** | 715 (611 to 820) | 2161 (1804 to 2640) | 202.2% (153.2 to 263.6) | 2872.1 (2473.5 to 3268.7) | 4290.6 (3621.8 to 5185.5) | 1.18 (0.91 to 1.46) |
| **Andorra** | 210 (166 to 261) | 661 (518 to 864) | 215.3% (150.9 to 295.1) | 364.6 (290.5 to 450.4) | 448.8 (348.4 to 591.6) | 0.75 (0.60 to 0.90) |
| **Angola** | 59454 (48557 to 74014) | 217296 (174411 to 269223) | 265.5% (176.6 to 370.5) | 1397.5 (1153.5 to 1725.8) | 1607.9 (1317.2 to 1972.6) | 0.37 (0.30 to 0.44) |
| **Antigua and Barbuda** | 1017 (921 to 1145) | 2278 (1909 to 2740) | 124.0% (101.5 to 145.2) | 1937.0 (1753.0 to 2187.1) | 2100.3 (1771.3 to 2515.8) | -0.05 (-0.17 to 0.06) |
| **Argentina** | 218600 (196620 to 246039) | 406389 (335178 to 497737) | 85.9% (68.3 to 105.4) | 673.6 (607.0 to 758.6) | 738.4 (607.1 to 905.8) | 0.04 (-0.11 to 0.20) |
| **Armenia** | 18626 (16225 to 21842) | 29964 (24200 to 36738) | 60.9% (43.9 to 77.2) | 655.5 (572.7 to 766.8) | 707.6 (572.5 to 875.2) | -0.13 (-0.81 to 0.55) |
| **Australia** | 67910 (58928 to 79547) | 177576 (142602 to 223642) | 161.5% (135.1 to 190.5) | 348.8 (302.8 to 409.3) | 412.2 (329.5 to 518.4) | 0.33 (0.23 to 0.42) |
| **Austria** | 41335 (37439 to 45650) | 61651 (51051 to 74918) | 49.1% (32.4 to 66.1) | 349.4 (315.8 to 389.9) | 359.6 (290.5 to 446.3) | 0.50 (0.16 to 0.84) |
| **Azerbaijan** | 23106 (19035 to 28057) | 89179 (68781 to 113849) | 286.0% (199.7 to 369.0) | 434.1 (358.8 to 526.5) | 796.9 (619.7 to 1012.1) | 1.69 (1.39 to 1.98) |
| **Bahrain** | 4424 (3861 to 5017) | 31203 (25684 to 37954) | 605.3% (489.2 to 718.5) | 2585.2 (2277.0 to 2907.1) | 3396.3 (2879.8 to 4009.2) | 0.59 (0.34 to 0.84) |
| **Bangladesh** | 372495 (315851 to 433835) | 1532109 (1237051 to 1907809) | 311.3% (249.8 to 382.6) | 762.5 (656.8 to 872.2) | 1112.4 (915.8 to 1376.3) | 1.16 (0.94 to 1.37) |
| **Barbados** | 5867 (5303 to 6557) | 10158 (8444 to 12343) | 73.1% (49.1 to 99.0) | 2068.6 (1870.7 to 2314.6) | 2041.6 (1693.9 to 2480.7) | -0.28 (-0.41 to -0.14) |
| **Belarus** | 28099 (22649 to 34770) | 46650 (35949 to 60405) | 66.0% (50.0 to 81.3) | 217.5 (175.3 to 270.6) | 307.0 (237.1 to 397.0) | 0.34 (0.04 to 0.65) |
| **Belgium** | 55190 (46723 to 65086) | 90800 (67872 to 121192) | 64.5% (43.7 to 86.4) | 371.0 (311.2 to 444.7) | 453.0 (334.2 to 619.1) | 0.47 (0.38 to 0.55) |
| **Belize** | 1390 (1264 to 1564) | 6450 (5501 to 7740) | 363.9% (320.1 to 410.1) | 1449.8 (1317.8 to 1629.1) | 2008.7 (1719.2 to 2382.3) | 1.00 (0.59 to 1.40) |
| **Benin** | 17529 (15003 to 20504) | 77051 (61453 to 96110) | 339.6% (256.8 to 420.9) | 838.6 (719.2 to 976.0) | 1313.2 (1062.0 to 1590.9) | 1.36 (1.27 to 1.45) |
| **Bermuda** | 632 (564 to 720) | 1107 (886 to 1394) | 75.1% (50.8 to 99.8) | 1011.1 (906.8 to 1147.1) | 885.7 (702.9 to 1128.0) | -0.54 (-0.68 to -0.39) |
| **Bhutan** | 1838 (1488 to 2273) | 6264 (5059 to 7812) | 240.7% (179.1 to 317.4) | 704.1 (565.4 to 866.1) | 1005.2 (811.4 to 1246.0) | 1.15 (1.12 to 1.19) |
| **Bolivia** | 37899 (31919 to 44526) | 133816 (108268 to 166331) | 253.1% (189.4 to 337.8) | 1140.3 (963.4 to 1340.1) | 1429.4 (1163.5 to 1786.1) | 0.71 (0.68 to 0.73) |
| **Bosnia and Herzegovina** | 24452 (20062 to 29601) | 72952 (58697 to 89807) | 198.3% (158.3 to 244.5) | 589.9 (484.8 to 712.7) | 1176.8 (942.3 to 1453.0) | 2.63 (2.39 to 2.87) |
| **Botswana** | 7987 (6257 to 10158) | 24377 (20133 to 29714) | 205.2% (127.1 to 309.8) | 1425.2 (1126.9 to 1799.6) | 1655.7 (1397.3 to 1995.2) | 0.68 (0.45 to 0.92) |
| **Brazil** | 991855 (883523 to 1114214) | 2593542 (2202320 to 3054119) | 161.5% (147.9 to 173.9) | 1056.6 (944.4 to 1183.2) | 1023.6 (870.9 to 1203.7) | -0.14 (-0.18 to -0.09) |
| **Brunei** | 2299 (2005 to 2613) | 7967 (6497 to 9815) | 246.6% (188.6 to 309.9) | 2149.4 (1870.5 to 2449.5) | 2059.1 (1717.6 to 2509.8) | -0.15 (-0.20 to -0.10) |
| **Bulgaria** | 77276 (64964 to 91544) | 107793 (87539 to 136309) | 39.5% (27.2 to 54.2) | 616.8 (519.9 to 730.5) | 803.5 (644.7 to 1012.0) | 0.62 (0.45 to 0.79) |
| **Burkina Faso** | 40306 (34053 to 47730) | 111241 (89591 to 137811) | 176.0% (118.9 to 242.4) | 913.0 (788.1 to 1079.1) | 1092.2 (893.0 to 1337.9) | 0.57 (0.48 to 0.65) |
| **Burundi** | 32429 (25059 to 41317) | 62733 (46687 to 87611) | 93.4% (48.3 to 153.4) | 1336.6 (1038.9 to 1707.2) | 1194.0 (885.1 to 1644.5) | -0.82 (-0.99 to -0.66) |
| **Cambodia** | 40741 (33300 to 49062) | 148868 (117436 to 186392) | 265.4% (195.1 to 356.3) | 840.9 (693.1 to 1010.1) | 1160.0 (918.3 to 1451.5) | 1.02 (0.90 to 1.14) |
| **Cameroon** | 47021 (38508 to 56550) | 210505 (160408 to 268193) | 347.7% (254.2 to 461.1) | 1016.0 (833.1 to 1215.7) | 1507.3 (1177.3 to 1916.3) | 1.17 (0.94 to 1.39) |
| **Canada** | 115716 (101795 to 134121) | 381588 (290034 to 499360) | 229.8% (183.1 to 278.0) | 356.1 (313.6 to 412.6) | 557.1 (421.3 to 734.7) | 0.79 (0.54 to 1.05) |
| **Cape Verde** | 1105 (916 to 1369) | 5974 (4825 to 7410) | 440.6% (375.1 to 518.9) | 479.0 (396.8 to 592.5) | 1295.6 (1053.8 to 1611.5) | 3.01 (2.70 to 3.33) |
| **Central African Republic** | 22206 (18757 to 25737) | 53647 (42546 to 66806) | 141.6% (95.4 to 197.9) | 1773.2 (1510.8 to 2059.4) | 2058.1 (1663.7 to 2515.9) | 0.46 (0.39 to 0.53) |
| **Chad** | 20034 (16322 to 24501) | 76996 (61731 to 95946) | 284.3% (208.7 to 367.0) | 688.4 (560.4 to 836.6) | 1182.7 (955.1 to 1460.8) | 1.69 (1.43 to 1.96) |
| **Chile** | 55595 (47599 to 64036) | 177740 (137204 to 228388) | 219.7% (181.2 to 258.7) | 545.5 (468.5 to 626.5) | 697.8 (537.5 to 898.5) | 0.84 (0.65 to 1.02) |
| **China** | 3960057 (3188322 to 4872252) | 11465018 (8834151 to 14700712) | 189.5% (162.1 to 219.4) | 438.7 (358.5 to 531.5) | 569.8 (435.4 to 734.2) | 0.69 (0.54 to 0.84) |
| **Colombia** | 161056 (134335 to 194962) | 456037 (357263 to 580067) | 183.2% (155.3 to 211.8) | 839.1 (707.8 to 1006.5) | 826.6 (647.1 to 1050.8) | -0.76 (-0.94 to -0.57) |
| **Comoros** | 2378 (1820 to 3033) | 6610 (5028 to 8301) | 178.0% (113.9 to 265.1) | 1162.4 (918.4 to 1472.2) | 1302.4 (994.2 to 1621.2) | 0.22 (0.11 to 0.33) |
| **Congo** | 19766 (16188 to 23358) | 57384 (46213 to 71677) | 190.3% (132.6 to 264.9) | 1759.0 (1458.8 to 2056.7) | 1907.3 (1563.2 to 2332.6) | 0.05 (-0.06 to 0.16) |
| **Cook Islands** | 532 (456 to 619) | 997 (837 to 1209) | 87.5% (52.7 to 125.6) | 4102.9 (3539.1 to 4737.2) | 4005.2 (3321.4 to 4852.4) | -0.24 (-0.32 to -0.17) |
| **Costa Rica** | 13221 (10800 to 16241) | 58276 (46023 to 74133) | 340.8% (308.6 to 371.9) | 712.9 (586.4 to 871.0) | 1061.0 (838.6 to 1349.8) | 0.98 (0.75 to 1.21) |
| **Cote d' Ivoire** | 39866 (33588 to 47750) | 169007 (134991 to 211091) | 323.9% (237.7 to 417.6) | 908.3 (771.8 to 1073.6) | 1343.1 (1096.4 to 1660.5) | 1.17 (0.98 to 1.37) |
| **Croatia** | 32345 (26546 to 39400) | 60353 (47805 to 75694) | 86.6% (72.7 to 101.3) | 533.1 (437.9 to 645.5) | 702.3 (552.5 to 889.5) | 0.57 (0.42 to 0.72) |
| **Cuba** | 82838 (71300 to 97515) | 143768 (110186 to 187122) | 73.6% (50.9 to 93.3) | 804.6 (693.6 to 945.4) | 783.6 (596.9 to 1017.9) | -0.25 (-0.57 to 0.07) |
| **Cyprus** | 10231 (8878 to 11733) | 15988 (13104 to 19646) | 56.3% (33.6 to 80.7) | 1436.7 (1265.7 to 1622.9) | 809.8 (668.7 to 995.6) | -2.18 (-2.30 to -2.06) |
| **Czech Republic** | 74727 (62007 to 90864) | 163785 (131956 to 202069) | 119.2% (99.8 to 140.8) | 544.6 (451.2 to 664.5) | 787.1 (626.5 to 984.3) | 2.00 (1.67 to 2.34) |
| **Democratic Republic of the Congo** | 214759 (172019 to 261920) | 611775 (481699 to 766855) | 184.9% (117.9 to 262.2) | 1317.8 (1068.7 to 1589.5) | 1505.7 (1196.2 to 1882.0) | 0.39 (0.30 to 0.48) |
| **Denmark** | 22049 (19732 to 24858) | 42482 (35493 to 51155) | 92.7% (74.6 to 109.3) | 288.6 (256.0 to 328.1) | 390.7 (318.2 to 480.3) | 0.98 (0.69 to 1.27) |
| **Djibouti** | 1311 (1009 to 1699) | 8348 (6501 to 10987) | 536.5% (375.3 to 751.7) | 901.1 (707.3 to 1161.8) | 1234.2 (970.9 to 1583.0) | 0.98 (0.87 to 1.08) |
| **Dominica** | 1201 (1077 to 1362) | 2090 (1744 to 2521) | 74.0% (53.7 to 98.6) | 2039.6 (1828.9 to 2314.0) | 2503.6 (2096.3 to 3009.4) | 0.45 (0.38 to 0.52) |
| **Dominican Republic** | 34872 (29495 to 42782) | 154287 (122213 to 192885) | 342.4% (281.6 to 407.6) | 874.1 (740.6 to 1061.1) | 1496.2 (1185.5 to 1864.3) | 1.99 (1.91 to 2.07) |
| **Ecuador** | 42948 (37789 to 49682) | 204310 (162354 to 249064) | 375.7% (312.1 to 439.6) | 778.0 (688.8 to 893.2) | 1235.4 (983.3 to 1503.5) | 1.31 (1.01 to 1.61) |
| **Egypt** | 224945 (201947 to 250173) | 1162517 (960429 to 1432858) | 416.8% (340.7 to 514.1) | 793.5 (717.6 to 874.6) | 1783.0 (1511.2 to 2164.1) | 3.22 (2.99 to 3.45) |
| **El Salvador** | 25158 (21737 to 28774) | 97260 (81119 to 116945) | 286.6% (228.7 to 354.0) | 803.5 (696.1 to 916.3) | 1591.9 (1327.2 to 1916.7) | 2.08 (1.92 to 2.23) |
| **Equatorial Guinea** | 3176 (2539 to 3878) | 11526 (8726 to 14903) | 262.9% (162.8 to 391.6) | 1541.5 (1245.5 to 1852.4) | 1950.8 (1497.7 to 2493.0) | 0.83 (0.58 to 1.09) |
| **Eritrea** | 16988 (13512 to 21563) | 46774 (36231 to 59814) | 175.3% (117.3 to 242.1) | 1334.4 (1078.0 to 1664.8) | 1525.4 (1194.7 to 1914.1) | 0.43 (0.38 to 0.48) |
| **Estonia** | 5105 (4059 to 6416) | 13394 (10885 to 16737) | 162.4% (142.9 to 186.7) | 252.9 (200.4 to 318.9) | 560.5 (453.1 to 717.2) | 2.20 (1.93 to 2.47) |
| **Ethiopia** | 375593 (323482 to 439120) | 498851 (421662 to 583405) | 32.8% (7.2 to 59.6) | 1753.7 (1525.4 to 2017.6) | 1061.5 (907.6 to 1224.1) | -2.09 (-2.29 to -1.89) |
| **Federated States of Micronesia** | 1340 (1089 to 1666) | 3202 (2548 to 3965) | 138.9% (87.5 to 206.6) | 2610.3 (2133.6 to 3252.3) | 3912.0 (3172.5 to 4779.1) | 1.31 (1.09 to 1.54) |
| **Fiji** | 20729 (17701 to 24280) | 59130 (47437 to 73700) | 185.3% (122.4 to 269.6) | 5235.9 (4493.2 to 6114.0) | 7322.7 (5942.6 to 9076.0) | 0.94 (0.75 to 1.13) |
| **Finland** | 21393 (17082 to 26971) | 46905 (34539 to 62510) | 119.2% (99.5 to 137.8) | 311.2 (245.9 to 396.4) | 462.6 (336.1 to 622.4) | 1.22 (1.05 to 1.39) |
| **France** | 206476 (181497 to 237254) | 402067 (330335 to 496040) | 94.7% (77.4 to 113.2) | 253.3 (221.1 to 293.2) | 318.8 (253.7 to 403.5) | 0.82 (0.53 to 1.12) |
| **Gabon** | 9917 (8009 to 12339) | 24786 (19771 to 31098) | 149.9% (97.0 to 216.2) | 1709.1 (1381.0 to 2124.7) | 2219.5 (1793.9 to 2786.2) | 0.80 (0.68 to 0.92) |
| **Georgia** | 27501 (23246 to 33098) | 46756 (37883 to 57477) | 70.0% (51.0 to 88.3) | 432.4 (365.0 to 520.6) | 836.0 (674.0 to 1033.8) | 2.74 (2.33 to 3.15) |
| **Germany** | 472295 (426607 to 528909) | 756572 (619470 to 931564) | 60.2% (41.6 to 78.6) | 373.0 (335.8 to 422.2) | 439.6 (351.6 to 547.2) | 0.22 (-0.06 to 0.50) |
| **Ghana** | 53803 (44737 to 65395) | 267217 (214713 to 326854) | 396.7% (285.0 to 517.1) | 796.4 (667.6 to 966.5) | 1465.0 (1186.0 to 1793.8) | 2.41 (2.11 to 2.71) |
| **Greece** | 51999 (41566 to 65345) | 97776 (74899 to 128308) | 88.0% (76.0 to 101.5) | 355.9 (282.2 to 449.6) | 503.5 (375.7 to 677.4) | 0.93 (0.82 to 1.05) |
| **Greenland** | 134 (118 to 154) | 326 (262 to 408) | 142.5% (97.3 to 199.7) | 379.6 (335.2 to 429.5) | 455.0 (368.7 to 563.3) | 0.44 (0.23 to 0.66) |
| **Grenada** | 1679 (1504 to 1853) | 3169 (2678 to 3744) | 88.8% (67.3 to 111.1) | 2398.3 (2137.8 to 2667.1) | 2742.2 (2337.6 to 3206.3) | 0.35 (0.16 to 0.54) |
| **Guam** | 1053 (923 to 1209) | 2421 (1961 to 2994) | 129.8% (102.3 to 158.2) | 1281.4 (1142.0 to 1447.0) | 1194.4 (964.7 to 1472.3) | -0.11 (-0.27 to 0.05) |
| **Guatemala** | 28214 (23987 to 33417) | 271646 (232104 to 319451) | 862.8% (756.6 to 985.2) | 732.6 (630.6 to 858.8) | 2331.6 (2001.8 to 2729.7) | 3.07 (2.67 to 3.47) |
| **Guinea** | 26839 (20995 to 32732) | 74785 (60165 to 92017) | 178.6% (121.7 to 261.1) | 795.8 (624.6 to 977.2) | 1230.1 (1003.0 to 1516.7) | 1.44 (1.28 to 1.60) |
| **Guinea-Bissau** | 5254 (4316 to 6353) | 14078 (11380 to 17332) | 168.0% (114.0 to 235.2) | 1216.5 (1005.4 to 1458.9) | 1683.5 (1377.7 to 2026.1) | 1.09 (0.95 to 1.23) |
| **Guyana** | 10880 (9519 to 12533) | 23155 (18794 to 28570) | 112.8% (78.3 to 149.3) | 2647.1 (2330.7 to 3047.1) | 3382.3 (2761.7 to 4140.0) | 0.62 (0.29 to 0.95) |
| **Haiti** | 86962 (71588 to 104787) | 215965 (168576 to 271547) | 148.3% (97.4 to 214.3) | 2527.1 (2084.9 to 3051.4) | 2734.5 (2144.8 to 3428.4) | 0.33 (0.29 to 0.37) |
| **Honduras** | 17083 (13962 to 21508) | 97275 (77376 to 124995) | 469.4% (401.8 to 553.3) | 750.2 (617.1 to 935.0) | 1413.4 (1130.3 to 1798.3) | 2.16 (2.05 to 2.28) |
| **Hungary** | 80823 (66399 to 96541) | 130789 (103799 to 163761) | 61.8% (48.6 to 74.2) | 553.7 (455.8 to 662.7) | 710.8 (559.5 to 894.4) | 0.91 (0.70 to 1.13) |
| **Iceland** | 641 (526 to 804) | 1939 (1452 to 2567) | 202.4% (169.4 to 230.0) | 228.9 (186.4 to 288.1) | 373.5 (272.9 to 497.2) | 1.57 (1.52 to 1.62) |
| **India** | 3488168 (3019821 to 3988790) | 13013466 (11010697 to 15356408) | 273.1% (230.9 to 313.5) | 713.1 (618.1 to 812.1) | 1057.4 (903.7 to 1242.6) | 1.29 (1.11 to 1.47) |
| **Indonesia** | 717488 (611019 to 831166) | 2607500 (2172231 to 3041835) | 263.4% (214.3 to 316.2) | 672.6 (573.4 to 778.5) | 1031.4 (857.8 to 1199.8) | 1.25 (1.20 to 1.29) |
| **Iran** | 131381 (109046 to 158213) | 782282 (636940 to 968489) | 495.4% (421.8 to 547.8) | 500.5 (419.3 to 598.9) | 976.8 (802.5 to 1197.0) | 2.50 (2.37 to 2.63) |
| **Iraq** | 118677 (98922 to 144473) | 566722 (446136 to 724622) | 377.5% (292.4 to 464.7) | 1420.3 (1186.7 to 1728.2) | 2144.8 (1716.7 to 2701.3) | 1.25 (1.16 to 1.34) |
| **Ireland** | 13793 (11813 to 16445) | 24273 (18413 to 32164) | 76.0% (52.6 to 97.1) | 341.9 (291.1 to 410.2) | 327.9 (246.4 to 436.7) | -0.12 (-0.22 to -0.02) |
| **Israel** | 29709 (26048 to 34701) | 79123 (65456 to 97475) | 166.3% (145.1 to 184.4) | 621.0 (545.6 to 726.3) | 658.5 (540.8 to 813.6) | -0.21 (-0.75 to 0.34) |
| **Italy** | 480335 (429722 to 544988) | 616345 (506369 to 757937) | 28.3% (17.9 to 39.7) | 543.6 (484.1 to 621.3) | 459.1 (370.8 to 579.6) | -0.34 (-0.45 to -0.22) |
| **Jamaica** | 33092 (30487 to 36185) | 64398 (53114 to 78434) | 94.6% (63.7 to 130.6) | 1867.6 (1720.7 to 2048.1) | 2073.7 (1709.1 to 2529.4) | 0.04 (-0.17 to 0.25) |
| **Japan** | 665842 (533021 to 838494) | 1360124 (986916 to 1821168) | 104.3% (83.1 to 125.4) | 395.3 (316.8 to 498.3) | 491.7 (353.3 to 669.1) | 0.63 (0.51 to 0.75) |
| **Jordan** | 22263 (19123 to 26479) | 140054 (109817 to 177751) | 529.1% (403.4 to 648.6) | 1626.3 (1413.3 to 1914.7) | 1717.7 (1376.0 to 2160.6) | -0.10 (-0.33 to 0.14) |
| **Kazakhstan** | 51758 (41184 to 64461) | 133181 (100563 to 174113) | 157.3% (127.8 to 185.8) | 390.2 (311.8 to 486.8) | 704.4 (533.0 to 909.7) | 1.36 (1.14 to 1.59) |
| **Kenya** | 57466 (48176 to 69840) | 227668 (192908 to 271991) | 296.2% (230.3 to 370.2) | 679.5 (571.2 to 826.7) | 952.0 (806.2 to 1133.7) | 1.25 (1.12 to 1.39) |
| **Kiribati** | 1640 (1408 to 1892) | 4432 (3556 to 5663) | 170.3% (115.1 to 260.6) | 4057.5 (3513.8 to 4691.4) | 5517.1 (4478.8 to 6913.2) | 0.92 (0.77 to 1.07) |
| **Kuwait** | 7135 (5591 to 9043) | 59337 (43986 to 78844) | 731.7% (645.7 to 805.8) | 991.6 (804.6 to 1239.6) | 1619.9 (1228.9 to 2127.4) | 1.55 (1.32 to 1.78) |
| **Kyrgyzstan** | 8757 (7174 to 10804) | 28202 (22126 to 35993) | 222.1% (189.7 to 256.3) | 281.3 (230.6 to 346.5) | 522.1 (411.2 to 664.1) | 1.47 (1.23 to 1.71) |
| **Laos** | 24614 (19578 to 30197) | 65809 (52752 to 82672) | 167.4% (105.0 to 244.5) | 1111.1 (892.7 to 1354.6) | 1349.4 (1077.5 to 1677.2) | 0.48 (0.43 to 0.53) |
| **Latvia** | 9985 (8197 to 12097) | 22569 (18765 to 27637) | 126.0% (108.9 to 145.5) | 284.0 (232.7 to 345.2) | 645.0 (534.8 to 794.5) | 2.40 (2.11 to 2.69) |
| **Lebanon** | 26473 (21899 to 32551) | 90067 (69953 to 112969) | 240.2% (180.8 to 300.4) | 1208.9 (1007.5 to 1477.9) | 1505.3 (1166.0 to 1893.0) | 0.97 (0.87 to 1.08) |
| **Lesotho** | 9370 (7850 to 11434) | 28649 (22132 to 36001) | 205.8% (128.9 to 308.8) | 1111.0 (932.4 to 1356.7) | 2588.9 (2018.8 to 3208.2) | 3.59 (3.10 to 4.09) |
| **Liberia** | 10395 (8582 to 12623) | 34738 (26822 to 44955) | 234.2% (167.1 to 314.9) | 875.8 (728.5 to 1057.8) | 1403.4 (1106.1 to 1788.1) | 1.60 (1.49 to 1.72) |
| **Libya** | 11804 (9378 to 14560) | 78970 (61109 to 103604) | 569.0% (469.3 to 665.8) | 589.7 (470.9 to 720.5) | 1352.3 (1045.8 to 1758.1) | 3.15 (2.97 to 3.33) |
| **Lithuania** | 9680 (7730 to 12387) | 25148 (20486 to 31334) | 159.8% (138.9 to 181.6) | 217.2 (173.0 to 277.2) | 494.8 (399.8 to 627.2) | 2.27 (1.95 to 2.60) |
| **Luxembourg** | 1859 (1605 to 2203) | 3995 (3044 to 5182) | 114.9% (87.3 to 139.8) | 349.8 (300.9 to 417.3) | 401.6 (301.9 to 525.7) | 0.42 (0.32 to 0.52) |
| **Macedonia** | 14324 (11757 to 17286) | 38965 (31292 to 49125) | 172.0% (132.8 to 217.5) | 761.0 (625.7 to 915.6) | 1173.0 (952.5 to 1468.4) | 1.45 (1.19 to 1.71) |
| **Madagascar** | 47796 (39881 to 57325) | 123961 (97862 to 156875) | 159.4% (105.3 to 227.9) | 899.9 (746.8 to 1077.3) | 995.3 (789.0 to 1257.7) | 0.31 (0.26 to 0.36) |
| **Malawi** | 42646 (36097 to 50178) | 96853 (80415 to 115309) | 127.1% (86.3 to 182.7) | 1074.5 (915.7 to 1262.2) | 1217.3 (1013.7 to 1441.9) | 0.12 (-0.13 to 0.37) |
| **Malaysia** | 92417 (79930 to 106246) | 304844 (245485 to 372212) | 229.9% (184.4 to 268.0) | 955.6 (835.1 to 1100.4) | 1044.2 (840.5 to 1269.1) | 0.09 (-0.05 to 0.22) |
| **Maldives** | 1002 (858 to 1166) | 3031 (2418 to 3823) | 202.4% (146.8 to 267.2) | 1042.3 (897.9 to 1209.3) | 832.2 (674.1 to 1058.2) | -1.01 (-1.24 to -0.79) |
| **Mali** | 44850 (37818 to 53605) | 161559 (130139 to 203331) | 260.2% (200.0 to 321.0) | 1075.3 (913.2 to 1281.6) | 1630.6 (1321.7 to 2026.4) | 1.44 (1.35 to 1.53) |
| **Malta** | 2496 (2238 to 2810) | 5864 (4753 to 7465) | 134.9% (105.7 to 167.4) | 590.0 (528.8 to 664.0) | 669.4 (533.5 to 865.9) | 0.43 (0.11 to 0.75) |
| **Marshall Islands** | 606 (498 to 729) | 2358 (1799 to 3178) | 288.9% (204.3 to 380.7) | 3375.1 (2785.5 to 4078.5) | 5749.1 (4434.4 to 7748.6) | 1.69 (1.47 to 1.92) |
| **Mauritania** | 8263 (6840 to 9771) | 22927 (18105 to 28643) | 177.4% (120.2 to 249.9) | 809.2 (667.5 to 955.8) | 1026.3 (807.3 to 1290.7) | 0.57 (0.49 to 0.64) |
| **Mauritius** | 13432 (12208 to 14971) | 62832 (56825 to 70752) | 367.8% (338.0 to 394.3) | 1738.0 (1575.4 to 1932.1) | 3385.9 (3066.4 to 3810.0) | 3.31 (2.65 to 3.98) |
| **Mexico** | 1036743 (930500 to 1161801) | 3065910 (2648353 to 3563377) | 195.7% (173.8 to 220.3) | 2281.8 (2065.6 to 2538.7) | 2344.6 (2028.4 to 2723.7) | -0.13 (-0.32 to 0.06) |
| **Moldova** | 16558 (13547 to 20283) | 35034 (27386 to 44340) | 111.6% (96.1 to 128.2) | 365.1 (298.4 to 447.6) | 612.2 (478.1 to 780.2) | 1.24 (0.98 to 1.49) |
| **Mongolia** | 3032 (2400 to 3722) | 14455 (11370 to 18324) | 376.7% (295.3 to 464.3) | 266.3 (210.0 to 326.5) | 531.6 (422.2 to 665.1) | 2.36 (2.29 to 2.43) |
| **Montenegro** | 3518 (2812 to 4399) | 8041 (6418 to 10119) | 128.6% (100.9 to 156.5) | 559.6 (450.9 to 699.0) | 829.3 (665.4 to 1040.3) | 1.29 (1.21 to 1.38) |
| **Morocco** | 93245 (73740 to 120358) | 560299 (423278 to 734524) | 500.9% (427.6 to 563.8) | 626.9 (496.8 to 811.8) | 1555.9 (1186.5 to 2020.1) | 3.17 (3.09 to 3.25) |
| **Mozambique** | 60801 (51865 to 71673) | 178462 (139085 to 219706) | 193.5% (129.0 to 274.6) | 979.6 (847.2 to 1139.2) | 1419.1 (1119.0 to 1727.9) | 1.68 (1.52 to 1.85) |
| **Myanmar** | 424382 (341324 to 521689) | 962561 (789402 to 1171149) | 126.8% (75.0 to 194.2) | 1722.3 (1407.3 to 2104.0) | 1932.4 (1580.2 to 2343.5) | 0.12 (0.03 to 0.20) |
| **Namibia** | 9579 (8130 to 11737) | 25760 (20245 to 32114) | 168.9% (103.4 to 243.5) | 1470.1 (1247.1 to 1783.9) | 1866.3 (1485.4 to 2307.8) | 0.55 (0.26 to 0.85) |
| **Nepal** | 71035 (58471 to 86001) | 287934 (228666 to 361542) | 305.3% (237.7 to 394.1) | 712.1 (590.3 to 859.5) | 1191.6 (956.6 to 1482.5) | 1.75 (1.55 to 1.95) |
| **Netherlands** | 93849 (83021 to 106085) | 126188 (98607 to 159674) | 34.5% (17.2 to 54.0) | 473.6 (418.1 to 537.4) | 396.1 (299.4 to 507.7) | -0.74 (-0.91 to -0.57) |
| **New Zealand** | 16453 (14080 to 19565) | 37157 (29336 to 46350) | 125.8% (104.4 to 144.6) | 429.7 (366.4 to 510.3) | 472.5 (370.8 to 588.9) | 0.01 (-0.19 to 0.21) |
| **Nicaragua** | 16531 (13954 to 19723) | 75230 (61693 to 91919) | 355.1% (307.0 to 406.2) | 978.2 (832.9 to 1157.6) | 1445.4 (1191.4 to 1754.6) | 1.22 (1.10 to 1.34) |
| **Niger** | 20339 (16340 to 24824) | 89355 (70169 to 114031) | 339.3% (262.0 to 427.6) | 673.7 (544.4 to 818.5) | 978.7 (772.5 to 1242.7) | 1.22 (1.14 to 1.29) |
| **Nigeria** | 409757 (346765 to 488700) | 1015687 (821899 to 1251125) | 147.9% (98.5 to 215.6) | 908.7 (775.0 to 1073.4) | 1051.2 (869.3 to 1272.2) | 0.37 (0.32 to 0.42) |
| **North Korea** | 85695 (66046 to 105434) | 241699 (192545 to 304963) | 182.0% (131.2 to 239.6) | 502.5 (389.3 to 615.0) | 726.3 (578.1 to 913.7) | 1.22 (1.16 to 1.29) |
| **Northern Mariana Islands** | 411 (333 to 502) | 1206 (1045 to 1425) | 193.4% (132.7 to 255.6) | 1902.4 (1574.9 to 2305.9) | 2162.8 (1879.7 to 2540.0) | 0.39 (0.16 to 0.62) |
| **Norway** | 20509 (16737 to 25309) | 32383 (25161 to 41947) | 57.9% (49.7 to 66.1) | 330.7 (265.6 to 411.6) | 363.9 (277.1 to 474.1) | 0.09 (-0.05 to 0.24) |
| **Oman** | 8244 (6482 to 10480) | 36157 (29485 to 44264) | 338.6% (215.7 to 458.8) | 1166.4 (919.9 to 1478.9) | 1565.0 (1305.9 to 1891.3) | 1.09 (0.97 to 1.20) |
| **Pakistan** | 483550 (406860 to 579452) | 2012195 (1629998 to 2454515) | 316.1% (257.7 to 377.3) | 835.0 (702.8 to 997.3) | 1525.2 (1260.9 to 1837.7) | 1.85 (1.61 to 2.08) |
| **Palestine** | 11638 (9450 to 14174) | 45920 (38382 to 55146) | 294.6% (214.9 to 385.8) | 1363.3 (1110.2 to 1647.7) | 1767.2 (1489.2 to 2085.1) | 0.88 (0.77 to 0.98) |
| **Panama** | 12302 (10509 to 14803) | 54998 (44524 to 68457) | 347.1% (293.4 to 397.5) | 786.9 (674.0 to 945.4) | 1243.4 (1005.7 to 1548.9) | 1.33 (1.18 to 1.47) |
| **Papua New Guinea** | 52586 (40018 to 67721) | 180817 (145851 to 222446) | 243.8% (150.4 to 364.8) | 2582.0 (1985.6 to 3280.3) | 3024.5 (2475.0 to 3705.5) | 0.50 (0.48 to 0.53) |
| **Paraguay** | 19671 (16955 to 23789) | 107495 (87443 to 132054) | 446.5% (356.4 to 557.3) | 853.3 (732.4 to 1023.8) | 1807.6 (1475.0 to 2215.5) | 2.65 (2.37 to 2.93) |
| **Peru** | 60635 (51855 to 71383) | 237765 (189324 to 295912) | 292.1% (228.5 to 361.0) | 485.6 (416.2 to 570.5) | 699.7 (558.1 to 871.3) | 1.08 (0.90 to 1.26) |
| **Philippines** | 295418 (261479 to 331995) | 1116920 (965303 to 1301512) | 278.1% (234.9 to 330.0) | 923.9 (819.9 to 1038.8) | 1294.1 (1124.4 to 1510.5) | 1.18 (1.12 to 1.25) |
| **Poland** | 263899 (222950 to 313488) | 494688 (396003 to 609892) | 87.5% (76.0 to 96.6) | 599.7 (506.4 to 713.3) | 725.9 (578.7 to 900.2) | 0.80 (0.56 to 1.04) |
| **Portugal** | 89075 (78887 to 102144) | 153027 (121321 to 194633) | 71.8% (52.2 to 93.9) | 648.4 (571.5 to 748.3) | 700.4 (541.1 to 915.2) | -0.07 (-0.22 to 0.09) |
| **Principality of Monaco** | 113 (90 to 143) | 265 (197 to 348) | 133.1% (104.4 to 162.7) | 186.8 (146.4 to 238.9) | 338.3 (245.7 to 450.7) | 1.97 (1.93 to 2.00) |
| **Puerto Rico** | 58913 (52200 to 67181) | 117561 (97344 to 144666) | 99.5% (81.5 to 119.1) | 1640.8 (1454.4 to 1867.9) | 1877.0 (1529.4 to 2338.0) | 0.15 (-0.04 to 0.33) |
| **Qatar** | 2341 (1936 to 2802) | 30041 (22944 to 38754) | 1183.1% (902.3 to 1458.2) | 2125.1 (1784.9 to 2543.1) | 2524.9 (2033.5 to 3132.6) | 0.47 (-0.07 to 1.02) |
| **Republic of Nauru** | 185 (149 to 231) | 322 (261 to 398) | 74.2% (37.7 to 123.0) | 3547.1 (2866.0 to 4377.0) | 4875.8 (4004.7 to 5917.0) | 0.86 (0.69 to 1.03) |
| **Republic of Niue** | 55 (47 to 66) | 89 (71 to 109) | 61.5% (31.6 to 99.7) | 2542.1 (2140.6 to 3047.8) | 4152.3 (3318.3 to 5107.5) | 1.46 (1.32 to 1.59) |
| **Republic of Palau** | 272 (222 to 327) | 883 (720 to 1078) | 225.1% (155.1 to 312.1) | 2616.9 (2167.1 to 3148.0) | 3719.2 (3077.0 to 4507.6) | 1.18 (1.06 to 1.30) |
| **Republic of San Marino** | 92 (76 to 113) | 229 (171 to 304) | 148.2% (112.7 to 185.9) | 270.7 (222.8 to 335.4) | 364.1 (269.4 to 488.0) | 1.11 (1.02 to 1.21) |
| **Romania** | 102878 (84183 to 125398) | 158872 (124355 to 203025) | 54.4% (41.6 to 67.8) | 364.2 (297.8 to 444.1) | 457.5 (351.7 to 593.0) | 0.81 (0.69 to 0.92) |
| **Russian Federation** | 445854 (359124 to 549923) | 1473449 (1263183 to 1757433) | 230.5% (212.8 to 254.0) | 246.3 (198.3 to 304.6) | 625.3 (532.9 to 750.4) | 2.63 (2.16 to 3.10) |
| **Rwanda** | 43312 (31982 to 56442) | 69064 (46574 to 95241) | 59.5% (21.0 to 113.4) | 1445.9 (1070.7 to 1888.8) | 1059.7 (720.8 to 1457.2) | -1.97 (-2.32 to -1.61) |
| **Saint Kitts and Nevis** | 785 (713 to 877) | 1414 (1146 to 1747) | 80.0% (54.8 to 107.8) | 2148.6 (1948.1 to 2411.4) | 1971.3 (1618.3 to 2403.7) | -0.08 (-0.26 to 0.10) |
| **Saint Lucia** | 2421 (2179 to 2728) | 5452 (4460 to 6652) | 125.2% (97.1 to 153.1) | 2781.4 (2512.3 to 3124.5) | 2275.3 (1861.9 to 2772.5) | -1.18 (-1.35 to -1.01) |
| **Saint Vincent and the Grenadines** | 1945 (1765 to 2175) | 3730 (3159 to 4456) | 91.7% (71.5 to 114.1) | 2725.4 (2474.7 to 3042.8) | 2625.8 (2228.5 to 3136.6) | -0.48 (-0.65 to -0.30) |
| **Samoa** | 2096 (1741 to 2511) | 5192 (4279 to 6308) | 147.6% (104.0 to 202.3) | 2339.6 (1957.8 to 2787.2) | 3379.5 (2807.2 to 4085.2) | 1.15 (1.09 to 1.21) |
| **Sao Tome and Principe** | 329 (271 to 413) | 1102 (875 to 1400) | 234.5% (191.0 to 279.1) | 497.7 (411.5 to 622.2) | 875.7 (705.6 to 1112.0) | 1.82 (1.74 to 1.89) |
| **Saudi Arabia** | 55684 (43947 to 69236) | 370227 (289464 to 472194) | 564.9% (402.6 to 723.0) | 857.0 (674.2 to 1060.2) | 1409.2 (1137.0 to 1778.4) | 1.47 (1.39 to 1.55) |
| **Senegal** | 33159 (27797 to 40048) | 128365 (101896 to 158057) | 287.1% (224.6 to 359.7) | 971.7 (814.8 to 1165.0) | 1561.2 (1234.3 to 1906.0) | 1.61 (1.49 to 1.74) |
| **Serbia** | 82822 (68202 to 99151) | 154932 (124502 to 192884) | 87.1% (66.0 to 110.8) | 747.3 (621.1 to 890.1) | 962.1 (773.3 to 1203.3) | 0.68 (0.56 to 0.79) |
| **Seychelles** | 382 (327 to 455) | 1773 (1389 to 2264) | 363.7% (310.9 to 413.9) | 673.1 (575.2 to 801.5) | 1490.7 (1171.7 to 1891.5) | 2.73 (2.58 to 2.88) |
| **Sierra Leone** | 14998 (12471 to 18107) | 47272 (38136 to 60232) | 215.2% (153.3 to 282.2) | 709.1 (592.4 to 856.6) | 1122.2 (910.9 to 1406.3) | 1.65 (1.47 to 1.83) |
| **Singapore** | 19343 (15753 to 24217) | 54785 (37725 to 77453) | 183.2% (138.3 to 226.0) | 807.6 (664.3 to 999.9) | 643.3 (442.5 to 909.9) | -0.89 (-1.07 to -0.71) |
| **Slovakia** | 29211 (24123 to 35590) | 48820 (38151 to 62682) | 67.1% (50.4 to 85.0) | 487.1 (401.2 to 595.1) | 526.4 (410.4 to 678.0) | 0.25 (0.20 to 0.30) |
| **Slovenia** | 11903 (9843 to 14557) | 21151 (16769 to 27161) | 77.7% (63.3 to 92.0) | 483.9 (399.0 to 590.5) | 512.4 (403.0 to 667.2) | -0.66 (-0.93 to -0.40) |
| **Solomon Islands** | 3683 (2502 to 5097) | 14112 (11198 to 17454) | 283.2% (175.7 to 470.6) | 2387.6 (1693.7 to 3228.0) | 3468.3 (2798.1 to 4245.5) | 1.25 (1.18 to 1.32) |
| **Somalia** | 35579 (27023 to 45970) | 103906 (81202 to 126381) | 192.0% (127.5 to 278.2) | 1336.5 (1041.4 to 1668.6) | 1493.6 (1182.0 to 1808.4) | 0.37 (0.29 to 0.45) |
| **South Africa** | 262094 (237877 to 291327) | 1010377 (914208 to 1116271) | 285.5% (253.0 to 314.9) | 1220.3 (1106.0 to 1351.6) | 2154.2 (1956.6 to 2375.6) | 2.12 (1.75 to 2.48) |
| **South Korea** | 251385 (214857 to 294696) | 829985 (621168 to 1099982) | 230.2% (181.6 to 277.0) | 771.0 (662.6 to 893.9) | 942.8 (702.0 to 1250.5) | 0.28 (0.10 to 0.47) |
| **South Sudan** | 29875 (22603 to 39280) | 61855 (48012 to 81075) | 107.0% (60.4 to 185.9) | 1142.5 (868.7 to 1497.3) | 1481.4 (1155.4 to 1941.7) | 0.74 (0.58 to 0.89) |
| **Spain** | 325654 (274189 to 388792) | 528689 (399537 to 705137) | 62.3% (44.7 to 80.9) | 604.2 (506.6 to 719.2) | 604.7 (450.7 to 822.6) | -0.20 (-0.27 to -0.14) |
| **Sri Lanka** | 123442 (105776 to 142117) | 517582 (401226 to 646881) | 319.3% (236.4 to 416.6) | 1162.0 (998.5 to 1341.9) | 1911.3 (1485.1 to 2386.6) | 2.06 (1.82 to 2.30) |
| **Sudan** | 49133 (39747 to 61058) | 208455 (159031 to 272023) | 324.3% (252.8 to 398.8) | 503.6 (411.3 to 620.1) | 944.8 (729.8 to 1223.4) | 2.17 (2.09 to 2.25) |
| **Suriname** | 3833 (3303 to 4432) | 13583 (10917 to 17037) | 254.3% (206.2 to 309.8) | 1431.2 (1234.1 to 1649.7) | 2087.2 (1679.8 to 2608.6) | 1.36 (1.25 to 1.48) |
| **Swaziland** | 5706 (4634 to 6787) | 18773 (14477 to 24504) | 229.0% (150.7 to 342.9) | 1991.5 (1635.9 to 2370.0) | 3242.4 (2550.1 to 4173.5) | 1.96 (1.33 to 2.61) |
| **Sweden** | 46423 (39362 to 55690) | 77635 (62149 to 98861) | 67.2% (53.9 to 80.3) | 332.6 (277.4 to 402.5) | 410.8 (320.0 to 528.8) | 0.75 (0.66 to 0.84) |
| **Switzerland** | 47787 (40337 to 57047) | 85267 (63309 to 115739) | 78.4% (55.2 to 101.8) | 471.9 (392.7 to 570.2) | 541.4 (396.6 to 740.4) | 0.21 (0.08 to 0.33) |
| **Syria** | 38384 (31650 to 46503) | 144677 (111878 to 190545) | 276.9% (215.0 to 349.8) | 691.7 (575.6 to 832.9) | 1053.3 (819.9 to 1378.8) | 1.09 (0.97 to 1.22) |
| **Taiwan (Province of China)** | 164823 (147158 to 185515) | 388605 (318504 to 473915) | 135.8% (111.9 to 157.4) | 1013.6 (911.9 to 1133.8) | 942.2 (767.2 to 1147.7) | -1.07 (-1.43 to -0.70) |
| **Tajikistan** | 13191 (11070 to 15823) | 46036 (36789 to 59178) | 249.0% (189.0 to 325.7) | 447.7 (378.3 to 534.2) | 677.4 (542.8 to 865.4) | 1.06 (0.85 to 1.26) |
| **Tanzania** | 100614 (84717 to 117855) | 277039 (222198 to 345047) | 175.3% (114.9 to 251.3) | 905.1 (770.4 to 1048.4) | 1023.6 (831.7 to 1266.4) | 0.23 (0.15 to 0.30) |
| **Thailand** | 287437 (241973 to 336774) | 1048680 (839694 to 1304488) | 264.8% (197.7 to 344.8) | 768.2 (646.2 to 904.4) | 984.8 (788.7 to 1221.3) | 0.33 (0.16 to 0.50) |
| **The Bahamas** | 2597 (2272 to 2992) | 7159 (5729 to 8918) | 175.6% (136.7 to 215.6) | 1600.3 (1405.2 to 1825.1) | 1680.6 (1354.0 to 2084.8) | -0.13 (-0.27 to 0.00) |
| **The Gambia** | 2938 (2330 to 3643) | 14612 (11730 to 18385) | 397.3% (298.9 to 513.6) | 777.8 (621.6 to 955.2) | 1361.1 (1096.5 to 1705.6) | 1.74 (1.62 to 1.87) |
| **Timor-Leste** | 1815 (1411 to 2270) | 8924 (7132 to 11338) | 391.7% (284.6 to 526.3) | 584.8 (460.4 to 718.6) | 1012.5 (811.8 to 1283.6) | 1.94 (1.77 to 2.12) |
| **Togo** | 9102 (7618 to 10996) | 43607 (34382 to 55196) | 379.1% (287.8 to 506.3) | 678.3 (562.7 to 818.4) | 1050.3 (847.1 to 1312.8) | 1.39 (1.23 to 1.54) |
| **Tokelau** | 33 (27 to 41) | 49 (40 to 60) | 47.3% (17.1 to 79.4) | 2476.7 (2004.8 to 3072.9) | 3325.1 (2713.0 to 4094.3) | 0.86 (0.75 to 0.97) |
| **Tonga** | 1549 (1327 to 1784) | 2971 (2440 to 3668) | 91.8% (52.7 to 137.4) | 2675.1 (2297.7 to 3081.7) | 3615.6 (2977.5 to 4456.0) | 0.94 (0.83 to 1.05) |
| **Trinidad and Tobago** | 32054 (29553 to 34964) | 64981 (52975 to 79960) | 102.7% (70.2 to 136.4) | 3780.9 (3496.7 to 4111.1) | 3371.1 (2753.5 to 4147.9) | -0.70 (-0.84 to -0.56) |
| **Tunisia** | 25171 (20073 to 32228) | 147847 (110825 to 198874) | 487.4% (410.1 to 571.7) | 487.4 (391.8 to 618.1) | 1091.4 (824.6 to 1467.3) | 2.59 (2.45 to 2.73) |
| **Turkey** | 323021 (278847 to 377737) | 987741 (799541 to 1217662) | 205.8% (149.9 to 262.6) | 943.2 (818.8 to 1097.5) | 1048.5 (850.5 to 1286.4) | 0.53 (0.23 to 0.82) |
| **Turkmenistan** | 7704 (6557 to 8979) | 38812 (31889 to 48122) | 403.8% (318.4 to 509.0) | 372.4 (318.7 to 431.8) | 857.2 (707.1 to 1062.4) | 2.23 (1.94 to 2.51) |
| **Tuvalu** | 178 (147 to 220) | 350 (288 to 420) | 96.5% (58.0 to 134.0) | 2517.0 (2086.9 to 3084.9) | 3232.7 (2671.7 to 3862.2) | 0.80 (0.73 to 0.88) |
| **Uganda** | 65697 (47802 to 99425) | 191264 (143891 to 265993) | 191.1% (113.3 to 286.1) | 1005.5 (733.1 to 1511.5) | 1210.5 (903.5 to 1651.5) | 0.14 (-0.09 to 0.36) |
| **Ukraine** | 171010 (138285 to 208856) | 265379 (199498 to 343018) | 55.2% (39.8 to 70.1) | 244.1 (196.0 to 299.7) | 369.1 (276.8 to 479.9) | 0.73 (0.54 to 0.91) |
| **United Arab Emirates** | 6092 (4859 to 7578) | 77857 (58537 to 101693) | 1177.9% (851.4 to 1440.2) | 1271.7 (1020.9 to 1582.3) | 1465.8 (1175.6 to 1796.9) | 1.35 (0.96 to 1.75) |
| **United Kingdom** | 285152 (243424 to 342094) | 570922 (423806 to 758333) | 100.2% (73.6 to 122.6) | 335.6 (281.7 to 405.1) | 541.4 (392.5 to 729.3) | 1.37 (1.16 to 1.58) |
| **United States** | 1696589 (1476616 to 1982145) | 4794607 (3780641 to 6009339) | 182.6% (154.3 to 206.1) | 553.7 (481.0 to 648.4) | 886.6 (699.9 to 1108.6) | 1.27 (1.09 to 1.45) |
| **Uruguay** | 19164 (17053 to 21723) | 37269 (30686 to 45747) | 94.5% (75.4 to 115.3) | 492.4 (437.1 to 560.0) | 715.2 (583.2 to 886.0) | 1.24 (1.15 to 1.33) |
| **Uzbekistan** | 49145 (42230 to 56804) | 315211 (264963 to 380970) | 541.4% (476.7 to 613.1) | 401.3 (345.9 to 464.6) | 1071.4 (903.9 to 1285.6) | 2.99 (2.63 to 3.35) |
| **Vanuatu** | 1429 (1116 to 2019) | 5877 (4863 to 7041) | 311.4% (215.7 to 431.1) | 2074.8 (1638.9 to 2915.0) | 3002.2 (2509.8 to 3605.8) | 1.09 (1.04 to 1.14) |
| **Venezuela** | 115177 (101477 to 133157) | 468853 (382323 to 579250) | 307.1% (245.5 to 374.8) | 1118.1 (993.3 to 1276.0) | 1541.9 (1260.4 to 1899.9) | 0.90 (0.67 to 1.12) |
| **Vietnam** | 325720 (272095 to 396422) | 1064325 (864983 to 1298019) | 226.8% (158.7 to 310.0) | 811.2 (680.2 to 978.3) | 1093.8 (899.2 to 1328.8) | 1.08 (0.99 to 1.16) |
| **Virgin Islands, U.S.** | 1349 (1132 to 1649) | 3026 (2350 to 3895) | 124.2% (91.9 to 161.5) | 1544.7 (1302.2 to 1860.3) | 1852.4 (1422.8 to 2385.3) | 0.60 (0.52 to 0.68) |
| **Yemen** | 24387 (19008 to 32194) | 119765 (91998 to 158507) | 391.1% (299.2 to 483.2) | 466.9 (366.7 to 615.1) | 760.2 (587.5 to 995.0) | 1.58 (1.45 to 1.71) |
| **Zambia** | 38957 (33047 to 45695) | 114849 (91008 to 141630) | 194.8% (123.6 to 283.0) | 1283.4 (1100.6 to 1511.9) | 1431.1 (1154.9 to 1746.3) | -0.08 (-0.27 to 0.10) |
| **Zimbabwe** | 39006 (32349 to 45747) | 133242 (107315 to 163900) | 241.6% (165.5 to 341.4) | 957.0 (795.3 to 1118.6) | 1847.5 (1502.6 to 2244.5) | 2.69 (2.16 to 3.24) |

ASDR, age-standardized DALYs rate; UI, uncertainty interval; EAPC, estimated annual percentage change; CI, confidence.

# Supplementary Table 10. Contribution of population-level drivers to changes in incidence numbers of type 2 diabetes mellitus from 1990 to 2021

|  | Overall differencea | Agingb | Populationc | Epidemiological changesd |
| --- | --- | --- | --- | --- |
| Global | 7796794.57 | 997104.66 (12.79%) | 4000830.49 (51.31%) | 2798859.43 (35.90%) |
| **Sex** | | | | |
| Male | 3905514.06 | 537515.59 (13.76%) | 1995428.85 (51.09%) | 1372569.62 (35.14%) |
| Female | 3849168.15 | 451267.93 (11.72%) | 2006835.89 (52.14%) | 1391064.33 (36.14%) |
| **SDI regions** | | | | |
| High SDI | 3110655.50 | 378674.64 (12.17%) | 861547.35 (27.70%) | 1870433.51 (60.13%) |
| High-middle SDI | 2282363.47 | 358868.68 (15.72%) | 852355.37 (37.35%) | 1071139.42 (46.93%) |
| Middle SDI | 3209621.16 | 651509.12 (20.30%) | 1496209.54 (46.62%) | 1061902.50 (33.08%) |
| Low-middle SDI | 2105909.22 | 132511.14 (6.29%) | 1142824.82 (54.27%) | 830573.27 (39.44%) |
| Low SDI | 494794.39 | -9145.00 (-1.85%) | 591371.00 (119.52%) | -87431.61 (-17.67%) |
| **21 GBD regions** | | | | |
| Andean Latin America | 62977.11 | 4570.10 (7.26%) | 37344.54 (59.30%) | 21062.47 (33.44%) |
| Australasia | 37783.39 | 6586.04 (17.43%) | 16433.19 (43.49%) | 14764.16 (39.08%) |
| Caribbean | 145790.86 | 16273.90 (11.16%) | 62106.69 (42.60%) | 67410.27 (46.24%) |
| Central Asia | 112276.64 | -21302.66 (-18.97%) | 58950.99 (52.51%) | 74628.31 (66.47%) |
| Central Europe | 185137.10 | 22682.38 (12.25%) | 23186.16 (12.52%) | 139268.55 (75.22%) |
| Central Latin America | 368805.78 | 70922.32 (19.23%) | 221774.52 (60.13%) | 76108.94 (20.64%) |
| Central Sub-Saharan Africa | 36070.74 | -2501.60 (-6.94%) | 83079.35 (230.32%) | -44507.01 (-123.39%) |
| East Asia | 1543226.18 | 459393.15 (29.77%) | 597916.29 (38.74%) | 485916.75 (31.49%) |
| Eastern Europe | 171456.74 | 29378.92 (17.13%) | 1925.69 (1.12%) | 140152.12 (81.74%) |
| Eastern Sub-Saharan Africa | 128665.91 | -3318.78 (-2.58%) | 144885.19 (112.61%) | -12900.49 (-10.03%) |
| High-income Asia Pacific | 464480.95 | 135353.79 (29.14%) | 102243.01 (22.01%) | 226884.15 (48.85%) |
| High-income North America | 1159492.07 | 219587.42 (18.94%) | 284613.26 (24.55%) | 655291.39 (56.52%) |
| North Africa and Middle East | 3580435.80 | 124744.42 (3.48%) | 1742719.14 (48.67%) | 1712972.23 (47.84%) |
| Oceania | 15852.09 | 447.78 (2.82%) | 17516.24 (110.50%) | -2111.94 (-13.32%) |
| South Asia | 4999973.16 | 13729.02 (0.27%) | 3667568.09 (73.35%) | 1318676.06 (26.37%) |
| Southeast Asia | 495893.85 | 16714.99 (3.37%) | 433318.00 (87.38%) | 45860.86 (9.25%) |
| Southern Latin America | 87951.05 | 10234.24 (11.64%) | 33403.67 (37.98%) | 44313.15 (50.38%) |
| Southern Sub-Saharan Africa | 18810.18 | -12347.48 (-65.64%) | 61725.56 (328.15%) | -30567.90 (-162.51%) |
| Tropical Latin America | 235957.59 | 55416.49 (23.49%) | 145044.12 (61.47%) | 35496.98 (15.04%) |
| Western Europe | 477799.77 | 95884.98 (20.07%) | 117870.83 (24.67%) | 264043.96 (55.26%) |
| Western Sub-Saharan Africa | 208241.20 | -10049.84 (-4.83%) | 295130.71 (141.73%) | -76839.67 (-36.90%) |
| **Countries/territories** | | | | |
| Afghanistan | 34782.01 | -21199.95 (-60.95%) | 30934.35 (88.94%) | 25047.61 (72.01%) |
| Albania | 2376.12 | 1214.23 (51.10%) | 251.21 (10.57%) | 910.68 (38.33%) |
| Algeria | 70125.92 | 5802.41 (8.27%) | 34639.08 (49.40%) | 29684.42 (42.33%) |
| American Samoa | 204.27 | 58.07 (28.43%) | 35.99 (17.62%) | 110.21 (53.96%) |
| Andorra | 192.53 | 22.72 (11.80%) | 77.20 (40.10%) | 92.61 (48.10%) |
| Angola | 19088.67 | 34.07 (0.18%) | 15338.59 (80.35%) | 3716.01 (19.47%) |
| Antigua and Barbuda | 240.65 | 31.97 (13.28%) | 118.47 (49.23%) | 90.21 (37.48%) |
| Argentina | 61523.61 | 3155.82 (5.13%) | 25911.27 (42.12%) | 32456.52 (52.75%) |
| Armenia | 3702.57 | 361.91 (9.77%) | 463.74 (12.52%) | 2876.91 (77.70%) |
| Australia | 40197.05 | -4898.05 (-12.19%) | 23216.85 (57.76%) | 21878.25 (54.43%) |
| Austria | 5079.86 | 523.26 (10.30%) | 1574.30 (30.99%) | 2982.30 (58.71%) |
| Azerbaijan | 9176.60 | 715.68 (7.80%) | 3860.07 (42.06%) | 4600.84 (50.14%) |
| Bahrain | 4702.24 | 817.52 (17.39%) | 2354.63 (50.07%) | 1530.09 (32.54%) |
| Bangladesh | 155205.17 | -14447.49 (-9.31%) | 171868.65 (110.74%) | -2215.98 (-1.43%) |
| Barbados | 646.26 | 122.53 (18.96%) | 267.08 (41.33%) | 256.65 (39.71%) |
| Belarus | 5718.01 | 1394.05 (24.38%) | -8.41 (-0.15%) | 4332.37 (75.77%) |
| Belgium | 17122.70 | 1057.14 (6.17%) | 3894.09 (22.74%) | 12171.47 (71.08%) |
| Belize | 721.07 | 28.99 (4.02%) | 461.30 (63.97%) | 230.78 (32.01%) |
| Benin | 5767.78 | -224.07 (-3.88%) | 8365.75 (145.04%) | -2373.91 (-41.16%) |
| Bermuda | 105.77 | 36.98 (34.96%) | 21.92 (20.73%) | 46.87 (44.31%) |
| Bhutan | 436.46 | -46.88 (-10.74%) | 448.23 (102.70%) | 35.10 (8.04%) |
| Bolivia | 15214.56 | 1406.77 (9.25%) | 8161.73 (53.64%) | 5646.06 (37.11%) |
| Bosnia and Herzegovina | 6763.92 | 3195.26 (47.24%) | -946.96 (-14.00%) | 4515.63 (66.76%) |
| Botswana | 684.88 | -506.67 (-73.98%) | 2297.61 (335.48%) | -1106.06 (-161.50%) |
| Brazil | 224313.20 | 50374.96 (22.46%) | 141811.63 (63.22%) | 32126.61 (14.32%) |
| Brunei | 1467.72 | 287.51 (19.59%) | 569.12 (38.78%) | 611.08 (41.63%) |
| Bulgaria | 10663.29 | 3432.47 (32.19%) | -2530.84 (-23.73%) | 9761.66 (91.54%) |
| Burkina Faso | 3706.51 | -167.14 (-4.51%) | 13107.70 (353.64%) | -9234.05 (-249.13%) |
| Burundi | 2682.99 | -299.50 (-11.16%) | 7822.85 (291.57%) | -4840.36 (-180.41%) |
| Cambodia | 35668.43 | 2969.21 (8.32%) | 16078.65 (45.08%) | 16620.58 (46.60%) |
| Cameroon | 16067.59 | -903.55 (-5.62%) | 14342.92 (89.27%) | 2628.21 (16.36%) |
| Canada | 102035.01 | 21043.97 (20.62%) | 24686.42 (24.19%) | 56304.62 (55.18%) |
| Cape Verde | 597.78 | -69.83 (-11.68%) | 394.78 (66.04%) | 272.83 (45.64%) |
| Central African Republic | 3691.94 | -14.81 (-0.40%) | 2487.67 (67.38%) | 1219.09 (33.02%) |
| Chad | 5062.96 | -276.90 (-5.47%) | 7822.01 (154.49%) | -2482.15 (-49.03%) |
| Chile | 41605.68 | 8690.15 (20.89%) | 14193.55 (34.11%) | 18721.98 (45.00%) |
| China | 1527543.68 | 488270.04 (31.96%) | 571863.38 (37.44%) | 467410.26 (30.60%) |
| Colombia | 68251.61 | 15851.60 (23.23%) | 36474.08 (53.44%) | 15925.93 (23.33%) |
| Comoros | 423.86 | 13.16 (3.10%) | 350.58 (82.71%) | 60.12 (14.18%) |
| Congo | 3680.96 | -113.51 (-3.08%) | 3077.16 (83.60%) | 717.30 (19.49%) |
| Cook Islands | 56.03 | 21.71 (38.75%) | 13.70 (24.45%) | 20.62 (36.81%) |
| Costa Rica | 8948.28 | 1724.12 (19.27%) | 4242.00 (47.41%) | 2982.16 (33.33%) |
| Cote d'Ivoire | 7385.30 | -735.52 (-9.96%) | 15846.40 (214.57%) | -7725.58 (-104.61%) |
| Croatia | 5115.44 | 2281.27 (44.60%) | -307.22 (-6.01%) | 3141.39 (61.41%) |
| Cuba | 18370.57 | 5505.00 (29.97%) | 4315.47 (23.49%) | 8550.09 (46.54%) |
| Cyprus | 1825.37 | 317.06 (17.37%) | 1179.66 (64.63%) | 328.65 (18.00%) |
| Czech Republic | 17211.72 | 4662.45 (27.09%) | 2790.27 (16.21%) | 9759.01 (56.70%) |
| Democratic Republic of the Congo | 19244.10 | -1687.88 (-8.77%) | 52510.89 (272.87%) | -31578.92 (-164.10%) |
| Denmark | 5275.17 | 699.87 (13.27%) | 971.99 (18.43%) | 3603.31 (68.31%) |
| Djibouti | 599.29 | -24.57 (-4.10%) | 583.66 (97.39%) | 40.20 (6.71%) |
| Dominica | 231.47 | 52.49 (22.68%) | 53.86 (23.27%) | 125.12 (54.06%) |
| Dominican Republic | 35694.15 | 3150.79 (8.83%) | 15421.68 (43.21%) | 17121.67 (47.97%) |
| Ecuador | 31617.84 | 4369.82 (13.82%) | 14467.65 (45.76%) | 12780.37 (40.42%) |
| Egypt | 161396.10 | 8496.04 (5.26%) | 61097.42 (37.86%) | 91802.64 (56.88%) |
| El Salvador | 13642.44 | 802.78 (5.88%) | 5210.32 (38.19%) | 7629.34 (55.92%) |
| Equatorial Guinea | 743.09 | -59.77 (-8.04%) | 1179.03 (158.67%) | -376.17 (-50.62%) |
| Eritrea | 2862.89 | 45.85 (1.60%) | 2432.34 (84.96%) | 384.71 (13.44%) |
| Estonia | 1544.91 | 350.76 (22.70%) | -147.94 (-9.58%) | 1342.09 (86.87%) |
| Ethiopia | 33085.76 | 2777.51 (8.39%) | 74457.68 (225.04%) | -44149.42 (-133.44%) |
| Federated States of Micronesia | 218.63 | 32.67 (14.94%) | 61.98 (28.35%) | 123.98 (56.71%) |
| Fiji | 1023.21 | 421.50 (41.19%) | 884.11 (86.41%) | -282.41 (-27.60%) |
| Finland | 7839.26 | 1783.10 (22.75%) | 1510.52 (19.27%) | 4545.64 (57.99%) |
| France | 47783.88 | 11612.57 (24.30%) | 12292.11 (25.72%) | 23879.19 (49.97%) |
| Gabon | 1425.37 | -110.07 (-7.72%) | 1042.67 (73.15%) | 492.77 (34.57%) |
| Georgia | 26657.39 | 1757.96 (6.59%) | 6294.35 (23.61%) | 18605.08 (69.79%) |
| Germany | 150430.87 | 14336.79 (9.53%) | 18525.08 (12.31%) | 117568.99 (78.15%) |
| Ghana | 21002.60 | 92.81 (0.44%) | 15575.83 (74.16%) | 5333.96 (25.40%) |
| Greece | 9843.27 | -149.26 (-1.52%) | 1981.21 (20.13%) | 8011.31 (81.39%) |
| Greenland | 63.99 | 21.24 (33.19%) | 3.25 (5.07%) | 39.51 (61.74%) |
| Grenada | 456.69 | 79.07 (17.31%) | 190.57 (41.73%) | 187.06 (40.96%) |
| Guam | 280.82 | 92.46 (32.92%) | 76.81 (27.35%) | 111.55 (39.72%) |
| Guatemala | 24041.25 | 1563.37 (6.50%) | 12971.13 (53.95%) | 9506.75 (39.54%) |
| Guinea | 3335.11 | -62.16 (-1.86%) | 5780.79 (173.33%) | -2383.52 (-71.47%) |
| Guinea-Bissau | 455.41 | -87.75 (-19.27%) | 1037.26 (227.76%) | -494.10 (-108.50%) |
| Guyana | 2021.82 | 607.67 (30.06%) | 407.26 (20.14%) | 1006.88 (49.80%) |
| Haiti | 15946.03 | -817.20 (-5.12%) | 12140.59 (76.14%) | 4622.64 (28.99%) |
| Honduras | 28991.19 | 501.74 (1.73%) | 17457.81 (60.22%) | 11031.64 (38.05%) |
| Hungary | 10969.08 | 2826.84 (25.77%) | 596.51 (5.44%) | 7545.72 (68.79%) |
| Iceland | 433.02 | 51.93 (11.99%) | 146.87 (33.92%) | 234.22 (54.09%) |
| India | 1592633.77 | 114481.10 (7.19%) | 935422.32 (58.73%) | 542730.35 (34.08%) |
| Indonesia | 208279.91 | 1284.47 (0.62%) | 155114.67 (74.47%) | 51880.76 (24.91%) |
| Iran | 104385.90 | 9975.55 (9.56%) | 50954.08 (48.81%) | 43456.26 (41.63%) |
| Iraq | 75089.00 | 3492.53 (4.65%) | 44171.21 (58.83%) | 27425.26 (36.52%) |
| Ireland | 7158.46 | 570.09 (7.96%) | 3230.55 (45.13%) | 3357.81 (46.91%) |
| Israel | 10339.04 | 1117.77 (10.81%) | 6278.44 (60.73%) | 2942.82 (28.46%) |
| Italy | 55810.10 | 22651.04 (40.59%) | 12488.89 (22.38%) | 20670.17 (37.04%) |
| Jamaica | 3516.40 | -34.17 (-0.97%) | 2170.22 (61.72%) | 1380.34 (39.25%) |
| Japan | 235294.71 | 104380.21 (44.36%) | 36018.82 (15.31%) | 94895.68 (40.33%) |
| Jordan | 22452.46 | 2157.54 (9.61%) | 14470.41 (64.45%) | 5824.50 (25.94%) |
| Kazakhstan | 17551.04 | 1948.13 (11.10%) | 3910.83 (22.28%) | 11692.09 (66.62%) |
| Kenya | 20324.72 | -4598.98 (-22.63%) | 36891.78 (181.51%) | -11968.09 (-58.88%) |
| Kiribati | 472.34 | 20.07 (4.25%) | 232.69 (49.26%) | 219.58 (46.49%) |
| Kuwait | 10255.80 | 1563.01 (15.24%) | 5680.87 (55.39%) | 3011.93 (29.37%) |
| Kyrgyzstan | 5068.49 | 172.62 (3.41%) | 2109.78 (41.63%) | 2786.10 (54.97%) |
| Laos | 5330.51 | -207.75 (-3.90%) | 5091.86 (95.52%) | 446.40 (8.37%) |
| Latvia | 1600.89 | 343.90 (21.48%) | -714.33 (-44.62%) | 1971.32 (123.14%) |
| Lebanon | 11696.09 | 738.46 (6.31%) | 6217.04 (53.15%) | 4740.60 (40.53%) |
| Lesotho | 1093.32 | -139.12 (-12.72%) | 556.96 (50.94%) | 675.48 (61.78%) |
| Liberia | 2903.24 | -335.67 (-11.56%) | 2320.42 (79.93%) | 918.49 (31.64%) |
| Libya | 11271.33 | 618.43 (5.49%) | 5640.40 (50.04%) | 5012.50 (44.47%) |
| Lithuania | 2649.72 | 237.88 (8.98%) | -808.00 (-30.49%) | 3219.84 (121.52%) |
| Luxembourg | 781.51 | 40.07 (5.13%) | 379.29 (48.53%) | 362.15 (46.34%) |
| Macedonia | 5331.68 | 1390.00 (26.07%) | 1360.61 (25.52%) | 2581.07 (48.41%) |
| Madagascar | 8021.63 | -600.04 (-7.48%) | 9058.00 (112.92%) | -436.33 (-5.44%) |
| Malawi | 4924.08 | -272.51 (-5.53%) | 12870.76 (261.38%) | -7674.18 (-155.85%) |
| Malaysia | 32607.67 | 3501.97 (10.74%) | 26294.79 (80.64%) | 2810.91 (8.62%) |
| Maldives | 355.30 | -107.92 (-30.38%) | 480.23 (135.16%) | -17.00 (-4.79%) |
| Mali | 15938.87 | -1292.96 (-8.11%) | 12424.16 (77.95%) | 4807.67 (30.16%) |
| Malta | 917.27 | 209.43 (22.83%) | 239.62 (26.12%) | 468.22 (51.05%) |
| Marshall Islands | 301.56 | 13.22 (4.38%) | 144.77 (48.01%) | 143.56 (47.61%) |
| Mauritania | 4410.37 | 45.60 (1.03%) | 3091.45 (70.09%) | 1273.32 (28.87%) |
| Mauritius | 3270.69 | 1091.26 (33.36%) | 1098.38 (33.58%) | 1081.05 (33.05%) |
| Mexico | 245074.38 | 59813.15 (24.41%) | 152644.16 (62.28%) | 32617.07 (13.31%) |
| Moldova | 4567.22 | 1107.62 (24.25%) | 60.42 (1.32%) | 3399.18 (74.43%) |
| Mongolia | 2566.86 | 210.41 (8.20%) | 1164.36 (45.36%) | 1192.09 (46.44%) |
| Montenegro | 926.30 | 219.09 (23.65%) | 168.54 (18.20%) | 538.67 (58.15%) |
| Morocco | 120654.20 | 17274.16 (14.32%) | 41628.16 (34.50%) | 61751.88 (51.18%) |
| Mozambique | 7464.99 | 2569.27 (34.42%) | 18239.11 (244.33%) | -13343.39 (-178.75%) |
| Myanmar | 209830.83 | 23123.45 (11.02%) | 82425.94 (39.28%) | 104281.44 (49.70%) |
| Namibia | 1059.82 | -71.67 (-6.76%) | 1074.33 (101.37%) | 57.16 (5.39%) |
| Nepal | 35999.12 | 2170.19 (6.03%) | 19975.45 (55.49%) | 13853.48 (38.48%) |
| Netherlands | 11605.91 | 3791.92 (32.67%) | 3968.96 (34.20%) | 3845.03 (33.13%) |
| New Zealand | 8090.55 | -1185.27 (-14.65%) | 5421.56 (67.01%) | 3854.25 (47.64%) |
| Nicaragua | 8489.95 | 786.36 (9.26%) | 5394.18 (63.54%) | 2309.42 (27.20%) |
| Niger | 27623.53 | -570.92 (-2.07%) | 18930.75 (68.53%) | 9263.70 (33.54%) |
| Nigeria | 67281.42 | -2033.49 (-3.02%) | 114683.20 (170.45%) | -45368.29 (-67.43%) |
| North Korea | 24595.67 | 3095.91 (12.59%) | 10371.62 (42.17%) | 11128.14 (45.24%) |
| Northern Mariana Islands | 56.37 | 30.34 (53.83%) | 12.91 (22.90%) | 13.12 (23.28%) |
| Norway | 2571.77 | 271.14 (10.54%) | 1962.66 (76.32%) | 337.96 (13.14%) |
| Oman | 3924.63 | -219.85 (-5.60%) | 2873.94 (73.23%) | 1270.53 (32.37%) |
| Pakistan | 319155.07 | -9241.55 (-2.90%) | 200955.42 (62.96%) | 127441.20 (39.93%) |
| Palestine | 6877.22 | -176.39 (-2.56%) | 4258.25 (61.92%) | 2795.36 (40.65%) |
| Panama | 5994.34 | 755.00 (12.60%) | 3217.72 (53.68%) | 2021.62 (33.73%) |
| Papua New Guinea | 34929.53 | 441.26 (1.26%) | 20380.73 (58.35%) | 14107.53 (40.39%) |
| Paraguay | 18281.33 | 1180.82 (6.46%) | 9492.43 (51.92%) | 7608.08 (41.62%) |
| Peru | 25936.72 | 1110.42 (4.28%) | 16758.92 (64.61%) | 8067.37 (31.10%) |
| Philippines | 75662.20 | 8301.11 (10.97%) | 60640.65 (80.15%) | 6720.44 (8.88%) |
| Poland | 68734.92 | 8247.57 (12.00%) | 20015.61 (29.12%) | 40471.74 (58.88%) |
| Portugal | 25078.75 | 5094.79 (20.32%) | 4827.95 (19.25%) | 15156.01 (60.43%) |
| Principality of Monaco | 53.86 | 3.60 (6.69%) | 10.78 (20.01%) | 39.48 (73.30%) |
| Puerto Rico | 10044.22 | 3805.97 (37.89%) | 1631.45 (16.24%) | 4606.80 (45.87%) |
| Qatar | 3737.60 | 180.53 (4.83%) | 2519.84 (67.42%) | 1037.24 (27.75%) |
| Republic of Nauru | 15.84 | 0.81 (5.14%) | 3.81 (24.07%) | 11.21 (70.76%) |
| Republic of Niue | 4.59 | 0.97 (21.22%) | -0.88 (-19.24%) | 4.50 (97.97%) |
| Republic of Palau | 111.61 | 15.59 (13.96%) | 41.38 (37.08%) | 54.64 (48.95%) |
| Republic of San Marino | 61.42 | 4.84 (7.88%) | 21.85 (35.57%) | 34.73 (56.55%) |
| Romania | 15258.54 | 2290.00 (15.01%) | -2663.27 (-17.45%) | 15631.81 (102.45%) |
| Russian Federation | 159292.18 | 31125.55 (19.54%) | 8655.70 (5.43%) | 119510.92 (75.03%) |
| Rwanda | 3018.36 | -42.11 (-1.40%) | 4243.28 (140.58%) | -1182.81 (-39.19%) |
| Saint Kitts and Nevis | 157.87 | 9.72 (6.16%) | 89.54 (56.72%) | 58.61 (37.12%) |
| Saint Lucia | 603.86 | 145.11 (24.03%) | 312.71 (51.79%) | 146.04 (24.18%) |
| Saint Vincent and the Grenadines | 344.00 | 96.83 (28.15%) | 116.97 (34.00%) | 130.21 (37.85%) |
| Samoa | 238.10 | 24.20 (10.16%) | 149.97 (62.98%) | 63.94 (26.85%) |
| Sao Tome and Principe | 83.23 | -14.23 (-17.10%) | 104.08 (125.06%) | -6.62 (-7.95%) |
| Saudi Arabia | 76308.17 | 3301.41 (4.33%) | 45882.48 (60.13%) | 27124.29 (35.55%) |
| Senegal | 5892.03 | -254.71 (-4.32%) | 10443.35 (177.25%) | -4296.62 (-72.92%) |
| Serbia | 17188.40 | 5438.79 (31.64%) | 1050.17 (6.11%) | 10699.44 (62.25%) |
| Seychelles | 308.85 | 24.95 (8.08%) | 108.61 (35.17%) | 175.28 (56.75%) |
| Sierra Leone | 2480.68 | -133.95 (-5.40%) | 4359.74 (175.75%) | -1745.11 (-70.35%) |
| Singapore | 13698.94 | 1858.26 (13.57%) | 10486.48 (76.55%) | 1354.20 (9.89%) |
| Slovakia | 6335.96 | 1903.26 (30.04%) | 1694.82 (26.75%) | 2737.89 (43.21%) |
| Slovenia | 1936.35 | 718.22 (37.09%) | 514.18 (26.55%) | 703.95 (36.35%) |
| Solomon Islands | 481.69 | -25.35 (-5.26%) | 651.75 (135.31%) | -144.71 (-30.04%) |
| Somalia | 6082.69 | 1494.28 (24.57%) | 12134.69 (199.50%) | -7546.29 (-124.06%) |
| South Africa | 18471.18 | -9648.66 (-52.24%) | 47750.07 (258.51%) | -19630.24 (-106.27%) |
| South Korea | 165286.04 | 54628.56 (33.05%) | 41284.48 (24.98%) | 69373.00 (41.97%) |
| South Sudan | -853.14 | -769.81 (90.23%) | 3671.10 (-430.30%) | -3754.43 (440.07%) |
| Spain | 82603.58 | 17263.30 (20.90%) | 28743.76 (34.80%) | 36596.52 (44.30%) |
| Sri Lanka | 120593.04 | 26508.88 (21.98%) | 36542.89 (30.30%) | 57541.28 (47.72%) |
| Sudan | 30837.87 | -2658.61 (-8.62%) | 20442.82 (66.29%) | 13053.66 (42.33%) |
| Suriname | 1418.33 | 227.06 (16.01%) | 604.13 (42.59%) | 587.13 (41.40%) |
| Swaziland | -155.01 | -158.13 (102.01%) | 955.59 (-616.47%) | -952.47 (614.46%) |
| Sweden | 9013.90 | 928.01 (10.30%) | 3018.17 (33.48%) | 5067.72 (56.22%) |
| Switzerland | 13441.09 | 1971.25 (14.67%) | 4669.78 (34.74%) | 6800.06 (50.59%) |
| Syria | 20480.19 | 5563.47 (27.17%) | 6841.52 (33.41%) | 8075.20 (39.43%) |
| Taiwan (Province of China) | 69030.21 | 17110.98 (24.79%) | 24583.62 (35.61%) | 27335.62 (39.60%) |
| Tajikistan | 5436.80 | -126.46 (-2.33%) | 3096.47 (56.95%) | 2466.78 (45.37%) |
| Tanzania | 17747.22 | -2102.05 (-11.84%) | 41211.62 (232.21%) | -21362.35 (-120.37%) |
| Thailand | 83029.56 | 19736.94 (23.77%) | 39256.58 (47.28%) | 24036.04 (28.95%) |
| The Bahamas | 920.92 | 206.57 (22.43%) | 396.52 (43.06%) | 317.82 (34.51%) |
| The Gambia | 3134.09 | 36.53 (1.17%) | 1844.53 (58.85%) | 1253.03 (39.98%) |
| Timor-Leste | 1544.78 | 171.82 (11.12%) | 675.39 (43.72%) | 697.57 (45.16%) |
| Togo | 2055.45 | -370.69 (-18.03%) | 4415.82 (214.83%) | -1989.68 (-96.80%) |
| Tokelau | 1.84 | 0.22 (12.23%) | 0.12 (6.79%) | 1.49 (81.03%) |
| Tonga | 178.02 | 20.37 (11.44%) | 48.73 (27.37%) | 108.92 (61.19%) |
| Trinidad and Tobago | 4199.95 | 1321.91 (31.47%) | 1656.09 (39.43%) | 1221.94 (29.09%) |
| Tunisia | 24728.95 | 3922.94 (15.86%) | 9243.32 (37.38%) | 11562.68 (46.76%) |
| Turkmenistan | 6841.97 | 526.93 (7.70%) | 2610.65 (38.16%) | 3704.39 (54.14%) |
| Tuvalu | 11.62 | 1.14 (9.82%) | 8.08 (69.51%) | 2.40 (20.70%) |
| Uganda | 11682.10 | -542.50 (-4.64%) | 13843.35 (118.50%) | -1618.76 (-13.86%) |
| Ukraine | 28894.84 | 3275.94 (11.34%) | -4406.85 (-15.25%) | 30025.76 (103.91%) |
| United Arab Emirates | 19824.24 | 3757.01 (18.95%) | 10780.35 (54.38%) | 5286.88 (26.67%) |
| United Kingdom | 115592.26 | 8244.06 (7.13%) | 24920.16 (21.56%) | 82428.04 (71.31%) |
| United States | 1055771.29 | 198056.49 (18.76%) | 258454.41 (24.48%) | 599260.39 (56.76%) |
| Uruguay | 7559.20 | 111.92 (1.48%) | 1515.43 (20.05%) | 5931.85 (78.47%) |
| Uzbekistan | 43220.17 | 4047.59 (9.37%) | 17009.35 (39.36%) | 22163.23 (51.28%) |
| Vanuatu | 342.08 | 22.65 (6.62%) | 291.40 (85.19%) | 28.02 (8.19%) |
| Venezuela | 75239.01 | 11551.02 (15.35%) | 36060.12 (47.93%) | 27627.86 (36.72%) |
| Vietnam | 72382.82 | -9811.12 (-13.55%) | 72805.28 (100.58%) | 9388.67 (12.97%) |
| Virgin Islands, U.S. | 292.09 | -28.09 (-9.62%) | 28.75 (9.84%) | 291.42 (99.77%) |
| Yemen | 18816.66 | -543.82 (-2.89%) | 13549.34 (72.01%) | 5811.14 (30.88%) |
| Zambia | 7641.87 | -643.94 (-8.43%) | 8675.55 (113.53%) | -389.75 (-5.10%) |
| Zimbabwe | 5303.33 | -527.59 (-9.95%) | 4746.60 (89.50%) | 1084.32 (20.45%) |

a. Change in incidence numbers between 1990 and 2021.a is the sum of b, c, and d

b-d. Change in incidence numbers due to change in the age structure, population number and epidemiologic changes, respectively. SDI, socio-demographic index.

# Supplementary Table 11. Contribution of population-level drivers to changes in prevalence numbers of type 2 diabetes mellitus from 1990 to 2021

|  | Overall difference^a^ | Aging^b^ | Population^c^ | Epidemiological changes^d^ |
| --- | --- | --- | --- | --- |
| Global | 372501958.13 | 44363750.69 (11.91%) | 144452970.16 (38.78%) | 183685237.28 (49.31%) |
| **Sex** | | | | |
| Male | 196187004.22 | 23849690.29 (12.16%) | 74820229.43 (38.14%) | 97517084.50 (49.71%) |
| Female | 178999714.58 | 21023021.83 (11.74%) | 70655638.30 (39.47%) | 87321054.45 (48.78%) |
| **SDI regions** | | | | |
| High SDI | 102671184.34 | 10900413.77 (10.62%) | 26920786.80 (26.22%) | 64849983.77 (63.16%) |
| High-middle SDI | 85248251.26 | 11856142.49 (13.91%) | 28773738.01 (33.75%) | 44618370.76 (52.34%) |
| Middle SDI | 119985862.48 | 22293981.97 (18.58%) | 49862921.82 (41.56%) | 47828958.69 (39.86%) |
| Low-middle SDI | 157936161.23 | -698381.83 (-0.44%) | 81727709.60 (51.75%) | 76906833.46 (48.69%) |
| Low SDI | 26894243.76 | -240817.67 (-0.90%) | 16126501.78 (59.96%) | 11008559.65 (40.93%) |
| **21 GBD regions** | | | | |
| Andean Latin America | 2447120.61 | 300035.22 (12.26%) | 1110050.17 (45.36%) | 1037035.22 (42.38%) |
| Australasia | 1386545.48 | 203285.33 (14.66%) | 546557.78 (39.42%) | 636702.37 (45.92%) |
| Caribbean | 3203404.78 | 485178.53 (15.15%) | 1167320.20 (36.44%) | 1550906.05 (48.41%) |
| Central Asia | 4889762.81 | -577552.73 (-11.81%) | 2137747.85 (43.72%) | 3329567.69 (68.09%) |
| Central Europe | 5079183.90 | 1568035.86 (30.87%) | 449539.21 (8.85%) | 3061608.83 (60.28%) |
| Central Latin America | 24071849.79 | 595935.26 (2.48%) | 16648339.35 (69.16%) | 6827575.18 (28.36%) |
| Central Sub-Saharan Africa | 2835291.71 | -18329.16 (-0.65%) | 1659949.85 (58.55%) | 1193671.02 (42.10%) |
| East Asia | 82471503.94 | 18061245.98 (21.90%) | 27480472.75 (33.32%) | 36929785.21 (44.78%) |
| Eastern Europe | 6297430.63 | 1006611.43 (15.98%) | 69177.25 (1.10%) | 5221641.94 (82.92%) |
| Eastern Sub-Saharan Africa | 14636054.57 | 254665.14 (1.74%) | 9486826.86 (64.82%) | 4894562.57 (33.44%) |
| High-income Asia Pacific | 17182600.71 | 3959403.10 (23.04%) | 3729609.08 (21.71%) | 9493588.53 (55.25%) |
| High-income North America | 43686359.60 | 6329599.18 (14.49%) | 10265633.72 (23.50%) | 27091126.70 (62.01%) |
| North Africa and Middle East | 136048006.60 | 3300498.05 (2.43%) | 64108457.13 (47.12%) | 68639051.42 (50.45%) |
| Oceania | 898291.44 | 33616.48 (3.74%) | 455961.72 (50.76%) | 408713.23 (45.50%) |
| South Asia | 201857514.09 | -255066.54 (-0.13%) | 114144033.08 (56.55%) | 87968547.55 (43.58%) |
| Southeast Asia | 29634505.15 | 4802572.75 (16.21%) | 11906795.17 (40.18%) | 12925137.23 (43.62%) |
| Southern Latin America | 3478491.13 | 328420.93 (9.44%) | 1224926.43 (35.21%) | 1925143.77 (55.34%) |
| Southern Sub-Saharan Africa | 2302344.77 | 184147.68 (8.00%) | 1096716.09 (47.63%) | 1021480.99 (44.37%) |
| Tropical Latin America | 17706132.33 | 214922.04 (1.21%) | 11764240.11 (66.44%) | 5726970.18 (32.34%) |
| Western Europe | 21368580.40 | 3401588.38 (15.92%) | 4027802.86 (18.85%) | 13939189.15 (65.23%) |
| Western Sub-Saharan Africa | 8818027.04 | -411394.67 (-4.67%) | 5260299.27 (59.65%) | 3969122.44 (45.01%) |
| **Countries/territories** | | | | |
| Afghanistan | 2197166.80 | -513231.63 (-23.36%) | 1390120.36 (63.27%) | 1320278.07 (60.09%) |
| Albania | 84837.45 | 40685.08 (47.96%) | 8704.66 (10.26%) | 35447.71 (41.78%) |
| Algeria | 7866880.05 | 359717.44 (4.57%) | 3681651.29 (46.80%) | 3825511.31 (48.63%) |
| American Samoa | 9627.91 | -382.49 (-3.97%) | 2454.35 (25.49%) | 7556.05 (78.48%) |
| Andorra | 4620.43 | 801.83 (17.35%) | 1619.49 (35.05%) | 2199.11 (47.60%) |
| Angola | 940492.74 | 3207.58 (0.34%) | 585780.19 (62.28%) | 351504.97 (37.37%) |
| Antigua and Barbuda | 9865.34 | 1373.35 (13.92%) | 4417.94 (44.78%) | 4074.05 (41.30%) |
| Argentina | 2378261.07 | 97089.05 (4.08%) | 907769.80 (38.17%) | 1373402.21 (57.75%) |
| Armenia | 119561.64 | 34842.36 (29.14%) | 9991.39 (8.36%) | 74727.90 (62.50%) |
| Australia | 1463123.67 | 90631.89 (6.19%) | 607984.89 (41.55%) | 764506.89 (52.25%) |
| Austria | 239633.86 | 32333.01 (13.49%) | 45710.67 (19.08%) | 161590.18 (67.43%) |
| Azerbaijan | 408480.90 | 39028.63 (9.55%) | 148922.94 (36.46%) | 220529.33 (53.99%) |
| Bahrain | 574856.38 | -2245.70 (-0.39%) | 356493.25 (62.01%) | 220608.83 (38.38%) |
| Bangladesh | 14367126.35 | 180205.41 (1.25%) | 7153683.05 (49.79%) | 7033237.89 (48.95%) |
| Barbados | 28747.61 | 6730.04 (23.41%) | 8817.62 (30.67%) | 13199.94 (45.92%) |
| Belarus | 212601.12 | 46403.42 (21.83%) | -307.89 (-0.14%) | 166505.59 (78.32%) |
| Belgium | 513525.75 | 66872.53 (13.02%) | 92672.29 (18.05%) | 353980.94 (68.93%) |
| Belize | 28430.11 | 1504.69 (5.29%) | 16106.74 (56.65%) | 10818.68 (38.05%) |
| Benin | 446053.60 | -7243.28 (-1.62%) | 266387.83 (59.72%) | 186909.05 (41.90%) |
| Bermuda | 5088.61 | -187.57 (-3.69%) | 1274.04 (25.04%) | 4002.14 (78.65%) |
| Bhutan | 23844.03 | 3686.51 (15.46%) | 9625.39 (40.37%) | 10532.13 (44.17%) |
| Bolivia | 588650.00 | 45134.09 (7.67%) | 282108.92 (47.92%) | 261406.99 (44.41%) |
| Bosnia and Herzegovina | 233096.39 | 97583.66 (41.86%) | -32473.11 (-13.93%) | 167985.84 (72.07%) |
| Botswana | 58382.29 | 2354.26 (4.03%) | 32075.19 (54.94%) | 23952.84 (41.03%) |
| Brazil | 16982056.66 | 210722.46 (1.24%) | 11362797.92 (66.91%) | 5408536.28 (31.85%) |
| Brunei | 63250.33 | 9911.53 (15.67%) | 24690.72 (39.04%) | 28648.08 (45.29%) |
| Bulgaria | 339467.91 | 86862.86 (25.59%) | -86479.43 (-25.47%) | 339084.48 (99.89%) |
| Burkina Faso | 456981.02 | -13995.16 (-3.06%) | 257188.66 (56.28%) | 213787.52 (46.78%) |
| Burundi | 390863.80 | 9253.75 (2.37%) | 248134.18 (63.48%) | 133475.86 (34.15%) |
| Cambodia | 585996.84 | 49698.64 (8.48%) | 238515.90 (40.70%) | 297782.30 (50.82%) |
| Cameroon | 2050724.12 | 24161.04 (1.18%) | 1268633.89 (61.86%) | 757929.19 (36.96%) |
| Canada | 3631823.53 | 618794.57 (17.04%) | 833433.15 (22.95%) | 2179595.81 (60.01%) |
| Cape Verde | 27078.45 | -1196.56 (-4.42%) | 13643.10 (50.38%) | 14631.91 (54.04%) |
| Central African Republic | 189954.06 | -107.32 (-0.06%) | 98606.56 (51.91%) | 91454.82 (48.15%) |
| Chad | 790735.58 | 2149.54 (0.27%) | 458209.38 (57.95%) | 330376.66 (41.78%) |
| Chile | 1579293.67 | 288258.90 (18.25%) | 517537.62 (32.77%) | 773497.15 (48.98%) |
| China | 79315120.15 | 17489313.46 (22.05%) | 26581630.10 (33.51%) | 35244176.59 (44.44%) |
| Colombia | 2418085.79 | 512756.64 (21.21%) | 1204984.99 (49.83%) | 700344.16 (28.96%) |
| Comoros | 47758.79 | 1801.60 (3.77%) | 27214.06 (56.98%) | 18743.12 (39.25%) |
| Congo | 158929.11 | 1421.26 (0.89%) | 86498.32 (54.43%) | 71009.52 (44.68%) |
| Cook Islands | 2973.39 | -161.75 (-5.44%) | 858.91 (28.89%) | 2276.23 (76.55%) |
| Costa Rica | 371457.97 | 54735.99 (14.74%) | 172366.64 (46.40%) | 144355.34 (38.86%) |
| Cote d'Ivoire | 739661.52 | -5214.07 (-0.70%) | 428307.00 (57.91%) | 316568.58 (42.80%) |
| Croatia | 181355.16 | 74984.76 (41.35%) | -10318.56 (-5.69%) | 116688.96 (64.34%) |
| Cuba | 775843.40 | 210572.06 (27.14%) | 162874.21 (20.99%) | 402397.13 (51.87%) |
| Cyprus | 65775.38 | 9293.21 (14.13%) | 41894.14 (63.69%) | 14588.04 (22.18%) |
| Czech Republic | 581133.25 | 144960.24 (24.94%) | 95697.01 (16.47%) | 340475.99 (58.59%) |
| Democratic Republic of the Congo | 1676786.60 | -14059.61 (-0.84%) | 976796.91 (58.25%) | 714049.30 (42.58%) |
| Denmark | 229386.99 | 27593.51 (12.03%) | 32633.58 (14.23%) | 169159.90 (73.74%) |
| Djibouti | 72976.03 | 2920.02 (4.00%) | 46245.12 (63.37%) | 23810.89 (32.63%) |
| Dominica | 5061.69 | 888.31 (17.55%) | 1008.13 (19.92%) | 3165.24 (62.53%) |
| Dominican Republic | 741487.39 | 100205.65 (13.51%) | 290153.48 (39.13%) | 351128.26 (47.35%) |
| Ecuador | 1217027.27 | 137895.10 (11.33%) | 530843.62 (43.62%) | 548288.55 (45.05%) |
| Egypt | 15640732.66 | 36223.22 (0.23%) | 5875546.67 (37.57%) | 9728962.78 (62.20%) |
| El Salvador | 523978.50 | -5659.93 (-1.08%) | 207956.64 (39.69%) | 321681.79 (61.39%) |
| Equatorial Guinea | 47084.99 | -2926.86 (-6.22%) | 32254.08 (68.50%) | 17757.77 (37.71%) |
| Eritrea | 334521.20 | 2453.99 (0.73%) | 198481.21 (59.33%) | 133586.00 (39.93%) |
| Estonia | 55933.77 | 11140.12 (19.92%) | -5313.44 (-9.50%) | 50107.09 (89.58%) |
| Ethiopia | 1147281.20 | -37733.76 (-3.29%) | 882105.33 (76.89%) | 302909.62 (26.40%) |
| Federated States of Micronesia | 9282.94 | 715.18 (7.70%) | 2293.57 (24.71%) | 6274.19 (67.59%) |
| Fiji | 90245.10 | 16371.44 (18.14%) | 24617.83 (27.28%) | 49255.83 (54.58%) |
| Finland | 319157.52 | 61669.93 (19.32%) | 51969.38 (16.28%) | 205518.21 (64.39%) |
| France | 2045053.47 | 389115.17 (19.03%) | 402546.30 (19.68%) | 1253392.00 (61.29%) |
| Gabon | 166612.46 | 6601.81 (3.96%) | 83611.30 (50.18%) | 76399.34 (45.85%) |
| Georgia | 1041075.22 | 175407.06 (16.85%) | 199981.25 (19.21%) | 665686.91 (63.94%) |
| Germany | 4308734.12 | 632190.84 (14.67%) | 387709.15 (9.00%) | 3288834.13 (76.33%) |
| Ghana | 905301.65 | 10222.90 (1.13%) | 490979.20 (54.23%) | 404099.55 (44.64%) |
| Greece | 456522.25 | 98804.17 (21.64%) | 50510.36 (11.06%) | 307207.72 (67.29%) |
| Greenland | 4043.03 | -262.93 (-6.50%) | 244.31 (6.04%) | 4061.65 (100.46%) |
| Grenada | 8378.57 | 591.87 (7.06%) | 3501.74 (41.79%) | 4284.97 (51.14%) |
| Guam | 11658.25 | 3127.84 (26.83%) | 2751.82 (23.60%) | 5778.59 (49.57%) |
| Guatemala | 978410.64 | 38052.18 (3.89%) | 477755.23 (48.83%) | 462603.22 (47.28%) |
| Guinea | 250023.55 | -11171.35 (-4.47%) | 144010.01 (57.60%) | 117184.90 (46.87%) |
| Guinea-Bissau | 52176.42 | -2668.77 (-5.11%) | 29978.51 (57.46%) | 24866.68 (47.66%) |
| Guyana | 79536.32 | 16000.11 (20.12%) | 15542.79 (19.54%) | 47993.42 (60.34%) |
| Haiti | 714023.16 | -7538.17 (-1.06%) | 427174.39 (59.83%) | 294386.94 (41.23%) |
| Honduras | 1232357.53 | -3276.37 (-0.27%) | 746283.99 (60.56%) | 489349.91 (39.71%) |
| Hungary | 400691.63 | 113122.23 (28.23%) | 19102.45 (4.77%) | 268466.95 (67.00%) |
| Iceland | 17754.23 | 1737.39 (9.79%) | 5630.11 (31.71%) | 10386.72 (58.50%) |
| India | 63901785.16 | 4207026.29 (6.58%) | 32101586.64 (50.24%) | 27593172.23 (43.18%) |
| Indonesia | 8819297.25 | 1236580.64 (14.02%) | 3809838.19 (43.20%) | 3772878.41 (42.78%) |
| Iran | 12105963.25 | 524546.96 (4.33%) | 5664550.43 (46.79%) | 5916865.86 (48.88%) |
| Iraq | 9521377.69 | 202395.67 (2.13%) | 5277614.09 (55.43%) | 4041367.93 (42.45%) |
| Ireland | 159614.77 | 19579.42 (12.27%) | 66195.90 (41.47%) | 73839.46 (46.26%) |
| Israel | 408231.02 | 39130.24 (9.59%) | 229964.61 (56.33%) | 139136.17 (34.08%) |
| Italy | 2068611.17 | 680805.90 (32.91%) | 404444.61 (19.55%) | 983360.66 (47.54%) |
| Jamaica | 147535.95 | 15622.89 (10.59%) | 58512.00 (39.66%) | 73401.06 (49.75%) |
| Japan | 8632395.29 | 3027929.12 (35.08%) | 1363144.83 (15.79%) | 4241321.34 (49.13%) |
| Jordan | 1272595.83 | -48362.92 (-3.80%) | 823503.45 (64.71%) | 497455.30 (39.09%) |
| Kazakhstan | 773860.65 | 64473.44 (8.33%) | 162080.89 (20.94%) | 547306.31 (70.72%) |
| Kenya | 360771.01 | 12039.91 (3.34%) | 256490.77 (71.10%) | 92240.34 (25.57%) |
| Kiribati | 15709.81 | 492.08 (3.13%) | 7102.41 (45.21%) | 8115.33 (51.66%) |
| Kuwait | 534248.96 | -11080.03 (-2.07%) | 321694.01 (60.21%) | 223634.98 (41.86%) |
| Kyrgyzstan | 198294.78 | 7293.03 (3.68%) | 78928.80 (39.80%) | 112072.96 (56.52%) |
| Laos | 270209.84 | 3033.96 (1.12%) | 126597.53 (46.85%) | 140578.35 (52.03%) |
| Latvia | 66791.96 | 18012.03 (26.97%) | -24423.77 (-36.57%) | 73203.71 (109.60%) |
| Lebanon | 1052937.21 | -7921.55 (-0.75%) | 568252.03 (53.97%) | 492606.73 (46.78%) |
| Lesotho | 45972.82 | -3990.62 (-8.68%) | 15179.79 (33.02%) | 34783.65 (75.66%) |
| Liberia | 133854.35 | -9916.00 (-7.41%) | 74033.95 (55.31%) | 69736.41 (52.10%) |
| Libya | 590677.87 | -43268.79 (-7.33%) | 301084.03 (50.97%) | 332862.63 (56.35%) |
| Lithuania | 89660.80 | 27293.83 (30.44%) | -18275.58 (-20.38%) | 80642.55 (89.94%) |
| Luxembourg | 33141.66 | 1546.03 (4.66%) | 14675.54 (44.28%) | 16920.09 (51.05%) |
| Macedonia | 173533.87 | 36490.23 (21.03%) | 46224.26 (26.64%) | 90819.38 (52.34%) |
| Madagascar | 348543.42 | -10323.29 (-2.96%) | 228638.40 (65.60%) | 130228.30 (37.36%) |
| Malawi | 141411.39 | -1563.31 (-1.11%) | 92119.08 (65.14%) | 50855.62 (35.96%) |
| Malaysia | 1831058.45 | 211828.52 (11.57%) | 921252.20 (50.31%) | 697977.74 (38.12%) |
| Maldives | 18438.71 | 165.07 (0.90%) | 12013.90 (65.16%) | 6259.74 (33.95%) |
| Mali | 778175.76 | -48795.13 (-6.27%) | 461198.18 (59.27%) | 365772.71 (47.00%) |
| Malta | 38154.36 | 7080.64 (18.56%) | 8419.30 (22.07%) | 22654.42 (59.38%) |
| Marshall Islands | 8976.13 | 706.86 (7.87%) | 3530.37 (39.33%) | 4738.90 (52.79%) |
| Mauritania | 139280.25 | 1740.35 (1.25%) | 85185.41 (61.16%) | 52354.49 (37.59%) |
| Mauritius | 151616.57 | 42434.87 (27.99%) | 38003.29 (25.07%) | 71178.41 (46.95%) |
| Mexico | 8115605.11 | 1773673.55 (21.86%) | 4906498.06 (60.46%) | 1435433.50 (17.69%) |
| Moldova | 172220.56 | 34864.85 (20.24%) | 2291.97 (1.33%) | 135063.75 (78.42%) |
| Mongolia | 108060.71 | 9709.86 (8.99%) | 43186.74 (39.97%) | 55164.11 (51.05%) |
| Montenegro | 35412.96 | 10169.34 (28.72%) | 5580.73 (15.76%) | 19662.89 (55.52%) |
| Morocco | 4233364.88 | 503037.38 (11.88%) | 1436825.14 (33.94%) | 2293502.36 (54.18%) |
| Mozambique | 977135.42 | -38545.14 (-3.94%) | 558441.71 (57.15%) | 457238.85 (46.79%) |
| Myanmar | 3979837.31 | 534939.36 (13.44%) | 1361964.01 (34.22%) | 2082933.94 (52.34%) |
| Namibia | 146286.77 | 5291.32 (3.62%) | 82702.40 (56.53%) | 58293.04 (39.85%) |
| Nepal | 1660270.21 | 74481.20 (4.49%) | 797176.11 (48.01%) | 788612.90 (47.50%) |
| Netherlands | 591391.85 | 144841.70 (24.49%) | 123788.41 (20.93%) | 322761.74 (54.58%) |
| New Zealand | 309190.39 | 18627.37 (6.02%) | 151083.60 (48.86%) | 139479.43 (45.11%) |
| Nicaragua | 345536.79 | 38887.05 (11.25%) | 182466.12 (52.81%) | 124183.62 (35.94%) |
| Niger | 1093987.51 | -6012.16 (-0.55%) | 674170.56 (61.63%) | 425829.11 (38.92%) |
| Nigeria | 3464121.91 | -134694.97 (-3.89%) | 2398793.94 (69.25%) | 1200022.95 (34.64%) |
| North Korea | 1271889.22 | 143619.66 (11.29%) | 435512.91 (34.24%) | 692756.65 (54.47%) |
| Northern Mariana Islands | 3404.02 | 831.44 (24.43%) | 483.47 (14.20%) | 2089.11 (61.37%) |
| Norway | 142057.35 | 18761.95 (13.21%) | 65781.58 (46.31%) | 57513.82 (40.49%) |
| Oman | 209720.19 | -17928.95 (-8.55%) | 130081.93 (62.03%) | 97567.21 (46.52%) |
| Pakistan | 21427198.93 | 403210.52 (1.88%) | 11252637.73 (52.52%) | 9771350.68 (45.60%) |
| Palestine | 840455.64 | 20217.25 (2.41%) | 477065.51 (56.76%) | 343172.88 (40.83%) |
| Panama | 244782.51 | 15979.22 (6.53%) | 124241.42 (50.76%) | 104561.87 (42.72%) |
| Papua New Guinea | 640848.29 | 11598.53 (1.81%) | 350069.05 (54.63%) | 279180.72 (43.56%) |
| Paraguay | 319881.29 | 7678.58 (2.40%) | 172726.84 (54.00%) | 139475.87 (43.60%) |
| Peru | 984180.96 | 128956.29 (13.10%) | 466083.45 (47.36%) | 389141.22 (39.54%) |
| Philippines | 3402841.59 | 466739.32 (13.72%) | 2098512.11 (61.67%) | 837590.16 (24.61%) |
| Poland | 1808008.60 | 511428.42 (28.29%) | 385726.37 (21.33%) | 910853.81 (50.38%) |
| Portugal | 983732.84 | 145300.30 (14.77%) | 181613.54 (18.46%) | 656818.99 (66.77%) |
| Principality of Monaco | 2036.86 | 92.03 (4.52%) | 395.67 (19.43%) | 1549.16 (76.06%) |
| Puerto Rico | 362789.17 | 94261.66 (25.98%) | 55847.10 (15.39%) | 212680.41 (58.62%) |
| Qatar | 257283.75 | -10993.87 (-4.27%) | 177453.46 (68.97%) | 90824.17 (35.30%) |
| Republic of Nauru | 1362.38 | -11.65 (-0.85%) | 291.32 (21.38%) | 1082.70 (79.47%) |
| Republic of Niue | 207.25 | -5.68 (-2.74%) | -46.26 (-22.32%) | 259.19 (125.06%) |
| Republic of Palau | 2699.50 | 401.66 (14.88%) | 841.15 (31.16%) | 1456.69 (53.96%) |
| Republic of San Marino | 2252.03 | 205.23 (9.11%) | 726.77 (32.27%) | 1320.03 (58.61%) |
| Romania | 520417.67 | 211596.02 (40.66%) | -55729.61 (-10.71%) | 364551.25 (70.05%) |
| Russian Federation | 5730107.70 | 1081903.96 (18.88%) | 314435.40 (5.49%) | 4333768.35 (75.63%) |
| Rwanda | 133016.19 | 4993.70 (3.75%) | 86455.29 (65.00%) | 41567.21 (31.25%) |
| Saint Kitts and Nevis | 5212.68 | 441.85 (8.48%) | 2682.62 (51.46%) | 2088.22 (40.06%) |
| Saint Lucia | 22817.02 | 4743.44 (20.79%) | 11384.93 (49.90%) | 6688.64 (29.31%) |
| Saint Vincent and the Grenadines | 12595.05 | 3004.42 (23.85%) | 4093.30 (32.50%) | 5497.33 (43.65%) |
| Samoa | 17328.75 | 1415.53 (8.17%) | 5312.22 (30.66%) | 10601.00 (61.18%) |
| Sao Tome and Principe | 6342.62 | -360.29 (-5.68%) | 3434.65 (54.15%) | 3268.26 (51.53%) |
| Saudi Arabia | 9657858.09 | 321395.22 (3.33%) | 5817739.37 (60.24%) | 3518723.49 (36.43%) |
| Senegal | 1400170.00 | 16160.05 (1.15%) | 771552.34 (55.10%) | 612457.61 (43.74%) |
| Serbia | 532343.99 | 135779.20 (25.51%) | 35517.12 (6.67%) | 361047.67 (67.82%) |
| Seychelles | 14420.62 | 1535.83 (10.65%) | 4607.37 (31.95%) | 8277.42 (57.40%) |
| Sierra Leone | 230583.04 | -5326.71 (-2.31%) | 125347.05 (54.36%) | 110562.70 (47.95%) |
| Singapore | 600870.96 | 136584.04 (22.73%) | 321263.50 (53.47%) | 143023.42 (23.80%) |
| Slovakia | 220439.81 | 57348.28 (26.02%) | 58437.75 (26.51%) | 104653.79 (47.47%) |
| Slovenia | 78787.46 | 32321.10 (41.02%) | 17086.95 (21.69%) | 29379.42 (37.29%) |
| Solomon Islands | 29597.30 | 997.13 (3.37%) | 15042.02 (50.82%) | 13558.16 (45.81%) |
| Somalia | 184381.12 | -9035.03 (-4.90%) | 119624.03 (64.88%) | 73792.12 (40.02%) |
| South Africa | 1910125.25 | 194279.18 (10.17%) | 910301.55 (47.66%) | 805544.52 (42.17%) |
| South Korea | 7007092.50 | 1794156.50 (25.60%) | 1720946.73 (24.56%) | 3491989.27 (49.84%) |
| South Sudan | 89439.59 | -4676.37 (-5.23%) | 49935.36 (55.83%) | 44180.60 (49.40%) |
| Spain | 3397536.21 | 596187.48 (17.55%) | 1039619.00 (30.60%) | 1761729.74 (51.85%) |
| Sri Lanka | 2204572.34 | 538308.76 (24.42%) | 585736.42 (26.57%) | 1080527.16 (49.01%) |
| Sudan | 2095205.68 | -89084.54 (-4.25%) | 1113154.58 (53.13%) | 1071135.63 (51.12%) |
| Suriname | 58247.52 | 9066.95 (15.57%) | 21691.75 (37.24%) | 27488.81 (47.19%) |
| Swaziland | 105791.68 | 2457.85 (2.32%) | 45324.90 (42.84%) | 58008.93 (54.83%) |
| Sweden | 406609.90 | 42122.29 (10.36%) | 105866.99 (26.04%) | 258620.62 (63.60%) |
| Switzerland | 622284.31 | 86015.75 (13.82%) | 173684.99 (27.91%) | 362583.57 (58.27%) |
| Syria | 1018618.17 | 84939.69 (8.34%) | 340471.16 (33.42%) | 593207.32 (58.24%) |
| Taiwan (Province of China) | 1793338.22 | 533318.78 (29.74%) | 528620.29 (29.48%) | 731399.16 (40.78%) |
| Tajikistan | 226181.96 | 5634.16 (2.49%) | 108316.44 (47.89%) | 112231.35 (49.62%) |
| Tanzania | 549366.18 | -32.70 (-0.01%) | 312980.82 (56.97%) | 236418.07 (43.03%) |
| Thailand | 4782245.64 | 1390397.72 (29.07%) | 1246264.17 (26.06%) | 2145583.76 (44.87%) |
| The Bahamas | 36425.01 | 6298.63 (17.29%) | 14796.91 (40.62%) | 15329.46 (42.08%) |
| The Gambia | 141570.81 | 1119.99 (0.79%) | 78391.77 (55.37%) | 62059.05 (43.84%) |
| Timor-Leste | 61839.81 | 7696.71 (12.45%) | 19274.76 (31.17%) | 34868.34 (56.38%) |
| Togo | 145124.84 | -1944.97 (-1.34%) | 92151.62 (63.50%) | 54918.19 (37.84%) |
| Tokelau | 126.96 | 10.42 (8.21%) | 4.69 (3.70%) | 111.85 (88.10%) |
| Tonga | 8532.06 | 655.99 (7.69%) | 1947.89 (22.83%) | 5928.18 (69.48%) |
| Trinidad and Tobago | 172919.05 | 49449.35 (28.60%) | 57841.99 (33.45%) | 65627.71 (37.95%) |
| Tunisia | 2230187.52 | 81458.50 (3.65%) | 870747.96 (39.04%) | 1277981.06 (57.30%) |
| Turkmenistan | 134443.93 | 13823.17 (10.28%) | 47117.72 (35.05%) | 73503.04 (54.67%) |
| Tuvalu | 1448.81 | -14.46 (-1.00%) | 492.19 (33.97%) | 971.08 (67.03%) |
| Uganda | 450599.52 | -16567.98 (-3.68%) | 290133.50 (64.39%) | 177034.00 (39.29%) |
| Ukraine | 1096552.20 | 154719.62 (14.11%) | -150341.92 (-13.71%) | 1092174.50 (99.60%) |
| United Arab Emirates | 2874900.13 | 112797.20 (3.92%) | 2121870.26 (73.81%) | 640232.67 (22.27%) |
| United Kingdom | 5409626.55 | 291684.60 (5.39%) | 998297.10 (18.45%) | 4119644.84 (76.15%) |
| United States | 40080336.03 | 5689067.60 (14.19%) | 9372329.27 (23.38%) | 25018939.16 (62.42%) |
| Uruguay | 177251.03 | 13780.61 (7.77%) | 28947.47 (16.33%) | 134522.95 (75.89%) |
| Uzbekistan | 1669540.94 | 133009.35 (7.97%) | 651542.93 (39.03%) | 884988.66 (53.01%) |
| Vanuatu | 20298.52 | 999.29 (4.92%) | 9585.89 (47.22%) | 9713.34 (47.85%) |
| Venezuela | 2044058.00 | -77666.45 (-3.80%) | 1177811.02 (57.62%) | 943913.43 (46.18%) |
| Vietnam | 4460655.34 | 740064.91 (16.59%) | 1802556.76 (40.41%) | 1918033.66 (43.00%) |
| Virgin Islands, U.S. | 14190.44 | 4710.47 (33.19%) | 717.66 (5.06%) | 8762.31 (61.75%) |
| Yemen | 852824.00 | -11199.80 (-1.31%) | 494722.07 (58.01%) | 369301.73 (43.30%) |
| Zambia | 406520.02 | -6720.70 (-1.65%) | 262444.47 (64.56%) | 150796.25 (37.09%) |
| Zimbabwe | 322587.74 | 162.37 (0.05%) | 137860.71 (42.74%) | 184564.66 (57.21%) |

a. Change in prevalence numbers between 1990 and 2021.a is the sum of b, c, and d

b-d. Change in prevalence numbers due to change in the age structure, population number and epidemiologic changes, respectively. SDI, socio-demographic index.

# Supplementary Table 12. Contribution of population-level drivers to changes in death numbers of type 2 diabetes mellitus from 1990 to 2021

|  | Overall difference^a^ | Aging^b^ | Population^c^ | Epidemiological changes^d^ |
| --- | --- | --- | --- | --- |
| Global | 975110.61 | 323503.29 (33.18%) | 554260.31 (56.84%) | 97347.00 (9.98%) |
| **Sex** | | | | |
| Male | 483746.89 | 165730.32 (34.26%) | 257290.42 (53.19%) | 60726.14 (12.55%) |
| Female | 491363.72 | 158933.36 (32.35%) | 297231.99 (60.49%) | 35198.37 (7.16%) |
| **SDI regions** | | | | |
| High SDI | 125510.53 | 117184.39 (93.37%) | 144964.26 (115.50%) | -136638.11 (-108.87%) |
| High-middle SDI | 294977.14 | 122974.01 (41.69%) | 156807.48 (53.16%) | 15195.65 (5.15%) |
| Middle SDI | 583274.20 | 231156.96 (39.63%) | 290794.81 (49.86%) | 61322.44 (10.51%) |
| Low-middle SDI | 463848.26 | 76263.04 (16.44%) | 267101.61 (57.58%) | 120483.61 (25.97%) |
| Low SDI | 86281.13 | -4305.27 (-4.99%) | 92106.81 (106.75%) | -1520.40 (-1.76%) |
| **21 GBD regions** | | | | |
| Andean Latin America | 10242.08 | 2530.86 (24.71%) | 6297.73 (61.49%) | 1413.49 (13.80%) |
| Australasia | 3883.56 | 2262.24 (58.25%) | 2915.91 (75.08%) | -1294.59 (-33.34%) |
| Caribbean | 8384.32 | 4708.12 (56.15%) | 6896.95 (82.26%) | -3220.75 (-38.41%) |
| Central Asia | 31224.45 | -2771.99 (-8.88%) | 13687.32 (43.84%) | 20309.13 (65.04%) |
| Central Europe | 14063.42 | 9973.91 (70.92%) | 1606.56 (11.42%) | 2482.95 (17.66%) |
| Central Latin America | 80182.87 | 30391.24 (37.90%) | 49179.29 (61.33%) | 612.34 (0.76%) |
| Central Sub-Saharan Africa | 13692.27 | -927.66 (-6.78%) | 15100.94 (110.29%) | -481.01 (-3.51%) |
| East Asia | 116010.11 | 81803.95 (70.51%) | 49312.90 (42.51%) | -15106.74 (-13.02%) |
| Eastern Europe | 32733.08 | 6890.37 (21.05%) | 218.46 (0.67%) | 25624.25 (78.28%) |
| Eastern Sub-Saharan Africa | 45412.93 | -4561.62 (-10.04%) | 59801.38 (131.68%) | -9826.84 (-21.64%) |
| High-income Asia Pacific | 3938.20 | 20248.01 (514.14%) | 6821.02 (173.20%) | -23130.84 (-587.35%) |
| High-income North America | 36821.68 | 24984.15 (67.85%) | 30567.42 (83.01%) | -18729.89 (-50.87%) |
| North Africa and Middle East | 159356.16 | 22596.48 (14.18%) | 111153.51 (69.75%) | 25606.16 (16.07%) |
| Oceania | 4608.68 | 465.03 (10.09%) | 3943.37 (85.56%) | 200.28 (4.35%) |
| South Asia | 596853.12 | 134308.06 (22.50%) | 334327.06 (56.01%) | 128218.00 (21.48%) |
| Southeast Asia | 129861.89 | 40229.95 (30.98%) | 74908.39 (57.68%) | 14723.55 (11.34%) |
| Southern Latin America | 3765.66 | 2647.96 (70.32%) | 5243.01 (139.23%) | -4125.31 (-109.55%) |
| Southern Sub-Saharan Africa | 28569.32 | 2665.59 (9.33%) | 13781.21 (48.24%) | 12122.52 (42.43%) |
| Tropical Latin America | 40311.40 | 20365.53 (50.52%) | 28378.16 (70.40%) | -8432.30 (-20.92%) |
| Western Europe | 7127.68 | 37806.02 (530.41%) | 16691.78 (234.18%) | -47370.13 (-664.59%) |
| Western Sub-Saharan Africa | 62709.15 | -9804.97 (-15.64%) | 59843.11 (95.43%) | 12671.01 (20.21%) |
| **Countries/territories** | | | | |
| Afghanistan | 2819.38 | -3397.30 (-120.50%) | 4332.07 (153.65%) | 1884.62 (66.85%) |
| Albania | 117.32 | 112.71 (96.07%) | 14.01 (11.95%) | -9.40 (-8.02%) |
| Algeria | 4240.84 | 965.40 (22.76%) | 2235.16 (52.71%) | 1040.29 (24.53%) |
| American Samoa | 45.09 | 25.78 (57.18%) | 10.38 (23.02%) | 8.92 (19.79%) |
| Andorra | 7.90 | 6.40 (81.04%) | 5.46 (69.18%) | -3.97 (-50.27%) |
| Angola | 4850.28 | 34.25 (0.71%) | 4987.45 (102.83%) | -171.42 (-3.53%) |
| Antigua and Barbuda | 34.38 | 0.72 (2.08%) | 40.80 (118.66%) | -7.13 (-20.74%) |
| Argentina | 2661.77 | 1471.80 (55.29%) | 5451.87 (204.82%) | -4261.90 (-160.12%) |
| Armenia | 215.63 | 316.10 (146.59%) | 48.37 (22.43%) | -148.84 (-69.02%) |
| Australia | 7948.00 | 3102.13 (39.03%) | 6519.90 (82.03%) | -1674.03 (-21.06%) |
| Austria | 207.33 | 612.75 (295.54%) | 387.34 (186.82%) | -792.76 (-382.37%) |
| Azerbaijan | 1145.53 | 178.33 (15.57%) | 564.04 (49.24%) | 403.17 (35.19%) |
| Bahrain | 1004.29 | 232.88 (23.19%) | 732.83 (72.97%) | 38.58 (3.84%) |
| Bangladesh | 27590.23 | 8203.97 (29.74%) | 16214.44 (58.77%) | 3171.82 (11.50%) |
| Barbados | 82.72 | 56.15 (67.88%) | 86.68 (104.78%) | -60.10 (-72.66%) |
| Belarus | 281.12 | 178.24 (63.40%) | -0.76 (-0.27%) | 103.64 (36.87%) |
| Belgium | -366.51 | 693.02 (-189.09%) | 295.67 (-80.67%) | -1355.19 (369.76%) |
| Belize | 158.99 | 2.72 (1.71%) | 137.25 (86.33%) | 19.02 (11.96%) |
| Benin | 1082.98 | -206.63 (-19.08%) | 1033.42 (95.42%) | 256.19 (23.66%) |
| Bermuda | 3.85 | 18.16 (471.61%) | 4.44 (115.22%) | -18.74 (-486.73%) |
| Bhutan | 128.01 | 52.48 (40.99%) | 51.46 (40.20%) | 24.07 (18.81%) |
| Bolivia | 3588.43 | 662.75 (18.47%) | 2676.99 (74.60%) | 248.70 (6.93%) |
| Bosnia and Herzegovina | 1427.88 | 804.60 (56.35%) | -159.60 (-11.18%) | 782.88 (54.83%) |
| Botswana | 452.41 | 19.29 (4.26%) | 431.32 (95.34%) | 1.81 (0.40%) |
| Brazil | 37713.43 | 19985.90 (52.99%) | 27248.33 (72.25%) | -9520.80 (-25.25%) |
| Brunei | 111.94 | 67.92 (60.67%) | 134.91 (120.52%) | -90.88 (-81.19%) |
| Bulgaria | 662.57 | 1117.86 (168.72%) | -429.75 (-64.86%) | -25.53 (-3.85%) |
| Burkina Faso | 1374.99 | -292.46 (-21.27%) | 1774.46 (129.05%) | -107.00 (-7.78%) |
| Burundi | 668.13 | -300.08 (-44.91%) | 1317.54 (197.20%) | -349.32 (-52.28%) |
| Cambodia | 2389.80 | 503.96 (21.09%) | 1743.74 (72.97%) | 142.09 (5.95%) |
| Cameroon | 6027.09 | -761.28 (-12.63%) | 5402.28 (89.63%) | 1386.10 (23.00%) |
| Canada | 2914.85 | 3800.11 (130.37%) | 3229.07 (110.78%) | -4114.33 (-141.15%) |
| Cape Verde | 186.12 | -34.30 (-18.43%) | 92.12 (49.50%) | 128.30 (68.94%) |
| Central African Republic | 865.82 | -72.31 (-8.35%) | 957.92 (110.64%) | -19.79 (-2.29%) |
| Chad | 1153.99 | -405.61 (-35.15%) | 1064.59 (92.25%) | 495.01 (42.90%) |
| Chile | 2362.03 | 1479.89 (62.65%) | 1756.49 (74.36%) | -874.35 (-37.02%) |
| China | 108107.40 | 74866.87 (69.25%) | 45032.42 (41.66%) | -11791.89 (-10.91%) |
| Colombia | 5119.57 | 3699.41 (72.26%) | 5005.39 (97.77%) | -3585.24 (-70.03%) |
| Comoros | 170.34 | 34.94 (20.51%) | 138.95 (81.57%) | -3.55 (-2.08%) |
| Congo | 1321.75 | -37.13 (-2.81%) | 1462.09 (110.62%) | -103.21 (-7.81%) |
| Cook Islands | 12.16 | 14.33 (117.81%) | 4.54 (37.38%) | -6.71 (-55.17%) |
| Costa Rica | 1124.19 | 333.99 (29.71%) | 554.05 (49.28%) | 236.15 (21.01%) |
| Cote d'Ivoire | 2728.19 | 311.82 (11.43%) | 1894.86 (69.45%) | 521.51 (19.12%) |
| Croatia | 812.66 | 651.27 (80.14%) | -41.43 (-5.10%) | 202.82 (24.96%) |
| Cuba | -309.07 | 1452.72 (-470.03%) | 666.51 (-215.65%) | -2428.30 (785.68%) |
| Cyprus | 65.60 | 325.91 (496.82%) | 413.38 (630.16%) | -673.70 (-1026.98%) |
| Czech Republic | 2418.74 | 1111.43 (45.95%) | 434.92 (17.98%) | 872.38 (36.07%) |
| Democratic Republic of the Congo | 8713.10 | -635.51 (-7.29%) | 9516.39 (109.22%) | -167.78 (-1.93%) |
| Denmark | 542.45 | 272.99 (50.32%) | 164.44 (30.32%) | 105.02 (19.36%) |
| Djibouti | 471.81 | 64.51 (13.67%) | 336.76 (71.38%) | 70.53 (14.95%) |
| Dominica | 16.16 | 5.92 (36.62%) | 9.11 (56.39%) | 1.12 (6.96%) |
| Dominican Republic | 2000.17 | 606.74 (30.33%) | 1023.94 (51.19%) | 369.49 (18.47%) |
| Ecuador | 5202.19 | 1278.68 (24.58%) | 2937.69 (56.47%) | 985.81 (18.95%) |
| Egypt | 33624.48 | 2305.08 (6.86%) | 17227.20 (51.23%) | 14092.21 (41.91%) |
| El Salvador | 2000.29 | 434.59 (21.73%) | 655.33 (32.76%) | 910.36 (45.51%) |
| Equatorial Guinea | 180.19 | -80.12 (-44.46%) | 247.70 (137.47%) | 12.61 (7.00%) |
| Eritrea | 1161.82 | 133.56 (11.50%) | 1006.63 (86.64%) | 21.63 (1.86%) |
| Estonia | 299.79 | 88.36 (29.47%) | -18.26 (-6.09%) | 229.69 (76.62%) |
| Ethiopia | 2423.90 | -131.74 (-5.44%) | 12045.66 (496.95%) | -9490.02 (-391.52%) |
| Federated States of Micronesia | 57.69 | 11.24 (19.48%) | 23.26 (40.32%) | 23.19 (40.19%) |
| Fiji | 1183.42 | 488.53 (41.28%) | 402.71 (34.03%) | 292.18 (24.69%) |
| Finland | 99.22 | 294.42 (296.74%) | 83.98 (84.64%) | -279.18 (-281.38%) |
| France | 4797.40 | 5327.14 (111.04%) | 2014.66 (41.99%) | -2544.39 (-53.04%) |
| Gabon | 535.24 | -104.80 (-19.58%) | 529.54 (98.93%) | 110.50 (20.64%) |
| Georgia | 4792.92 | 1638.80 (34.19%) | 982.97 (20.51%) | 2171.15 (45.30%) |
| Germany | 699.82 | 9458.19 (1351.52%) | 2190.44 (313.00%) | -10948.81 (-1564.52%) |
| Ghana | 8125.26 | 158.12 (1.95%) | 4979.12 (61.28%) | 2988.02 (36.77%) |
| Greece | 584.52 | 859.13 (146.98%) | 130.66 (22.35%) | -405.26 (-69.33%) |
| Greenland | 1.72 | 5.16 (299.94%) | 0.61 (35.41%) | -4.05 (-235.41%) |
| Grenada | 19.43 | -15.76 (-81.12%) | 35.91 (184.83%) | -0.72 (-3.70%) |
| Guam | 8.54 | 40.71 (476.65%) | 15.54 (182.03%) | -47.72 (-558.74%) |
| Guatemala | 6095.48 | 693.06 (11.37%) | 2421.71 (39.73%) | 2980.70 (48.90%) |
| Guinea | 1139.04 | -398.57 (-34.99%) | 1063.04 (93.33%) | 474.57 (41.66%) |
| Guinea-Bissau | 170.82 | -50.97 (-29.84%) | 178.58 (104.54%) | 43.21 (25.30%) |
| Guyana | 331.38 | 180.36 (54.43%) | 106.74 (32.21%) | 44.28 (13.36%) |
| Haiti | 2397.91 | -243.89 (-10.17%) | 3091.38 (128.92%) | -449.57 (-18.75%) |
| Honduras | 2096.24 | 109.41 (5.22%) | 1199.33 (57.21%) | 787.51 (37.57%) |
| Hungary | 930.05 | 697.56 (75.00%) | 67.16 (7.22%) | 165.34 (17.78%) |
| Iceland | 15.35 | 9.08 (59.16%) | 13.77 (89.73%) | -7.51 (-48.90%) |
| India | 359502.73 | 81988.33 (22.81%) | 191659.79 (53.31%) | 85854.61 (23.88%) |
| Indonesia | 44162.69 | 9413.85 (21.32%) | 23208.74 (52.55%) | 11540.10 (26.13%) |
| Iran | 11589.90 | 2941.08 (25.38%) | 5686.53 (49.06%) | 2962.28 (25.56%) |
| Iraq | 6521.59 | -256.07 (-3.93%) | 5971.39 (91.56%) | 806.28 (12.36%) |
| Ireland | 27.41 | 154.27 (562.81%) | 250.14 (912.58%) | -376.99 (-1375.39%) |
| Israel | 1464.05 | 544.84 (37.21%) | 1152.24 (78.70%) | -233.03 (-15.92%) |
| Italy | 1078.26 | 10060.48 (933.03%) | 2551.07 (236.59%) | -11533.29 (-1069.62%) |
| Jamaica | 929.49 | 182.20 (19.60%) | 723.40 (77.83%) | 23.89 (2.57%) |
| Japan | -1484.78 | 10956.66 (-737.93%) | 1776.58 (-119.65%) | -14218.02 (957.58%) |
| Jordan | 1650.70 | 382.13 (23.15%) | 1929.67 (116.90%) | -661.10 (-40.05%) |
| Kazakhstan | 598.19 | 141.58 (23.67%) | 242.31 (40.51%) | 214.30 (35.82%) |
| Kenya | 5043.22 | -53.26 (-1.06%) | 3817.75 (75.70%) | 1278.73 (25.36%) |
| Kiribati | 287.21 | 28.27 (9.84%) | 166.65 (58.02%) | 92.29 (32.13%) |
| Kuwait | 499.38 | 133.47 (26.73%) | 340.35 (68.15%) | 25.56 (5.12%) |
| Kyrgyzstan | 306.69 | -1.34 (-0.44%) | 175.46 (57.21%) | 132.56 (43.22%) |
| Laos | 808.03 | -2.35 (-0.29%) | 892.27 (110.42%) | -81.89 (-10.13%) |
| Latvia | 354.27 | 137.68 (38.86%) | -93.20 (-26.31%) | 309.80 (87.45%) |
| Lebanon | 1171.56 | 467.43 (39.90%) | 1009.75 (86.19%) | -305.62 (-26.09%) |
| Lesotho | 884.10 | -150.96 (-17.07%) | 358.44 (40.54%) | 676.62 (76.53%) |
| Liberia | 716.95 | -246.50 (-34.38%) | 693.48 (96.73%) | 269.98 (37.66%) |
| Libya | 899.79 | 44.16 (4.91%) | 506.61 (56.30%) | 349.02 (38.79%) |
| Lithuania | 393.21 | 139.24 (35.41%) | -53.24 (-13.54%) | 307.22 (78.13%) |
| Luxembourg | 15.58 | 18.44 (118.38%) | 60.59 (388.93%) | -63.46 (-407.33%) |
| Macedonia | 1011.92 | 332.17 (32.83%) | 269.95 (26.68%) | 409.80 (40.50%) |
| Madagascar | 2601.43 | -727.79 (-27.98%) | 3357.78 (129.07%) | -28.55 (-1.10%) |
| Malawi | 2411.22 | -206.54 (-8.57%) | 2358.54 (97.82%) | 259.22 (10.75%) |
| Malaysia | 2771.11 | 1123.54 (40.54%) | 3001.67 (108.32%) | -1354.10 (-48.87%) |
| Maldives | 25.14 | 1.30 (5.19%) | 61.11 (243.09%) | -37.27 (-148.27%) |
| Mali | 3062.21 | -477.74 (-15.60%) | 2821.34 (92.13%) | 718.60 (23.47%) |
| Malta | 47.75 | 87.24 (182.70%) | 43.95 (92.04%) | -83.43 (-174.73%) |
| Marshall Islands | 53.50 | 8.59 (16.05%) | 25.45 (47.57%) | 19.46 (36.38%) |
| Mauritania | 3500.28 | 99.58 (2.84%) | 2410.17 (68.86%) | 990.53 (28.30%) |
| Mauritius | 1527.30 | 550.97 (36.07%) | 359.79 (23.56%) | 616.55 (40.37%) |
| Mexico | 77065.01 | 28873.31 (37.47%) | 52324.62 (67.90%) | -4132.92 (-5.36%) |
| Moldova | 322.44 | 150.06 (46.54%) | 5.72 (1.77%) | 166.65 (51.69%) |
| Mongolia | 116.59 | 17.22 (14.77%) | 84.73 (72.67%) | 14.64 (12.56%) |
| Montenegro | 159.65 | 61.05 (38.24%) | 21.80 (13.65%) | 76.80 (48.11%) |
| Morocco | 8800.49 | 1522.30 (17.30%) | 3471.89 (39.45%) | 3806.30 (43.25%) |
| Mozambique | 2825.46 | -712.76 (-25.23%) | 2636.16 (93.30%) | 902.06 (31.93%) |
| Myanmar | 18958.32 | 6918.36 (36.49%) | 13250.35 (69.89%) | -1210.38 (-6.38%) |
| Namibia | 792.92 | 13.10 (1.65%) | 616.90 (77.80%) | 162.93 (20.55%) |
| Nepal | 7428.28 | 1495.75 (20.14%) | 3745.37 (50.42%) | 2187.16 (29.44%) |
| Netherlands | 5827.04 | -276.99 (-4.75%) | 1403.85 (24.09%) | 4700.18 (80.66%) |
| New Zealand | 1124.31 | 318.76 (28.35%) | 898.58 (79.92%) | -93.03 (-8.27%) |
| Nicaragua | 1040.88 | 211.73 (20.34%) | 667.36 (64.11%) | 161.80 (15.54%) |
| Niger | 11038.17 | -236.74 (-2.14%) | 8606.93 (77.97%) | 2667.97 (24.17%) |
| Nigeria | 14197.85 | -4346.77 (-30.62%) | 17988.99 (126.70%) | 555.63 (3.91%) |
| North Korea | 2509.05 | 1075.99 (42.88%) | 1293.77 (51.56%) | 139.29 (5.55%) |
| Northern Mariana Islands | 18.83 | 17.93 (95.22%) | 2.75 (14.60%) | -1.85 (-9.82%) |
| Norway | 111.96 | 73.81 (65.92%) | 156.17 (139.48%) | -118.01 (-105.41%) |
| Oman | -244.02 | -330.95 (135.62%) | 1179.77 (-483.47%) | -1092.85 (447.85%) |
| Pakistan | 49696.93 | -8593.17 (-17.29%) | 37399.32 (75.25%) | 20890.78 (42.04%) |
| Palestine | 1113.59 | -185.81 (-16.69%) | 1289.48 (115.79%) | 9.92 (0.89%) |
| Panama | 983.00 | 287.03 (29.20%) | 496.16 (50.47%) | 199.81 (20.33%) |
| Papua New Guinea | 2564.26 | 60.67 (2.37%) | 2604.35 (101.56%) | -100.77 (-3.93%) |
| Paraguay | 2597.97 | 248.02 (9.55%) | 1194.85 (45.99%) | 1155.11 (44.46%) |
| Peru | 4258.78 | 1034.89 (24.30%) | 2469.25 (57.98%) | 754.64 (17.72%) |
| Philippines | 31971.38 | 5607.95 (17.54%) | 18294.71 (57.22%) | 8068.73 (25.24%) |
| Poland | 4304.99 | 2782.08 (64.62%) | 1258.02 (29.22%) | 264.89 (6.15%) |
| Portugal | 1804.96 | 2856.15 (158.24%) | 1064.42 (58.97%) | -2115.61 (-117.21%) |
| Principality of Monaco | 2.59 | 0.56 (21.62%) | 1.46 (56.22%) | 0.58 (22.36%) |
| Puerto Rico | 1301.87 | 1800.81 (138.32%) | 379.82 (29.17%) | -878.76 (-67.50%) |
| Qatar | 318.84 | 11.82 (3.71%) | 362.96 (113.84%) | -55.94 (-17.54%) |
| Republic of Nauru | 4.13 | 0.66 (16.10%) | 1.52 (36.73%) | 1.95 (47.22%) |
| Republic of Niue | 1.08 | 0.17 (15.93%) | -0.37 (-34.54%) | 1.28 (118.33%) |
| Republic of Palau | 13.17 | 5.45 (41.41%) | 5.42 (41.18%) | 2.29 (17.39%) |
| Republic of San Marino | -1.40 | 2.71 (-193.86%) | 5.09 (-363.29%) | -9.20 (657.29%) |
| Romania | 758.26 | 939.80 (123.94%) | -145.86 (-19.24%) | -35.68 (-4.71%) |
| Russian Federation | 46037.65 | 7660.49 (16.64%) | 1441.92 (3.13%) | 36935.25 (80.23%) |
| Rwanda | 1276.58 | 104.96 (8.22%) | 2210.16 (173.13%) | -1038.53 (-81.35%) |
| Saint Kitts and Nevis | 7.62 | -7.06 (-92.66%) | 29.80 (391.08%) | -15.12 (-198.48%) |
| Saint Lucia | -640.42 | -45.57 (7.12%) | 427.00 (-66.67%) | -1021.84 (159.56%) |
| Saint Vincent and the Grenadines | 43.33 | 41.03 (94.70%) | 45.13 (104.16%) | -42.83 (-98.85%) |
| Samoa | 65.00 | 15.38 (23.66%) | 32.77 (50.42%) | 16.85 (25.92%) |
| Sao Tome and Principe | 9.50 | -4.18 (-44.02%) | 9.48 (99.75%) | 4.20 (44.23%) |
| Saudi Arabia | 6050.16 | -316.71 (-5.23%) | 5185.84 (85.71%) | 1181.04 (19.52%) |
| Senegal | 2140.74 | 10.55 (0.49%) | 1495.42 (69.86%) | 634.77 (29.65%) |
| Serbia | 2209.91 | 1887.53 (85.41%) | 192.61 (8.72%) | 129.76 (5.87%) |
| Seychelles | 25.92 | 2.29 (8.83%) | 13.66 (52.69%) | 9.97 (38.48%) |
| Sierra Leone | 615.59 | -165.95 (-26.96%) | 557.97 (90.64%) | 223.57 (36.32%) |
| Singapore | -260.14 | 369.02 (-141.86%) | 463.94 (-178.34%) | -1093.10 (420.20%) |
| Slovakia | 243.33 | 299.07 (122.91%) | 213.43 (87.71%) | -269.17 (-110.62%) |
| Slovenia | 116.04 | -73.33 (-63.19%) | 86.48 (74.52%) | 102.90 (88.67%) |
| Solomon Islands | 256.94 | 15.17 (5.91%) | 181.38 (70.59%) | 60.39 (23.50%) |
| Somalia | 1661.97 | -124.04 (-7.46%) | 1758.37 (105.80%) | 27.65 (1.66%) |
| South Africa | 24002.39 | 2910.12 (12.12%) | 11166.61 (46.52%) | 9925.66 (41.35%) |
| South Korea | 5010.80 | 7269.96 (145.09%) | 3504.81 (69.95%) | -5763.98 (-115.03%) |
| South Sudan | 780.90 | -160.51 (-20.55%) | 669.07 (85.68%) | 272.34 (34.88%) |
| Spain | -435.44 | 7586.42 (-1742.24%) | 4681.42 (-1075.10%) | -12703.28 (2917.34%) |
| Sri Lanka | 8583.40 | 3974.18 (46.30%) | 2996.97 (34.92%) | 1612.25 (18.78%) |
| Sudan | 2092.10 | -329.62 (-15.76%) | 1673.06 (79.97%) | 748.66 (35.79%) |
| Suriname | 163.15 | 56.66 (34.73%) | 95.68 (58.64%) | 10.82 (6.63%) |
| Swaziland | 387.40 | 13.97 (3.61%) | 222.74 (57.50%) | 150.68 (38.90%) |
| Sweden | 488.03 | 405.56 (83.10%) | 343.00 (70.28%) | -260.53 (-53.38%) |
| Switzerland | -370.51 | 539.63 (-145.64%) | 450.55 (-121.60%) | -1360.68 (367.25%) |
| Syria | 1329.46 | 625.35 (47.04%) | 652.82 (49.10%) | 51.29 (3.86%) |
| Taiwan (Province of China) | 7898.03 | 7633.75 (96.65%) | 4436.00 (56.17%) | -4171.73 (-52.82%) |
| Tajikistan | 449.83 | -30.69 (-6.82%) | 426.29 (94.77%) | 54.23 (12.05%) |
| Tanzania | 4838.50 | -206.46 (-4.27%) | 5069.71 (104.78%) | -24.74 (-0.51%) |
| Thailand | 16418.48 | 10991.01 (66.94%) | 6544.54 (39.86%) | -1117.07 (-6.80%) |
| The Bahamas | 97.69 | 54.69 (55.98%) | 88.11 (90.20%) | -45.11 (-46.18%) |
| The Gambia | 444.40 | 24.81 (5.58%) | 277.13 (62.36%) | 142.46 (32.06%) |
| Timor-Leste | 186.47 | 72.64 (38.96%) | 89.74 (48.13%) | 24.08 (12.91%) |
| Togo | 869.57 | 45.53 (5.24%) | 598.10 (68.78%) | 225.95 (25.98%) |
| Tokelau | 0.26 | 0.13 (50.38%) | 0.03 (11.54%) | 0.10 (38.08%) |
| Tonga | 58.12 | 18.19 (31.29%) | 20.48 (35.24%) | 19.45 (33.46%) |
| Trinidad and Tobago | 822.19 | 688.15 (83.70%) | 575.57 (70.00%) | -441.52 (-53.70%) |
| Tunisia | 1604.23 | 458.84 (28.60%) | 658.60 (41.05%) | 486.79 (30.34%) |
| Turkmenistan | 661.96 | 89.32 (13.49%) | 245.16 (37.03%) | 327.48 (49.47%) |
| Tuvalu | 4.35 | 1.01 (23.20%) | 2.57 (59.10%) | 0.77 (17.68%) |
| Uganda | 5380.98 | -842.29 (-15.65%) | 5529.15 (102.75%) | 694.12 (12.90%) |
| Ukraine | -186.83 | 362.69 (-194.13%) | -195.89 (104.85%) | -353.62 (189.28%) |
| United Arab Emirates | 1562.48 | 408.65 (26.15%) | 1515.58 (97.00%) | -361.75 (-23.15%) |
| United Kingdom | -2497.12 | 2514.19 (-100.68%) | 2483.57 (-99.46%) | -7494.89 (300.14%) |
| United States | 34011.75 | 21428.76 (63.00%) | 27321.25 (80.33%) | -14738.26 (-43.33%) |
| Uruguay | 299.70 | 179.43 (59.87%) | 152.88 (51.01%) | -32.61 (-10.88%) |
| Uzbekistan | 7564.88 | 725.50 (9.59%) | 3027.93 (40.03%) | 3811.45 (50.38%) |
| Vanuatu | 100.69 | 14.27 (14.17%) | 68.46 (67.99%) | 17.96 (17.84%) |
| Venezuela | 13271.01 | 5159.93 (38.88%) | 5758.62 (43.39%) | 2352.45 (17.73%) |
| Vietnam | 31480.57 | 6429.58 (20.42%) | 19613.96 (62.30%) | 5437.03 (17.27%) |
| Virgin Islands, U.S. | 46.16 | 45.43 (98.42%) | 2.76 (5.98%) | -2.03 (-4.40%) |
| Yemen | 1247.02 | -52.29 (-4.19%) | 1116.17 (89.51%) | 183.14 (14.69%) |
| Zambia | 2438.24 | -379.71 (-15.57%) | 2923.14 (119.89%) | -105.20 (-4.31%) |
| Zimbabwe | 3946.36 | -190.60 (-4.83%) | 2016.21 (51.09%) | 2120.76 (53.74%) |

a. Change in death numbers between 1990 and 2021.a is the sum of b, c, and d

b-d. Change in death numbers due to change in the age structure, population number and epidemiologic changes, respectively. SDI, socio-demographic index.

# Supplementary Table 13. Contribution of population-level drivers to changes in DALYs numbers of type 2 diabetes mellitus from 1990 to 2021

|  | Overall difference^a^ | Aging^b^ | Population^c^ | Epidemiological changes^d^ |
| --- | --- | --- | --- | --- |
| Global | 50224094.27 | 9867166.48 (19.65%) | 24075083.56 (47.94%) | 16281844.23 (32.42%) |
| **Sex** | | | | |
| Male | 25946085.80 | 5221218.76 (20.12%) | 11930249.72 (45.98%) | 8794617.32 (33.90%) |
| Female | 24278008.47 | 4677106.06 (19.26%) | 12144437.86 (50.02%) | 7456464.55 (30.71%) |
| **SDI regions** | | | | |
| High SDI | 10000854.11 | 2007414.04 (20.07%) | 4374067.68 (43.74%) | 3619372.40 (36.19%) |
| High-middle SDI | 11337591.95 | 2639845.45 (23.28%) | 5079196.10 (44.80%) | 3618550.40 (31.92%) |
| Middle SDI | 22922836.46 | 6197146.35 (27.03%) | 11022183.97 (48.08%) | 5703506.13 (24.88%) |
| Low-middle SDI | 16896703.72 | 1755476.14 (10.39%) | 9280074.29 (54.92%) | 5861153.30 (34.69%) |
| Low SDI | 4031312.12 | -189097.60 (-4.69%) | 3498804.96 (86.79%) | 721604.76 (17.90%) |
| **21 GBD regions** | | | | |
| Andean Latin America | 433797.66 | 77417.03 (17.85%) | 241493.98 (55.67%) | 114886.65 (26.48%) |
| Australasia | 160698.73 | 45332.01 (28.21%) | 91415.24 (56.89%) | 23951.48 (14.90%) |
| Caribbean | 494729.13 | 140991.45 (28.50%) | 272844.53 (55.15%) | 80893.15 (16.35%) |
| Central Asia | 801098.81 | -117850.46 (-14.71%) | 364977.07 (45.56%) | 553972.19 (69.15%) |
| Central Europe | 682175.33 | 313619.13 (45.97%) | 75095.40 (11.01%) | 293460.80 (43.02%) |
| Central Latin America | 3217190.37 | 926990.93 (28.81%) | 1917915.88 (59.61%) | 372283.57 (11.57%) |
| Central Sub-Saharan Africa | 636263.98 | -27904.71 (-4.39%) | 565357.48 (88.86%) | 98811.21 (15.53%) |
| East Asia | 7875187.55 | 3041177.92 (38.62%) | 2991848.60 (37.99%) | 1842161.02 (23.39%) |
| Eastern Europe | 1195640.01 | 241499.66 (20.20%) | 10782.76 (0.90%) | 943357.59 (78.90%) |
| Eastern Sub-Saharan Africa | 1533292.53 | -104034.76 (-6.79%) | 1768917.35 (115.37%) | -131590.06 (-8.58%) |
| High-income Asia Pacific | 1401234.13 | 574142.83 (40.97%) | 415264.52 (29.64%) | 411826.78 (29.39%) |
| High-income North America | 3981820.96 | 916877.55 (23.03%) | 1338517.25 (33.62%) | 1726426.16 (43.36%) |
| North Africa and Middle East | 9931390.51 | 930353.35 (9.37%) | 5389533.31 (54.27%) | 3611503.85 (36.36%) |
| Oceania | -79640.21 | 13881.74 (-17.43%) | 343769.22 (-431.65%) | -437291.17 (549.08%) |
| South Asia | 24739480.88 | 3408235.00 (13.78%) | 13523077.62 (54.66%) | 7808168.26 (31.56%) |
| Southeast Asia | 5568111.60 | 1357879.26 (24.39%) | 2900391.85 (52.09%) | 1309840.48 (23.52%) |
| Southern Latin America | 328031.03 | 65631.55 (20.01%) | 203280.82 (61.97%) | 59118.65 (18.02%) |
| Southern Sub-Saharan Africa | 904460.05 | 92750.84 (10.25%) | 432536.73 (47.82%) | 379172.48 (41.92%) |
| Tropical Latin America | 1692511.21 | 597505.16 (35.30%) | 1116898.41 (65.99%) | -21892.36 (-1.29%) |
| Western Europe | 1502934.17 | 735648.57 (48.95%) | 572674.94 (38.10%) | 194610.65 (12.95%) |
| Western Sub-Saharan Africa | 2358876.11 | -197741.15 (-8.38%) | 1900611.83 (80.57%) | 656005.43 (27.81%) |
| **Countries/territories** | | | | |
| Afghanistan | 235198.94 | -122639.17 (-52.14%) | 215358.94 (91.56%) | 142479.17 (60.58%) |
| Albania | 10289.26 | 6323.08 (61.45%) | 1070.50 (10.40%) | 2895.68 (28.14%) |
| Algeria | 352125.19 | 44680.74 (12.69%) | 161561.26 (45.88%) | 145883.20 (41.43%) |
| American Samoa | 1742.56 | 689.66 (39.58%) | 376.03 (21.58%) | 676.87 (38.84%) |
| Andorra | 451.26 | 153.94 (34.11%) | 216.98 (48.08%) | 80.34 (17.80%) |
| Angola | 210034.73 | 832.28 (0.40%) | 182688.67 (86.98%) | 26513.78 (12.62%) |
| Antigua and Barbuda | 1692.80 | 330.46 (19.52%) | 1191.49 (70.39%) | 170.86 (10.09%) |
| Argentina | 225685.46 | 22104.72 (9.79%) | 176451.89 (78.18%) | 27128.85 (12.02%) |
| Armenia | 12397.03 | 8872.37 (71.57%) | 1954.55 (15.77%) | 1570.11 (12.67%) |
| Australia | 199364.88 | 35837.74 (17.98%) | 132019.77 (66.22%) | 31507.36 (15.80%) |
| Austria | 20326.28 | 10647.35 (52.38%) | 10378.48 (51.06%) | -699.54 (-3.44%) |
| Azerbaijan | 66020.20 | 9698.33 (14.69%) | 27909.13 (42.27%) | 28412.73 (43.04%) |
| Bahrain | 40606.15 | 8853.08 (21.80%) | 25805.06 (63.55%) | 5948.01 (14.65%) |
| Bangladesh | 1151618.35 | 232348.40 (20.18%) | 599350.23 (52.04%) | 319919.72 (27.78%) |
| Barbados | 4291.16 | 1988.92 (46.35%) | 2520.22 (58.73%) | -217.99 (-5.08%) |
| Belarus | 21947.20 | 7215.49 (32.88%) | -43.51 (-0.20%) | 14775.22 (67.32%) |
| Belgium | 35589.79 | 14301.27 (40.18%) | 12473.21 (35.05%) | 8815.31 (24.77%) |
| Belize | 6621.77 | 454.63 (6.87%) | 4644.31 (70.14%) | 1522.83 (23.00%) |
| Benin | 57728.23 | -4667.37 (-8.09%) | 42511.14 (73.64%) | 19884.46 (34.44%) |
| Bermuda | 474.17 | 460.79 (97.18%) | 156.76 (33.06%) | -143.38 (-30.24%) |
| Bhutan | 4421.02 | 1229.98 (27.82%) | 1941.72 (43.92%) | 1249.31 (28.26%) |
| Bolivia | 126670.49 | 17381.08 (13.72%) | 85564.52 (67.55%) | 23724.89 (18.73%) |
| Bosnia and Herzegovina | 48507.02 | 24330.44 (50.16%) | -6384.62 (-13.16%) | 30561.20 (63.00%) |
| Botswana | 16323.09 | 579.70 (3.55%) | 13488.11 (82.63%) | 2255.28 (13.82%) |
| Brazil | 1604982.87 | 587556.41 (36.61%) | 1076293.52 (67.06%) | -58867.06 (-3.67%) |
| Brunei | 7471.55 | 2425.36 (32.46%) | 4983.97 (66.71%) | 62.23 (0.83%) |
| Bulgaria | 33560.94 | 21112.59 (62.91%) | -16056.77 (-47.84%) | 28505.13 (84.94%) |
| Burkina Faso | 68739.61 | -10104.92 (-14.70%) | 64066.77 (93.20%) | 14777.76 (21.50%) |
| Burundi | 29713.34 | -5881.05 (-19.79%) | 41926.05 (141.10%) | -6331.66 (-21.31%) |
| Cambodia | 107825.33 | 16910.46 (15.68%) | 66539.60 (61.71%) | 24375.27 (22.61%) |
| Cameroon | 217321.51 | -16999.88 (-7.82%) | 170788.38 (78.59%) | 63533.00 (29.23%) |
| Canada | 318558.82 | 98938.59 (31.06%) | 115315.21 (36.20%) | 104305.02 (32.74%) |
| Cape Verde | 6452.13 | -419.21 (-6.50%) | 3149.71 (48.82%) | 3721.64 (57.68%) |
| Central African Republic | 42008.31 | -683.15 (-1.63%) | 34858.80 (82.98%) | 7832.65 (18.65%) |
| Chad | 54562.39 | -10319.06 (-18.91%) | 40944.14 (75.04%) | 23937.31 (43.87%) |
| Chile | 152495.70 | 45921.97 (30.11%) | 72607.33 (47.61%) | 33966.39 (22.27%) |
| China | 7500151.87 | 2860108.87 (38.13%) | 2823319.32 (37.64%) | 1816723.68 (24.22%) |
| Colombia | 353333.46 | 117504.52 (33.26%) | 241334.95 (68.30%) | -5506.00 (-1.56%) |
| Comoros | 5629.14 | 754.43 (13.40%) | 4218.97 (74.95%) | 655.75 (11.65%) |
| Congo | 51830.41 | 1375.94 (2.65%) | 46647.69 (90.00%) | 3806.77 (7.34%) |
| Cook Islands | 465.42 | 341.31 (73.33%) | 148.34 (31.87%) | -24.23 (-5.21%) |
| Costa Rica | 55413.07 | 12313.38 (22.22%) | 27371.38 (49.40%) | 15728.31 (28.38%) |
| Cote d'Ivoire | 126020.79 | 8929.99 (7.09%) | 79531.46 (63.11%) | 37559.34 (29.80%) |
| Croatia | 28013.40 | 17232.04 (61.51%) | -1743.26 (-6.22%) | 12524.62 (44.71%) |
| Cuba | 68335.07 | 47799.55 (69.95%) | 28837.75 (42.20%) | -8302.23 (-12.15%) |
| Cyprus | 5760.61 | 4577.67 (79.47%) | 10119.23 (175.66%) | -8936.29 (-155.13%) |
| Czech Republic | 89078.97 | 30746.87 (34.52%) | 16984.19 (19.07%) | 41347.92 (46.42%) |
| Democratic Republic of the Congo | 390375.00 | -20970.48 (-5.37%) | 347564.01 (89.03%) | 63781.47 (16.34%) |
| Denmark | 20431.58 | 6006.22 (29.40%) | 4929.96 (24.13%) | 9495.39 (46.47%) |
| Djibouti | 9858.50 | 1153.05 (11.70%) | 6878.95 (69.78%) | 1826.51 (18.53%) |
| Dominica | 887.72 | 276.69 (31.17%) | 294.09 (33.13%) | 316.94 (35.70%) |
| Dominican Republic | 118940.06 | 22065.21 (18.55%) | 52674.21 (44.29%) | 44200.64 (37.16%) |
| Ecuador | 208723.27 | 35663.14 (17.09%) | 108495.54 (51.98%) | 64564.58 (30.93%) |
| Egypt | 1301847.26 | 81006.02 (6.22%) | 599710.52 (46.07%) | 621130.72 (47.71%) |
| El Salvador | 72002.58 | 11529.75 (16.01%) | 24900.79 (34.58%) | 35572.05 (49.40%) |
| Equatorial Guinea | 8170.76 | -2574.43 (-31.51%) | 9124.66 (111.67%) | 1620.53 (19.83%) |
| Eritrea | 40649.98 | 2052.00 (5.05%) | 33954.11 (83.53%) | 4643.88 (11.42%) |
| Estonia | 9664.87 | 2116.53 (21.90%) | -812.42 (-8.41%) | 8360.76 (86.51%) |
| Ethiopia | 121330.92 | -33418.70 (-27.54%) | 408084.62 (336.34%) | -253335.00 (-208.80%) |
| Federated States of Micronesia | 2387.41 | 493.32 (20.66%) | 786.81 (32.96%) | 1107.28 (46.38%) |
| Fiji | 38339.17 | 13692.91 (35.72%) | 13193.70 (34.41%) | 11452.56 (29.87%) |
| Finland | 25512.96 | 9301.37 (36.46%) | 5276.00 (20.68%) | 10935.59 (42.86%) |
| France | 195524.92 | 89654.26 (45.85%) | 58059.50 (29.69%) | 47811.16 (24.45%) |
| Gabon | 20254.51 | -1110.25 (-5.48%) | 15569.45 (76.87%) | 5795.31 (28.61%) |
| Georgia | 178827.30 | 43964.01 (24.58%) | 37810.99 (21.14%) | 97052.31 (54.27%) |
| Germany | 284230.16 | 171247.97 (60.25%) | 63029.40 (22.18%) | 49952.78 (17.57%) |
| Ghana | 284295.58 | 4644.90 (1.63%) | 169455.19 (59.61%) | 110195.49 (38.76%) |
| Greece | 45767.35 | 19019.82 (41.56%) | 6449.18 (14.09%) | 20298.35 (44.35%) |
| Greenland | 232.70 | 154.78 (66.51%) | 23.22 (9.98%) | 54.70 (23.51%) |
| Grenada | 1490.31 | 47.53 (3.19%) | 1118.74 (75.07%) | 324.04 (21.74%) |
| Guam | 1388.12 | 999.86 (72.03%) | 621.02 (44.74%) | -232.75 (-16.77%) |
| Guatemala | 242577.22 | 16385.44 (6.75%) | 104277.34 (42.99%) | 121914.44 (50.26%) |
| Guinea | 46378.30 | -10940.07 (-23.59%) | 36244.53 (78.15%) | 21073.83 (45.44%) |
| Guinea-Bissau | 8559.46 | -1350.46 (-15.78%) | 7015.38 (81.96%) | 2894.55 (33.82%) |
| Guyana | 15218.37 | 5888.63 (38.69%) | 4100.91 (26.95%) | 5228.84 (34.36%) |
| Haiti | 127994.85 | -6161.16 (-4.81%) | 122142.21 (95.43%) | 12013.80 (9.39%) |
| Honduras | 103304.66 | 4395.11 (4.25%) | 60904.75 (58.96%) | 38004.79 (36.79%) |
| Hungary | 49987.88 | 21080.28 (42.17%) | 3150.33 (6.30%) | 25757.27 (51.53%) |
| Iceland | 1534.41 | 282.64 (18.42%) | 625.98 (40.80%) | 625.78 (40.78%) |
| India | 12239181.63 | 1674205.69 (13.68%) | 6754148.31 (55.18%) | 3810827.63 (31.14%) |
| Indonesia | 1889099.65 | 363726.09 (19.25%) | 943461.41 (49.94%) | 581912.15 (30.80%) |
| Iran | 649964.97 | 96714.60 (14.88%) | 301332.55 (46.36%) | 251917.82 (38.76%) |
| Iraq | 445207.90 | 23965.54 (5.38%) | 295733.66 (66.43%) | 125508.70 (28.19%) |
| Ireland | 10834.22 | 3402.97 (31.41%) | 8794.02 (81.17%) | -1362.77 (-12.58%) |
| Israel | 49469.27 | 9558.60 (19.32%) | 37014.67 (74.82%) | 2896.00 (5.85%) |
| Italy | 136179.24 | 174781.07 (128.35%) | 73442.12 (53.93%) | -112043.95 (-82.28%) |
| Jamaica | 31298.47 | 5432.07 (17.36%) | 20704.24 (66.15%) | 5162.16 (16.49%) |
| Japan | 693634.35 | 389539.97 (56.16%) | 139512.61 (20.11%) | 164581.77 (23.73%) |
| Jordan | 117306.69 | 16891.86 (14.40%) | 92838.01 (79.14%) | 7576.82 (6.46%) |
| Kazakhstan | 81309.50 | 11673.94 (14.36%) | 19801.18 (24.35%) | 49834.38 (61.29%) |
| Kenya | 168392.49 | 4337.39 (2.58%) | 119334.50 (70.87%) | 44720.60 (26.56%) |
| Kiribati | 6897.28 | 514.25 (7.46%) | 4105.12 (59.52%) | 2277.91 (33.03%) |
| Kuwait | 52195.51 | 8918.48 (17.09%) | 28965.59 (55.49%) | 14311.44 (27.42%) |
| Kyrgyzstan | 25137.82 | 806.43 (3.21%) | 11357.24 (45.18%) | 12974.15 (51.61%) |
| Laos | 40921.97 | -308.30 (-0.75%) | 34396.71 (84.05%) | 6833.56 (16.70%) |
| Latvia | 12579.51 | 3982.93 (31.66%) | -4179.91 (-33.23%) | 12776.48 (101.57%) |
| Lebanon | 63468.38 | 7972.26 (12.56%) | 43293.67 (68.21%) | 12202.46 (19.23%) |
| Lesotho | 25885.70 | -3066.40 (-11.85%) | 9501.46 (36.71%) | 19450.64 (75.14%) |
| Liberia | 32510.41 | -4399.08 (-13.53%) | 23535.10 (72.39%) | 13374.40 (41.14%) |
| Libya | 66983.81 | 5271.20 (7.87%) | 31902.93 (47.63%) | 29809.68 (44.50%) |
| Lithuania | 15466.38 | 4905.40 (31.72%) | -2749.83 (-17.78%) | 13310.81 (86.06%) |
| Luxembourg | 2523.64 | 327.18 (12.96%) | 1970.98 (78.10%) | 225.48 (8.93%) |
| Macedonia | 32776.39 | 9135.15 (27.87%) | 9497.81 (28.98%) | 14143.44 (43.15%) |
| Madagascar | 104674.04 | -10344.94 (-9.88%) | 103389.34 (98.77%) | 11629.64 (11.11%) |
| Malawi | 73161.74 | -4537.29 (-6.20%) | 65320.27 (89.28%) | 12378.76 (16.92%) |
| Malaysia | 211841.30 | 49176.90 (23.21%) | 146472.36 (69.14%) | 16192.03 (7.64%) |
| Maldives | 2026.63 | -37.46 (-1.85%) | 2677.35 (132.11%) | -613.26 (-30.26%) |
| Mali | 149805.54 | -16783.83 (-11.20%) | 114304.44 (76.30%) | 52284.93 (34.90%) |
| Malta | -4615.47 | -396.25 (8.59%) | 2714.71 (-58.82%) | -6933.94 (150.23%) |
| Marshall Islands | 2253.79 | 364.21 (16.16%) | 962.99 (42.73%) | 926.59 (41.11%) |
| Mauritania | 63269.66 | 1343.74 (2.12%) | 45475.90 (71.88%) | 16450.02 (26.00%) |
| Mauritius | 49327.58 | 16453.81 (33.36%) | 12462.89 (25.27%) | 20410.88 (41.38%) |
| Mexico | 2525231.60 | 744872.55 (29.50%) | 1710495.92 (67.74%) | 69863.14 (2.77%) |
| Moldova | 21577.36 | 5913.56 (27.41%) | 334.21 (1.55%) | 15329.58 (71.04%) |
| Mongolia | 14746.98 | 1919.50 (13.02%) | 6328.67 (42.92%) | 6498.81 (44.07%) |
| Montenegro | 5742.32 | 1994.78 (34.74%) | 912.36 (15.89%) | 2835.19 (49.37%) |
| Morocco | 592058.05 | 82419.64 (13.92%) | 213927.22 (36.13%) | 295711.19 (49.95%) |
| Mozambique | 114962.59 | -20126.63 (-17.51%) | 89217.28 (77.61%) | 45871.94 (39.90%) |
| Myanmar | 719883.51 | 186781.53 (25.95%) | 437032.93 (60.71%) | 96069.04 (13.35%) |
| Namibia | 22523.94 | 394.13 (1.75%) | 16588.08 (73.65%) | 5541.73 (24.60%) |
| Nepal | 277608.73 | 30422.19 (10.96%) | 142499.18 (51.33%) | 104687.36 (37.71%) |
| Netherlands | 32341.91 | 37403.69 (115.65%) | 22101.60 (68.34%) | -27163.37 (-83.99%) |
| New Zealand | 36249.11 | 4428.24 (12.22%) | 25410.25 (70.10%) | 6410.61 (17.68%) |
| Nicaragua | 58519.59 | 8961.38 (15.31%) | 34004.94 (58.11%) | 15553.26 (26.58%) |
| Niger | 276813.24 | -6476.84 (-2.34%) | 206472.88 (74.59%) | 76817.20 (27.75%) |
| Nigeria | 589717.16 | -104856.41 (-17.78%) | 597785.38 (101.37%) | 96788.18 (16.41%) |
| North Korea | 155748.58 | 34028.71 (21.85%) | 65075.16 (41.78%) | 56644.71 (36.37%) |
| Northern Mariana Islands | 793.94 | 567.16 (71.44%) | 113.76 (14.33%) | 113.02 (14.24%) |
| Norway | 11867.58 | 2341.75 (19.73%) | 7435.74 (62.66%) | 2090.09 (17.61%) |
| Oman | 27843.70 | -496.44 (-1.78%) | 21874.69 (78.56%) | 6465.45 (23.22%) |
| Pakistan | 2058172.90 | -139517.57 (-6.78%) | 1308719.45 (63.59%) | 888971.02 (43.19%) |
| Palestine | 48264.06 | -1419.40 (-2.94%) | 39525.13 (81.89%) | 10158.33 (21.05%) |
| Panama | 42680.64 | 8379.81 (19.63%) | 21115.10 (49.47%) | 13185.74 (30.89%) |
| Papua New Guinea | 127070.34 | 2875.15 (2.26%) | 105068.86 (82.69%) | 19126.34 (15.05%) |
| Paraguay | 87528.34 | 7830.19 (8.95%) | 41759.09 (47.71%) | 37939.05 (43.34%) |
| Peru | 176780.61 | 31976.10 (18.09%) | 97193.51 (54.98%) | 47610.99 (26.93%) |
| Philippines | 1078283.45 | 162937.59 (15.11%) | 632715.48 (58.68%) | 282630.37 (26.21%) |
| Poland | 230802.18 | 99281.51 (43.02%) | 63047.09 (27.32%) | 68473.58 (29.67%) |
| Portugal | 76363.42 | 42321.98 (55.42%) | 29196.07 (38.23%) | 4845.37 (6.35%) |
| Principality of Monaco | 191.28 | 18.97 (9.92%) | 49.08 (25.66%) | 123.23 (64.42%) |
| Puerto Rico | 58671.91 | 38276.34 (65.24%) | 12744.65 (21.72%) | 7650.92 (13.04%) |
| Qatar | 27637.50 | 1719.10 (6.22%) | 21340.45 (77.22%) | 4577.94 (16.56%) |
| Republic of Nauru | 174.17 | 13.76 (7.90%) | 54.34 (31.20%) | 106.07 (60.90%) |
| Republic of Niue | 41.82 | 10.56 (25.25%) | -10.41 (-24.89%) | 41.67 (99.64%) |
| Republic of Palau | 610.21 | 215.04 (35.24%) | 215.29 (35.28%) | 179.88 (29.48%) |
| Republic of San Marino | 158.60 | 41.36 (26.08%) | 115.71 (72.96%) | 1.53 (0.96%) |
| Romania | 56035.63 | 34580.40 (61.71%) | -8303.60 (-14.82%) | 29758.83 (53.11%) |
| Russian Federation | 1299040.16 | 214959.89 (16.55%) | 56044.89 (4.31%) | 1028035.38 (79.14%) |
| Rwanda | 37871.63 | 2962.50 (7.82%) | 61818.12 (163.23%) | -26908.98 (-71.05%) |
| Saint Kitts and Nevis | 885.53 | 109.47 (12.36%) | 900.62 (101.70%) | -124.56 (-14.07%) |
| Saint Lucia | 3809.68 | 1695.15 (44.50%) | 3235.64 (84.93%) | -1121.11 (-29.43%) |
| Saint Vincent and the Grenadines | 2189.40 | 1132.96 (51.75%) | 1284.05 (58.65%) | -227.61 (-10.40%) |
| Samoa | 3080.84 | 496.28 (16.11%) | 1274.71 (41.38%) | 1309.85 (42.52%) |
| Sao Tome and Principe | 752.54 | -126.84 (-16.86%) | 503.88 (66.96%) | 375.51 (49.90%) |
| Saudi Arabia | 461421.32 | 17255.23 (3.74%) | 254340.80 (55.12%) | 189825.30 (41.14%) |
| Senegal | 93259.07 | 313.45 (0.34%) | 59826.45 (64.15%) | 33119.17 (35.51%) |
| Serbia | 79188.15 | 35202.84 (44.45%) | 6904.58 (8.72%) | 37080.72 (46.83%) |
| Seychelles | 1831.93 | 220.39 (12.03%) | 694.81 (37.93%) | 916.72 (50.04%) |
| Sierra Leone | 31288.50 | -4195.19 (-13.41%) | 21743.19 (69.49%) | 13740.50 (43.92%) |
| Singapore | 40472.06 | 18124.50 (44.78%) | 34503.65 (85.25%) | -12156.08 (-30.04%) |
| Slovakia | 23322.88 | 10532.81 (45.16%) | 9516.44 (40.80%) | 3273.63 (14.04%) |
| Slovenia | 9249.39 | 5892.22 (63.70%) | 2616.04 (28.28%) | 741.13 (8.01%) |
| Solomon Islands | 10362.31 | 482.02 (4.65%) | 6743.10 (65.07%) | 3137.18 (30.27%) |
| Somalia | 66670.35 | -4080.70 (-6.12%) | 62927.43 (94.39%) | 7823.62 (11.73%) |
| South Africa | 747212.79 | 97185.30 (13.01%) | 351185.03 (47.00%) | 298842.45 (39.99%) |
| South Korea | 578168.73 | 279284.12 (48.30%) | 206689.60 (35.75%) | 92195.01 (15.95%) |
| South Sudan | 31177.42 | -1273.89 (-4.09%) | 20637.87 (66.19%) | 11813.44 (37.89%) |
| Spain | 228574.59 | 127721.30 (55.88%) | 153294.62 (67.07%) | -52441.34 (-22.94%) |
| Sri Lanka | 393777.56 | 130403.62 (33.12%) | 121643.12 (30.89%) | 141730.82 (35.99%) |
| Sudan | 157752.48 | -12350.45 (-7.83%) | 97597.79 (61.87%) | 72505.14 (45.96%) |
| Suriname | 9731.01 | 2236.52 (22.98%) | 4478.18 (46.02%) | 3016.31 (31.00%) |
| Swaziland | 12978.99 | 553.21 (4.26%) | 6834.64 (52.66%) | 5591.14 (43.08%) |
| Sweden | 31214.41 | 7593.48 (24.33%) | 12456.85 (39.91%) | 11164.08 (35.77%) |
| Switzerland | 37463.74 | 13461.48 (35.93%) | 19641.76 (52.43%) | 4360.50 (11.64%) |
| Syria | 105937.20 | 35005.13 (33.04%) | 37332.31 (35.24%) | 33599.76 (31.72%) |
| Taiwan (Province of China) | 278732.64 | 177764.82 (63.78%) | 133306.36 (47.83%) | -32338.55 (-11.60%) |
| Tajikistan | 32730.53 | 650.96 (1.99%) | 21288.95 (65.04%) | 10790.61 (32.97%) |
| Tanzania | 173967.27 | -7549.85 (-4.34%) | 158086.38 (90.87%) | 23430.74 (13.47%) |
| Thailand | 640124.60 | 65177.74 (10.18%) | 319495.33 (49.91%) | 255451.53 (39.91%) |
| The Bahamas | 5686.92 | 1928.71 (33.91%) | 3472.89 (61.07%) | 285.32 (5.02%) |
| The Gambia | 15217.92 | 416.44 (2.74%) | 9225.79 (60.62%) | 5575.69 (36.64%) |
| Timor-Leste | 9077.29 | 2087.97 (23.00%) | 3763.52 (41.46%) | 3225.81 (35.54%) |
| Togo | 33909.93 | 2407.58 (7.10%) | 21539.01 (63.52%) | 9963.34 (29.38%) |
| Tokelau | 15.49 | 2.93 (18.91%) | 1.10 (7.13%) | 11.45 (73.94%) |
| Tonga | 1779.20 | 329.44 (18.52%) | 615.22 (34.58%) | 834.55 (46.91%) |
| Trinidad and Tobago | 32927.36 | 19391.58 (58.89%) | 19086.44 (57.97%) | -5550.65 (-16.86%) |
| Tunisia | 122483.14 | 22685.85 (18.52%) | 44340.02 (36.20%) | 55457.27 (45.28%) |
| Turkmenistan | 30922.70 | 4032.34 (13.04%) | 10988.51 (35.54%) | 15901.85 (51.42%) |
| Tuvalu | 170.92 | 20.00 (11.70%) | 88.92 (52.02%) | 61.99 (36.27%) |
| Uganda | 170945.85 | -15763.65 (-9.22%) | 153301.48 (89.68%) | 33408.02 (19.54%) |
| Ukraine | 94525.21 | 25783.42 (27.28%) | -18314.11 (-19.37%) | 87055.90 (92.10%) |
| United Arab Emirates | 117502.83 | 28165.91 (23.97%) | 82971.59 (70.61%) | 6365.32 (5.42%) |
| United Kingdom | 312107.82 | 52382.68 (16.78%) | 104031.54 (33.33%) | 155693.59 (49.88%) |
| United States | 3666774.48 | 817915.09 (22.31%) | 1216701.88 (33.18%) | 1632157.51 (44.51%) |
| Uruguay | 18106.43 | 3159.34 (17.45%) | 5285.20 (29.19%) | 9661.90 (53.36%) |
| Uzbekistan | 349055.89 | 36157.91 (10.36%) | 137359.59 (39.35%) | 175538.39 (50.29%) |
| Vanuatu | 4420.95 | 457.40 (10.35%) | 2684.77 (60.73%) | 1278.78 (28.93%) |
| Venezuela | 442296.66 | 143429.66 (32.43%) | 194091.48 (43.88%) | 104775.52 (23.69%) |
| Vietnam | 1014693.31 | 212317.08 (20.92%) | 550970.01 (54.30%) | 251406.22 (24.78%) |
| Virgin Islands, U.S. | 1797.59 | 1317.66 (73.30%) | 136.02 (7.57%) | 343.91 (19.13%) |
| Yemen | 94613.06 | -2796.95 (-2.96%) | 65749.49 (69.49%) | 31660.52 (33.46%) |
| Zambia | 101755.55 | -7218.14 (-7.09%) | 95874.72 (94.22%) | 13098.97 (12.87%) |
| Zimbabwe | 127988.75 | -903.28 (-0.71%) | 59119.94 (46.19%) | 69772.09 (54.51%) |

a. Change in DALYs numbers between 1990 and 2021.a is the sum of b, c, and d

b-d. Change in DALYs numbers due to change in the age structure, population number and epidemiologic changes, respectively. SDI, socio-demographic index. DALYs, disability-adjusted life years.

# Supplementary Table 14. Percentage contribution of major risk factors to type 2 diabetes mellitus age-standardized mortality by sex and SDI quintiles, 1990-2021.

| **Risk** | **1990 PAF (95%UI)** | **2021 PAF (95%UI)** | **Percentage change in PAF, 1990-2021**  **(95%UI)** |
| --- | --- | --- | --- |
| **High fasting plasma glucose** | | | |
| **Global** | 100.0% (99.6 to 100.1) | 100.0% (99.8 to 100.1) | 0.0% (-0.2 to 0.3) |
| **Male** | 100.0% (99.6 to 100.2) | 100.0% (99.8 to 100.2) | 0.0% (-0.3 to 0.4) |
| **Female** | 100.0% (99.6 to 100.1) | 100.0% (99.8 to 100.2) | 0.0% (-0.2 to 0.4) |
| **High-SDI quintile** | 100.0% (99.8 to 100.1) | 100.0% (99.7 to 100.1) | 0.0% (-0.2 to 0.1) |
| **High-middle-SDI quintile** | 100.0% (99.7 to 100.2) | 100.0% (99.8 to 100.2) | 0.0% (-0.3 to 0.3) |
| **Middle-SDI quintile** | 100.0% (99.7 to 100.2) | 100.0% (99.8 to 100.1) | 0.0% (-0.2 to 0.3) |
| **Low-middle-SDI quintile** | 100.0% (99.7 to 100.2) | 100.0% (99.8 to 100.2) | 0.0% (-0.3 to 0.4) |
| **Low-SDI quintile** | 100.0% (99.6 to 100.3) | 100.0% (99.6 to 100.2) | 0.0% (-0.4 to 0.4) |
| **High body-mass index** | | | |
| **Global** | 36.5% (14.7 to 55.3) | 44.5% (19.0 to 65.2) | 21.8% (16.3 to 28.8) |
| **Male** | 32.5% (13.0 to 49.9) | 41.7% (17.6 to 61.8) | 28.5% (21.5 to 37.6) |
| **Female** | 39.6% (16.0 to 59.5) | 46.9% (20.2 to 68.1) | 18.3% (13.2 to 25.3) |
| **High-SDI quintile** | 46.6% (19.6 to 68.8) | 54.5% (24.8 to 76.6) | 17.0% (10.9 to 26.7) |
| **High-middle-SDI quintile** | 43.1% (17.5 to 64.8) | 50.5% (21.6 to 73.1) | 17.3% (12.8 to 23.1) |
| **Middle-SDI quintile** | 34.2% (13.8 to 51.8) | 44.6% (19.0 to 65.5) | 30.3% (23.9 to 37.8) |
| **Low-middle-SDI quintile** | 25.8% (9.9 to 40.3) | 37.1% (15.2 to 56.0) | 43.8% (33.7 to 56.1) |
| **Low-SDI quintile** | 24.0% (9.2 to 39.0) | 32.1% (12.6 to 50.5) | 34.0% (26.3 to 43.2) |
| **Low physical activity** | | | |
| **Global** | 9.4% (4.1 to 14.4) | 9.4% (4.0 to 14.3) | 0.1% (-5.4 to 6.0) |
| **Male** | 7.2% (2.9 to 10.8) | 7.4% (3.2 to 11.4) | 3.5% (-5.6 to 14.2) |
| **Female** | 11.2% (4.9 to 16.9) | 11.3% (5.0 to 17.0) | 1.2% (-5.2 to 7.5) |
| **High-SDI quintile** | 10.5% (4.6 to 16.0) | 9.9% (4.2 to 15.1) | -5.3% (-14.2 to 4.1) |
| **High-middle-SDI quintile** | 10.5% (4.5 to 16.0) | 10.8% (4.8 to 16.4) | 2.6% (-6.3 to 12.8) |
| **Middle-SDI quintile** | 9.9% (4.2 to 15.2) | 10.1% (4.4 to 15.3) | 2.0% (-5.1 to 9.4) |
| **Low-middle-SDI quintile** | 8.7% (3.7 to 13.4) | 9.4% (4.0 to 14.2) | 7.3% (-3.1 to 19.1) |
| **Low-SDI quintile** | 5.9% (2.5 to 9.2) | 6.5% (2.7 to 9.9) | 9.6% (-0.1 to 21.2) |
| **Diet high in processed meat** | | | |
| **Global** | 8.6% (2.0 to 14.1) | 7.2% (1.7 to 11.9) | -16.9% (-20.4 to -13.1) |
| **Male** | 8.0% (1.9 to 13.0) | 7.0% (1.7 to 11.6) | -12.5% (-17.8 to -6.9) |
| **Female** | 9.1% (2.2 to 14.8) | 7.3% (1.8 to 12.1) | -19.2% (-22.7 to -15.4) |
| **High-SDI quintile** | 17.6% (4.2 to 28.4) | 17.2% (4.3 to 27.7) | -1.9% (-3.8 to 0.3) |
| **High-middle-SDI quintile** | 11.3% (2.7 to 18.3) | 10.6% (2.5 to 17.3) | -6.2% (-11.4 to -0.5) |
| **Middle-SDI quintile** | 3.2% (0.7 to 5.6) | 3.9% (0.9 to 6.8) | 21.3% (14.6 to 28.2) |
| **Low-middle-SDI quintile** | 4.3% (1.0 to 7.2) | 4.9% (1.2 to 8.4) | 14.1% (4.6 to 24.3) |
| **Low-SDI quintile** | 5.8% (1.4 to 9.7) | 6.3% (1.5 to 10.6) | 7.2% (0.7 to 13.2) |
| **Smoking** | | | |
| **Global** | 7.7% (6.3 to 9.1) | 5.7% (4.7 to 6.9) | -25.5% (-29.4 to -21.2) |
| **Male** | 12.5% (10.5 to 14.7) | 9.6% (8.0 to 11.5) | -23.0% (-26.3 to -19.7) |
| **Female** | 3.6% (2.9 to 4.3) | 1.9% (1.6 to 2.4) | -45.3% (-48.9 to -41.7) |
| **High-SDI quintile** | 9.3% (7.6 to 11.4) | 7.3% (5.7 to 9.1) | -22.2% (-26.9 to -17.6) |
| **High-middle-SDI quintile** | 8.0% (6.7 to 9.4) | 6.8% (5.6 to 8.1) | -14.9% (-22.3 to -7.3) |
| **Middle-SDI quintile** | 7.6% (6.3 to 8.9) | 5.5% (4.6 to 6.5) | -28.0% (-34.1 to -21.9) |
| **Low-middle-SDI quintile** | 7.3% (6.1 to 8.6) | 5.4% (4.4 to 6.3) | -27.0% (-34.0 to -19.0) |
| **Low-SDI quintile** | 4.2% (3.5 to 5.1) | 3.4% (2.8 to 4.2) | -18.5% (-26.1 to -10.3) |
| **Diet low in whole grains** | | | |
| **Global** | 7.3% (2.1 to 11.8) | 6.4% (1.8 to 10.4) | -12.1% (-14.5 to -9.8) |
| **Male** | 7.7% (2.2 to 12.3) | 6.8% (2.0 to 11.1) | -10.5% (-14.0 to -7.4) |
| **Female** | 6.9% (2.0 to 11.3) | 6.0% (1.7 to 9.7) | -14.2% (-17.6 to -10.9) |
| **High-SDI quintile** | 6.2% (1.8 to 10.2) | 7.0% (2.0 to 11.6) | 13.6% (8.5 to 18.7) |
| **High-middle-SDI quintile** | 13.5% (3.9 to 21.5) | 11.1% (3.2 to 17.9) | -17.7% (-21.2 to -14.3) |
| **Middle-SDI quintile** | 4.8% (1.4 to 7.9) | 4.7% (1.3 to 7.7) | -2.2% (-7.4 to 2.5) |
| **Low-middle-SDI quintile** | 6.1% (1.8 to 10.0) | 5.5% (1.5 to 8.9) | -10.5% (-15.9 to -4.3) |
| **Low-SDI quintile** | 7.8% (2.2 to 12.7) | 6.8% (2.0 to 11.5) | -11.7% (-16.6 to -6.4) |
| **Secondhand smoke** | | | |
| **Global** | 5.5% (2.0 to 9.1) | 4.6% (1.7 to 7.5) | -17.1% (-19.9 to -14.4) |
| **Male** | 4.4% (1.6 to 7.3) | 3.9% (1.4 to 6.5) | -9.7% (-13.4 to -5.9) |
| **Female** | 6.5% (2.4 to 10.7) | 5.2% (1.9 to 8.5) | -20.3% (-23.7 to -16.8) |
| **High-SDI quintile** | 3.8% (1.4 to 6.4) | 3.0% (1.1 to 5.0) | -21.5% (-25.0 to -18.0) |
| **High-middle-SDI quintile** | 6.5% (2.3 to 10.7) | 5.5% (2.0 to 9.0) | -15.5% (-19.9 to -11.6) |
| **Middle-SDI quintile** | 6.9% (2.6 to 11.3) | 4.9% (1.8 to 8.1) | -28.6% (-31.7 to -25.6) |
| **Low-middle-SDI quintile** | 6.1% (2.2 to 9.9) | 5.0% (1.8 to 8.1) | -19.0% (-22.6 to -15.7) |
| **Low-SDI quintile** | 3.7% (1.3 to 6.0) | 3.2% (1.2 to 5.4) | -11.1% (-14.9 to -7.0) |
| **Diet low in fruits** | | | |
| **Global** | 5.2% (0.8 to 9.2) | 4.8% (0.7 to 8.2) | -8.2% (-12.4 to -3.8) |
| **Male** | 5.4% (0.8 to 9.6) | 4.7% (0.7 to 8.1) | -12.8% (-18.2 to -6.2) |
| **Female** | 5.0% (0.8 to 8.9) | 4.8% (0.8 to 8.4) | -4.4% (-9.3 to 1.4) |
| **High-SDI quintile** | 3.4% (0.6 to 6.2) | 2.6% (0.4 to 4.7) | -25.0% (-30.8 to -18.5) |
| **High-middle-SDI quintile** | 3.1% (0.5 to 5.7) | 2.3% (0.4 to 4.0) | -27.8% (-34.5 to -19.8) |
| **Middle-SDI quintile** | 5.3% (0.8 to 9.4) | 4.2% (0.7 to 7.4) | -19.8% (-23.9 to -15.2) |
| **Low-middle-SDI quintile** | 8.0% (1.2 to 14.1) | 7.1% (1.1 to 12.4) | -10.5% (-14.1 to -6.7) |
| **Low-SDI quintile** | 7.0% (1.0 to 12.3) | 7.0% (1.1 to 12.2) | 0.1% (-3.7 to 3.9) |
| **Diet high in red meat** | | | |
| **Global** | 4.6% (-0.7 to 10.2) | 4.3% (-0.6 to 9.6) | -5.7% (-12.9 to 6.0) |
| **Male** | 4.2% (-0.6 to 9.5) | 4.4% (-0.6 to 9.8) | 3.6% (-5.8 to 22.8) |
| **Female** | 4.8% (-0.7 to 10.7) | 4.2% (-0.6 to 9.4) | -12.1% (-21.2 to -0.6) |
| **High-SDI quintile** | 8.4% (-1.3 to 18.6) | 8.7% (-1.3 to 19.1) | 2.8% (-1.6 to 18.3) |
| **High-middle-SDI quintile** | 6.8% (-1.0 to 14.9) | 6.8% (-1.0 to 15.1) | 1.0% (-6.0 to 16.6) |
| **Middle-SDI quintile** | 3.5% (-0.5 to 7.9) | 4.4% (-0.6 to 9.9) | 27.8% (17.3 to 197.8) |
| **Low-middle-SDI quintile** | 1.3% (-0.2 to 3.0) | 1.7% (-0.2 to 3.9) | 32.3% (20.2 to 362.5) |
| **Low-SDI quintile** | 1.2% (-0.2 to 2.8) | 1.3% (-0.2 to 3.0) | 5.8% (-7.8 to 430.2) |
| **Diet high in sugar-sweetened beverages** | | | |
| **Global** | 2.8% (1.5 to 4.1) | 3.3% (1.8 to 4.9) | 18.3% (10.9 to 26.9) |
| **Male** | 2.5% (1.3 to 3.6) | 3.2% (1.7 to 4.7) | 30.9% (18.5 to 43.8) |
| **Female** | 3.1% (1.6 to 4.4) | 3.4% (1.8 to 5.0) | 11.3% (2.5 to 20.8) |
| **High-SDI quintile** | 5.3% (2.6 to 7.6) | 7.2% (3.8 to 10.4) | 37.1% (22.2 to 52.8) |
| **High-middle-SDI quintile** | 3.5% (1.8 to 5.1) | 3.9% (2.1 to 5.7) | 13.8% (2.5 to 24.6) |
| **Middle-SDI quintile** | 2.4% (1.3 to 3.5) | 3.5% (1.8 to 5.1) | 46.1% (29.8 to 67.9) |
| **Low-middle-SDI quintile** | 1.0% (0.5 to 1.5) | 1.8% (0.9 to 2.7) | 78.6% (63.5 to 94.2) |
| **Low-SDI quintile** | 0.8% (0.4 to 1.1) | 0.9% (0.5 to 1.4) | 25.2% (12.9 to 38.8) |
| **Diet low in vegetables** | | | |
| **Global** | 1.8% (-0.7 to 3.9) | 1.0% (-0.4 to 2.2) | -45.4% (-50.0 to -40.8) |
| **Male** | 2.1% (-0.8 to 4.6) | 1.0% (-0.4 to 2.2) | -53.2% (-58.4 to -47.7) |
| **Female** | 1.6% (-0.6 to 3.5) | 1.0% (-0.4 to 2.2) | -37.7% (-43.7 to -31.3) |
| **High-SDI quintile** | 0.3% (-0.1 to 0.6) | 0.2% (-0.1 to 0.5) | -17.5% (-28.2 to -6.4) |
| **High-middle-SDI quintile** | 0.2% (-0.1 to 0.5) | 0.1% (-0.0 to 0.2) | -56.4% (-62.9 to -50.3) |
| **Middle-SDI quintile** | 1.1% (-0.4 to 2.6) | 0.3% (-0.1 to 0.7) | -71.6% (-75.2 to -67.9) |
| **Low-middle-SDI quintile** | 3.7% (-1.3 to 7.8) | 1.5% (-0.6 to 3.3) | -59.2% (-64.8 to -53.5) |
| **Low-SDI quintile** | 7.2% (-2.7 to 15.2) | 5.1% (-1.9 to 10.9) | -29.3% (-34.5 to -24.5) |
| **Alcohol use** | | | |
| **Global** | 1.7% (0.6 to 3.1) | 1.8% (0.6 to 3.4) | 6.4% (-6.6 to 29.9) |
| **Male** | 2.7% (1.0 to 4.9) | 3.2% (1.2 to 5.9) | 16.0% (0.2 to 43.5) |
| **Female** | 0.8% (0.2 to 1.6) | 0.5% (0.1 to 1.0) | -40.9% (-56.6 to -27.4) |
| **High-SDI quintile** | 3.5% (1.2 to 6.4) | 3.9% (1.3 to 7.3) | 12.5% (-5.8 to 30.2) |
| **High-middle-SDI quintile** | 2.7% (1.0 to 4.7) | 2.1% (0.8 to 4.0) | -19.8% (-30.1 to -8.3) |
| **Middle-SDI quintile** | 0.9% (0.3 to 1.9) | 1.7% (0.6 to 3.1) | 76.5% (40.7 to 190.1) |
| **Low-middle-SDI quintile** | 0.4% (-0.1 to 1.0) | 1.0% (0.3 to 1.9) | 168.3% (-772.8 to 2635.7) |
| **Low-SDI quintile** | 0.2% (-0.5 to 1.3) | 0.8% (0.1 to 1.9) | 220.3% (-2169.0 to 1756.9) |
| **Diet low in fiber** | | | |
| **Global** | 1.4% (0.8 to 2.0) | 1.1% (0.6 to 1.5) | -22.9% (-26.4 to -19.6) |
| **Male** | 1.4% (0.8 to 2.0) | 1.0% (0.6 to 1.5) | -24.9% (-29.3 to -20.8) |
| **Female** | 1.4% (0.8 to 2.0) | 1.1% (0.6 to 1.6) | -21.1% (-25.3 to -16.7) |
| **High-SDI quintile** | 1.3% (0.8 to 1.9) | 1.1% (0.6 to 1.5) | -19.8% (-24.5 to -15.2) |
| **High-middle-SDI quintile** | 1.0% (0.6 to 1.5) | 0.8% (0.5 to 1.2) | -22.8% (-28.8 to -16.5) |
| **Middle-SDI quintile** | 1.5% (0.8 to 2.1) | 1.1% (0.6 to 1.5) | -28.7% (-33.3 to -23.7) |
| **Low-middle-SDI quintile** | 1.9% (1.1 to 2.7) | 1.4% (0.8 to 2.0) | -28.0% (-33.0 to -22.7) |
| **Low-SDI quintile** | 0.9% (0.5 to 1.3) | 0.8% (0.5 to 1.2) | -5.7% (-13.0 to 2.3) |
| **Household air pollution from solid fuels** | | | |
| **Global** | 9.1% (5.3 to 13.5) | 5.9% (2.9 to 10.2) | -34.8% (-51.2 to -16.3) |
| **Male** | 9.3% (5.4 to 13.7) | 5.5% (2.7 to 9.5) | -41.2% (-56.4 to -21.8) |
| **Female** | 9.0% (5.2 to 13.3) | 6.4% (3.1 to 11.0) | -29.5% (-46.8 to -10.0) |
| **High-SDI quintile** | 0.7% (0.2 to 1.8) | 0.0% (0.0 to 0.4) | -94.0% (-100.0 to -80.1) |
| **High-middle-SDI quintile** | 5.3% (2.6 to 9.6) | 0.7% (0.0 to 3.6) | -86.5% (-98.7 to -58.0) |
| **Middle-SDI quintile** | 11.1% (6.3 to 16.7) | 3.9% (1.2 to 9.6) | -65.1% (-86.1 to -36.8) |
| **Low-middle-SDI quintile** | 15.8% (9.7 to 22.7) | 10.7% (5.6 to 17.1) | -32.1% (-50.3 to -15.8) |
| **Low-SDI quintile** | 17.5% (10.9 to 24.9) | 16.6% (10.2 to 23.9) | -4.8% (-9.9 to 0.2) |
| **Ambient particulate matter pollution** | | | |
| **Global** | 9.3% (5.4 to 13.6) | 11.5% (6.7 to 17.0) | 23.7% (6.8 to 41.4) |
| **Male** | 9.2% (5.3 to 13.4) | 11.9% (6.8 to 17.6) | 29.5% (12.7 to 46.8) |
| **Female** | 9.3% (5.4 to 13.5) | 11.2% (6.3 to 16.4) | 19.8% (1.6 to 38.7) |
| **High-SDI quintile** | 14.9% (8.2 to 22.1) | 10.7% (6.0 to 16.5) | -28.0% (-43.1 to -9.8) |
| **High-middle-SDI quintile** | 13.0% (7.6 to 19.1) | 15.8% (9.0 to 22.5) | 21.2% (5.8 to 44.4) |
| **Middle-SDI quintile** | 8.3% (4.5 to 12.2) | 14.4% (8.0 to 20.9) | 72.8% (50.7 to 100.9) |
| **Low-middle-SDI quintile** | 4.0% (2.2 to 6.2) | 8.6% (4.4 to 13.7) | 112.7% (58.1 to 172.0) |
| **Low-SDI quintile** | 2.7% (1.5 to 4.3) | 3.6% (2.0 to 5.7) | 30.4% (-0.8 to 62.7) |
| **Low temperature** | | | |
| **Global** | 4.9% (4.0 to 6.1) | 4.0% (3.1 to 5.3) | -18.0% (-23.9 to -11.4) |
| **Male** | 4.8% (3.8 to 6.0) | 4.1% (3.2 to 5.3) | -14.1% (-19.0 to -9.1) |
| **Female** | 5.1% (4.2 to 6.1) | 4.0% (3.0 to 5.3) | -20.7% (-28.3 to -13.0) |
| **High-SDI quintile** | 6.9% (5.9 to 7.8) | 6.6% (5.7 to 7.5) | -4.2% (-8.7 to 0.8) |
| **High-middle-SDI quintile** | 6.8% (5.8 to 7.9) | 6.0% (4.9 to 7.1) | -12.3% (-16.7 to -7.9) |
| **Middle-SDI quintile** | 4.1% (3.2 to 5.3) | 3.6% (2.9 to 4.7) | -12.4% (-18.9 to -6.6) |
| **Low-middle-SDI quintile** | 3.0% (1.3 to 4.9) | 2.8% (1.2 to 4.6) | -8.0% (-14.4 to 0.3) |
| **Low-SDI quintile** | 2.9% (1.8 to 4.1) | 2.6% (1.6 to 3.8) | -8.4% (-18.1 to 13.6) |
| **High temperature** | | | |
| **Global** | 1.5% (0.5 to 2.7) | 2.5% (0.8 to 4.4) | 63.2% (52.9 to 84.8) |
| **Male** | 1.7% (0.5 to 3.0) | 2.5% (0.8 to 4.4) | 47.3% (33.1 to 64.9) |
| **Female** | 1.4% (0.4 to 2.6) | 2.5% (0.8 to 4.4) | 76.4% (63.8 to 110.4) |
| **High-SDI quintile** | 0.5% (-0.3 to 1.5) | 1.2% (-0.2 to 2.8) | 137.0% (-315.3 to 759.0) |
| **High-middle-SDI quintile** | 0.5% (-0.2 to 1.3) | 0.9% (-0.2 to 2.4) | 90.8% (-98.5 to 313.5) |
| **Middle-SDI quintile** | 1.5% (0.6 to 2.6) | 2.2% (0.8 to 3.7) | 42.5% (33.0 to 52.5) |
| **Low-middle-SDI quintile** | 3.3% (1.4 to 5.5) | 4.2% (1.6 to 7.2) | 25.4% (10.2 to 35.2) |
| **Low-SDI quintile** | 2.2% (1.1 to 3.5) | 3.0% (1.4 to 5.0) | 39.8% (16.2 to 68.0) |

PAF, population attributable fraction; UI, uncertainty interval; SDI, socio-demographic index.

# Supplementary Table 15. Percentage contribution of major risk factors to type 2 diabetes mellitus age-standardized DALYs by sex and SDI quintiles, 1990-2021.

| **Risk** | **1990 PAF (95%UI)** | **2021 PAF (95%UI)** | **Percentage change in PAF, 1990-2021**  **(95%UI)** |
| --- | --- | --- | --- |
| **High fasting plasma glucose** | | | |
| **Global** | 100.0% (99.8 to 100.1) | 100.0% (99.9 to 100.1) | 0.0% (-0.1 to 0.2) |
| **Male** | 100.0% (99.8 to 100.1) | 100.0% (99.9 to 100.1) | 0.0% (-0.2 to 0.2) |
| **Female** | 100.0% (99.8 to 100.1) | 100.0% (99.9 to 100.1) | 0.0% (-0.1 to 0.2) |
| **High-SDI quintile** | 100.0% (99.9 to 100.0) | 100.0% (99.9 to 100.0) | 0.0% (-0.1 to 0.1) |
| **High-middle-SDI quintile** | 100.0% (99.9 to 100.1) | 100.0% (99.9 to 100.1) | 0.0% (-0.1 to 0.2) |
| **Middle-SDI quintile** | 100.0% (99.8 to 100.1) | 100.0% (99.9 to 100.1) | 0.0% (-0.1 to 0.2) |
| **Low-middle-SDI quintile** | 100.0% (99.8 to 100.1) | 100.0% (99.9 to 100.1) | 0.0% (-0.2 to 0.2) |
| **Low-SDI quintile** | 100.0% (99.8 to 100.2) | 100.0% (99.7 to 100.2) | 0.0% (-0.3 to 0.3) |
| **High body-mass index** | | | |
| **Global** | 41.1% (18.0 to 59.6) | 51.9% (24.7 to 71.5) | 26.4% (20.0 to 34.8) |
| **Male** | 37.7% (16.6 to 55.3) | 49.5% (23.3 to 68.9) | 31.5% (24.3 to 41.0) |
| **Female** | 44.1% (19.4 to 63.3) | 54.2% (26.1 to 74.1) | 23.0% (16.8 to 31.9) |
| **High-SDI quintile** | 52.0% (24.0 to 73.3) | 61.2% (31.4 to 80.9) | 17.8% (10.6 to 29.6) |
| **High-middle-SDI quintile** | 47.5% (21.1 to 68.0) | 57.7% (28.2 to 78.3) | 21.6% (15.2 to 31.2) |
| **Middle-SDI quintile** | 39.3% (17.2 to 57.2) | 51.8% (24.5 to 71.5) | 31.6% (24.4 to 40.4) |
| **Low-middle-SDI quintile** | 30.1% (12.6 to 45.5) | 44.4% (20.0 to 63.0) | 47.6% (37.9 to 60.9) |
| **Low-SDI quintile** | 28.0% (11.4 to 43.6) | 38.7% (16.5 to 57.0) | 38.2% (31.2 to 47.1) |
| **Behavioral risks** | | | |
| **Global** | 42.5% (25.0 to 54.8) | 39.9% (23.0 to 52.4) | -6.1% (-9.6 to -3.9) |
| **Male** | 44.5% (27.4 to 56.4) | 42.0% (25.9 to 54.4) | -5.5% (-8.9 to -3.2) |
| **High-SDI quintile** | 51.7% (30.9 to 65.9) | 50.7% (28.9 to 65.9) | -1.9% (-7.5 to 0.5) |
| **High-middle-SDI quintile** | 48.6% (28.9 to 62.0) | 46.2% (27.2 to 59.8) | -5.1% (-8.0 to -3.0) |
| **Middle-SDI quintile** | 38.0% (24.7 to 48.1) | 36.6% (22.4 to 47.6) | -3.6% (-9.8 to 0.3) |
| **Low-middle-SDI quintile** | 37.5% (21.9 to 48.5) | 35.4% (20.5 to 45.9) | -5.6% (-8.8 to -2.1) |
| **Low-SDI quintile** | 35.9% (15.6 to 51.0) | 34.5% (15.8 to 48.4) | -3.9% (-6.2 to 2.2) |
| **Low physical activity** | | | |
| **Global** | 7.5% (3.2 to 11.4) | 7.4% (3.2 to 11.3) | -2.0% (-7.0 to 3.3) |
| **Male** | 5.7% (2.3 to 8.9) | 5.7% (2.4 to 8.8) | -0.6% (-8.8 to 8.7) |
| **Female** | 9.1% (3.9 to 13.8) | 9.1% (3.9 to 13.8) | -0.5% (-6.7 to 5.5) |
| **High-SDI quintile** | 8.4% (3.5 to 12.8) | 7.8% (3.3 to 11.9) | -6.5% (-14.4 to 1.4) |
| **High-middle-SDI quintile** | 7.8% (3.3 to 11.8) | 7.5% (3.3 to 11.5) | -3.6% (-12.0 to 5.4) |
| **Middle-SDI quintile** | 8.0% (3.3 to 12.2) | 8.0% (3.4 to 12.0) | -0.3% (-7.4 to 6.6) |
| **Low-middle-SDI quintile** | 7.3% (3.1 to 11.0) | 7.3% (3.1 to 11.2) | 1.0% (-7.6 to 10.1) |
| **Low-SDI quintile** | 5.1% (2.1 to 7.8) | 5.3% (2.2 to 8.2) | 5.7% (-3.0 to 15.5) |
| **Diet high in processed meat** | | | |
| **Global** | 8.3% (2.0 to 13.5) | 8.1% (2.0 to 13.5) | -2.6% (-5.3 to 0.7) |
| **Male** | 7.9% (1.9 to 13.1) | 7.9% (1.9 to 13.1) | -0.5% (-4.6 to 3.8) |
| **Female** | 8.6% (2.0 to 14.0) | 8.3% (2.0 to 13.8) | -3.5% (-7.3 to -0.1) |
| **High-SDI quintile** | 17.5% (4.3 to 28.2) | 17.9% (4.4 to 28.8) | 1.9% (0.3 to 3.5) |
| **High-middle-SDI quintile** | 10.7% (2.5 to 17.4) | 10.2% (2.4 to 16.7) | -5.1% (-8.5 to -1.9) |
| **Middle-SDI quintile** | 3.4% (0.8 to 5.7) | 4.4% (1.1 to 7.5) | 31.0% (25.0 to 37.2) |
| **Low-middle-SDI quintile** | 4.3% (1.0 to 7.1) | 5.3% (1.2 to 8.9) | 24.0% (16.4 to 32.7) |
| **Low-SDI quintile** | 6.1% (1.5 to 10.2) | 6.7% (1.6 to 11.3) | 9.6% (3.7 to 15.3) |
| **Smoking** | | | |
| **Global** | 9.4% (7.9 to 10.9) | 7.2% (6.0 to 8.4) | -23.7% (-26.1 to -21.3) |
| **Male** | 14.9% (12.6 to 17.2) | 11.6% (9.7 to 13.5) | -22.3% (-24.4 to -20.0) |
| **Female** | 4.1% (3.4 to 4.8) | 2.5% (2.1 to 3.1) | -38.0% (-41.5 to -34.4) |
| **High-SDI quintile** | 12.2% (10.1 to 14.3) | 9.1% (7.5 to 11.1) | -25.0% (-28.2 to -21.5) |
| **High-middle-SDI quintile** | 10.5% (9.0 to 12.1) | 9.5% (8.0 to 11.0) | -10.1% (-13.6 to -6.3) |
| **Middle-SDI quintile** | 9.2% (7.8 to 10.7) | 6.8% (5.7 to 7.9) | -26.6% (-30.0 to -22.9) |
| **Low-middle-SDI quintile** | 8.1% (6.8 to 9.4) | 6.1% (5.1 to 7.1) | -24.7% (-29.5 to -19.7) |
| **Low-SDI quintile** | 4.8% (4.0 to 5.6) | 4.0% (3.3 to 4.7) | -16.5% (-22.1 to -10.5) |
| **Diet low in whole grains** | | | |
| **Global** | 7.2% (2.1 to 11.7) | 6.7% (2.0 to 11.0) | -6.8% (-8.7 to -4.9) |
| **Male** | 7.6% (2.2 to 12.2) | 7.1% (2.1 to 11.7) | -5.6% (-8.3 to -3.1) |
| **Female** | 6.9% (2.0 to 11.3) | 6.3% (1.8 to 10.3) | -8.5% (-11.1 to -5.9) |
| **High-SDI quintile** | 6.1% (1.7 to 10.0) | 6.8% (1.9 to 11.3) | 12.1% (7.8 to 15.9) |
| **High-middle-SDI quintile** | 12.4% (3.6 to 19.8) | 10.4% (3.0 to 16.7) | -16.3% (-18.8 to -13.7) |
| **Middle-SDI quintile** | 5.2% (1.5 to 8.6) | 5.4% (1.6 to 8.9) | 3.6% (-0.1 to 7.3) |
| **Low-middle-SDI quintile** | 6.1% (1.7 to 10.0) | 5.8% (1.7 to 9.6) | -4.0% (-8.0 to 0.0) |
| **Low-SDI quintile** | 7.9% (2.2 to 13.0) | 7.2% (2.1 to 11.9) | -9.2% (-13.3 to -4.7) |
| **Secondhand smoke** | | | |
| **Global** | 5.9% (2.2 to 9.8) | 4.9% (1.8 to 8.0) | -17.8% (-20.0 to -15.6) |
| **Male** | 4.5% (1.6 to 7.5) | 4.1% (1.5 to 6.7) | -9.8% (-13.1 to -6.4) |
| **Female** | 7.3% (2.7 to 12.0) | 5.7% (2.1 to 9.4) | -21.8% (-24.1 to -19.7) |
| **High-SDI quintile** | 4.7% (1.7 to 7.8) | 3.6% (1.3 to 6.0) | -23.6% (-26.5 to -20.8) |
| **High-middle-SDI quintile** | 6.9% (2.5 to 11.3) | 6.1% (2.2 to 10.0) | -11.3% (-14.6 to -7.9) |
| **Middle-SDI quintile** | 6.9% (2.6 to 11.4) | 5.2% (1.9 to 8.6) | -24.5% (-27.0 to -21.9) |
| **Low-middle-SDI quintile** | 6.1% (2.2 to 10.1) | 5.1% (1.8 to 8.3) | -17.6% (-20.7 to -14.8) |
| **Low-SDI quintile** | 3.8% (1.4 to 6.3) | 3.5% (1.2 to 5.7) | -9.0% (-12.4 to -5.2) |
| **Diet low in fruits** | | | |
| **Global** | 5.5% (0.8 to 9.6) | 4.5% (0.7 to 7.8) | -18.0% (-20.7 to -15.0) |
| **Male** | 5.6% (0.9 to 9.8) | 4.5% (0.7 to 7.7) | -19.9% (-23.6 to -15.8) |
| **Female** | 5.4% (0.8 to 9.5) | 4.5% (0.7 to 7.9) | -16.3% (-19.7 to -12.3) |
| **High-SDI quintile** | 3.7% (0.6 to 6.6) | 2.8% (0.5 to 5.0) | -24.1% (-29.2 to -18.7) |
| **High-middle-SDI quintile** | 4.0% (0.6 to 7.2) | 2.4% (0.4 to 4.1) | -41.1% (-45.6 to -35.5) |
| **Middle-SDI quintile** | 5.5% (0.8 to 9.6) | 4.0% (0.6 to 7.0) | -26.6% (-29.9 to -23.3) |
| **Low-middle-SDI quintile** | 8.1% (1.2 to 14.2) | 6.9% (1.1 to 12.1) | -13.8% (-16.3 to -11.4) |
| **Low-SDI quintile** | 7.1% (1.1 to 12.6) | 7.1% (1.1 to 12.4) | -0.6% (-4.0 to 2.5) |
| **Diet high in red meat** | | | |
| **Global** | 4.7% (-0.7 to 10.6) | 5.0% (-0.7 to 11.3) | 6.8% (2.1 to 37.7) |
| **Male** | 4.6% (-0.7 to 10.2) | 5.1% (-0.8 to 11.4) | 12.7% (6.1 to 48.8) |
| **Female** | 4.8% (-0.7 to 10.8) | 4.9% (-0.7 to 11.0) | 2.0% (-4.4 to 32.3) |
| **High-SDI quintile** | 8.6% (-1.3 to 18.9) | 8.9% (-1.4 to 19.5) | 3.5% (-0.5 to 17.1) |
| **High-middle-SDI quintile** | 7.0% (-1.0 to 15.3) | 7.8% (-1.2 to 17.0) | 11.5% (4.2 to 55.6) |
| **Middle-SDI quintile** | 3.8% (-0.6 to 8.7) | 5.0% (-0.7 to 11.1) | 31.3% (21.8 to 241.7) |
| **Low-middle-SDI quintile** | 1.3% (-0.2 to 3.1) | 1.9% (-0.3 to 4.4) | 41.4% (31.8 to 375.5) |
| **Low-SDI quintile** | 1.3% (-0.2 to 3.1) | 1.4% (-0.2 to 3.4) | 9.5% (1.9 to 669.0) |
| **Diet high in sugar-sweetened beverages** | | | |
| **Global** | 2.9% (1.5 to 4.2) | 4.0% (2.1 to 5.8) | 38.4% (31.1 to 46.2) |
| **Male** | 2.6% (1.4 to 3.8) | 3.8% (2.0 to 5.6) | 46.1% (34.6 to 58.0) |
| **Female** | 3.1% (1.6 to 4.5) | 4.2% (2.2 to 6.1) | 33.6% (23.8 to 43.1) |
| **High-SDI quintile** | 5.7% (3.0 to 8.4) | 8.4% (4.5 to 12.0) | 46.0% (32.2 to 61.3) |
| **High-middle-SDI quintile** | 3.2% (1.7 to 4.6) | 4.5% (2.3 to 6.4) | 40.2% (30.2 to 50.3) |
| **Middle-SDI quintile** | 2.5% (1.3 to 3.6) | 3.9% (2.0 to 5.5) | 55.2% (41.2 to 70.3) |
| **Low-middle-SDI quintile** | 1.2% (0.6 to 1.8) | 2.1% (1.1 to 3.1) | 77.9% (65.6 to 90.3) |
| **Low-SDI quintile** | 0.8% (0.4 to 1.3) | 1.1% (0.6 to 1.6) | 28.4% (16.6 to 41.7) |
| **Diet low in vegetables** | | | |
| **Global** | 1.6% (-0.6 to 3.4) | 0.8% (-0.3 to 1.7) | -50.4% (-53.8 to -47.2) |
| **Male** | 1.8% (-0.6 to 3.8) | 0.8% (-0.3 to 1.7) | -55.4% (-59.2 to -51.4) |
| **Female** | 1.5% (-0.5 to 3.1) | 0.8% (-0.3 to 1.8) | -45.3% (-49.4 to -41.3) |
| **High-SDI quintile** | 0.2% (-0.1 to 0.5) | 0.2% (-0.1 to 0.4) | -30.7% (-39.6 to -22.7) |
| **High-middle-SDI quintile** | 0.2% (-0.1 to 0.4) | 0.1% (-0.0 to 0.2) | -64.4% (-69.4 to -59.7) |
| **Middle-SDI quintile** | 0.9% (-0.4 to 2.1) | 0.3% (-0.1 to 0.6) | -73.3% (-76.3 to -69.9) |
| **Low-middle-SDI quintile** | 3.1% (-1.1 to 6.7) | 1.1% (-0.4 to 2.5) | -63.7% (-67.8 to -60.2) |
| **Low-SDI quintile** | 6.8% (-2.5 to 14.4) | 4.8% (-1.8 to 10.3) | -29.3% (-34.0 to -25.5) |
| **Alcohol use** | | | |
| **Global** | 1.5% (0.2 to 3.1) | 1.8% (0.4 to 3.6) | 21.7% (-0.5 to 97.9) |
| **Male** | 2.5% (0.5 to 5.0) | 3.1% (1.0 to 6.1) | 24.1% (4.5 to 92.5) |
| **Female** | 0.5% (-0.0 to 1.2) | 0.4% (-0.1 to 1.1) | -16.1% (-96.8 to 31.2) |
| **High-SDI quintile** | 3.1% (0.6 to 6.4) | 3.3% (0.4 to 6.9) | 4.8% (-41.3 to 36.8) |
| **High-middle-SDI quintile** | 2.3% (0.6 to 4.4) | 2.0% (0.4 to 4.0) | -10.8% (-34.9 to 7.1) |
| **Middle-SDI quintile** | 0.9% (0.0 to 2.0) | 1.6% (0.5 to 3.2) | 89.2% (29.0 to 488.3) |
| **Low-middle-SDI quintile** | 0.3% (-0.3 to 1.1) | 1.0% (0.2 to 2.1) | 224.8% (-1875.4 to 2218.4) |
| **Low-SDI quintile** | 0.1% (-0.8 to 1.3) | 0.7% (-0.1 to 1.9) | 664.2% (-1521.1 to 1986.1) |
| **Diet low in fiber** | | | |
| **Global** | 1.4% (0.8 to 1.9) | 1.0% (0.6 to 1.5) | -23.8% (-27.2 to -20.9) |
| **Male** | 1.3% (0.8 to 1.9) | 1.0% (0.6 to 1.4) | -25.1% (-29.0 to -21.2) |
| **Female** | 1.4% (0.8 to 1.9) | 1.1% (0.6 to 1.5) | -22.5% (-26.7 to -18.8) |
| **High-SDI quintile** | 1.2% (0.7 to 1.8) | 1.0% (0.6 to 1.5) | -17.2% (-21.8 to -12.9) |
| **High-middle-SDI quintile** | 1.1% (0.6 to 1.5) | 0.8% (0.5 to 1.2) | -24.8% (-31.1 to -18.9) |
| **Middle-SDI quintile** | 1.5% (0.8 to 2.1) | 1.0% (0.6 to 1.4) | -30.2% (-34.4 to -26.2) |
| **Low-middle-SDI quintile** | 1.8% (1.0 to 2.6) | 1.3% (0.7 to 1.8) | -29.0% (-32.6 to -25.3) |
| **Low-SDI quintile** | 0.9% (0.5 to 1.3) | 0.8% (0.5 to 1.2) | -4.5% (-9.9 to 1.9) |
| **Household air pollution from solid fuels** | | | |
| **Global** | 9.5% (5.5 to 14.1) | 5.3% (2.6 to 9.3) | -44.8% (-59.2 to -26.1) |
| **Male** | 9.4% (5.4 to 13.9) | 4.8% (2.4 to 8.7) | -48.7% (-62.4 to -30.4) |
| **Female** | 9.7% (5.6 to 14.3) | 5.7% (2.8 to 10.0) | -41.2% (-56.1 to -22.2) |
| **High-SDI quintile** | 0.8% (0.2 to 2.2) | 0.0% (0.0 to 0.3) | -96.2% (-100.0 to -86.6) |
| **High-middle-SDI quintile** | 6.6% (3.4 to 11.1) | 0.8% (0.0 to 4.1) | -87.5% (-98.9 to -57.5) |
| **Middle-SDI quintile** | 11.4% (6.5 to 16.9) | 3.7% (1.1 to 9.4) | -67.7% (-88.0 to -38.6) |
| **Low-middle-SDI quintile** | 15.7% (9.6 to 22.5) | 10.5% (5.5 to 16.6) | -33.2% (-50.7 to -17.2) |
| **Low-SDI quintile** | 17.3% (10.8 to 24.6) | 16.3% (10.0 to 23.5) | -5.7% (-11.0 to -0.4) |
| **Ambient particulate matter pollution** | | | |
| **Global** | 8.8% (5.1 to 13.0) | 11.8% (6.8 to 17.3) | 33.4% (17.8 to 50.2) |
| **Male** | 9.0% (5.2 to 13.1) | 12.2% (6.9 to 17.8) | 35.0% (18.8 to 52.9) |
| **Female** | 8.6% (5.0 to 12.6) | 11.4% (6.5 to 16.7) | 32.3% (15.7 to 49.3) |
| **High-SDI quintile** | 14.5% (7.8 to 21.7) | 10.8% (6.2 to 16.7) | -25.4% (-41.2 to -7.4) |
| **High-middle-SDI quintile** | 11.9% (6.9 to 17.4) | 16.1% (9.2 to 22.7) | 34.9% (17.7 to 58.7) |
| **Middle-SDI quintile** | 7.9% (4.3 to 11.7) | 14.6% (8.2 to 21.2) | 84.2% (59.0 to 116.0) |
| **Low-middle-SDI quintile** | 4.0% (2.2 to 6.2) | 8.6% (4.6 to 13.7) | 115.5% (60.4 to 175.1) |
| **Low-SDI quintile** | 2.7% (1.5 to 4.3) | 3.7% (2.1 to 5.8) | 33.4% (0.4 to 67.3) |
| **Low temperature** | | | |
| **Global** | 2.9% (2.2 to 3.7) | 1.8% (1.3 to 2.5) | -36.9% (-42.9 to -30.6) |
| **Male** | 2.8% (2.1 to 3.6) | 1.9% (1.3 to 2.5) | -33.8% (-39.6 to -27.8) |
| **Female** | 3.0% (2.3 to 3.8) | 1.8% (1.2 to 2.5) | -39.3% (-46.4 to -31.6) |
| **High-SDI quintile** | 3.8% (3.0 to 4.4) | 1.8% (1.3 to 2.4) | -52.2% (-57.2 to -46.5) |
| **High-middle-SDI quintile** | 3.5% (2.7 to 4.2) | 2.1% (1.5 to 2.7) | -40.0% (-45.5 to -34.0) |
| **Middle-SDI quintile** | 2.6% (1.9 to 3.3) | 1.8% (1.4 to 2.5) | -28.2% (-34.4 to -22.1) |
| **Low-middle-SDI quintile** | 2.1% (0.9 to 3.4) | 1.7% (0.7 to 2.8) | -19.5% (-26.7 to -11.1) |
| **Low-SDI quintile** | 2.3% (1.4 to 3.2) | 1.7% (1.0 to 2.5) | -26.4% (-36.2 to -7.0) |
| **High temperature** | | | |
| **Global** | 1.0% (0.3 to 1.8) | 1.2% (0.4 to 2.2) | 24.8% (11.5 to 39.6) |
| **Male** | 1.0% (0.4 to 1.9) | 1.2% (0.4 to 2.3) | 17.0% (1.9 to 35.1) |
| **Female** | 1.0% (0.3 to 1.7) | 1.3% (0.4 to 2.3) | 31.6% (18.3 to 51.2) |
| **High-SDI quintile** | 0.3% (-0.2 to 0.9) | 0.4% (-0.0 to 1.0) | 38.9% (-308.1 to 383.7) |
| **High-middle-SDI quintile** | 0.3% (-0.1 to 0.7) | 0.4% (-0.1 to 0.9) | 31.9% (-139.2 to 117.3) |
| **Middle-SDI quintile** | 0.9% (0.4 to 1.6) | 1.1% (0.4 to 2.0) | 20.6% (8.8 to 32.1) |
| **Low-middle-SDI quintile** | 2.3% (1.0 to 3.8) | 2.5% (0.9 to 4.3) | 7.3% (-9.9 to 19.4) |
| **Low-SDI quintile** | 1.6% (0.9 to 2.6) | 1.9% (0.9 to 3.2) | 19.2% (-1.4 to 44.0) |

PAF, population attributable fraction; UI, uncertainty interval; SDI, socio-demographic index.
